# Burst expansion, distribution and diversification of MITEs in the silkworm genome

**DOI:** 10.1186/1471-2164-11-520

**Published:** 2010-09-27

**Authors:** Min-Jin Han, Yi-Hong Shen, Ying-Hui Gao, Li-Yong Chen, Zhong-Huai Xiang, Ze Zhang

**Affiliations:** 1 The Key Sericultural Laboratory of Agricultural Ministry, Southwest University, Chongqing 400715, China; 2 Department of Anesthesiology, Research Institute of Surgery and Daping Hospital, Third Medical University, Chongqing, 400042, China; 3 The Institute of Agricultural and Life Sciences, Chongqing University, Chongqing 400044, China

## Abstract

**Background:**

Miniature inverted-repeat transposable elements (MITEs) are widespread in plants and animals. Although silkworm (*Bombyx mori*) has a large amount of and a variety of transposable elements, the genome-wide information of the silkworm MITEs is unknown.

**Results:**

We used structure-based and homology approaches to search for MITEs in the silkworm genome. We identified 17 MITE families with a total of 5785 members, accounting for ~0.4% of the genome. 7 of 17 MITE families are completely novel based on the nucleotide composition of target site duplication (TSD) and/or terminal inverted repeats (TIR). Silkworm MITEs were widely and nonrandom distributed in the genome. One family named BmMITE-2 might experience a recent burst expansion. Network and diversity analyses for each family revealed different diversification patterns of the silkworm MITEs, reflecting the signatures of genome-shocks that silkworm experienced. Most silkworm MITEs preferentially inserted into or near genes and BmMITE-11 that encodes a germline-restricted small RNA might silence its the closest genes in silkworm ovary through a small RNA pathway.

**Conclusions:**

Silkworm harbors 17 MITE families. The silkworm MITEs preferred to reside in or near genes and one MITE might be involved in gene silence. Our results emphasize the exceptional role of MITEs in transcriptional regulation of genes and have general implications to understand interaction between MITEs and their host genome.

## Background

Since transposable elements (TEs) were first discovered in maize by McClintock [1], TEs have been a hotspot subject in genetics. As the advent of genomics era, it is now known that TEs constitute a significant component of eukaryotic genomes and that there are two major classes of TEs [2,3]. Class I elements (retrotransposon) transpose by the mechanism of 'copy-and-paste' through a RNA intermediate, while class II elements (DNA transposons) by the mechanism of 'cut-and-paste' through a DNA intermediate.

In addition to transposition through a DNA intermediate rather than a RNA intermediate, DNA transposable element is distinguished by terminal inverted repeats (TIRs). With the action of transposase, DNA transposons usually excise from one site and reinsert elsewhere in the genome. Furthermore, DNA transposons can be also further classified into autonomous and non-autonomous. Of non-autonomous DNA transposons, a group of miniature inverted repeat transposable elements (MITEs) first discovered in maize were found to be widespread in various higher organisms [4-9]. MITEs have some common characteristics: short, terminal inverted repeats (TIR), target site duplication (TSD), high AT content, potential to form stable secondary structure, high number of copies in a genome [3,9-11]. Importantly, recent genome-wide analyses revealed that MITEs insert preferentially into or near genes [5,12,13] and that several families of miRNAs in humans, Arabidopsis, rice and Solanaceae were derived from MITEs [14-16]. These results suggest that MITEs play important roles not only in genome evolution but also in transcriptional regulation of genes.

MITEs were originally discovered in plants and have two major superfamilies. They are *Tourist*-like and *Stowaway*-like on the basis of their similarity to two elements previously identified in maize and sorghum [3,4,10]. It is now clear that these two MITEs originated by internal deletion of the corresponding autonomous elements [4] and could transpose by other autonomous DNA elements [17,18]. Virtually, all MITEs have been identified through computer-assisted database searches [11]. As more and more genome sequences become available, therefore, several programs have been developed for MITE identification [8,19,20]. Strikingly, a recently developed program, known as MUST, has been verified to be very powerful and efficient for identification of MITEs [20]. This facilitates discovery of more novel MITEs.

Silkworm, *Bombyx mori*, is a model insect for the order Lepidoptera and has importantly economic value for silk production and bioreactor. The draft genome sequences of silkworm were released by Mita et al. [21] and Xia et al. [22], respectively. Recently, a new assembly has been completed [23]. Analyses of the silkworm genome sequence suggested that ~40% of the genome is composed of the known TEs [24]. This number is only smaller than the TE proportion (47%) in *Aedes aegypti *genome that has the largest proportion of TEs in the insect genomes sequenced to date [25]. DNA transposons (*Tc1-mariner*, *Helitron*, *Harbinger*, *hAT*, *P *and *Piggybac*) are only ~3% of the genome while most of the silkworm TEs are retrotransposons [24]. Although two MITEs, *Hoshidandy *and *Organdy*, were previously discovered in silkworm [26] (also see GenBank accession no. AB455941), the genome-wide information about the silkworm MITEs remains unclear.

In this study, we scanned the new assembly of the silkworm genome sequence to identify MITEs by using a recently developed program, MUST and a strict filtering approach [20]. The results indicated that the silkworm genome harbors 17 MITE families. Estimates of insertion date and diversity for each MITE family showed that the silkworm MITE families might have experienced burst expansions at different time points of evolution and exhibited various patterns of diversification. That MITEs preferentially insert into or near genes has been also confirmed in silkworm. In addition, we found evidence that a sRNA derived from a MITE might silence its host and neighbor genes.

## Results

### Mining and characterization of MITEs

MUST [20], a program designed to detect MITE elements, was first used to search the silkworm genome sequence. The program identifies candidates based on common features of MITEs (short, TSDs and TIRs structure) and sequence alignment. With MUST, we mined 143333 MITE candidates in the silkworm genome, which were grouped into 1350 families. Then, we filtered out pseudo-MITEs from predicted ones by a strict approach: Those containing undetermined fragments (designated as Ns in scaffolds) as well as those solely composed of simple repeats and nested in repeats were considered as pseudo-MITEs (additional file 1). By this way, the number of the MITE families reduced to 17 (Table 1). These 17 families include 3337 intact MITEs and were designated as BmMITE-1 to BmMITE-17, respectively. TSD lengths of all MITEs range from 2 to 9 bp, TIR lengths from 8 to 59 bp, and full lengths of complete MITEs from 210 to 567 bp. Three families are flanked by TA, three families flanked by TWA, four families flanked by NNNNNNNN (the N represents A, T, C or G) and seven families flanked by WW, TDA, ATT, ATAT, ATATAT, TTCATTT, TTACTGTAT (the W represents A or T; D represents A, T or G), respectively. Based on the nucleotide composition of TSD, 3 MITE families (BmMITE-4,5,6) belong to *Tourist*-like family [4], 3 families (BmMITE-2,3,8) belong to *Stowaway*-like family [10]; based on the nucleotide composition of TSD and TIR, 4 families (BmMITE-13,14,15,16) belong to *Pegasus*-like family [27], and the remaining 7 families (BmMITE-1, 7, 9, 10, 11, 12, 17) were novel.

**Table 1:** Families and characteristics of silkworm MITEs

Family	**TSD**^**1**^	TIR	Size (bp)	AT content (%)	**No.FC**^**2**^	**No.FLC**^**3 **^**(%)**	**-ΔG**^**4**^	Known family
BmMITE-1	WW	TCGATGGCTCCAATGAACACTAC	234	68	216	44(17)	39	Novel
BmMITE-2	TA/AT	TGAGTCGACTATTATCAAAG	278	67	419	2371(85)	66	*Hoshidandy*^5^
BmMITE-3	TA	GATATGTGTCGTTCG	306	54	12	34(74)	47	*Stowaway*-like
BmMITE-4	TWA	GGGTCAATTCCCACTGAAAGAGCAGCGGC	567	44	9	7(44)	125	*Tourist*-like
BmMITE-5	TWA	AGCCTTGTTCGCACTAAGCGAGTATTTTA GTCGAGTACCGAGTAATTTAGTGGCTAAA	213	61	53	81(60)	74	*Tourist*-like
BmMITE-6	TWA	GGGCCTGTGCACACCACGTTTTTTAA	270	52	11	8(42)	81	*Tourist*-like
BmMITE-7	TDA	TGCTGGAACCACACTGCG	548	55	7	13(65)	93	*Organdy*^6^
BmMITE-8	TA	TATATCGACGCTTGAAAGGCAAAC	266	67	1364	147(10)	47	*Stowaway*-like
BmMITE-9	ATT	GGTAGTTTTCCAATTACAG	418	63	75	88(54)	52	Novel
BmMITE-10	ATAT	CGTCGCTGTCAAACCAAAATCTGCTATGTGCAA	258	70	142	159(53)	38	Novel
BmMITE-11	ATATAT	GTGGGATT	238	67	1	15(94)	8	Novel
BmMITE-12	TTCATTT	TTACTTTGCA	210	73	20	121(86)	14	Novel
BmMITE-13	NNNNNNNN	CAAGGGCGGATCCAG	263	59	69	171(71)	32	*Pegasus*-like
BmMITE-14	NNNNNNNN	CAGTGGCGGATTA	431	59	12	22(65)	38	*Pegasus*-like
BmMITE-15	NNNNNNNN	CAGTGGCGTACCTA	300	65	0	9(100)	60	*Pegasus*-like
BmMITE-16	NNNNNNNN	CAGTGGCGGATTT	265	55	18	25(58)	43	*Pegasus*-like
BmMITE-17	TTACTGTAT	GCGCGCGAGTTCATGT	494	59	20	22(52)	53	Novel

^1 ^N = A/T/C/G; W = A/T; D = A/T/G. ^2 ^No. FC means the number of fragmentary copies. ^3^No.FLC indicates the number of full length copies, the number of the bracket is the ratios of full length copies in total of each MITE family. ^4^-ΔG means the average negative ΔG(kcal/mol) value of each MITE family. ^5^*Hoshidandy *was referred to the accession No. AB455941 in NCBI. ^6^*Organdy *was first discovered in silkworm by Komoto *et al*. [26]. MITE families were classified based on TIR, TSD and internal sequences.

Three families flanked respectively by TA and by TWA have no any similarities in TIR and intra-sequence; they may have independent origins but may be transposed by the same transposase. Four families flanked by NNNNNNNN have no any similarities in intra-sequence but have high similarities in TIR regions; they may derive from a common ancestor and may be transposed by the same transposase [3]. Seven families with different TSD, TIR and intra-sequences may have different origins and may be transposed by different transposases.

Next, a homology search was used to estimate number of copies for each MITE family in the silkworm genome. That is, a BLASTN (E < e^-5^) search [10] was used against the silkworm genome sequence. With this approach, we identified 5785 MITEs in total, which constitute ~1.86 Mb (0.4%) of the silkworm genome. Then, each MITE was classified as intact (full length) or fragmentary: individual with both complete TIRs was regarded as intact and otherwise as fragmentary. As a result, 3337 MITEs are intact whereas 2448 MITEs are fragmentary. As mentioned above, 5785 MITEs belong to 17 families. However, family size varies greatly among MITEs, which ranges from 9 to 2790 (Table 1). The information about insertion sites of the MITEs into scaffold was shown in Additional file 2. The largest family is BmMITE-2 with 2790 copies. The copies of both BmMITE-2 and BmMITE-8 occupy approximately 74% of all MITEs. The ratio of full length to all copies of each family ranges from 10% to 100%. Four MITE families (BmMITE-1, BmMITE-4, BmMITE-6 and BmMITE-8) have the ratios less than 50% and all others more than 50%. Especially, BmMITT-2, BmMITE-11, BmMITE-12, and BmMITE-15 have 85%, 94%, 86% and 100% of high ratios, respectively, suggesting that they may be very young and active recently. It should be noted that BmMITE-2 has the largest number (2371) of full length MITEs whereas BmMITE-8 has the largest number (1364) of fragmentary MITEs among the families, suggesting that the former might experience a recent burst expansion whereas the latter might undergo an old burst expansion.

The average AT content for each MITE family ranges from 44% to 73%. Only BmMITE-4 has less than 50% AT content and all others more than 50%, suggesting that the silkworm MITEs are AT rich. The average AT content of the silkworm genome is approximately 62% [23]. Eight families BmMITE-1, BmMITE-2, BmMITE-8, BmMITE-9, BmMITE-10, BmMITE-11, BmMITE-12 and BmMITE-15 have AT content more than the average of the silkworm genome.

**Figure 1:**
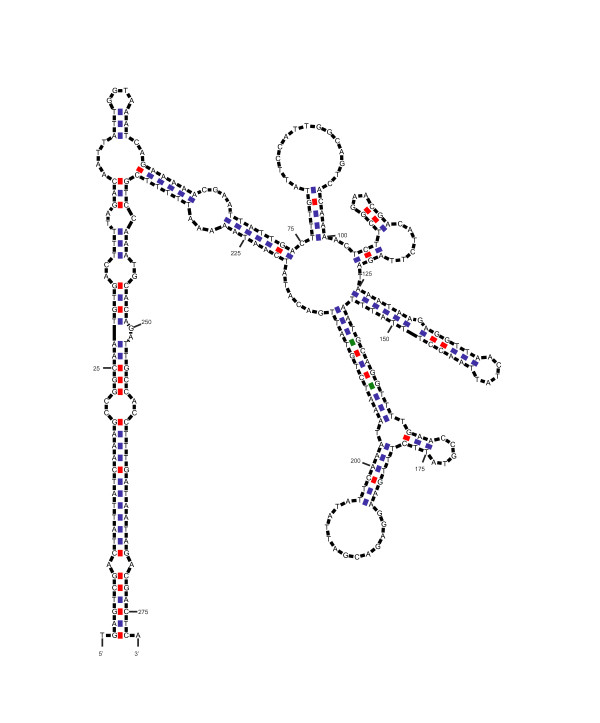
**The secondary structure of BmMITE-2 predicted by the program UNADOLD**.

The secondary structure and negative ΔG value of Gibbs energy were predicted by UNAFOLD [28]. The results suggested that all the silkworm MITEs have the predicted secondary structures (Figure 1 and additional file 3). Furthermore, almost all MITE families have high negative ΔG values except for BmMITE-11 (8 kcal/mol) and BmMITE-12 (14 kcal/mol), indicating that most silkworm MITEs have potential to form stable secondary structures. These results imply that they may play important roles in transcriptional regulation of genes.

The identified silkworm MITE families were annotated based on BLAST search. Using a representative member of each MITE family as query, we searched ISfinder, RepBase and NCBI nr databases, respectively. These three databases almost include all known MITEs and transposable elements. We found that BmMITE-2 perfectly matched to one silkworm MITE named *Hoshidandy *(No. AB455941) in NCBI nr database and BmMITE-7 perfectly matched to *Organdy *identified previously in silkworm [26] (Table 1). Another 15 MITE families have no match to any known MITEs or transposable elements. This suggested that the program MUST used in this study is feasible and efficient for identification of MITEs because 2 known families of the silkworm MITEs were all recovered and 15 novel families were discovered by MUST [20].

### Verification of the predicted MITEs

Four predicted insertion sites of BmMITE-2 were selected for verification by PCR in 14 silkworm accessions representing four main geographic strains (Chinese, Japanese, European, and Tropical). Primers were designed based on flanking regions of each insertion site (additional file 4). The PCR results for the four insertion sites were summarized in additional file 5. Furthermore, sequencing of the corresponding PCR products confirmed the presence or absence of BmMITE-2 (additional file 6). The polymorphic distribution of indel for all four tested insertion sites suggested that they are not fixed residents of the silkworm genome and validated the prediction by the program MUST [20].

**Figure 2:**
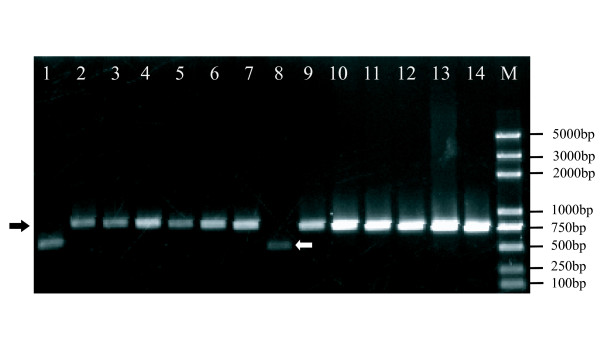
**PCR verification in 14 silkworm strains for BmMITE-2**. Lanes from 1 to M are: Wu-B, Wu-D, DongDe-201, Wu-E, Lu-10, DaXianTuZhong, FuRongHuiLuan, PeiXianZhong, Ri-9, HeiZi, ChunSi, QiongShanHaiNan, Ri-110, ShangSanHuBan, DNA marker, respectively. The black arrow points to the BmMITE-2 occupied at this genomic location. The white arrow represents the BmMITE-2 lack at this genomic location.

Figure 2 shows the results of amplification for insertion site 4: The BmMITE-2 is present at the lines Wu-D, DongDe-201, Wu-E, Lu-10, DaXianTuZhong, FuRongHuiLuan, Ri-9, HeiZi, ChunSi, QiongShanHaiNan, Ri-110, ShangSanHuBan and absent at strains Wu-B, PeiXianZhong. The presence/absence polymorphisms at all four insertion sites of BmMITE-2 across the 14 tested silkworm lines suggested that BmMITE-2 may be recently active.

### Estimates of insertion date and diversity

**Figure 3:**
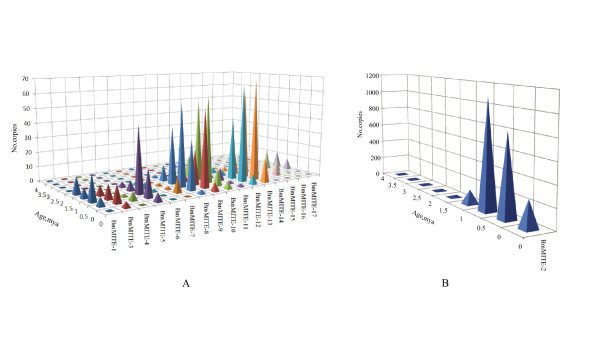
**Insertion dates of each MITE family in the silkworm genome**. The level of nucleotide substitutions (k) between each MITE element and the family consensus sequence was estimated using Kimura 2-parameter distance. Then, the insertion time was calculated by the method in [13].

We estimated age of each full length MITE by the method used for maize MITE families [13]. Briefly, we first determined divergence between each MITE and the family consensus sequence, and then estimated insertion time based on the divergence. Figure 3 showed that insertion date varies greatly among members of each family as well as among families, which ranges from 0 to 4 million years ago (mya). Strikingly, BmMITE-2 might be dramatically expanded during a period from 0 mya to 1 mya (Figure 3B) and accumulated up to 2173 copies during this short period while the rest 16 families might experience major expansion events within 2 mya (Figure 3).

**Figure 4:**
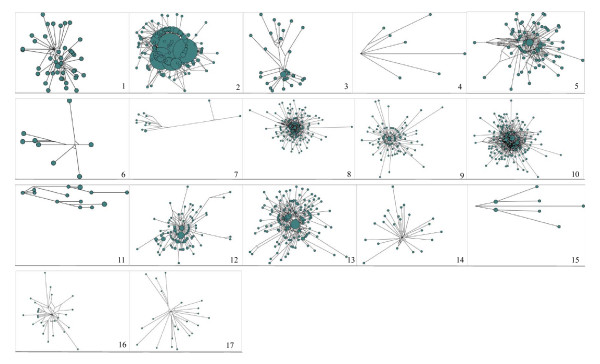
**Median-joining networks of 17 silkworm MITE families**. 1 to 17 represent BmMITE-1 to BmMITE-17 family, respectively. The circle, circle area and branch length represent MITE sequence, proportional to the number of identical copies and proportional to the number of nucleotide changes, respectively.

To examine intrafamily diversity pattern for each family, we performed network analyses on the basis of the alignment of full length sequences. Topology of a network reflects corresponding demographic events that each MITE family experienced [13,29]. For instance, a network topology showing numerous nodes distributed around its centre and separated by long branches implies that this family might experience an old population expansion. In contrast, a network topology characterized by a central node surrounded by many, almost identical, and short branches, indicates a very recent expansion from an ancestral element [13]. 8 (BmMITE-4, BmMITE-6, BmMITE-7, BmMITE-11, BmMITE-14, BmMITE-15, BmMITE-16 and BmMITE-17) of 17 MITE families presented the topologies that these MITE families might experience old population expansions. The topologies of the rest 9 families indicated that these families might undergo recent expansions (Figure 4). These observations are basically consistent with the insertion time estimates.

Pattern of regional variation for each family provides details of divergence between copies. We performed diversity (pi) sliding window analysis for the alignment of full length sequence of each MITE family. Window size is 20 nucleotides and step size is 20 nucleotides [13]. The regions with diversity equal or higher than average sequence diversity (pi) +2SD was defined as variable whereas those with diversity less than average sequence diversity (pi)-2SD were considered as conserved regions (additional file 7). It was found that regional variation pattern varies greatly among these 17 silkworm MITE families. Almost all families have highly conserved TIRs, however, 6 families (BmMITE-4, BmMITE-5, BmMITE-9, BmMITE-12, BmMITE-14, and BmMITE-15) have only one conserved TIR. Because TIRs must be first recognized by corresponding transposase for transposition, it is not surprising to see more conserved in TIR than intra-sequence.

### Distributions of MITEs on chromosomes and Estimation of MITE richness in genes

We recorded information about position of each MITE on a chromosome to look at distribution of MITEs in the genome. The results suggested that all MITE families are widely distributed on all 28 silkworm chromosomes. Then, we examined whether MITEs were randomly distributed among 28 silkworm chromosomes using χ^2 ^test. The null hypothesis was rejected (*P *< 0.01), suggesting that the distribution of MITEs is nonrandom in the silkworm genome (additional file 8).

Next, we examined whether insertion site of each MITE preferentially is in or close to genes. If a MITE inserts into within the 5 kb flanking regions of a genes, this MITE is regarded as close to a gene [13,30-33]. The results indicated that a larger number of MITEs inserted into gene regions (exon, intron) and flanking regions of genes. 3794 (66%) of the 5785 predicted MITEs inserted into gene regions (Table 2). Of 3794 MITEs inserted into gene regions, 962 (25%) were located in 5` flanking regions of the closest genes, 60 (2%) in exons, 1427 (38%) in introns and 1343 (35%) in 3`flanking regions of the closest genes, respectively. It appears that the silkworm MITEs preferentially inserted into introns and 3'-flanking regions rather than 5'-flanking regions and exons. To determine whether insertions of MITEs into gene regions are due to chance, we performed a computer simulation as a negative control (see Methods for details). It was found that the silkworm MITEs have significantly higher insertion frequencies into gene regions than those in control (66% vs 39%: χ^2 ^test, *P *= 0.0, Table 2), implying that the silkworm MITEs preferentially do insert into gene regions. When a MITE insertion into within respective 500, 500-3000, and 3000-5000 bp flanking regions of a gene was assumed to be close to a gene, the patterns of MITE insertion in the genome are also similar to that a MITE within 5 kb flanking regions of a genes (additional file 9).

**Table 2:** Characteristics of insertion sites of the silkworm MITEs.

Insert into						
		
Family	No. analyzed copies	5'-flank < 5 kb^1^	Exon	Intron	3'-flank < 5 kb^1^	Total
BmMITE-1	260	38(15)	3(1)	53(20)	61(23)	155(60)
BmMITE-2	2790	482(17)	15(1)	651(23)	643(23)	1791(64)
BmMITE-3	46	12(26)	0(0)	16(35)	15(33)	43(93)
BmMITE-4	16	2(13)	2(13)	3(19)	8(50)	15(94)
BmMITE-5	134	22(16)	8(6)	26(19)	25(19)	81(60)
BmMITE-6	19	3(16)	4(21)	6(32)	3(16)	16(84)
BmMITE-7	20	4(20)	0(0)	8(40)	4(20)	16(80)
BmMITE-8	1511	225(15)	23(2)	431(29)	365(24)	1044(69)
BmMITE-9	163	40(25)	0(0)	26(16)	58(36)	124(76)
BmMITE-10	301	32(11)	0(0)	96(32)	57(19)	185(61)
BmMITE-11	16	1(6)	0(0)	2(13)	3(19)	6(38)
BmMITE-12	141	18(13)	0(0)	16(11)	21(15)	55(39)
BmMITE-13	240	54(23)	2(1)	42(18)	49(20)	147(61)
BmMITE-14	34	7(21)	0(0)	16(47)	7(21)	30(88)
BmMITE-15	9	5(56)	1(11)	1(11)	2(22)	11(100)
BmMITE-16	43	14(33)	1(2)	9(21)	10(23)	34(79)
BmMITE-17	42	3(7)	1(2)	25(60)	12(29)	41(98)
Total	5785	962(17)	60(1)	1427(25)	1343(23)	3794(66)
Control	5000	712(14)	152(3)	453(9)	653(13)	1970(39)

^1^The distance of the insertion site from predicted gene. Number in each bracket is the percentage of insertion sites. Control indicates a negative experiment similar to that in [33]

To investigate whether silkworm MITEs preferentially insert in a special gene family, we annotated all the closest genes using WEGO, an online gene ontology website, and compared the closest genes with all silkworm genes (additional file 10). The results indicated that functionalities of the closest genes were randomly selected.

### A MITE containing small RNA and expressions of its closest genes

**Figure 5:**
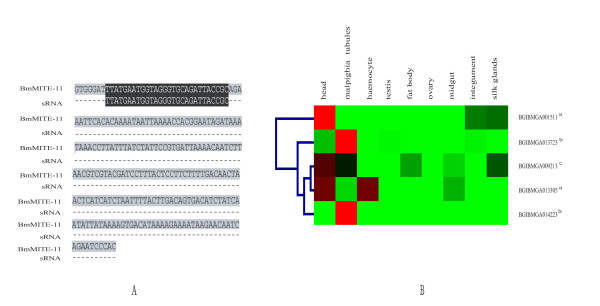
**BmMITE-11 containing the small RNA and expression profiles of its the closest genes**. (A) The alignment of BmMITE-11 and small RNA (RNA-36850, Genbank accession no. AB423040). (B) Expression profiles of the closest genes to which BmMITE-11 is located. Red indicates upregulated genes, green down-regulated genes, black no expression change. ^a, b, c ^represent the closest genes with BmMITE-11 inserted into their introns, 3` flanking and 5`flanking regions, respectively.

We scanned nr database at NCBI using a representative member of each silkworm MITE family as query and found a small RNA known as RNA-36850 (No. AB423040) perfectly matched to BmMITE-11 (Figure 5A). This small RNA is 28 bp long and was first discovered in silkworm ovary by Kawaoka et al. [30]. Previous studies showed that this small RNA (RNA-36850) belonged to a new class of germline-restricted small RNAs (26-33 bp) called piRNAs, which is thought to defend the host genome against transposons [31].

To determine whether BmMITE-11 can regulate expressions of the closest genes through the small RNA, we examined expression profiles of the closest genes using the available microarray data [32]. First, we found the six closest genes of BmMITE-11: BGIBMGA009213, BGIBMGA001511, BGIBMGA013723, BGIBMGA013305, and BGIBMGA014223 in SilkDB http://silkworm.swu.edu.cn/silkdb/. With information of these six genes, corresponding expression profiles were extracted from the microarray data. Because BGIBMGA002049 has no information in microarray data, so it was not included in the study. Figure 5B showed that the five closest genes have different expression patterns. However, they have a common feature that all the five genes were not expressed in the ovary and testis. There are 10393 active genes in the silkworm microarray data. 8431 of 10393 genes were expressed in ovary or testis [32]. Thus, the probability of 5 random selected genes that were not expressed in ovary or testis is 0.000241 (*P *= (1- 8431/10393)^5 ^= 0.000241). Chance cannot explain the observed expression pattern. Therefore, BmMITE-11 might self-silence germline-specifically by small RNA. As a result, BmMITE-11 might silence these five closest genes in silkworm ovary through small RNA pathway.

## Discussion

### Discovery and characterization of the silkworm MITEs

In this study, we carried out a systematic and genome-wide analysis to search for MITEs in silkworm using a novel computational approach, MUST [20]. First, 143333 MITEs were predicted and were grouped into 1350 families. Having further examined these predicted MITEs, however, we found that a large number of MITEs were pseudo-MITEs. After removing pseudo-MITEs by a strict filtering process, we identified only 17 silkworm MITE families. Of 17 families, *Hoshidandy *and *Organdy *have been reported previously [26]. However, 7 of 17 families are novel according to the classification of nucleotide composition of TSD. This suggests that the procedure for MITE identification used in this study is reliable.

Previous studies identified various MITEs in other insects. For example, eight MITE families were found in mosquito [8]. Our results indicated that silkworm harbors 17 MITE families and 7 of which are completely novel. Furthermore, the silkworm MITEs show huge diversification in both TSD composition and full length sequence (Table 1). However, all families have MITE-specific features: high number of copies, high AT content and potential to form a stable secondary structure. This also suggested validity of MUST in predicting the silkworm MITE. In addition, the verification of predicted MITEs by PCR and sequencing of the PCR products showed existence of MITEs.

It should be pointed out that a large number of candidate MITEs were predicted by MUST at first. However, it was found that most of these candidates were false positive after filtering out by a strict process. Probably, a high rate of false positive in MITE identification by MUST is due to incompleteness of the silkworm genome sequence, that is, there are many gaps to be sequenced in the genome. Again, that silkworm has a large proportion of repetitive sequences may affect correct identification of MITEs. In addition, a high sensitivity of MUST in identifying MITEs may be a reason. It can be known from considerations above that 17 MITE families contained in silkworm should be a conserved number. More silkworm MITEs are to be identified in future.

### Expansion and diversity pattern

The ages of 17 MITE families vary greatly (Figure 3), ranging from 0 to 4 mya ago. Strikingly, BmMITE-2 might be dramatically amplified during a period from 0 mya to 1 mya (Figure 3B). A similar result has been reported that *mPing *underwent a dramatic amplification during rice domestication [33]. It is interesting to determine whether silkworm MITEs experienced recent expansions is due to bottleneck of silkworm domestication in future.

It should also be noted that the method used to estimate time of insertion of MITE copies in this study has an implicit assumption that all full sequences evolve neutrally and rate-equally [13]. However, this may not be true: Not all sequences are under neutral evolution; there may be differential rate of substitution among nucleotides as some regions may be important for transposition and there may be different rate of change in different genomic regions. All these will affect the accuracy of estimate time of MITE insertion. To solve these problems, a sophisticated estimation method needs to be established in future. Nevertheless, the results of MITE insertion time presented in this study should provide some useful information about the evolutionary dynamics of the silkworm MITEs.

Our diversity analyses suggested that members of each MITE family have a high level of similarity and similar sequence lengths. This shows that they can be considered as a population that experienced several successive steps of amplifications from a handful of master copies [13]. Right after amplification, copies are almost identical but will diverge in sequence and length over time due to random mutation. Therefore, a large number of identical or very similar copies within a MITE family imply a recent burst. Our network analyses revealed that 9 MITE families (BmMITE-1-3, BmMITE-5, BmMITE-8-10, BmMITE-12, and BmMITE-13) might experience several successive recent expansions (Figure 4). If these recent expansions were associated with the demographic events that silkworm experienced such as domestication bottleneck, then network topologies of above 9 MITE families can be explained by the genome-shock theory proposed by McClintock [34]. Thus, it is interesting to investigate dynamics of these silkworm MITE families during domestication in future.

### Distribution of MITEs in the genome and their contribution to gene regulation

Our observations suggested that the silkworm MITEs are widely distributed in the genome. However, they are not randomly distributed on chromosomes (additional file 8). Since the silkworm MITEs preferentially insert into gene regions like MITEs in other higher organisms, a nonrandom distribution of the silkworm MITEs may be due to different gene densities on chromosomes. However, we did not find the significant correlation between the densities of genes and MITEs among the silkworm chromosomes (*R*^2 ^= 0.055, *P *> 0.05, df = 26). Thus, the causes of nonrandom distribution of the silkworm MITEs on chromosomes are to be explored.

We observed that the silkworm MITEs in introns, 3'flank and 5'flank are in total much more than in exons (Table 2). It should be noted that the silkworm MITEs inserted into introns and 3' flanking regions significantly more than 5' flanking regions (χ^2 ^test: *P *< 0.01, respectively). These observations could be explained by two reasons: MITE insertions in exons were rapidly purged out from a population because they are deleterious [12]; because many MITEs have been found to contain poly (A) signal [10], these MITEs are likely to be maintained in 3' flanking regions to act in regulation. Since introns have been suggested to harbor regulatory elements [35], much more silkworm MITE insertions into introns imply that the MITEs may play important roles in gene expression by changing regulatory motifs. In addition, that MITEs preferentially insert into gene regions provides a material basis for establishment of a TE-derived genetic regulatory network [36] and this is also an interesting topic to be studied in future.

A recent study tried to relate MITEs to biogenesis of their siRNAs in Solanaceae [7]. However, evidence for functional implications of MITEs in gene regulation through small RNA pathways is still lacking. Most importantly, we found a BmMITE-11 from which a silkworm ovarian small RNA (RNA-36850: No. AB423040) derived (Figure 5A). The expression data further suggested that the closest genes of BmMITE-11 were germline-specifically silenced (Figure 5B). Because this small RNA (RNA-36850) belongs to a new class of germline-restricted small RNAs (26-33 bp) called piRNAs [30,31], our result may be the first evidence for effects of a MITE on its neighbor genes in transcriptional regulation through the sRNA pathway. The validation of this mechanism is underway. Given the high copy number of MITEs, many small RNAs and miRNAs derived from MITEs [14-16] and their preferential insertion into gene regions, it will be important to systematically account for the different mechanisms of MITEs and their potential functional roles in transcriptional regulation of genes.

## Conclusions

Although MITEs in various higher organisms including mosquito and *Drosophila *as well as beetle have been extensively investigated, little is known about the genome-wide information of MITEs in the silkworm genome. We identified the 17 silkworm MITE families by using a recently developed algorism to scan the genome sequence. Silkworm has 17 MITE families, and furthermore, 7 of 17 families are completely novel based on the nucleotide composition of TSD. These results added new knowledge for understanding the evolution of MITEs. Importantly, we not only corroborated the preference of MITEs inserted into or near genes seen in the other genomes but also found that BmMITE-11 might silence its closest genes through sRNA pathway. These results emphasize the exceptional role of MITEs in the transcriptional regulation of genes and have general implications to understand the interaction between MITEs and their host genome.

## Methods

### Mining and characterization of MITEs

The new assembly of the silkworm genome sequence was downloaded from SilkDB http://silkworm.swu.edu.cn/silkdb[23]. First, a structure-based approach implemented in the MUST program was used to search the silkworm genome sequence for candidate MITEs that have characteristics [20]: TIR with 8-50 bp of length; TSD with 2-30 bp of length; 100-600 bp of the sequence length. The parameters were selected based on the common features of known MITEs. Candidate MITEs were grouped by all-blast-all. The MITEs for which any pair has an identity >0.8 were defined as a family. Families that have <3 members were excluded to reduce false positive rate for identification of MITEs.

Next, a homology BLAST search was used to scan the silkworm genome sequence with a representative member for estimating copy number of each MITE family. At this step, a MITE family was defined by a sequence similarity with E < e^-5 ^for BLASTN. This standard has been recently used by Kuang et al. [7]. The BLAST results were filtered out using a Perl script according to criteria: a minimum nucleotide identity rate >90% and query coverage >80%. Those including both complete TIRs were defined as intact MITEs and others as fragmentary MITEs.

UNAFOLD http://www.bioinfo.rpi.edu/applications/hybrid/twostate-fold.php was used to predict secondary structures of MITEs [28]. Then, all MITE families were used against the ISfinder database http://www-is.biotoul.f/is.html[37], RepBase database (version 14.11) [38] and NCBI nr database respectively to find known families.

### Verification of predicted MITEs

Fourteen silkworm accessions (Wu-B, Wu-D, DongDe-201, Wu-E, Lu-10, DaXianTuZhong, FuRongHuiLuan, PeiXianZhong, Ri-9, HeiZi, ChunSi, QiongShanHaiNan, Ri-110, ShangSanHuBan) that represent four main geographic strains (Chinese, Japanese, European, and Tropical) were used in insertion validation of a predicted MITE at four sites. A MITE used in insertion validation was randomly selected and the primers were designed based on flanking regions of each of insertion sites (additional file 4).

### Estimates of insertion time and diversity of MITE families

To estimate the age of MITEs, DNA sequences of each MITE family were aligned using Clustal W [39] and the family consensus sequences were constructed using the program DAMBE [40], then the level of nucleotide substitutions (k) between each MITE element and the family consensus sequence was estimated using Kimura 2-parameter distance [41]. The each MITE age was estimated using the formula T = k/2r, assumed r = 1.56 × 10^-8^, it's the fruitfly neutral rate of substitutions per year and has been used in silkworm [22].

All full length sequences of each MITE family were aligned using Clustal W [39]. Then, using the alignment of all full length sequences of each family and the program Network4.5 http://www.fluxus-engineering.com/sharenet.htm[42], we constructed median-joining (MJ) networks to estimate BmMITE intrafamily diversity. This method has been used in maize [13]. Additionally, a sliding window analysis with a window size 20 bp and a step size 20 bp was used to look at pattern of regional nucleotide diversity (pi) for each family and completed by DnaSP (version5.10) [43].

### Distributions of MITEs on chromosomes and Estimation of MITE richness in genes

All identified members of each MITE family were mapped to chromosomes by SilkMap http://silkworm.swu.edu.cn/silksoft/silkmap.html and copy numbers of each family on chromosomes were counted.

Two files for positions of predicted genes in scaffolds and for lengths of scaffolds were downloaded from SilkDB http://silkworm.swu.edu.cn/silkdb. Then, Perl script was written to scan the files for extracting the information of MITEs close to or in predicted genes. To determine whether insertions of MITEs close to gene regions are due to chance, a computer simulation, which is similar to that used by Naito et al. [33], was performed. Briefly, the fragments of up to 10 kb were randomly sampled from the silkworm genome sequence, the middle of each 10 kb sequence was presumed as the insertion site and the information about the insertion site close to or in predicted genes (i.e., in intron and exon) was recorded accordingly. The genes that have less than 5 kb distances to MITEs or contain MITEs were defined as the closest genes.

The closest genes were annotated by WEGO http://silkworm.swu.edu.cn/cgi-bin/wego/index.pl, and gene expression profiles were examined by using the available microarray data as described in Xia et al. [32].

## Authors' contributions

ZZ and MJH designed the study. MJH did the data analyses and drafted the manuscript. YHS and YHG performed the experiments. LYC revised the manuscript. ZHX supervised the study. ZZ drafted and revised the manuscript. All authors read and approved the final manuscript.

## Supplementary Material

Additional file 1 **The examples for Pseudo-MITEs**: (A) undetermined fragments (designated as Ns in scaffolds), (B) solely composed of simple repeats, and (C) nested in repeats, arrow represents TSD and underline represents TIR. Click here for file

Additional file 2 **The information about insertion sites of the MITEs into scaffolds**. Click here for file

Additional file 3 **The secondary structures of silkworm MITEs predicted by the program of UNADOLD: 1: BmMITE-1,2-16: BmMITE-3-17, respectively**. Click here for file

Additional file 4 **Primers for PCR verification of BmMITE-2**; Click here for file

Additional file 5 **The results for PCR verification of predicted BmMITE-2**. Click here for file

Additional file 6 **Sequencing result of PCR products for the presence or absence of BmMITE-2 at insertion site 3**. "DXTZ", "SSHB" represent DaXianTuZhong and ShangSanHuBan strains, respectively. The box represents the sequence of BmMITE-2. Click here for file

Additional file 7 **Nucleotide variation (π) along MITE sequence for each family**. Nucleotide diversity π and its standard deviation were calculated by the program of DnaSP version 5.10. Then, the same program was used to define conserved and variable of the MITE sequences by sliding window analysis with both window size and step size 20 nucleotides. Windows with diversity equal or higher than average sequence diversity (π) + 2SD were defined as variable. Those with diversity less than average sequence diversity (π) - 2SD were considered as conserved. Click here for file

Additional file 8 **Distribution of the silkworm MITEs on the 28 chromosomes**. The observed distribution is significantly different from the expected one based on the total length of 28 chromosomes (Chi-square = 297, df = 27, P < 0.01). *The chromosomes that show the observed copies more than expected. Click here for file

Additional file 9 **MITE distances to the nearest genes**. Click here for file

Additional file 10 **The annotation of the closest genes using WEGO**. Click here for file
