# Supplementary material for: Burst expansion, distribution and diversification of MITEs in the silkworm genome
Source: BMC Genomics. 2010 Sep 27;11:520. doi: 10.1186/1471-2164-11-520 (PMC2997013; doi:10.1186/1471-2164-11-520)
Supplement: Additional file 1 — The examples for Pseudo-MITEs: (A) undetermined fragments (designated as Ns in scaffolds), (B) solely composed of simple repeats, and (C) nested in repeats, arrow represents TSD and underline represents TIR. [file 1471-2164-11-520-S1.PDF]

```
TGCACGTCGACCTCTTTGTCGAGTTCGACGA
GTAACGGTTACCGGGGTCCCTAAGCCTNNN
NNNNNNNNNNNNNNNNNNNNNNNNNNNNNN
NNNNNNNNNNNNNNNNNNNNNNNNNNNNNNN
NNNNNNNNNNNNNNNNNNNNNNNNNNNNNNN
NNNNGCAACGATAAGGCGATTATCATGACG
CATAGCTTATCGAAGTATCGTTCCGACGCTG
ACTTCATGTATTTCCGAATGATTTCGAGGCC
CAGGTCGTCGTGTA
```

A

```
CTATACTATACTATACTATACTATACTATACTATACTATACTAT
ACTATACTATACTATACTATACTATACTATACTATACTATACTA
CACTATACTATACTATACTACACTATACTATACTATACTATA
CTATACTATACTATACTATACTATAC
```

B

```
                 *        20         *        40         *        60         *
nscaf2902  : TGGGATGTAATGGGGCAATTAACACCACACTGCCTCAAATGCCAACCCTACCTGCACGTGGTGCATCCTGCT :  72
nscaf2964  : TGGGATGTAATGGGGCAATTAACACCACACTGCCTCAAATGCCAACCCTACCTGCACGTGGTGCATCCTGCT :  72
preMITE    : ------------------------------------------------------------------------ :   -
nscaf2529  : -GGGATGTAATGGGGCAATTAACACCACACTGCCTCAAATGCCAACCCTACCTGCACGTGGTGCATCCTGCT :  71
scaffold35 : TGGGATGTAATGGGGCAATTAACACCACACTGCCTCAAATGCCAACCCTACCTGCACGTGGTGCATCCTGCT :  72
              gggatgtaatggggcaattaacaccacactgcctcaaatgccaaccctacctgcacgtggtgcatcctgct

                 80         *       100         *       120         *       140
nscaf2902  : GATTGACGAACAGGGCTCTGGCGACCGATAGATGGCGGAATAACCATCAACAAAAACCGCTCGGTGCGGAAA : 144
nscaf2964  : GATTGACGAACAGGGCTCTGGCGACCGATAGATGGCGGAATAACCATCAACAAAAACCGCTCGGTGCGGAAA : 144
preMITE    : ------------------------------------------------------------------------ :   -
nscaf2529  : GATTGACGAACAGGGCTCTGGCGACCGATAGATGGCGGAATAACCATCAACAAAAACCGCTCGGTGCGGAAA : 143
scaffold35 : GATTGACGAACAGGGCTCTGGCGACCGATAGATGGCGGAATAACCATCAACAAAAACCGCTCGGTGCGGAAA : 144
             gattgacgaacagggctctggcgaccgatagatggcggaataaccatcaacaaaaaccgctcggtgcggaaa

                 *       160         *       180         *       200         *
nscaf2902  : TCTCCTGGCCGGTAACGGGCAGCAGCATTACCGGTGCAAGGCACCGAGTACTGCGATCGCCAACCCGCCTGC : 216
nscaf2964  : TCTCCTGGCCGGTAACGGGCAGCAGCATTACCGGTGCAAGGCACCGAGTACTGCGATCGCCAACCCGCCTGC : 216
preMITE    : -------------------------CATTACCGGTGCAAGGCACCGAGTACTGCGATCGCCAACCCGCCTGC :  47
nscaf2529  : TCTCCTGGCCGGTAACGGGCAGCAGCATTACCGGTGCAAGGCACCGAGTACTGCGATCGCCAACCCGCCTGC : 215
scaffold35 : TCTCCTGGCCGGTAACGGGCAGCAGCATTACCGGTGCAAGGCACCGAGTACTGCGATCGCCAACCCGCCTGC : 216
             tctcctggccggtaacgggcagcagCATTACCGGTGCAAGGCACCGAGTACTGCGATCGCCAACCCGCCTGC

               220         *       240         *       260         *       280
nscaf2902  : CAAGCGTGGCGATTACGGCATAACCCCCCCTTATGGCAGCTACAAATAAAAAACGGCCCCCAGTTCCCGGCA : 288
nscaf2964  : CAAGCGTGGCGATTACGGCATAACCCCCCCTTATGGCAGCTACAAATAAAAAACGGCCCCCAGTTCCCGGCA : 288
preMITE    : CAAGCGTGGCGATTACGGCATAACCCCCCCTTATGGCAGCTACAAATAAAAAACGGCCCCCAGTTCCCGGCA : 119
nscaf2529  : CAAGCGTGGCGATTACGGCATAACCCCCCCTTATGGCAGCTACAAATAAAAAACGGCCCCCAGTTCCCGGCA : 287
scaffold35 : CAAGCGTGGCGATTACGGCATAACCCCCCCTTATGGCAGCTACAAATAAAAAACGGCCCCCAGTTCCCGGCA : 288
             CAAGCGTGGCGATTACGGCATAACCCCCCCTTATGGCAGCTACAAATAAAAAACGGCCCCCAGTTCCCGGCA

               *       300         *       320         *       340         *       360
nscaf2902  : GCTTTACTATCCCTGGCTACGGTAGCAGCCATGGTAACGGTAAGGCAGGGGGTGCGAAGAATCCCCGGGCTA : 360
nscaf2964  : GCTTTACTATCCCTGGCTACGGTAGCAGCCATGGTAACGGTAAGGCAGGGGGTGCGAAGAATCCCCGGGCTA : 360
preMITE    : GCTTTACTATCCCTGGCTACGGTAGCAGCCATGGTAACGGTAAGG--------------------------- : 164
nscaf2529  : GCTTTACTATCCCTGGCTACGGTAGCAGCCATGGTAACGGTAAGGCAGGGGGTGCGAAGAATCCCCGGGCTA : 359
scaffold35 : GCTTTACTATCCCTGGCTACGGTAGCAGCCATGGTAACGGTAAGGCAGGGGGTGCGAAGAATCCCCGGGCTA : 360
             GCTTTACTATCCCTGGCTACGGTAGCAGCCATGGTAACGGTAAGGcagggggtgcgaagaatccccgggcta

                 *       380         *       400         *       420
nscaf2902  : CGGACAGCTACCACCAAATGACCCTGGCGACTCTCAATACCCGGACTCTGCGGCTTGACG-------
nscaf2964  : CGGACAGCTACCACCAAATGACCCTGGCGACTCTCAATACCCGGACTCTGCGGCTTGACGAACATAT
preMITE    : -------------------------------------------------------------------
nscaf2529  : CGGACAGCTACCACCAAATGACCCTGGCGACTCTCAATACCCGGACTCTGCGGCTTGACGAACATAT
scaffold35 : CGGACAGCTACCACCAAATGACCCTGGCGACTCTCAATACCCGGACTCTGCGGCTTGACGAACATAT
             cggacagctaccaccaaatgaccctggcgactctcaatacccggactctgcggcttgacg
```

C
