# Supplementary material for: Burst expansion, distribution and diversification of MITEs in the silkworm genome
Source: BMC Genomics. 2010 Sep 27;11:520. doi: 10.1186/1471-2164-11-520 (PMC2997013; doi:10.1186/1471-2164-11-520)
Supplement: Additional file 2 — The information about insertion sites of the MITEs into scaffolds. [file 1471-2164-11-520-S2.PDF]

Positions of MITE Copies

Family	Nscaf	start	end	strand
	Full length copies			
Family	Nscaf	start	end	strand
BmMITE-1	nscaf2855	6436190	6435940	-
BmMITE-1	nscaf2998	1107532	1107264	-
BmMITE-1	nscaf2868	226766	226519	-
BmMITE-1	nscaf2829	349926	349671	-
BmMITE-1	nscaf3026	1140056	1139805	-
BmMITE-1	nscaf1732	2304	2046	-
BmMITE-1	nscaf3032	1781014	1780772	-
BmMITE-1	nscaf2943	1417684	1417425	-
BmMITE-1	nscaf2993	4983668	4983425	-
BmMITE-1	nscaf1071	281934	281674	-
BmMITE-1	nscaf2865	4028542	4028286	-
BmMITE-1	nscaf2655	1002506	1002251	-
BmMITE-1	nscaf2204	1773563	1773315	-
BmMITE-1	nscaf2983	3173480	3173222	-
BmMITE-1	nscaf2674	2766633	2766378	-
BmMITE-1	nscaf2962	366348	366092	-
BmMITE-1	scaffold1552	2827	2572	-
BmMITE-1	nscaf2916	535930	535675	-
BmMITE-1	nscaf2398	467673	467416	-
BmMITE-1	nscaf3055	1549347	1549092	-
BmMITE-1	nscaf2927	61542	61287	-
BmMITE-1	nscaf1108	3305655	3305399	-
BmMITE-1	nscaf463	236680	236432	-
BmMITE-1	nscaf2398	260792	261042	+
BmMITE-1	nscaf2823	3343154	3343404	+
BmMITE-1	nscaf2986	5110017	5110270	+
BmMITE-1	nscaf2853	2805086	2805340	+
BmMITE-1	nscaf2964	4576004	4576260	+
BmMITE-1	nscaf2930	1502500	1502744	+
BmMITE-1	nscaf2823	362883	363138	+
BmMITE-1	nscaf2136	1382304	1382559	+
BmMITE-1	nscaf3058	7290141	7290399	+
BmMITE-1	nscaf2655	1122438	1122691	+
BmMITE-1	nscaf3027	2882622	2882874	+
BmMITE-1	nscaf1681	5075358	5075613	+
BmMITE-1	nscaf3013	210009	210264	+
BmMITE-1	nscaf2931	492331	492587	+
BmMITE-1	nscaf2930	778661	778916	+
BmMITE-1	nscaf2899	638819	639072	+
BmMITE-1	nscaf3003	4428666	4428921	+

BmMITE-1	scaffold33163	211	466	+
BmMITE-1	nscaf2781	126688	126943	+
BmMITE-1	nscaf3053	120415	120670	+
BmMITE-2	nscaf2759	612079	611802	-
BmMITE-2	scaffold35457	442	165	-
BmMITE-2	scaffold2694	579	302	-
BmMITE-2	nscaf3098	1665880	1665604	-
BmMITE-2	nscaf3098	1528367	1528090	-
BmMITE-2	nscaf3098	1341987	1341710	-
BmMITE-2	nscaf3098	446835	446558	-
BmMITE-2	nscaf3098	144982	144708	-
BmMITE-2	nscaf306	1821	1544	-
BmMITE-2	nscaf3045	1965338	1965061	-
BmMITE-2	nscaf3045	1916795	1916518	-
BmMITE-2	nscaf3045	1807497	1807220	-
BmMITE-2	nscaf3045	1351316	1351039	-
BmMITE-2	nscaf3045	736370	736093	-
BmMITE-2	nscaf3045	298908	298631	-
BmMITE-2	nscaf3045	227376	227099	-
BmMITE-2	nscaf3033	200975	200698	-
BmMITE-2	nscaf3003	4695863	4695586	-
BmMITE-2	nscaf3003	4681109	4680832	-
BmMITE-2	nscaf3003	4651165	4650887	-
BmMITE-2	nscaf3003	4570656	4570379	-
BmMITE-2	nscaf3003	4256202	4255925	-
BmMITE-2	nscaf3003	4035497	4035221	-
BmMITE-2	nscaf3003	3651545	3651262	-
BmMITE-2	nscaf3003	3349706	3349429	-
BmMITE-2	nscaf3003	3280888	3280611	-
BmMITE-2	nscaf3003	2038717	2038441	-
BmMITE-2	nscaf3003	1695917	1695640	-
BmMITE-2	nscaf3003	763488	763211	-
BmMITE-2	nscaf3003	548237	547961	-
BmMITE-2	nscaf3003	25025	24748	-
BmMITE-2	nscaf2986	5219963	5219686	-
BmMITE-2	nscaf2986	4905673	4905396	-
BmMITE-2	nscaf2986	4052337	4052060	-
BmMITE-2	nscaf2986	3615929	3615652	-
BmMITE-2	nscaf2986	3002858	3002581	-
BmMITE-2	nscaf2986	2706094	2705807	-
BmMITE-2	nscaf2986	2588337	2588060	-
BmMITE-2	nscaf2986	2536116	2535836	-

BmMITE-2	nscaf2986	1934412	1934135	-
BmMITE-2	nscaf2986	1494347	1494070	-
BmMITE-2	nscaf2986	1354788	1354511	-
BmMITE-2	nscaf2986	122376	122101	-
BmMITE-2	nscaf2931	1780888	1780611	-
BmMITE-2	nscaf2931	1639690	1639413	-
BmMITE-2	nscaf2931	1340129	1339853	-
BmMITE-2	nscaf2931	1324688	1324411	-
BmMITE-2	nscaf2931	780964	780687	-
BmMITE-2	nscaf2931	575980	575704	-
BmMITE-2	nscaf2931	56549	56272	-
BmMITE-2	nscaf2931	9916	9640	-
BmMITE-2	nscaf2930	5500811	5500534	-
BmMITE-2	nscaf2930	5470929	5470652	-
BmMITE-2	nscaf2930	5280993	5280717	-
BmMITE-2	nscaf2930	4985837	4985560	-
BmMITE-2	nscaf2930	4974514	4974237	-
BmMITE-2	nscaf2930	4292264	4291987	-
BmMITE-2	nscaf2930	4109555	4109278	-
BmMITE-2	nscaf2930	3602889	3602613	-
BmMITE-2	nscaf2930	2276304	2276027	-
BmMITE-2	nscaf2930	1989229	1988952	-
BmMITE-2	nscaf2930	1954040	1953764	-
BmMITE-2	nscaf2930	1644299	1644022	-
BmMITE-2	nscaf2930	1390953	1390676	-
BmMITE-2	nscaf2930	1331702	1331425	-
BmMITE-2	nscaf2930	1273808	1273531	-
BmMITE-2	nscaf2930	1206294	1206017	-
BmMITE-2	nscaf2930	461337	461060	-
BmMITE-2	nscaf2930	90087	89810	-
BmMITE-2	nscaf2887	1992201	1991924	-
BmMITE-2	nscaf2887	1963515	1963235	-
BmMITE-2	nscaf2887	1931028	1930751	-
BmMITE-2	nscaf2887	1426439	1426163	-
BmMITE-2	nscaf2887	1197298	1197021	-
BmMITE-2	nscaf2887	1183845	1183569	-
BmMITE-2	nscaf2887	1028794	1028518	-
BmMITE-2	nscaf2887	921589	921312	-
BmMITE-2	nscaf2887	627110	626833	-
BmMITE-2	nscaf2887	372801	372524	-
BmMITE-2	nscaf2886	1024213	1023936	-
BmMITE-2	nscaf2886	938608	938331	-
BmMITE-2	nscaf2886	890937	890660	-

BmMITE-2	nscaf2886	605833	605565	-
BmMITE-2	nscaf2886	591962	591685	-
BmMITE-2	nscaf2886	351210	350933	-
BmMITE-2	nscaf2886	178147	177870	-
BmMITE-2	nscaf2847	8353223	8352946	-
BmMITE-2	nscaf2847	8298028	8297751	-
BmMITE-2	nscaf2847	8183716	8183439	-
BmMITE-2	nscaf2847	7476822	7476545	-
BmMITE-2	nscaf2847	7061976	7061699	-
BmMITE-2	nscaf2847	7025416	7025139	-
BmMITE-2	nscaf2847	6605277	6605000	-
BmMITE-2	nscaf2847	6454582	6454305	-
BmMITE-2	nscaf2847	5966677	5966400	-
BmMITE-2	nscaf2847	5874835	5874558	-
BmMITE-2	nscaf2847	5757126	5756849	-
BmMITE-2	nscaf2847	5557030	5556754	-
BmMITE-2	nscaf2847	5488819	5488542	-
BmMITE-2	nscaf2847	5470117	5469840	-
BmMITE-2	nscaf2847	5222018	5221741	-
BmMITE-2	nscaf2847	5058991	5058714	-
BmMITE-2	nscaf2847	4539456	4539179	-
BmMITE-2	nscaf2847	4133290	4133014	-
BmMITE-2	nscaf2847	3765062	3764785	-
BmMITE-2	nscaf2847	3686382	3686105	-
BmMITE-2	nscaf2847	3068422	3068145	-
BmMITE-2	nscaf2847	2637644	2637367	-
BmMITE-2	nscaf2847	2095004	2094727	-
BmMITE-2	nscaf2847	1807274	1806997	-
BmMITE-2	nscaf2847	1703085	1702809	-
BmMITE-2	nscaf2847	1596991	1596714	-
BmMITE-2	nscaf2847	1252710	1252433	-
BmMITE-2	nscaf2847	1235944	1235667	-
BmMITE-2	nscaf2847	214291	214014	-
BmMITE-2	nscaf2827	1433130	1432853	-
BmMITE-2	nscaf2827	857300	857023	-
BmMITE-2	nscaf2827	832407	832130	-
BmMITE-2	nscaf2826	481783	481506	-
BmMITE-2	nscaf2826	310468	310191	-
BmMITE-2	nscaf2826	200098	199821	-
BmMITE-2	nscaf2818	2805458	2805181	-
BmMITE-2	nscaf2818	2569425	2569148	-
BmMITE-2	nscaf2818	1941051	1940774	-
BmMITE-2	nscaf2818	1865450	1865173	-

BmMITE-2	nscaf2818	1061843	1059002	-
BmMITE-2	nscaf2818	891038	890761	-
BmMITE-2	nscaf2818	801249	800972	-
BmMITE-2	nscaf2818	120992	120691	-
BmMITE-2	nscaf2818	3132	2855	-
BmMITE-2	nscaf2781	181227	180951	-
BmMITE-2	nscaf2781	125752	125475	-
BmMITE-2	nscaf2781	42166	41890	-
BmMITE-2	nscaf2767	4188576	4188299	-
BmMITE-2	nscaf2767	4071089	4070799	-
BmMITE-2	nscaf2767	3362971	3362694	-
BmMITE-2	nscaf2767	3092348	3092072	-
BmMITE-2	nscaf2767	2456964	2456687	-
BmMITE-2	nscaf2767	2089145	2088868	-
BmMITE-2	nscaf2767	1858158	1857881	-
BmMITE-2	nscaf2767	1137027	1136752	-
BmMITE-2	nscaf2767	1105240	1104963	-
BmMITE-2	nscaf2767	1061399	1061123	-
BmMITE-2	nscaf2767	826326	826049	-
BmMITE-2	nscaf2767	583099	582822	-
BmMITE-2	nscaf2767	30752	30477	-
BmMITE-2	nscaf2623	1318875	1318598	-
BmMITE-2	nscaf2623	990803	990526	-
BmMITE-2	nscaf2623	921571	921294	-
BmMITE-2	nscaf2623	817170	816893	-
BmMITE-2	nscaf2623	627380	626447	-
BmMITE-2	nscaf2529	4549175	4548898	-
BmMITE-2	nscaf2529	4230075	4229794	-
BmMITE-2	nscaf2529	3585497	3585220	-
BmMITE-2	nscaf2529	2944797	2944520	-
BmMITE-2	nscaf2529	2563083	2562806	-
BmMITE-2	nscaf2529	1842821	1842544	-
BmMITE-2	nscaf2529	1689517	1689240	-
BmMITE-2	nscaf2529	1556730	1556453	-
BmMITE-2	nscaf2529	1527926	1527649	-
BmMITE-2	nscaf2529	1471421	1471144	-
BmMITE-2	nscaf2529	619161	618884	-
BmMITE-2	nscaf2529	367143	366866	-
BmMITE-2	nscaf2529	154767	154490	-
BmMITE-2	nscaf2529	128827	128550	-
BmMITE-2	nscaf2529	115908	115631	-
BmMITE-2	nscaf2330	4186662	4186385	-
BmMITE-2	nscaf2330	4081565	4081288	-

BmMITE-2	nscaf2330	3993734	3993458	-
BmMITE-2	nscaf2330	3969223	3968946	-
BmMITE-2	nscaf2330	3498491	3498214	-
BmMITE-2	nscaf2330	3421286	3421009	-
BmMITE-2	nscaf2330	3253500	3253224	-
BmMITE-2	nscaf2330	3151729	3151452	-
BmMITE-2	nscaf2330	2384262	2383985	-
BmMITE-2	nscaf2330	2313695	2313416	-
BmMITE-2	nscaf2330	1582553	1582276	-
BmMITE-2	nscaf2330	1440970	1440693	-
BmMITE-2	nscaf2330	1407740	1407463	-
BmMITE-2	nscaf2330	1125070	1124793	-
BmMITE-2	nscaf2330	767694	767418	-
BmMITE-2	nscaf2330	219457	219180	-
BmMITE-2	nscaf2330	197478	197201	-
BmMITE-2	nscaf2053	642263	641958	-
BmMITE-2	nscaf2053	598382	598105	-
BmMITE-2	nscaf2053	377216	376938	-
BmMITE-2	nscaf2053	157062	156785	-
BmMITE-2	nscaf1108	3168422	3168147	-
BmMITE-2	nscaf1108	2890268	2889991	-
BmMITE-2	nscaf1108	2620918	2620641	-
BmMITE-2	nscaf1108	2003687	2003410	-
BmMITE-2	nscaf1108	1926938	1926656	-
BmMITE-2	nscaf1108	1873722	1873445	-
BmMITE-2	nscaf1108	1543998	1543721	-
BmMITE-2	nscaf1108	1491701	1491424	-
BmMITE-2	nscaf1108	1301066	1300789	-
BmMITE-2	nscaf1108	1058784	1058352	-
BmMITE-2	nscaf1108	932749	932472	-
BmMITE-2	nscaf1108	621842	621565	-
BmMITE-2	nscaf1108	610687	610410	-
BmMITE-2	nscaf1108	528336	528059	-
BmMITE-2	nscaf1108	436535	436258	-
BmMITE-2	nscaf1108	421587	421310	-
BmMITE-2	nscaf1071	362834	362557	-
BmMITE-2	nscaf1071	331547	331269	-
BmMITE-2	nscaf2888	8407179	8406902	-
BmMITE-2	nscaf2888	8046940	8046285	-
BmMITE-2	nscaf2888	7413625	7413356	-
BmMITE-2	nscaf2888	7192935	7192658	-
BmMITE-2	nscaf2888	6991374	6991097	-
BmMITE-2	nscaf2888	6576633	6576356	-

BmMITE-2	nscaf2888	6279728	6279451	-
BmMITE-2	nscaf2888	6046334	6046057	-
BmMITE-2	nscaf2888	5405046	5404767	-
BmMITE-2	nscaf2888	5241926	5241649	-
BmMITE-2	nscaf2888	4731871	4731594	-
BmMITE-2	nscaf2888	4537398	4537121	-
BmMITE-2	nscaf2888	4406707	4406431	-
BmMITE-2	nscaf2888	4326696	4325661	-
BmMITE-2	nscaf2888	3704074	3703797	-
BmMITE-2	nscaf2888	3516860	3516583	-
BmMITE-2	nscaf2888	2927502	2927225	-
BmMITE-2	nscaf2888	2846077	2845800	-
BmMITE-2	nscaf2888	2135059	2134782	-
BmMITE-2	nscaf2888	1475803	1475526	-
BmMITE-2	nscaf2888	1459154	1458877	-
BmMITE-2	nscaf2888	1089059	1088782	-
BmMITE-2	nscaf2888	645058	644782	-
BmMITE-2	nscaf2888	491013	490736	-
BmMITE-2	nscaf2888	291797	291520	-
BmMITE-2	nscaf2865	5239926	5239649	-
BmMITE-2	nscaf2865	4988895	4988618	-
BmMITE-2	nscaf2865	4820529	4820252	-
BmMITE-2	nscaf2865	4534873	4534596	-
BmMITE-2	nscaf2865	3848841	3848565	-
BmMITE-2	nscaf2865	3681948	3681671	-
BmMITE-2	nscaf2865	3647296	3647019	-
BmMITE-2	nscaf2865	3550286	3550009	-
BmMITE-2	nscaf2865	3338619	3338342	-
BmMITE-2	nscaf2865	3055161	3054884	-
BmMITE-2	nscaf2865	2632510	2632233	-
BmMITE-2	nscaf2865	2463247	2462971	-
BmMITE-2	nscaf2865	2210144	2209870	-
BmMITE-2	nscaf2865	1784357	1784080	-
BmMITE-2	nscaf2865	1515918	1515641	-
BmMITE-2	nscaf2865	829952	829675	-
BmMITE-2	nscaf2865	586284	586007	-
BmMITE-2	nscaf2575	4300602	4300325	-
BmMITE-2	nscaf2575	3909043	3908767	-
BmMITE-2	nscaf2575	3864819	3864542	-
BmMITE-2	nscaf2575	3601956	3601679	-
BmMITE-2	nscaf2575	3567007	3566730	-
BmMITE-2	nscaf2575	3495511	3495234	-
BmMITE-2	nscaf2575	3205908	3205631	-

BmMITE-2	nscaf2575	2399506	2399229	-
BmMITE-2	nscaf2575	2286705	2286428	-
BmMITE-2	nscaf2575	1736645	1736358	-
BmMITE-2	nscaf2575	1683928	1683651	-
BmMITE-2	nscaf2575	940484	940207	-
BmMITE-2	nscaf2575	131324	131047	-
BmMITE-2	nscaf2953	2390139	2389862	-
BmMITE-2	nscaf2953	2241224	2240947	-
BmMITE-2	nscaf2953	2138792	2138515	-
BmMITE-2	nscaf2953	1974100	1973823	-
BmMITE-2	nscaf2953	762681	762405	-
BmMITE-2	scaffold8654	683	406	-
BmMITE-2	scaffold836	5108	4831	-
BmMITE-2	scaffold7833	283	6	-
BmMITE-2	scaffold35112	480	203	-
BmMITE-2	scaffold12443	280	3	-
BmMITE-2	scaffold1024	8293	8016	-
BmMITE-2	nscaf481	1327877	1327600	-
BmMITE-2	nscaf481	837451	837173	-
BmMITE-2	nscaf481	783381	783104	-
BmMITE-2	nscaf481	636658	636381	-
BmMITE-2	nscaf481	492209	491932	-
BmMITE-2	nscaf481	357104	356370	-
BmMITE-2	nscaf481	332160	331883	-
BmMITE-2	nscaf481	80514	80237	-
BmMITE-2	nscaf3099	3188733	3188456	-
BmMITE-2	nscaf3099	3144574	3144297	-
BmMITE-2	nscaf3099	2683874	2683597	-
BmMITE-2	nscaf3099	2565782	2565505	-
BmMITE-2	nscaf3099	1853062	1852784	-
BmMITE-2	nscaf3099	1636774	1636483	-
BmMITE-2	nscaf3099	1538530	1538253	-
BmMITE-2	nscaf3099	1373484	1373207	-
BmMITE-2	nscaf3099	1219489	1219217	-
BmMITE-2	nscaf3099	1117541	1117264	-
BmMITE-2	nscaf3099	966669	966392	-
BmMITE-2	nscaf3099	814471	814194	-
BmMITE-2	nscaf3099	622521	622240	-
BmMITE-2	nscaf3099	420192	419915	-
BmMITE-2	nscaf3099	360358	360082	-
BmMITE-2	nscaf3099	137693	137416	-
BmMITE-2	nscaf3097	3146346	3146069	-
BmMITE-2	nscaf3097	2470813	2470536	-

BmMITE-2	nscaf3097	2373001	2372725	-
BmMITE-2	nscaf3097	2252492	2252215	-
BmMITE-2	nscaf3097	2195604	2195327	-
BmMITE-2	nscaf3097	1548706	1548429	-
BmMITE-2	nscaf3097	1357176	1356898	-
BmMITE-2	nscaf3097	1063024	1062747	-
BmMITE-2	nscaf3097	1035390	1035112	-
BmMITE-2	nscaf3097	824875	824602	-
BmMITE-2	nscaf3097	468918	468641	-
BmMITE-2	nscaf3097	108952	108675	-
BmMITE-2	nscaf3089	479547	479270	-
BmMITE-2	nscaf3089	344174	343897	-
BmMITE-2	nscaf3089	241905	241628	-
BmMITE-2	nscaf3089	2714	2437	-
BmMITE-2	nscaf3062	438810	438541	-
BmMITE-2	nscaf3062	389735	389458	-
BmMITE-2	nscaf3062	366858	366581	-
BmMITE-2	nscaf3058	8841463	8841186	-
BmMITE-2	nscaf3058	8710820	8710543	-
BmMITE-2	nscaf3058	8489078	8488801	-
BmMITE-2	nscaf3058	8270455	8270099	-
BmMITE-2	nscaf3058	8022074	8021797	-
BmMITE-2	nscaf3058	7821482	7821204	-
BmMITE-2	nscaf3058	7491973	7491696	-
BmMITE-2	nscaf3058	7391597	7391321	-
BmMITE-2	nscaf3058	7322824	7322547	-
BmMITE-2	nscaf3058	7116307	7116030	-
BmMITE-2	nscaf3058	7085348	7085072	-
BmMITE-2	nscaf3058	7007114	7006837	-
BmMITE-2	nscaf3058	6955361	6955079	-
BmMITE-2	nscaf3058	6891844	6891567	-
BmMITE-2	nscaf3058	6645195	6644918	-
BmMITE-2	nscaf3058	6293871	6293594	-
BmMITE-2	nscaf3058	5956774	5956497	-
BmMITE-2	nscaf3058	5922361	5922084	-
BmMITE-2	nscaf3058	5886326	5886049	-
BmMITE-2	nscaf3058	5434488	5434211	-
BmMITE-2	nscaf3058	5356791	5356514	-
BmMITE-2	nscaf3058	4035742	4035461	-
BmMITE-2	nscaf3058	3576707	3576430	-
BmMITE-2	nscaf3058	3508159	3507882	-
BmMITE-2	nscaf3058	3247204	3246926	-
BmMITE-2	nscaf3058	3187793	3187518	-

BmMITE-2	nscaf3058	2656992	2656715	-
BmMITE-2	nscaf3058	2594087	2593810	-
BmMITE-2	nscaf3058	1088539	1088257	-
BmMITE-2	nscaf3058	516328	516052	-
BmMITE-2	nscaf3058	159515	159237	-
BmMITE-2	nscaf3058	141350	141073	-
BmMITE-2	nscaf3058	23234	22957	-
BmMITE-2	nscaf3035	996575	996298	-
BmMITE-2	nscaf3035	707935	707658	-
BmMITE-2	nscaf3035	599933	599657	-
BmMITE-2	nscaf3035	234450	234176	-
BmMITE-2	nscaf3035	143526	143249	-
BmMITE-2	nscaf3032	1730035	1729757	-
BmMITE-2	nscaf3032	1357460	1357183	-
BmMITE-2	nscaf3032	1232947	1232670	-
BmMITE-2	nscaf3032	1004274	1003997	-
BmMITE-2	nscaf3032	671654	671377	-
BmMITE-2	nscaf3031	5829844	5829568	-
BmMITE-2	nscaf3031	5446284	5445975	-
BmMITE-2	nscaf3031	4525016	4524739	-
BmMITE-2	nscaf3031	4294706	4294429	-
BmMITE-2	nscaf3031	3976340	3976063	-
BmMITE-2	nscaf3031	3374587	3374310	-
BmMITE-2	nscaf3031	3317663	3317392	-
BmMITE-2	nscaf3031	2847935	2847658	-
BmMITE-2	nscaf3031	2787154	2786877	-
BmMITE-2	nscaf3031	2607748	2607455	-
BmMITE-2	nscaf3031	2506380	2506103	-
BmMITE-2	nscaf3031	2223090	2222813	-
BmMITE-2	nscaf3031	2019234	2018957	-
BmMITE-2	nscaf3031	1807032	1806755	-
BmMITE-2	nscaf3031	1762689	1762412	-
BmMITE-2	nscaf3031	1184339	1184062	-
BmMITE-2	nscaf3031	431591	431314	-
BmMITE-2	nscaf3031	31864	31587	-
BmMITE-2	nscaf3026	6589755	6589468	-
BmMITE-2	nscaf3026	6381639	6381356	-
BmMITE-2	nscaf3026	5579885	5579609	-
BmMITE-2	nscaf3026	5525113	5524836	-
BmMITE-2	nscaf3026	5345201	5344924	-
BmMITE-2	nscaf3026	4746440	4746163	-
BmMITE-2	nscaf3026	4319311	4319034	-
BmMITE-2	nscaf3026	4236381	4236104	-

BmMITE-2	nscaf3026	4048095	4047818	-
BmMITE-2	nscaf3026	3900746	3900474	-
BmMITE-2	nscaf3026	3468038	3467762	-
BmMITE-2	nscaf3026	3294351	3294074	-
BmMITE-2	nscaf3026	3249307	3249030	-
BmMITE-2	nscaf3026	3092501	3092224	-
BmMITE-2	nscaf3026	3024987	3024712	-
BmMITE-2	nscaf3026	2911550	2911273	-
BmMITE-2	nscaf3026	2858737	2858460	-
BmMITE-2	nscaf3026	2688876	2688599	-
BmMITE-2	nscaf3026	2253378	2253101	-
BmMITE-2	nscaf3026	2135014	2134737	-
BmMITE-2	nscaf3026	2039171	2038894	-
BmMITE-2	nscaf3026	1235697	1235426	-
BmMITE-2	nscaf3026	1185916	1185639	-
BmMITE-2	nscaf3026	1156487	1156210	-
BmMITE-2	nscaf3026	745466	745189	-
BmMITE-2	nscaf3026	661089	660812	-
BmMITE-2	nscaf3022	507298	507021	-
BmMITE-2	nscaf2998	1591883	1591606	-
BmMITE-2	nscaf2998	1393078	1392801	-
BmMITE-2	nscaf2998	1288606	1288329	-
BmMITE-2	nscaf2998	1084842	1084565	-
BmMITE-2	nscaf2998	868097	867820	-
BmMITE-2	nscaf2998	614076	613799	-
BmMITE-2	nscaf2998	451718	451441	-
BmMITE-2	nscaf2983	3089342	3089065	-
BmMITE-2	nscaf2983	2872769	2872492	-
BmMITE-2	nscaf2983	2145228	2144951	-
BmMITE-2	nscaf2983	1782335	1782058	-
BmMITE-2	nscaf2983	997708	997433	-
BmMITE-2	nscaf2983	590913	590636	-
BmMITE-2	nscaf2983	516676	516399	-
BmMITE-2	nscaf2983	41948	41671	-
BmMITE-2	nscaf2964	4920489	4920212	-
BmMITE-2	nscaf2964	4899598	4899321	-
BmMITE-2	nscaf2964	4817222	4816945	-
BmMITE-2	nscaf2964	4792988	4792712	-
BmMITE-2	nscaf2964	4426843	4426562	-
BmMITE-2	nscaf2964	4355511	4355240	-
BmMITE-2	nscaf2964	4342743	4342462	-
BmMITE-2	nscaf2964	4020095	4019827	-
BmMITE-2	nscaf2964	3960863	3960592	-

BmMITE-2	nscaf2964	3894209	3893932	-
BmMITE-2	nscaf2964	3749223	3748946	-
BmMITE-2	nscaf2964	3472704	3472427	-
BmMITE-2	nscaf2964	3396276	3395999	-
BmMITE-2	nscaf2964	3241586	3241309	-
BmMITE-2	nscaf2964	3138457	3138178	-
BmMITE-2	nscaf2964	3051376	3051099	-
BmMITE-2	nscaf2964	2637947	2637666	-
BmMITE-2	nscaf2964	1854921	1854644	-
BmMITE-2	nscaf2964	1763002	1762723	-
BmMITE-2	nscaf2964	1725616	1725339	-
BmMITE-2	nscaf2964	1631979	1631703	-
BmMITE-2	nscaf2964	1449202	1448925	-
BmMITE-2	nscaf2964	1394828	1394553	-
BmMITE-2	nscaf2964	1300786	1300509	-
BmMITE-2	nscaf2964	1226552	1226275	-
BmMITE-2	nscaf2964	1175160	1174883	-
BmMITE-2	nscaf2964	741769	741494	-
BmMITE-2	nscaf2964	635234	634957	-
BmMITE-2	nscaf2964	392652	392379	-
BmMITE-2	nscaf2964	298180	297906	-
BmMITE-2	nscaf2951	1384807	1384531	-
BmMITE-2	nscaf2951	1344153	1343877	-
BmMITE-2	nscaf2951	988137	987869	-
BmMITE-2	nscaf2951	971968	971692	-
BmMITE-2	nscaf2951	637817	637539	-
BmMITE-2	nscaf2951	607294	607017	-
BmMITE-2	nscaf2951	20493	20216	-
BmMITE-2	nscaf2943	3781392	3781115	-
BmMITE-2	nscaf2943	1788198	1787922	-
BmMITE-2	nscaf2943	1263423	1263147	-
BmMITE-2	nscaf2943	1043639	1043363	-
BmMITE-2	nscaf2943	983157	982880	-
BmMITE-2	nscaf2943	929686	929410	-
BmMITE-2	nscaf2943	273546	273269	-
BmMITE-2	nscaf2943	117045	116768	-
BmMITE-2	nscaf2927	188388	188111	-
BmMITE-2	nscaf2903	1423144	1422867	-
BmMITE-2	nscaf2903	947522	947245	-
BmMITE-2	nscaf2902	10722479	10722202	-
BmMITE-2	nscaf2902	10458424	10458148	-
BmMITE-2	nscaf2902	9959802	9959526	-
BmMITE-2	nscaf2902	9602958	9602682	-

BmMITE-2	nscaf2902	9182518	9182241	-
BmMITE-2	nscaf2902	8702091	8701814	-
BmMITE-2	nscaf2902	8560223	8559946	-
BmMITE-2	nscaf2902	8162726	8162449	-
BmMITE-2	nscaf2902	7987512	7987235	-
BmMITE-2	nscaf2902	7424497	7424220	-
BmMITE-2	nscaf2902	6529788	6529511	-
BmMITE-2	nscaf2902	6421481	6421204	-
BmMITE-2	nscaf2902	6323803	6323526	-
BmMITE-2	nscaf2902	6262720	6262443	-
BmMITE-2	nscaf2902	6087617	6087340	-
BmMITE-2	nscaf2902	5438746	5438469	-
BmMITE-2	nscaf2902	4682543	4682266	-
BmMITE-2	nscaf2902	4571007	4570730	-
BmMITE-2	nscaf2902	3965730	3965453	-
BmMITE-2	nscaf2902	3748683	3748406	-
BmMITE-2	nscaf2902	3679516	3679239	-
BmMITE-2	nscaf2902	3602366	3602089	-
BmMITE-2	nscaf2902	3426154	3425877	-
BmMITE-2	nscaf2902	3119761	3119484	-
BmMITE-2	nscaf2902	2840759	2840482	-
BmMITE-2	nscaf2902	2151306	2151034	-
BmMITE-2	nscaf2902	2115988	2115711	-
BmMITE-2	nscaf2902	1964344	1964067	-
BmMITE-2	nscaf2902	1845566	1845289	-
BmMITE-2	nscaf2902	1042620	1042342	-
BmMITE-2	nscaf2902	786677	786400	-
BmMITE-2	nscaf2902	753969	753692	-
BmMITE-2	nscaf2902	677528	677251	-
BmMITE-2	nscaf2902	494469	494191	-
BmMITE-2	nscaf2902	375424	375147	-
BmMITE-2	nscaf2902	95463	95186	-
BmMITE-2	nscaf2901	1611410	1611133	-
BmMITE-2	nscaf2901	1377889	1377612	-
BmMITE-2	nscaf2901	225543	225266	-
BmMITE-2	nscaf2901	105100	104823	-
BmMITE-2	nscaf2876	1485221	1484944	-
BmMITE-2	nscaf2876	1416810	1416533	-
BmMITE-2	nscaf2876	856948	856631	-
BmMITE-2	nscaf2868	359892	359615	-
BmMITE-2	nscaf2868	242888	242610	-
BmMITE-2	nscaf2868	93414	93137	-
BmMITE-2	nscaf2860	2828603	2828326	-

BmMITE-2	nscaf2860	2646260	2645983	-
BmMITE-2	nscaf2860	2609720	2609443	-
BmMITE-2	nscaf2860	1485316	1485039	-
BmMITE-2	nscaf2860	410496	410219	-
BmMITE-2	nscaf2855	7111695	7111418	-
BmMITE-2	nscaf2855	6130206	6129845	-
BmMITE-2	nscaf2855	5853633	5853356	-
BmMITE-2	nscaf2855	5788426	5788150	-
BmMITE-2	nscaf2855	5734918	5734641	-
BmMITE-2	nscaf2855	5021936	5021659	-
BmMITE-2	nscaf2855	4508539	4508262	-
BmMITE-2	nscaf2855	4456115	4455838	-
BmMITE-2	nscaf2855	4427423	4427146	-
BmMITE-2	nscaf2855	3473754	3473477	-
BmMITE-2	nscaf2855	2911367	2911090	-
BmMITE-2	nscaf2855	2743080	2742803	-
BmMITE-2	nscaf2855	2111726	2111449	-
BmMITE-2	nscaf2855	1448168	1447891	-
BmMITE-2	nscaf2855	1402557	1402280	-
BmMITE-2	nscaf2855	1354678	1354401	-
BmMITE-2	nscaf2855	1221027	1220752	-
BmMITE-2	nscaf2855	916426	916149	-
BmMITE-2	nscaf2853	6804585	6804308	-
BmMITE-2	nscaf2853	6692816	6692539	-
BmMITE-2	nscaf2853	6348780	6348503	-
BmMITE-2	nscaf2853	5914411	5914134	-
BmMITE-2	nscaf2853	4303924	4303647	-
BmMITE-2	nscaf2853	2794947	2794671	-
BmMITE-2	nscaf2853	2260549	2260273	-
BmMITE-2	nscaf2853	1823295	1823018	-
BmMITE-2	nscaf2853	1291265	1290989	-
BmMITE-2	nscaf2853	1012037	1011760	-
BmMITE-2	nscaf2853	415871	415594	-
BmMITE-2	nscaf2842	1950038	1949761	-
BmMITE-2	nscaf2842	1446738	1446462	-
BmMITE-2	nscaf2842	106451	106174	-
BmMITE-2	nscaf2839	473174	472897	-
BmMITE-2	nscaf2839	367328	367051	-
BmMITE-2	nscaf2839	297325	297049	-
BmMITE-2	nscaf2829	4402556	4402279	-
BmMITE-2	nscaf2829	4259526	4259249	-
BmMITE-2	nscaf2829	4219914	4219637	-
BmMITE-2	nscaf2829	4092282	4092006	-

BmMITE-2	nscaf2829	3836672	3836398	-
BmMITE-2	nscaf2829	3143522	3143255	-
BmMITE-2	nscaf2829	2792896	2792619	-
BmMITE-2	nscaf2829	2277403	2277127	-
BmMITE-2	nscaf2829	2263937	2263660	-
BmMITE-2	nscaf2829	2222110	2221833	-
BmMITE-2	nscaf2829	2192096	2191819	-
BmMITE-2	nscaf2829	2041577	2041300	-
BmMITE-2	nscaf2829	996683	996409	-
BmMITE-2	nscaf2829	820379	820102	-
BmMITE-2	nscaf2829	53512	53235	-
BmMITE-2	nscaf2828	5127189	5126913	-
BmMITE-2	nscaf2828	4990509	4990232	-
BmMITE-2	nscaf2828	4558004	4557727	-
BmMITE-2	nscaf2828	4480159	4479876	-
BmMITE-2	nscaf2828	4343627	4343350	-
BmMITE-2	nscaf2828	3372214	3371937	-
BmMITE-2	nscaf2828	3319231	3318954	-
BmMITE-2	nscaf2828	3151626	3151349	-
BmMITE-2	nscaf2828	2660780	2660503	-
BmMITE-2	nscaf2828	2556908	2556631	-
BmMITE-2	nscaf2828	2026623	2026346	-
BmMITE-2	nscaf2828	1989907	1989630	-
BmMITE-2	nscaf2828	1594623	1594345	-
BmMITE-2	nscaf2828	1311978	1311702	-
BmMITE-2	nscaf2828	1100615	1100338	-
BmMITE-2	nscaf2828	757257	756980	-
BmMITE-2	nscaf2828	596357	596080	-
BmMITE-2	nscaf2828	495763	495486	-
BmMITE-2	nscaf2828	324425	324148	-
BmMITE-2	nscaf2823	4369660	4369383	-
BmMITE-2	nscaf2823	4320404	4320127	-
BmMITE-2	nscaf2823	4007702	4007421	-
BmMITE-2	nscaf2823	2699236	2698959	-
BmMITE-2	nscaf2823	2688308	2688031	-
BmMITE-2	nscaf2823	2195394	2195117	-
BmMITE-2	nscaf2823	2041997	2041720	-
BmMITE-2	nscaf2823	1854863	1854586	-
BmMITE-2	nscaf2823	1237323	1237046	-
BmMITE-2	nscaf2823	1202434	1202157	-
BmMITE-2	nscaf2823	897002	896725	-
BmMITE-2	nscaf2823	816614	816337	-
BmMITE-2	nscaf2823	192184	191908	-

BmMITE-2	nscaf2822	1576100	1575823	-
BmMITE-2	nscaf2822	827062	826785	-
BmMITE-2	nscaf2822	317540	317263	-
BmMITE-2	nscaf2822	257547	257279	-
BmMITE-2	nscaf2822	212970	212693	-
BmMITE-2	nscaf2822	20066	19789	-
BmMITE-2	nscaf2800	1881043	1880767	-
BmMITE-2	nscaf2800	1106188	1105911	-
BmMITE-2	nscaf2800	1046957	1046680	-
BmMITE-2	nscaf2800	963107	962830	-
BmMITE-2	nscaf2800	945933	945659	-
BmMITE-2	nscaf2800	756477	756200	-
BmMITE-2	nscaf2800	330276	329999	-
BmMITE-2	nscaf2800	263376	263099	-
BmMITE-2	nscaf2800	199185	198908	-
BmMITE-2	nscaf2800	144692	144415	-
BmMITE-2	nscaf2800	128408	128131	-
BmMITE-2	nscaf2800	24038	23761	-
BmMITE-2	nscaf2795	4104526	4104249	-
BmMITE-2	nscaf2795	3807867	3807590	-
BmMITE-2	nscaf2795	3604451	3604174	-
BmMITE-2	nscaf2795	3269685	3269408	-
BmMITE-2	nscaf2795	3170938	3170662	-
BmMITE-2	nscaf2795	2874882	2874605	-
BmMITE-2	nscaf2795	2655499	2655222	-
BmMITE-2	nscaf2795	2412895	2412619	-
BmMITE-2	nscaf2795	2381452	2381175	-
BmMITE-2	nscaf2795	2182668	2182391	-
BmMITE-2	nscaf2795	2115475	2115198	-
BmMITE-2	nscaf2795	1858819	1858542	-
BmMITE-2	nscaf2795	1785989	1785712	-
BmMITE-2	nscaf2795	1555608	1555327	-
BmMITE-2	nscaf2795	1469110	1468833	-
BmMITE-2	nscaf2795	1100342	1100065	-
BmMITE-2	nscaf2795	914041	913764	-
BmMITE-2	nscaf2795	873933	873656	-
BmMITE-2	nscaf2789	1422189	1421913	-
BmMITE-2	nscaf2789	1401351	1401074	-
BmMITE-2	nscaf2789	1240959	1240682	-
BmMITE-2	nscaf2789	1149762	1149485	-
BmMITE-2	nscaf2789	1086766	1086490	-
BmMITE-2	nscaf2789	988275	987998	-
BmMITE-2	nscaf2789	961144	960867	-

BmMITE-2	nscaf2789	172951	172675	-
BmMITE-2	nscaf2789	17270	16993	-
BmMITE-2	nscaf2789	4100	3823	-
BmMITE-2	nscaf2770	1334477	1334200	-
BmMITE-2	nscaf2770	1155761	1155484	-
BmMITE-2	nscaf2770	1034742	1034466	-
BmMITE-2	nscaf2770	993716	993439	-
BmMITE-2	nscaf2770	802754	802477	-
BmMITE-2	nscaf2770	696296	696019	-
BmMITE-2	nscaf2770	682374	682097	-
BmMITE-2	nscaf2770	250915	250638	-
BmMITE-2	nscaf2763	27452	27175	-
BmMITE-2	nscaf2655	2671376	2671099	-
BmMITE-2	nscaf2655	2638877	2638600	-
BmMITE-2	nscaf2655	2627496	2627212	-
BmMITE-2	nscaf2655	1794367	1794090	-
BmMITE-2	nscaf2655	1496733	1496456	-
BmMITE-2	nscaf2655	1435444	1435167	-
BmMITE-2	nscaf2655	1124295	1124018	-
BmMITE-2	nscaf2655	1008192	1007915	-
BmMITE-2	nscaf2655	919723	919446	-
BmMITE-2	nscaf2655	158048	157771	-
BmMITE-2	nscaf2655	146120	145843	-
BmMITE-2	nscaf2655	133382	133107	-
BmMITE-2	nscaf2655	9310	9033	-
BmMITE-2	nscaf2589	6605340	6605063	-
BmMITE-2	nscaf2589	5968440	5968162	-
BmMITE-2	nscaf2589	5672258	5671981	-
BmMITE-2	nscaf2589	5650299	5650022	-
BmMITE-2	nscaf2589	5334612	5334335	-
BmMITE-2	nscaf2589	5270647	5270370	-
BmMITE-2	nscaf2589	4617631	4617354	-
BmMITE-2	nscaf2589	4570510	4570233	-
BmMITE-2	nscaf2589	4374173	4373898	-
BmMITE-2	nscaf2589	4113072	4112795	-
BmMITE-2	nscaf2589	4098723	4098446	-
BmMITE-2	nscaf2589	3519257	3518980	-
BmMITE-2	nscaf2589	2627490	2627213	-
BmMITE-2	nscaf2589	2579884	2579607	-
BmMITE-2	nscaf2589	2524336	2524059	-
BmMITE-2	nscaf2589	1206231	1205954	-
BmMITE-2	nscaf2589	978048	977771	-
BmMITE-2	nscaf2511	6242157	6241880	-

BmMITE-2	nscaf2511	5442257	5441987	-
BmMITE-2	nscaf2511	5111121	5110844	-
BmMITE-2	nscaf2511	5009027	5008750	-
BmMITE-2	nscaf2511	4731186	4727827	-
BmMITE-2	nscaf2511	3804243	3803966	-
BmMITE-2	nscaf2511	3491182	3490905	-
BmMITE-2	nscaf2511	3441532	3441255	-
BmMITE-2	nscaf2511	3271233	3270956	-
BmMITE-2	nscaf2511	3221572	3221295	-
BmMITE-2	nscaf2511	3150110	3149833	-
BmMITE-2	nscaf2511	2911131	2910855	-
BmMITE-2	nscaf2511	2871109	2870832	-
BmMITE-2	nscaf2511	2612316	2612039	-
BmMITE-2	nscaf2511	2532218	2531941	-
BmMITE-2	nscaf2511	2300707	2300430	-
BmMITE-2	nscaf2511	2073795	2073518	-
BmMITE-2	nscaf2511	1984201	1983924	-
BmMITE-2	nscaf2511	1818267	1817990	-
BmMITE-2	nscaf2204	4290437	4290162	-
BmMITE-2	nscaf2204	4198474	4198197	-
BmMITE-2	nscaf2204	4007038	4006761	-
BmMITE-2	nscaf2204	3518451	3518175	-
BmMITE-2	nscaf2204	3468020	3467748	-
BmMITE-2	nscaf2204	3398388	3398111	-
BmMITE-2	nscaf2204	3040306	3040029	-
BmMITE-2	nscaf2204	1872857	1872580	-
BmMITE-2	nscaf2204	939608	939331	-
BmMITE-2	nscaf2204	896467	896191	-
BmMITE-2	nscaf2204	870328	870051	-
BmMITE-2	nscaf2204	814674	814397	-
BmMITE-2	nscaf2204	648677	648400	-
BmMITE-2	nscaf2204	409725	409448	-
BmMITE-2	nscaf2176	3241401	3241124	-
BmMITE-2	nscaf2176	3179540	3179263	-
BmMITE-2	nscaf2176	2564380	2564103	-
BmMITE-2	nscaf2176	2499119	2498842	-
BmMITE-2	nscaf2176	2295226	2294949	-
BmMITE-2	nscaf2176	2251069	2250792	-
BmMITE-2	nscaf2176	2067293	2067016	-
BmMITE-2	nscaf2176	1964374	1964097	-
BmMITE-2	nscaf2176	1561428	1561151	-
BmMITE-2	nscaf2176	1160406	1160132	-
BmMITE-2	nscaf2176	1000498	1000221	-

BmMITE-2	nscaf2176	876040	875762	-
BmMITE-2	nscaf2176	659910	659633	-
BmMITE-2	nscaf2176	210996	210719	-
BmMITE-2	nscaf2136	8274834	8274557	-
BmMITE-2	nscaf2136	7719365	7719088	-
BmMITE-2	nscaf2136	7641779	7641503	-
BmMITE-2	nscaf2136	7126663	7126386	-
BmMITE-2	nscaf2136	7084351	7084075	-
BmMITE-2	nscaf2136	5377718	5377441	-
BmMITE-2	nscaf2136	5182013	5181734	-
BmMITE-2	nscaf2136	5096906	5096629	-
BmMITE-2	nscaf2136	4982382	4982105	-
BmMITE-2	nscaf2136	4158046	4157769	-
BmMITE-2	nscaf2136	4111808	4111531	-
BmMITE-2	nscaf2136	3085631	3085354	-
BmMITE-2	nscaf2136	2896594	2896317	-
BmMITE-2	nscaf2136	2585246	2584969	-
BmMITE-2	nscaf2136	2248071	2247794	-
BmMITE-2	nscaf2136	1571541	1571264	-
BmMITE-2	nscaf2136	1267254	1266977	-
BmMITE-2	nscaf1898	15206335	15206058	-
BmMITE-2	nscaf1898	14981203	14980926	-
BmMITE-2	nscaf1898	14860940	14860666	-
BmMITE-2	nscaf1898	14738447	14738170	-
BmMITE-2	nscaf1898	13429078	13428801	-
BmMITE-2	nscaf1898	13327382	13327105	-
BmMITE-2	nscaf1898	13197965	13197687	-
BmMITE-2	nscaf1898	12832550	12832273	-
BmMITE-2	nscaf1898	12809095	12808818	-
BmMITE-2	nscaf1898	12436605	12436328	-
BmMITE-2	nscaf1898	12363716	12363439	-
BmMITE-2	nscaf1898	11132235	11131959	-
BmMITE-2	nscaf1898	10907563	10907286	-
BmMITE-2	nscaf1898	10869148	10868871	-
BmMITE-2	nscaf1898	10485894	10485618	-
BmMITE-2	nscaf1898	10245247	10244970	-
BmMITE-2	nscaf1898	10212549	10212273	-
BmMITE-2	nscaf1898	10196973	10196696	-
BmMITE-2	nscaf1898	10114042	10113765	-
BmMITE-2	nscaf1898	9039818	9039541	-
BmMITE-2	nscaf1898	8527317	8527043	-
BmMITE-2	nscaf1898	7811720	7811443	-
BmMITE-2	nscaf1898	7680867	7680590	-

BmMITE-2	nscaf1898	7643795	7643518	-
BmMITE-2	nscaf1898	7549285	7549008	-
BmMITE-2	nscaf1898	7221456	7221179	-
BmMITE-2	nscaf1898	7195065	7194788	-
BmMITE-2	nscaf1898	6935880	6935603	-
BmMITE-2	nscaf1898	5578714	5578437	-
BmMITE-2	nscaf1898	5388037	5387760	-
BmMITE-2	nscaf1898	5319873	5319586	-
BmMITE-2	nscaf1898	4936304	4936029	-
BmMITE-2	nscaf1898	4685200	4684923	-
BmMITE-2	nscaf1898	4598419	4598142	-
BmMITE-2	nscaf1898	4420649	4420372	-
BmMITE-2	nscaf1898	3915420	3915143	-
BmMITE-2	nscaf1898	2380875	2380598	-
BmMITE-2	nscaf1898	2193548	2193271	-
BmMITE-2	nscaf1690	7320556	7320280	-
BmMITE-2	nscaf1690	6304783	6304508	-
BmMITE-2	nscaf1690	4634720	4634443	-
BmMITE-2	nscaf1690	4003229	4002952	-
BmMITE-2	nscaf1690	3203523	3203246	-
BmMITE-2	nscaf1690	2969825	2969548	-
BmMITE-2	nscaf1690	2932993	2932716	-
BmMITE-2	nscaf1690	2889916	2889635	-
BmMITE-2	nscaf1690	2850927	2850650	-
BmMITE-2	nscaf1690	2068266	2067989	-
BmMITE-2	nscaf1690	1659992	1659715	-
BmMITE-2	nscaf1690	1378842	1378565	-
BmMITE-2	nscaf1690	1306303	1306026	-
BmMITE-2	nscaf1690	656329	656052	-
BmMITE-2	nscaf1690	527210	526933	-
BmMITE-2	nscaf1690	497346	497069	-
BmMITE-2	nscaf1690	443504	443227	-
BmMITE-2	nscaf1690	66495	66218	-
BmMITE-2	nscaf1681	5705773	5705496	-
BmMITE-2	nscaf1681	5640898	5640622	-
BmMITE-2	nscaf1681	5289375	5289103	-
BmMITE-2	nscaf1681	5008102	5007826	-
BmMITE-2	nscaf1681	4842506	4842229	-
BmMITE-2	nscaf1681	4727090	4726813	-
BmMITE-2	nscaf1681	4685293	4685016	-
BmMITE-2	nscaf1681	4166564	4166287	-
BmMITE-2	nscaf1681	3596729	3596452	-
BmMITE-2	nscaf1681	3513604	3513327	-

BmMITE-2	nscaf1681	2386821	2386544	-
BmMITE-2	nscaf1681	1161374	1161095	-
BmMITE-2	nscaf1681	1145394	1145117	-
BmMITE-2	nscaf1681	346055	345778	-
BmMITE-2	nscaf3034	3966205	3965929	-
BmMITE-2	nscaf3034	3820794	3820517	-
BmMITE-2	nscaf3034	3402380	3402103	-
BmMITE-2	nscaf3034	3060479	3060202	-
BmMITE-2	nscaf3034	2632761	2632484	-
BmMITE-2	nscaf3034	2022696	2022419	-
BmMITE-2	nscaf3034	573140	572864	-
BmMITE-2	nscaf3034	185598	185322	-
BmMITE-2	nscaf2948	2486418	2486141	-
BmMITE-2	nscaf2948	1689258	1688979	-
BmMITE-2	nscaf2948	1492800	1492523	-
BmMITE-2	nscaf2948	1474112	1473826	-
BmMITE-2	nscaf2948	1422905	1422628	-
BmMITE-2	nscaf2948	1228950	1228673	-
BmMITE-2	nscaf2948	627755	627479	-
BmMITE-2	scaffold7300	675	398	-
BmMITE-2	scaffold1303	6367	6090	-
BmMITE-2	scaffold1234	1052	775	-
BmMITE-2	scaffold1044	4907	4630	-
BmMITE-2	nscaf98	941507	941230	-
BmMITE-2	nscaf98	852419	852142	-
BmMITE-2	nscaf98	193174	192897	-
BmMITE-2	nscaf463	1519439	1519162	-
BmMITE-2	nscaf463	1486996	1486719	-
BmMITE-2	nscaf463	1303431	1303154	-
BmMITE-2	nscaf463	49666	49389	-
BmMITE-2	nscaf3075	843273	842997	-
BmMITE-2	nscaf3075	549276	548999	-
BmMITE-2	nscaf3075	504568	504291	-
BmMITE-2	nscaf3075	84474	84194	-
BmMITE-2	nscaf3072	2810353	2810076	-
BmMITE-2	nscaf3072	1592198	1591921	-
BmMITE-2	nscaf3072	881714	881437	-
BmMITE-2	nscaf3072	865109	864833	-
BmMITE-2	nscaf3072	755047	754770	-
BmMITE-2	nscaf3072	592106	591834	-
BmMITE-2	nscaf3072	429934	429652	-
BmMITE-2	nscaf3064	81127	80850	-
BmMITE-2	nscaf3063	3267996	3267719	-

BmMITE-2	nscaf3063	2711552	2711275	-
BmMITE-2	nscaf3063	1781770	1781493	-
BmMITE-2	nscaf3063	1686516	1686239	-
BmMITE-2	nscaf3063	1477554	1477277	-
BmMITE-2	nscaf3063	1232847	1232570	-
BmMITE-2	nscaf3063	982034	981756	-
BmMITE-2	nscaf3063	803520	803243	-
BmMITE-2	nscaf3063	793124	792846	-
BmMITE-2	nscaf3055	1467322	1467045	-
BmMITE-2	nscaf3055	681279	681001	-
BmMITE-2	nscaf3055	37309	37033	-
BmMITE-2	nscaf3041	1644867	1644590	-
BmMITE-2	nscaf3041	1348848	1348570	-
BmMITE-2	nscaf3040	3033075	3032798	-
BmMITE-2	nscaf3040	2032420	2032143	-
BmMITE-2	nscaf3040	1968486	1968210	-
BmMITE-2	nscaf3040	1628916	1628639	-
BmMITE-2	nscaf3040	1440452	1440175	-
BmMITE-2	nscaf3040	896416	896139	-
BmMITE-2	nscaf3040	510884	510607	-
BmMITE-2	nscaf3040	209987	209710	-
BmMITE-2	nscaf3040	145620	145343	-
BmMITE-2	nscaf3027	3571489	3571213	-
BmMITE-2	nscaf3027	2932272	2931995	-
BmMITE-2	nscaf3027	2189387	2189110	-
BmMITE-2	nscaf3027	1756877	1756600	-
BmMITE-2	nscaf3027	1481206	1480929	-
BmMITE-2	nscaf3027	1461460	1461183	-
BmMITE-2	nscaf3027	751386	751109	-
BmMITE-2	nscaf3027	131176	130899	-
BmMITE-2	nscaf3027	84623	84346	-
BmMITE-2	nscaf3015	3989324	3989047	-
BmMITE-2	nscaf3015	1362004	1361727	-
BmMITE-2	nscaf3015	1221906	1221629	-
BmMITE-2	nscaf3015	695674	695398	-
BmMITE-2	nscaf3015	224157	223880	-
BmMITE-2	nscaf3008	2093422	2093145	-
BmMITE-2	nscaf3008	1516639	1516362	-
BmMITE-2	nscaf3008	1357033	1356756	-
BmMITE-2	nscaf3008	1223674	1223397	-
BmMITE-2	nscaf3008	1077550	1077273	-
BmMITE-2	nscaf3008	789250	788973	-
BmMITE-2	nscaf2993	7560559	7560282	-

BmMITE-2	nscaf2993	7548303	7548026	-
BmMITE-2	nscaf2993	7430603	7430327	-
BmMITE-2	nscaf2993	7254486	7254209	-
BmMITE-2	nscaf2993	6774659	6774382	-
BmMITE-2	nscaf2993	6684783	6684506	-
BmMITE-2	nscaf2993	6004744	6004467	-
BmMITE-2	nscaf2993	5915284	5915007	-
BmMITE-2	nscaf2993	5856494	5856217	-
BmMITE-2	nscaf2993	5840996	5840719	-
BmMITE-2	nscaf2993	5732139	5731862	-
BmMITE-2	nscaf2993	5248743	5248473	-
BmMITE-2	nscaf2993	5202985	5202708	-
BmMITE-2	nscaf2993	4836931	4836652	-
BmMITE-2	nscaf2993	4708290	4708013	-
BmMITE-2	nscaf2993	3816047	3815770	-
BmMITE-2	nscaf2993	2393889	2393613	-
BmMITE-2	nscaf2993	1750105	1749827	-
BmMITE-2	nscaf2993	1135418	1135144	-
BmMITE-2	nscaf2993	425547	425270	-
BmMITE-2	nscaf2993	399458	399181	-
BmMITE-2	nscaf2970	2295251	2294974	-
BmMITE-2	nscaf2970	1838562	1838285	-
BmMITE-2	nscaf2970	1054421	1054144	-
BmMITE-2	nscaf2970	943394	943118	-
BmMITE-2	nscaf2970	878603	878326	-
BmMITE-2	nscaf2970	839541	839264	-
BmMITE-2	nscaf2970	693891	693615	-
BmMITE-2	nscaf2970	515962	515686	-
BmMITE-2	nscaf2970	162439	162162	-
BmMITE-2	nscaf2890	1178515	1178238	-
BmMITE-2	nscaf2890	837650	837373	-
BmMITE-2	nscaf2890	287169	286892	-
BmMITE-2	nscaf2883	2329157	2328880	-
BmMITE-2	nscaf2883	2267373	2267093	-
BmMITE-2	nscaf2883	2197380	2197103	-
BmMITE-2	nscaf2883	1775465	1775188	-
BmMITE-2	nscaf2883	1746447	1746170	-
BmMITE-2	nscaf2883	1323235	1322960	-
BmMITE-2	nscaf2883	1082253	1081976	-
BmMITE-2	nscaf2883	1011035	1010758	-
BmMITE-2	nscaf2883	597707	597430	-
BmMITE-2	nscaf2874	522632	522356	-
BmMITE-2	nscaf2852	2628108	2627831	-

BmMITE-2	nscaf2852	1615934	1615657	-
BmMITE-2	nscaf2852	1354075	1353805	-
BmMITE-2	nscaf2852	1337144	1336867	-
BmMITE-2	nscaf2852	903131	902854	-
BmMITE-2	nscaf2852	645103	644826	-
BmMITE-2	nscaf2852	380331	380054	-
BmMITE-2	nscaf2848	14498	14221	-
BmMITE-2	nscaf2833	476073	475796	-
BmMITE-2	nscaf2833	153206	152929	-
BmMITE-2	nscaf2833	83888	83611	-
BmMITE-2	nscaf2825	667189	666912	-
BmMITE-2	nscaf2825	326383	326107	-
BmMITE-2	nscaf2825	160836	160560	-
BmMITE-2	nscaf2825	80594	80317	-
BmMITE-2	nscaf2825	38227	37959	-
BmMITE-2	nscaf2815	1640142	1639865	-
BmMITE-2	nscaf2815	1629225	1628948	-
BmMITE-2	nscaf2815	1578592	1578316	-
BmMITE-2	nscaf2801	495328	495051	-
BmMITE-2	nscaf2801	462253	461982	-
BmMITE-2	nscaf2797	402222	401947	-
BmMITE-2	nscaf2681	616628	616351	-
BmMITE-2	nscaf2674	7010748	7010471	-
BmMITE-2	nscaf2674	6479800	6479523	-
BmMITE-2	nscaf2674	5787008	5786731	-
BmMITE-2	nscaf2674	5625453	5625176	-
BmMITE-2	nscaf2674	5308194	5307917	-
BmMITE-2	nscaf2674	5286931	5286649	-
BmMITE-2	nscaf2674	4517218	4516941	-
BmMITE-2	nscaf2674	4385982	4385705	-
BmMITE-2	nscaf2674	3909522	3909245	-
BmMITE-2	nscaf2674	3797605	3797328	-
BmMITE-2	nscaf2674	3722751	3722470	-
BmMITE-2	nscaf2674	3346451	3346176	-
BmMITE-2	nscaf2674	2779305	2779028	-
BmMITE-2	nscaf2674	2157059	2156781	-
BmMITE-2	nscaf2674	1153865	1153588	-
BmMITE-2	nscaf2674	1091671	1091394	-
BmMITE-2	nscaf2674	843727	843450	-
BmMITE-2	nscaf2674	752545	752268	-
BmMITE-2	nscaf2674	719337	719060	-
BmMITE-2	nscaf2674	202554	202277	-
BmMITE-2	nscaf2210	4470278	4470002	-

BmMITE-2	nscaf2210	4311693	4311416	-
BmMITE-2	nscaf2210	2826495	2826218	-
BmMITE-2	nscaf2210	2542170	2541893	-
BmMITE-2	nscaf2210	2052726	2052449	-
BmMITE-2	nscaf2210	1939994	1939719	-
BmMITE-2	nscaf2210	1670469	1670192	-
BmMITE-2	nscaf1274	3116	2839	-
BmMITE-2	nscaf2937	1394311	1394034	-
BmMITE-2	nscaf2937	1219519	1219242	-
BmMITE-2	nscaf2937	1019286	1019010	-
BmMITE-2	nscaf2937	306132	305855	-
BmMITE-2	nscaf3079	1059392	1059115	-
BmMITE-2	nscaf3079	996101	995824	-
BmMITE-2	nscaf3079	916539	916262	-
BmMITE-2	scaffold3516	282	5	-
BmMITE-2	scaffold22372	585	308	-
BmMITE-2	nscaf3090	196689	196406	-
BmMITE-2	nscaf3090	135262	134985	-
BmMITE-2	nscaf3090	111272	110995	-
BmMITE-2	nscaf3053	396023	395750	-
BmMITE-2	nscaf3053	96323	96046	-
BmMITE-2	nscaf3053	82416	82139	-
BmMITE-2	nscaf3044	1196948	1196671	-
BmMITE-2	nscaf3044	555794	555517	-
BmMITE-2	nscaf3044	241758	241481	-
BmMITE-2	nscaf3007	84350	84074	-
BmMITE-2	nscaf3005	1618986	1618709	-
BmMITE-2	nscaf3005	1490332	1490055	-
BmMITE-2	nscaf3005	1194982	1194706	-
BmMITE-2	nscaf3005	1089091	1088814	-
BmMITE-2	nscaf3005	893937	893660	-
BmMITE-2	nscaf2916	552439	552164	-
BmMITE-2	nscaf2916	435994	435717	-
BmMITE-2	nscaf2916	361963	361687	-
BmMITE-2	nscaf2916	150962	150686	-
BmMITE-2	nscaf2916	23795	23518	-
BmMITE-2	nscaf2900	199731	199454	-
BmMITE-2	nscaf2899	157819	157538	-
BmMITE-2	nscaf2891	2560764	2560487	-
BmMITE-2	nscaf2891	2249298	2249021	-
BmMITE-2	nscaf2891	1747666	1747389	-
BmMITE-2	nscaf2851	943918	943641	-
BmMITE-2	nscaf2851	867539	867262	-

BmMITE-2	nscaf2851	816220	815943	-
BmMITE-2	nscaf2851	619231	618954	-
BmMITE-2	nscaf2838	2625582	2625305	-
BmMITE-2	nscaf2838	2479693	2479416	-
BmMITE-2	nscaf2838	2239456	2239179	-
BmMITE-2	nscaf2838	824129	823852	-
BmMITE-2	nscaf2836	186172	185895	-
BmMITE-2	nscaf2836	158997	158720	-
BmMITE-2	nscaf2836	5503	5226	-
BmMITE-2	nscaf2734	1102612	1102335	-
BmMITE-2	nscaf2734	1087818	1087538	-
BmMITE-2	nscaf2686	1129968	1129691	-
BmMITE-2	nscaf2686	980649	980372	-
BmMITE-2	nscaf2686	870720	870443	-
BmMITE-2	nscaf2686	756903	756626	-
BmMITE-2	nscaf2686	718817	718540	-
BmMITE-2	nscaf2686	555559	555281	-
BmMITE-2	nscaf2686	526797	526520	-
BmMITE-2	nscaf2686	238984	238707	-
BmMITE-2	nscaf2686	211754	211477	-
BmMITE-2	nscaf2686	57301	57025	-
BmMITE-2	nscaf2686	15606	15329	-
BmMITE-2	nscaf2564	328833	328556	-
BmMITE-2	nscaf2216	1571693	1571416	-
BmMITE-2	nscaf2216	289999	289723	-
BmMITE-2	nscaf2216	69967	69690	-
BmMITE-2	scaffold584	95269	94992	-
BmMITE-2	scaffold695	4629	4352	-
BmMITE-2	scaffold2903	1226	950	-
BmMITE-2	scaffold1771	601	324	-
BmMITE-2	nscaf797	582	305	-
BmMITE-2	nscaf3066	1687869	1687593	-
BmMITE-2	nscaf3066	1280516	1280240	-
BmMITE-2	nscaf3066	636397	636119	-
BmMITE-2	nscaf3066	95038	94761	-
BmMITE-2	nscaf3052	871442	871166	-
BmMITE-2	nscaf3052	763297	763020	-
BmMITE-2	nscaf3052	636547	636270	-
BmMITE-2	nscaf3052	411113	410836	-
BmMITE-2	nscaf2971	284755	284478	-
BmMITE-2	nscaf2952	87498	87216	-
BmMITE-2	nscaf2952	75647	75370	-
BmMITE-2	nscaf2952	14432	14155	-

BmMITE-2	nscaf2912	1301335	1301057	-
BmMITE-2	nscaf2912	1242336	1242059	-
BmMITE-2	nscaf2889	3388657	3388380	-
BmMITE-2	nscaf2889	2939246	2938969	-
BmMITE-2	nscaf2889	2122564	2122288	-
BmMITE-2	nscaf2889	867053	866776	-
BmMITE-2	nscaf2889	415868	415591	-
BmMITE-2	nscaf2819	110965	110690	-
BmMITE-2	nscaf2814	402353	402077	-
BmMITE-2	nscaf2814	48280	48003	-
BmMITE-2	nscaf125	247286	247010	-
BmMITE-2	nscaf125	120831	120554	-
BmMITE-2	nscaf125	6327	6050	-
BmMITE-2	scaffold2708	1695	1419	-
BmMITE-2	nscaf2810	439215	438943	-
BmMITE-2	nscaf2810	129293	129017	-
BmMITE-2	nscaf2793	81180	80903	-
BmMITE-2	nscaf2793	66514	66235	-
BmMITE-2	nscaf201	19225	18945	-
BmMITE-2	nscaf1705	1221855	1221577	-
BmMITE-2	nscaf1705	1185288	1185011	-
BmMITE-2	nscaf1705	1122027	1121750	-
BmMITE-2	nscaf1705	872689	872412	-
BmMITE-2	nscaf1705	738449	738172	-
BmMITE-2	nscaf1705	537824	537549	-
BmMITE-2	scaffold983	2192	1915	-
BmMITE-2	scaffold974	7703	7426	-
BmMITE-2	scaffold765	16612	16335	-
BmMITE-2	scaffold752	28381	28104	-
BmMITE-2	scaffold34892	555	278	-
BmMITE-2	nscaf3118	2903	2627	-
BmMITE-2	nscaf3084	15465	15188	-
BmMITE-2	nscaf3002	290719	290442	-
BmMITE-2	nscaf3002	190995	190718	-
BmMITE-2	nscaf2990	471045	470768	-
BmMITE-2	nscaf2990	262383	262106	-
BmMITE-2	nscaf2990	106139	105862	-
BmMITE-2	nscaf2962	1156326	1156049	-
BmMITE-2	nscaf2962	961319	961042	-
BmMITE-2	nscaf2962	164707	164430	-
BmMITE-2	nscaf2959	309848	309571	-
BmMITE-2	nscaf2959	289362	289085	-
BmMITE-2	nscaf2959	275196	274919	-

BmMITE-2	nscaf2959	230124	229847	-
BmMITE-2	nscaf2924	800366	800089	-
BmMITE-2	nscaf2924	318735	318458	-
BmMITE-2	nscaf2844	140892	140615	-
BmMITE-2	nscaf2844	123036	122759	-
BmMITE-2	nscaf2813	51537	51260	-
BmMITE-2	nscaf2780	163579	163302	-
BmMITE-2	nscaf2780	109023	108746	-
BmMITE-2	nscaf2766	904761	904484	-
BmMITE-2	nscaf1962	526108	525831	-
BmMITE-2	nscaf120	32169	31892	-
BmMITE-2	nscaf120	6591	6314	-
BmMITE-2	nscaf2910	543773	543497	-
BmMITE-2	nscaf2910	91821	91543	-
BmMITE-2	nscaf2910	79521	79238	-
BmMITE-2	scaffold816	10618	10341	-
BmMITE-2	scaffold6133	811	534	-
BmMITE-2	scaffold12343	408	131	-
BmMITE-2	nscaf3104	308037	307760	-
BmMITE-2	nscaf3083	6146	5869	-
BmMITE-2	nscaf3078	665761	665485	-
BmMITE-2	nscaf3038	145530	145253	-
BmMITE-2	nscaf3038	42615	42338	-
BmMITE-2	nscaf2893	6791	6514	-
BmMITE-2	scaffold3238	343	67	-
BmMITE-2	nscaf3031	1198387	1198098	-
BmMITE-2	nscaf2924	26574	26265	-
BmMITE-2	nscaf2529	1629406	1629122	-
BmMITE-2	nscaf2827	323016	322737	-
BmMITE-2	nscaf481	624708	623962	-
BmMITE-2	nscaf3063	1570997	1570729	-
BmMITE-2	nscaf2847	2596367	2596089	-
BmMITE-2	nscaf2795	1805920	1805654	-
BmMITE-2	nscaf2795	1243922	1243644	-
BmMITE-2	nscaf2176	2116148	2115870	-
BmMITE-2	nscaf1681	3793243	3792965	-
BmMITE-2	nscaf463	861506	860865	-
BmMITE-2	nscaf2686	93370	93063	-
BmMITE-2	nscaf2360	14969	14707	-
BmMITE-2	nscaf3037	51904	51617	-
BmMITE-2	nscaf2868	210076	209364	-
BmMITE-2	nscaf2176	2475146	2474870	-
BmMITE-2	nscaf3075	732040	731765	-

BmMITE-2	nscaf2970	2076263	2075983	-
BmMITE-2	nscaf2953	639612	639343	-
BmMITE-2	nscaf2902	9583129	9582854	-
BmMITE-2	nscaf2823	3838366	3838088	-
BmMITE-2	nscaf2822	195263	194985	-
BmMITE-2	nscaf2655	1259148	1258871	-
BmMITE-2	nscaf2674	7418918	7418641	-
BmMITE-2	nscaf3097	2557961	2557687	-
BmMITE-2	nscaf2964	3201612	3201337	-
BmMITE-2	nscaf2136	4778452	4778186	-
BmMITE-2	nscaf1898	6753800	6753537	-
BmMITE-2	nscaf2825	227100	226837	-
BmMITE-2	nscaf2681	742778	742500	-
BmMITE-2	nscaf3005	1388885	1388610	-
BmMITE-2	nscaf3060	65890	65621	-
BmMITE-2	nscaf2330	3954520	3954244	-
BmMITE-2	nscaf3040	1934873	1934596	-
BmMITE-2	nscaf3038	160360	160084	-
BmMITE-2	nscaf2767	281806	281527	-
BmMITE-2	nscaf2983	1750244	1749977	-
BmMITE-2	nscaf2868	762134	761876	-
BmMITE-2	nscaf2901	1269214	1268959	-
BmMITE-2	nscaf2529	5403460	5403201	-
BmMITE-2	nscaf2951	763391	763131	-
BmMITE-2	nscaf2674	2043525	2043272	-
BmMITE-2	nscaf2210	1959419	1959171	-
BmMITE-2	nscaf2983	3184174	3183922	-
BmMITE-2	nscaf2964	4829158	4828913	-
BmMITE-2	nscaf3015	1452536	1452279	-
BmMITE-2	nscaf3072	2735064	2734822	-
BmMITE-2	nscaf2796	91620	91380	-
BmMITE-2	nscaf2891	2747445	2747184	-
BmMITE-2	nscaf2998	188156	187907	-
BmMITE-2	nscaf2575	3418258	3418039	-
BmMITE-2	nscaf2883	2479298	2479064	-
BmMITE-2	nscaf2759	314588	314865	+
BmMITE-2	scaffold33133	298	575	+
BmMITE-2	nscaf3098	1266633	1266910	+
BmMITE-2	nscaf3098	1448468	1448746	+
BmMITE-2	nscaf3098	1601684	1601961	+
BmMITE-2	nscaf3098	1949137	1949414	+
BmMITE-2	nscaf3098	2077738	2078015	+
BmMITE-2	nscaf3045	78704	78981	+

BmMITE-2	nscaf3045	928519	928796	+
BmMITE-2	nscaf3045	1161165	1161441	+
BmMITE-2	nscaf3045	1481419	1481696	+
BmMITE-2	nscaf3045	1496497	1496774	+
BmMITE-2	nscaf3045	2312841	2313118	+
BmMITE-2	nscaf3033	317214	317491	+
BmMITE-2	nscaf3003	604105	604383	+
BmMITE-2	nscaf3003	681927	682204	+
BmMITE-2	nscaf3003	985261	985538	+
BmMITE-2	nscaf3003	1433746	1434023	+
BmMITE-2	nscaf3003	1450251	1450528	+
BmMITE-2	nscaf3003	2208025	2208302	+
BmMITE-2	nscaf3003	2238689	2238966	+
BmMITE-2	nscaf3003	2700626	2700904	+
BmMITE-2	nscaf3003	2867397	2867675	+
BmMITE-2	nscaf3003	3271051	3271328	+
BmMITE-2	nscaf3003	3284315	3284592	+
BmMITE-2	nscaf3003	3335612	3335889	+
BmMITE-2	nscaf3003	3368033	3368309	+
BmMITE-2	nscaf3003	3765068	3765345	+
BmMITE-2	nscaf3003	3874077	3874354	+
BmMITE-2	nscaf3003	3983287	3983575	+
BmMITE-2	nscaf3003	4049367	4049644	+
BmMITE-2	nscaf3003	4061409	4061686	+
BmMITE-2	nscaf3003	4232244	4232521	+
BmMITE-2	nscaf3003	4438660	4438937	+
BmMITE-2	nscaf2986	49314	49591	+
BmMITE-2	nscaf2986	574793	575070	+
BmMITE-2	nscaf2986	888490	888767	+
BmMITE-2	nscaf2986	1123218	1123495	+
BmMITE-2	nscaf2986	1707150	1707427	+
BmMITE-2	nscaf2986	1883380	1883657	+
BmMITE-2	nscaf2986	1976463	1976739	+
BmMITE-2	nscaf2986	2012187	2012464	+
BmMITE-2	nscaf2986	2259662	2259938	+
BmMITE-2	nscaf2986	2354180	2354457	+
BmMITE-2	nscaf2986	2445228	2445505	+
BmMITE-2	nscaf2986	2548483	2548760	+
BmMITE-2	nscaf2986	2804881	2805158	+
BmMITE-2	nscaf2986	3549996	3550266	+
BmMITE-2	nscaf2986	3786319	3786596	+
BmMITE-2	nscaf2986	4050746	4051019	+
BmMITE-2	nscaf2986	4390924	4391200	+

BmMITE-2	nscaf2986	4698011	4698288	+
BmMITE-2	nscaf2986	4842275	4842552	+
BmMITE-2	nscaf2986	5083666	5083942	+
BmMITE-2	nscaf2986	5227791	5228068	+
BmMITE-2	nscaf2986	5326805	5327082	+
BmMITE-2	nscaf2986	6298338	6298614	+
BmMITE-2	nscaf2931	290423	290700	+
BmMITE-2	nscaf2931	888649	888926	+
BmMITE-2	nscaf2931	1510451	1510735	+
BmMITE-2	nscaf2931	1945776	1946053	+
BmMITE-2	nscaf2931	2065313	2065590	+
BmMITE-2	nscaf2931	2129164	2129441	+
BmMITE-2	nscaf2930	70108	70385	+
BmMITE-2	nscaf2930	492852	493129	+
BmMITE-2	nscaf2930	788316	788593	+
BmMITE-2	nscaf2930	1067117	1067394	+
BmMITE-2	nscaf2930	1227495	1227772	+
BmMITE-2	nscaf2930	1949886	1950163	+
BmMITE-2	nscaf2930	2319129	2319406	+
BmMITE-2	nscaf2930	3056764	3057041	+
BmMITE-2	nscaf2930	3318867	3319144	+
BmMITE-2	nscaf2930	3724907	3725183	+
BmMITE-2	nscaf2930	4035296	4035573	+
BmMITE-2	nscaf2930	4830284	4830560	+
BmMITE-2	nscaf2930	5123085	5123362	+
BmMITE-2	nscaf2930	6269809	6270086	+
BmMITE-2	nscaf2887	228620	228897	+
BmMITE-2	nscaf2887	251526	251803	+
BmMITE-2	nscaf2887	314860	315137	+
BmMITE-2	nscaf2887	341302	341579	+
BmMITE-2	nscaf2887	1032691	1032968	+
BmMITE-2	nscaf2887	1057542	1057819	+
BmMITE-2	nscaf2887	1281822	1282099	+
BmMITE-2	nscaf2887	1377237	1377516	+
BmMITE-2	nscaf2887	1733936	1734213	+
BmMITE-2	nscaf2886	466598	466875	+
BmMITE-2	nscaf2886	799707	799984	+
BmMITE-2	nscaf2886	939404	939681	+
BmMITE-2	nscaf2886	1046640	1046917	+
BmMITE-2	nscaf2886	1061398	1061675	+
BmMITE-2	nscaf2886	1167309	1167586	+
BmMITE-2	nscaf2847	1218097	1218374	+
BmMITE-2	nscaf2847	3240707	3240984	+

BmMITE-2	nscaf2847	3521470	3521750	+
BmMITE-2	nscaf2847	3975005	3975282	+
BmMITE-2	nscaf2847	5045445	5045722	+
BmMITE-2	nscaf2847	5460989	5461266	+
BmMITE-2	nscaf2847	5524351	5524628	+
BmMITE-2	nscaf2847	5741686	5741963	+
BmMITE-2	nscaf2847	6682287	6682564	+
BmMITE-2	nscaf2847	7594769	7595046	+
BmMITE-2	nscaf2847	7944460	7944738	+
BmMITE-2	nscaf2847	8236497	8236774	+
BmMITE-2	nscaf2827	634411	634688	+
BmMITE-2	nscaf2827	746210	746487	+
BmMITE-2	nscaf2827	825430	825707	+
BmMITE-2	nscaf2826	43923	44200	+
BmMITE-2	nscaf2826	159946	160223	+
BmMITE-2	nscaf2826	469695	469974	+
BmMITE-2	nscaf2818	149107	149384	+
BmMITE-2	nscaf2818	755181	755458	+
BmMITE-2	nscaf2818	815258	815664	+
BmMITE-2	nscaf2818	1269069	1269346	+
BmMITE-2	nscaf2818	1327345	1327621	+
BmMITE-2	nscaf2818	1417155	1417432	+
BmMITE-2	nscaf2818	1588873	1589150	+
BmMITE-2	nscaf2818	1712139	1712416	+
BmMITE-2	nscaf2818	2346351	2346628	+
BmMITE-2	nscaf2818	2401393	2401670	+
BmMITE-2	nscaf2818	2960809	2961086	+
BmMITE-2	nscaf2781	95077	95354	+
BmMITE-2	nscaf2767	373815	374385	+
BmMITE-2	nscaf2767	805425	805702	+
BmMITE-2	nscaf2767	1399154	1399431	+
BmMITE-2	nscaf2767	1435979	1436256	+
BmMITE-2	nscaf2767	1755954	1756235	+
BmMITE-2	nscaf2767	1807599	1807875	+
BmMITE-2	nscaf2767	1831873	1832150	+
BmMITE-2	nscaf2767	2085849	2086125	+
BmMITE-2	nscaf2767	2622246	2622522	+
BmMITE-2	nscaf2767	3743119	3743395	+
BmMITE-2	nscaf2767	3795377	3795654	+
BmMITE-2	nscaf2767	3906112	3906389	+
BmMITE-2	nscaf2767	4043251	4043528	+
BmMITE-2	nscaf2767	4164629	4164906	+
BmMITE-2	nscaf2623	341429	341706	+

BmMITE-2	nscaf2623	353244	353521	+
BmMITE-2	nscaf2623	418073	418350	+
BmMITE-2	nscaf2623	469403	469680	+
BmMITE-2	nscaf2623	490507	490784	+
BmMITE-2	nscaf2623	573446	573723	+
BmMITE-2	nscaf2623	700593	700870	+
BmMITE-2	nscaf2623	718962	719239	+
BmMITE-2	nscaf2623	1065686	1065963	+
BmMITE-2	nscaf2623	1122020	1122297	+
BmMITE-2	nscaf2623	1216476	1216754	+
BmMITE-2	nscaf2623	1278161	1278438	+
BmMITE-2	nscaf2623	1409399	1409676	+
BmMITE-2	nscaf2623	1423291	1423568	+
BmMITE-2	nscaf2623	1599480	1599757	+
BmMITE-2	nscaf2623	1730466	1730744	+
BmMITE-2	nscaf2623	1838221	1838498	+
BmMITE-2	nscaf2529	147230	147507	+
BmMITE-2	nscaf2529	287545	287823	+
BmMITE-2	nscaf2529	479907	480184	+
BmMITE-2	nscaf2529	1588817	1589094	+
BmMITE-2	nscaf2529	2476993	2477270	+
BmMITE-2	nscaf2529	2697288	2697565	+
BmMITE-2	nscaf2529	2741235	2741512	+
BmMITE-2	nscaf2529	2943670	2943947	+
BmMITE-2	nscaf2529	2955870	2956147	+
BmMITE-2	nscaf2529	3127293	3127570	+
BmMITE-2	nscaf2529	4262935	4263212	+
BmMITE-2	nscaf2529	4440255	4440532	+
BmMITE-2	nscaf2529	4508173	4508450	+
BmMITE-2	nscaf2529	4721756	4722033	+
BmMITE-2	nscaf2529	4921300	4921577	+
BmMITE-2	nscaf2529	5160100	5160377	+
BmMITE-2	nscaf2529	5583360	5583637	+
BmMITE-2	nscaf2330	148090	148367	+
BmMITE-2	nscaf2330	582132	582409	+
BmMITE-2	nscaf2330	1437464	1437741	+
BmMITE-2	nscaf2330	1494240	1494517	+
BmMITE-2	nscaf2330	1561805	1562082	+
BmMITE-2	nscaf2330	1783255	1783529	+
BmMITE-2	nscaf2330	2068410	2068687	+
BmMITE-2	nscaf2330	2338072	2338349	+
BmMITE-2	nscaf2330	2556562	2556839	+
BmMITE-2	nscaf2330	3434199	3434476	+

BmMITE-2	nscaf2330	4080355	4080632	+
BmMITE-2	nscaf2330	4157685	4157962	+
BmMITE-2	nscaf2330	4253614	4253891	+
BmMITE-2	nscaf2053	289391	289668	+
BmMITE-2	nscaf1108	140183	140460	+
BmMITE-2	nscaf1108	256051	256328	+
BmMITE-2	nscaf1108	326311	326588	+
BmMITE-2	nscaf1108	535685	535962	+
BmMITE-2	nscaf1108	1805017	1805294	+
BmMITE-2	nscaf1108	1906549	1906826	+
BmMITE-2	nscaf1108	1930654	1930933	+
BmMITE-2	nscaf1108	2307784	2308061	+
BmMITE-2	nscaf1108	2321309	2321586	+
BmMITE-2	nscaf1108	2439138	2439415	+
BmMITE-2	nscaf1108	2664822	2665098	+
BmMITE-2	nscaf1108	2815537	2815814	+
BmMITE-2	nscaf1108	2871920	2872197	+
BmMITE-2	nscaf1108	2890365	2890642	+
BmMITE-2	nscaf1071	53613	53890	+
BmMITE-2	nscaf1071	743758	744035	+
BmMITE-2	nscaf1071	973036	973313	+
BmMITE-2	nscaf1071	1066306	1066583	+
BmMITE-2	nscaf2888	327090	327367	+
BmMITE-2	nscaf2888	494692	494969	+
BmMITE-2	nscaf2888	1147773	1148050	+
BmMITE-2	nscaf2888	1304278	1304555	+
BmMITE-2	nscaf2888	1708055	1708332	+
BmMITE-2	nscaf2888	2730681	2730958	+
BmMITE-2	nscaf2888	3062984	3063258	+
BmMITE-2	nscaf2888	3722367	3722641	+
BmMITE-2	nscaf2888	3745418	3745695	+
BmMITE-2	nscaf2888	3999184	3999461	+
BmMITE-2	nscaf2888	4149906	4150183	+
BmMITE-2	nscaf2888	4541187	4541464	+
BmMITE-2	nscaf2888	4601329	4601606	+
BmMITE-2	nscaf2888	4881770	4882047	+
BmMITE-2	nscaf2888	7271368	7271644	+
BmMITE-2	nscaf2888	7366985	7367262	+
BmMITE-2	nscaf2888	7410210	7410483	+
BmMITE-2	nscaf2888	8417925	8418202	+
BmMITE-2	nscaf2888	9491895	9492172	+
BmMITE-2	nscaf2865	471122	471399	+
BmMITE-2	nscaf2865	852159	852435	+

BmMITE-2	nscaf2865	995330	995607	+
BmMITE-2	nscaf2865	1439312	1439589	+
BmMITE-2	nscaf2865	2005190	2005467	+
BmMITE-2	nscaf2865	2059062	2059339	+
BmMITE-2	nscaf2865	2130059	2130336	+
BmMITE-2	nscaf2865	2538338	2538699	+
BmMITE-2	nscaf2865	2681972	2682248	+
BmMITE-2	nscaf2865	2805930	2806207	+
BmMITE-2	nscaf2865	2868736	2869013	+
BmMITE-2	nscaf2865	3429847	3430124	+
BmMITE-2	nscaf2865	3635372	3635649	+
BmMITE-2	nscaf2865	3661896	3662173	+
BmMITE-2	nscaf2865	4298088	4298365	+
BmMITE-2	nscaf2865	4654403	4654680	+
BmMITE-2	nscaf2865	4682487	4682764	+
BmMITE-2	nscaf2865	4883296	4883573	+
BmMITE-2	nscaf2865	5286374	5286644	+
BmMITE-2	nscaf2575	422955	423232	+
BmMITE-2	nscaf2575	677367	677644	+
BmMITE-2	nscaf2575	1534589	1534866	+
BmMITE-2	nscaf2575	1643019	1643294	+
BmMITE-2	nscaf2575	1976014	1976291	+
BmMITE-2	nscaf2575	2039751	2040028	+
BmMITE-2	nscaf2575	2116892	2117169	+
BmMITE-2	nscaf2575	2355313	2355590	+
BmMITE-2	nscaf2575	2510354	2510631	+
BmMITE-2	nscaf2575	2798017	2798293	+
BmMITE-2	nscaf2575	2849767	2850044	+
BmMITE-2	nscaf2575	3187769	3188050	+
BmMITE-2	nscaf2575	3507336	3507613	+
BmMITE-2	nscaf2575	3543787	3544064	+
BmMITE-2	nscaf2575	3722189	3722466	+
BmMITE-2	nscaf2575	3757554	3757832	+
BmMITE-2	nscaf2575	3980213	3980489	+
BmMITE-2	nscaf2575	4458685	4458962	+
BmMITE-2	nscaf2953	75651	75928	+
BmMITE-2	nscaf2953	172231	172508	+
BmMITE-2	nscaf2953	680717	680994	+
BmMITE-2	nscaf2953	1055914	1056191	+
BmMITE-2	nscaf2953	1123925	1124204	+
BmMITE-2	nscaf2953	1337625	1337901	+
BmMITE-2	nscaf2953	2648684	2648961	+
BmMITE-2	nscaf2953	3241332	3241609	+

BmMITE-2	nscaf2953	3730697	3730974	+
BmMITE-2	scaffold878	15778	16055	+
BmMITE-2	scaffold26217	205	482	+
BmMITE-2	scaffold23836	218	495	+
BmMITE-2	scaffold16257	356	633	+
BmMITE-2	scaffold1110	6353	6630	+
BmMITE-2	nscaf481	165783	166060	+
BmMITE-2	nscaf481	178455	178732	+
BmMITE-2	nscaf481	304755	305032	+
BmMITE-2	nscaf481	447751	448028	+
BmMITE-2	nscaf481	501957	502234	+
BmMITE-2	nscaf481	1201189	1201466	+
BmMITE-2	nscaf3099	424795	425072	+
BmMITE-2	nscaf3099	511404	511681	+
BmMITE-2	nscaf3099	534050	534327	+
BmMITE-2	nscaf3099	688348	688625	+
BmMITE-2	nscaf3099	842151	842428	+
BmMITE-2	nscaf3099	1017641	1017918	+
BmMITE-2	nscaf3099	1472659	1472936	+
BmMITE-2	nscaf3099	1644006	1644282	+
BmMITE-2	nscaf3099	1668819	1669096	+
BmMITE-2	nscaf3099	2018804	2019081	+
BmMITE-2	nscaf3099	2066146	2066423	+
BmMITE-2	nscaf3099	2095230	2095508	+
BmMITE-2	nscaf3099	2145955	2146232	+
BmMITE-2	nscaf3099	2242566	2242843	+
BmMITE-2	nscaf3099	2433809	2434086	+
BmMITE-2	nscaf3099	2763342	2763619	+
BmMITE-2	nscaf3099	3007947	3008223	+
BmMITE-2	nscaf3099	3071700	3071977	+
BmMITE-2	nscaf3099	3208159	3208436	+
BmMITE-2	nscaf3099	3844033	3844310	+
BmMITE-2	nscaf3099	4019723	4019999	+
BmMITE-2	nscaf3099	4138456	4138733	+
BmMITE-2	nscaf3099	4480323	4480600	+
BmMITE-2	nscaf3097	7836	8117	+
BmMITE-2	nscaf3097	385026	385303	+
BmMITE-2	nscaf3097	785143	785420	+
BmMITE-2	nscaf3097	931489	931766	+
BmMITE-2	nscaf3097	2141475	2141749	+
BmMITE-2	nscaf3097	2266160	2266437	+
BmMITE-2	nscaf3097	2307522	2307799	+
BmMITE-2	nscaf3097	2537602	2537879	+

BmMITE-2	nscaf3089	20292	20569	+
BmMITE-2	nscaf3089	130077	130354	+
BmMITE-2	nscaf3089	195600	195877	+
BmMITE-2	nscaf3089	229065	229341	+
BmMITE-2	nscaf3089	385130	385407	+
BmMITE-2	nscaf3062	579978	580254	+
BmMITE-2	nscaf3058	42408	42682	+
BmMITE-2	nscaf3058	235273	235550	+
BmMITE-2	nscaf3058	287646	287923	+
BmMITE-2	nscaf3058	419100	419380	+
BmMITE-2	nscaf3058	629799	630076	+
BmMITE-2	nscaf3058	799674	799951	+
BmMITE-2	nscaf3058	1527719	1527996	+
BmMITE-2	nscaf3058	2582964	2583241	+
BmMITE-2	nscaf3058	2616727	2617007	+
BmMITE-2	nscaf3058	3224613	3224890	+
BmMITE-2	nscaf3058	3480962	3481239	+
BmMITE-2	nscaf3058	3633875	3634152	+
BmMITE-2	nscaf3058	3658567	3658844	+
BmMITE-2	nscaf3058	3781523	3781800	+
BmMITE-2	nscaf3058	4237988	4238266	+
BmMITE-2	nscaf3058	4442586	4442863	+
BmMITE-2	nscaf3058	4798594	4798871	+
BmMITE-2	nscaf3058	4826396	4826672	+
BmMITE-2	nscaf3058	4895493	4895770	+
BmMITE-2	nscaf3058	4932162	4932444	+
BmMITE-2	nscaf3058	5057593	5057870	+
BmMITE-2	nscaf3058	5243058	5243335	+
BmMITE-2	nscaf3058	5406745	5407022	+
BmMITE-2	nscaf3058	5618864	5619141	+
BmMITE-2	nscaf3058	5670040	5670316	+
BmMITE-2	nscaf3058	5737770	5738046	+
BmMITE-2	nscaf3058	5971814	5972090	+
BmMITE-2	nscaf3058	7018600	7018877	+
BmMITE-2	nscaf3058	7041440	7041717	+
BmMITE-2	nscaf3058	7219275	7219552	+
BmMITE-2	nscaf3058	7343834	7344111	+
BmMITE-2	nscaf3058	7549062	7549338	+
BmMITE-2	nscaf3058	8120935	8121211	+
BmMITE-2	nscaf3058	8216662	8216938	+
BmMITE-2	nscaf3058	8450719	8450996	+
BmMITE-2	nscaf3058	8514204	8514481	+
BmMITE-2	nscaf3058	8916916	8917193	+

BmMITE-2	nscaf3035	107501	107777	+
BmMITE-2	nscaf3035	123231	123508	+
BmMITE-2	nscaf3035	150081	150358	+
BmMITE-2	nscaf3035	518007	518284	+
BmMITE-2	nscaf3035	730099	730382	+
BmMITE-2	nscaf3035	794515	794792	+
BmMITE-2	nscaf3035	958455	958731	+
BmMITE-2	nscaf3035	1281124	1281401	+
BmMITE-2	nscaf3035	1321956	1322232	+
BmMITE-2	nscaf3035	1460343	1460620	+
BmMITE-2	nscaf3032	431505	431778	+
BmMITE-2	nscaf3032	594114	594390	+
BmMITE-2	nscaf3032	870697	870974	+
BmMITE-2	nscaf3032	1875695	1875972	+
BmMITE-2	nscaf3032	2052328	2052605	+
BmMITE-2	nscaf3031	196621	196898	+
BmMITE-2	nscaf3031	439374	439651	+
BmMITE-2	nscaf3031	601447	601722	+
BmMITE-2	nscaf3031	1060902	1061179	+
BmMITE-2	nscaf3031	1187405	1187682	+
BmMITE-2	nscaf3031	2419263	2419540	+
BmMITE-2	nscaf3031	2754915	2755192	+
BmMITE-2	nscaf3031	3012180	3012456	+
BmMITE-2	nscaf3031	3384500	3384777	+
BmMITE-2	nscaf3031	3604103	3604380	+
BmMITE-2	nscaf3031	4287730	4288007	+
BmMITE-2	nscaf3031	4457220	4457497	+
BmMITE-2	nscaf3031	4474844	4475121	+
BmMITE-2	nscaf3031	4861746	4862023	+
BmMITE-2	nscaf3031	6176597	6176874	+
BmMITE-2	nscaf3026	155397	155674	+
BmMITE-2	nscaf3026	469635	469912	+
BmMITE-2	nscaf3026	546487	546764	+
BmMITE-2	nscaf3026	676547	676823	+
BmMITE-2	nscaf3026	1046035	1046312	+
BmMITE-2	nscaf3026	2138622	2138898	+
BmMITE-2	nscaf3026	2470871	2471148	+
BmMITE-2	nscaf3026	2673642	2673919	+
BmMITE-2	nscaf3026	3176315	3176592	+
BmMITE-2	nscaf3026	3384584	3384861	+
BmMITE-2	nscaf3022	103892	104170	+
BmMITE-2	nscaf3022	176511	176788	+
BmMITE-2	nscaf3022	204991	205267	+

BmMITE-2	nscaf3022	665233	665510	+
BmMITE-2	nscaf2998	516664	516941	+
BmMITE-2	nscaf2998	1139207	1139483	+
BmMITE-2	nscaf2998	1314245	1314522	+
BmMITE-2	nscaf2998	1605100	1605377	+
BmMITE-2	nscaf2983	18408	18685	+
BmMITE-2	nscaf2983	686021	686298	+
BmMITE-2	nscaf2983	739792	740069	+
BmMITE-2	nscaf2983	967860	968137	+
BmMITE-2	nscaf2983	1276126	1276403	+
BmMITE-2	nscaf2983	1369646	1369922	+
BmMITE-2	nscaf2983	1748420	1748697	+
BmMITE-2	nscaf2983	1968215	1968492	+
BmMITE-2	nscaf2983	2032092	2032369	+
BmMITE-2	nscaf2983	2177722	2177999	+
BmMITE-2	nscaf2983	3027090	3027367	+
BmMITE-2	nscaf2964	223353	223628	+
BmMITE-2	nscaf2964	402007	402284	+
BmMITE-2	nscaf2964	778371	778650	+
BmMITE-2	nscaf2964	1205070	1205347	+
BmMITE-2	nscaf2964	1215729	1216006	+
BmMITE-2	nscaf2964	1371369	1371646	+
BmMITE-2	nscaf2964	1464466	1464743	+
BmMITE-2	nscaf2964	1479125	1479402	+
BmMITE-2	nscaf2964	1539623	1539901	+
BmMITE-2	nscaf2964	1624514	1624790	+
BmMITE-2	nscaf2964	1764548	1764825	+
BmMITE-2	nscaf2964	1815310	1815587	+
BmMITE-2	nscaf2964	1884090	1884367	+
BmMITE-2	nscaf2964	1899337	1899615	+
BmMITE-2	nscaf2964	2257166	2257443	+
BmMITE-2	nscaf2964	2328212	2328488	+
BmMITE-2	nscaf2964	2610557	2610834	+
BmMITE-2	nscaf2964	2851587	2851863	+
BmMITE-2	nscaf2964	2989810	2990079	+
BmMITE-2	nscaf2964	3241973	3242251	+
BmMITE-2	nscaf2964	3338626	3338902	+
BmMITE-2	nscaf2964	3485228	3485505	+
BmMITE-2	nscaf2964	3505248	3505525	+
BmMITE-2	nscaf2964	3572410	3572686	+
BmMITE-2	nscaf2964	3648180	3648457	+
BmMITE-2	nscaf2964	3877916	3878193	+
BmMITE-2	nscaf2964	3902210	3902486	+

BmMITE-2	nscaf2964	4014575	4014852	+
BmMITE-2	nscaf2964	4066179	4066456	+
BmMITE-2	nscaf2964	4374316	4374593	+
BmMITE-2	nscaf2964	4524767	4525044	+
BmMITE-2	nscaf2964	4615399	4615676	+
BmMITE-2	nscaf2964	4683326	4683606	+
BmMITE-2	nscaf2964	4766054	4766541	+
BmMITE-2	nscaf2964	4911238	4911515	+
BmMITE-2	nscaf2951	205453	205730	+
BmMITE-2	nscaf2951	341830	342107	+
BmMITE-2	nscaf2951	426530	426806	+
BmMITE-2	nscaf2951	507145	507423	+
BmMITE-2	nscaf2951	940351	940628	+
BmMITE-2	nscaf2951	1027440	1027716	+
BmMITE-2	nscaf2951	1413072	1413349	+
BmMITE-2	nscaf2951	1480209	1480486	+
BmMITE-2	nscaf2951	1538054	1538331	+
BmMITE-2	nscaf2943	966075	966352	+
BmMITE-2	nscaf2943	1025614	1025891	+
BmMITE-2	nscaf2943	1062498	1062774	+
BmMITE-2	nscaf2943	1274281	1274558	+
BmMITE-2	nscaf2943	1445303	1445580	+
BmMITE-2	nscaf2943	1523806	1524083	+
BmMITE-2	nscaf2943	1749038	1749315	+
BmMITE-2	nscaf2943	2116274	2116550	+
BmMITE-2	nscaf2943	2188824	2189101	+
BmMITE-2	nscaf2943	2662854	2663131	+
BmMITE-2	nscaf2943	3034047	3034324	+
BmMITE-2	nscaf2943	3162909	3163186	+
BmMITE-2	nscaf2943	3315448	3315731	+
BmMITE-2	nscaf2943	3881288	3881565	+
BmMITE-2	nscaf2927	60624	60901	+
BmMITE-2	nscaf2927	148583	148859	+
BmMITE-2	nscaf2927	185249	185526	+
BmMITE-2	nscaf2903	1168594	1168871	+
BmMITE-2	nscaf2903	1410209	1410491	+
BmMITE-2	nscaf2902	503009	503285	+
BmMITE-2	nscaf2902	774037	774314	+
BmMITE-2	nscaf2902	994695	994972	+
BmMITE-2	nscaf2902	1237816	1238093	+
BmMITE-2	nscaf2902	1334433	1334710	+
BmMITE-2	nscaf2902	1366254	1366531	+
BmMITE-2	nscaf2902	2231350	2231627	+

BmMITE-2	nscaf2902	2346847	2347124	+
BmMITE-2	nscaf2902	2514999	2515276	+
BmMITE-2	nscaf2902	2600935	2601212	+
BmMITE-2	nscaf2902	2671062	2671334	+
BmMITE-2	nscaf2902	2724881	2725158	+
BmMITE-2	nscaf2902	3616963	3617240	+
BmMITE-2	nscaf2902	3658331	3658609	+
BmMITE-2	nscaf2902	3888634	3888912	+
BmMITE-2	nscaf2902	4418419	4418696	+
BmMITE-2	nscaf2902	4740182	4740455	+
BmMITE-2	nscaf2902	5582694	5582971	+
BmMITE-2	nscaf2902	6304790	6305066	+
BmMITE-2	nscaf2902	6356064	6356341	+
BmMITE-2	nscaf2902	7287274	7287557	+
BmMITE-2	nscaf2902	7370961	7371238	+
BmMITE-2	nscaf2902	8090345	8090622	+
BmMITE-2	nscaf2902	8580142	8580418	+
BmMITE-2	nscaf2902	8796905	8797178	+
BmMITE-2	nscaf2902	9172648	9172924	+
BmMITE-2	nscaf2902	9989556	9989833	+
BmMITE-2	nscaf2902	10235839	10236116	+
BmMITE-2	nscaf2902	10536008	10536285	+
BmMITE-2	nscaf2902	11106157	11106434	+
BmMITE-2	nscaf2902	11142841	11143118	+
BmMITE-2	nscaf2901	1299640	1299917	+
BmMITE-2	nscaf2901	1408450	1408727	+
BmMITE-2	nscaf2876	1168670	1168947	+
BmMITE-2	nscaf2876	1185590	1185866	+
BmMITE-2	nscaf2876	1409909	1410180	+
BmMITE-2	nscaf2868	238848	239125	+
BmMITE-2	nscaf2868	625417	625694	+
BmMITE-2	nscaf2868	668269	668546	+
BmMITE-2	nscaf2868	1790145	1790422	+
BmMITE-2	nscaf2860	268708	268985	+
BmMITE-2	nscaf2860	477937	478213	+
BmMITE-2	nscaf2860	1782554	1782831	+
BmMITE-2	nscaf2860	1933616	1933892	+
BmMITE-2	nscaf2860	2868301	2868578	+
BmMITE-2	nscaf2855	295636	295913	+
BmMITE-2	nscaf2855	1183616	1183893	+
BmMITE-2	nscaf2855	1779617	1779894	+
BmMITE-2	nscaf2855	2051989	2052266	+
BmMITE-2	nscaf2855	2965672	2965944	+

BmMITE-2	nscaf2855	3295069	3295347	+
BmMITE-2	nscaf2855	3321484	3321761	+
BmMITE-2	nscaf2855	3390473	3390750	+
BmMITE-2	nscaf2855	3692408	3692685	+
BmMITE-2	nscaf2855	4262732	4263009	+
BmMITE-2	nscaf2855	5172996	5173273	+
BmMITE-2	nscaf2855	5253053	5253330	+
BmMITE-2	nscaf2855	5369584	5369861	+
BmMITE-2	nscaf2855	5678942	5679218	+
BmMITE-2	nscaf2855	5873076	5873353	+
BmMITE-2	nscaf2855	6053182	6053459	+
BmMITE-2	nscaf2855	6096885	6097162	+
BmMITE-2	nscaf2855	6259083	6259360	+
BmMITE-2	nscaf2855	6742370	6742647	+
BmMITE-2	nscaf2853	674572	674849	+
BmMITE-2	nscaf2853	986184	986461	+
BmMITE-2	nscaf2853	1200217	1200494	+
BmMITE-2	nscaf2853	1328035	1328312	+
BmMITE-2	nscaf2853	1815164	1815441	+
BmMITE-2	nscaf2853	2070120	2070397	+
BmMITE-2	nscaf2853	2419776	2420053	+
BmMITE-2	nscaf2853	2639638	2639915	+
BmMITE-2	nscaf2853	2694910	2695187	+
BmMITE-2	nscaf2853	3885266	3885543	+
BmMITE-2	nscaf2853	4408345	4408626	+
BmMITE-2	nscaf2853	4500734	4501011	+
BmMITE-2	nscaf2853	4852686	4852963	+
BmMITE-2	nscaf2853	4941166	4941443	+
BmMITE-2	nscaf2853	5296666	5296943	+
BmMITE-2	nscaf2853	5342027	5342504	+
BmMITE-2	nscaf2853	5772668	5772944	+
BmMITE-2	nscaf2853	6334631	6334908	+
BmMITE-2	nscaf2853	6631994	6632271	+
BmMITE-2	nscaf2842	273216	273493	+
BmMITE-2	nscaf2842	978876	979153	+
BmMITE-2	nscaf2842	1965138	1965415	+
BmMITE-2	nscaf2839	45920	46197	+
BmMITE-2	nscaf2839	397429	397706	+
BmMITE-2	nscaf2829	20896	21172	+
BmMITE-2	nscaf2829	786393	786670	+
BmMITE-2	nscaf2829	1173367	1173644	+
BmMITE-2	nscaf2829	1432219	1432496	+
BmMITE-2	nscaf2829	1449491	1449767	+

BmMITE-2	nscaf2829	2653012	2653288	+
BmMITE-2	nscaf2829	2813153	2813430	+
BmMITE-2	nscaf2829	2883543	2883820	+
BmMITE-2	nscaf2829	3572678	3572955	+
BmMITE-2	nscaf2829	4176495	4176772	+
BmMITE-2	nscaf2828	364271	364547	+
BmMITE-2	nscaf2828	1030623	1030900	+
BmMITE-2	nscaf2828	2069230	2069507	+
BmMITE-2	nscaf2828	2189927	2190194	+
BmMITE-2	nscaf2828	2237683	2237960	+
BmMITE-2	nscaf2828	3005564	3005841	+
BmMITE-2	nscaf2828	3651177	3651446	+
BmMITE-2	nscaf2828	3741382	3741659	+
BmMITE-2	nscaf2828	3875189	3875466	+
BmMITE-2	nscaf2828	4214088	4214365	+
BmMITE-2	nscaf2828	4509674	4509951	+
BmMITE-2	nscaf2828	4560575	4560852	+
BmMITE-2	nscaf2828	5322521	5322798	+
BmMITE-2	nscaf2828	5391418	5391695	+
BmMITE-2	nscaf2828	5417889	5418166	+
BmMITE-2	nscaf2828	5740867	5741144	+
BmMITE-2	nscaf2828	5880274	5880551	+
BmMITE-2	nscaf2823	91795	92072	+
BmMITE-2	nscaf2823	140209	140486	+
BmMITE-2	nscaf2823	1055540	1055817	+
BmMITE-2	nscaf2823	1592989	1593266	+
BmMITE-2	nscaf2823	2405151	2405428	+
BmMITE-2	nscaf2823	2465652	2465943	+
BmMITE-2	nscaf2823	2918503	2918780	+
BmMITE-2	nscaf2823	3030029	3030306	+
BmMITE-2	nscaf2823	3093925	3094202	+
BmMITE-2	nscaf2823	3411585	3411866	+
BmMITE-2	nscaf2823	3646614	3646891	+
BmMITE-2	nscaf2823	3885795	3886072	+
BmMITE-2	nscaf2823	4175646	4175923	+
BmMITE-2	nscaf2823	4247747	4248024	+
BmMITE-2	nscaf2823	4305082	4305359	+
BmMITE-2	nscaf2823	4357393	4357670	+
BmMITE-2	nscaf2822	54349	54626	+
BmMITE-2	nscaf2822	324930	325207	+
BmMITE-2	nscaf2822	358210	358487	+
BmMITE-2	nscaf2822	695887	696164	+
BmMITE-2	nscaf2822	977830	978107	+

BmMITE-2	nscaf2822	1093876	1094153	+
BmMITE-2	nscaf2822	1116349	1116629	+
BmMITE-2	nscaf2822	1386872	1387149	+
BmMITE-2	nscaf2822	1472487	1472764	+
BmMITE-2	nscaf2822	1555969	1556246	+
BmMITE-2	nscaf2800	126959	127236	+
BmMITE-2	nscaf2800	421559	421837	+
BmMITE-2	nscaf2800	638181	638458	+
BmMITE-2	nscaf2800	694801	695077	+
BmMITE-2	nscaf2800	787240	787517	+
BmMITE-2	nscaf2800	1479152	1479429	+
BmMITE-2	nscaf2800	1844340	1844617	+
BmMITE-2	nscaf2800	2576042	2576319	+
BmMITE-2	nscaf2800	2797055	2797332	+
BmMITE-2	nscaf2796	114440	114717	+
BmMITE-2	nscaf2795	275295	275572	+
BmMITE-2	nscaf2795	332861	333138	+
BmMITE-2	nscaf2795	535725	536002	+
BmMITE-2	nscaf2795	546965	547242	+
BmMITE-2	nscaf2795	660735	661012	+
BmMITE-2	nscaf2795	781426	781703	+
BmMITE-2	nscaf2795	989879	990156	+
BmMITE-2	nscaf2795	1103283	1103560	+
BmMITE-2	nscaf2795	1239743	1240020	+
BmMITE-2	nscaf2795	1481195	1481472	+
BmMITE-2	nscaf2795	1553499	1553776	+
BmMITE-2	nscaf2795	2701760	2702037	+
BmMITE-2	nscaf2795	3145685	3145962	+
BmMITE-2	nscaf2795	3363762	3364039	+
BmMITE-2	nscaf2795	3457780	3458057	+
BmMITE-2	nscaf2795	3700566	3700843	+
BmMITE-2	nscaf2789	70500	70777	+
BmMITE-2	nscaf2789	176506	176783	+
BmMITE-2	nscaf2789	319138	319414	+
BmMITE-2	nscaf2789	411197	411480	+
BmMITE-2	nscaf2789	476366	476640	+
BmMITE-2	nscaf2789	1052190	1052471	+
BmMITE-2	nscaf2789	1209332	1209609	+
BmMITE-2	nscaf2789	1318676	1318952	+
BmMITE-2	nscaf2789	1392739	1393016	+
BmMITE-2	nscaf2789	1453532	1453809	+
BmMITE-2	nscaf2789	1539946	1540225	+
BmMITE-2	nscaf2783	50180	50457	+

BmMITE-2	nscaf2770	881054	881331	+
BmMITE-2	nscaf2770	922007	922284	+
BmMITE-2	nscaf2770	1360393	1360670	+
BmMITE-2	nscaf2770	2034809	2035086	+
BmMITE-2	nscaf2763	19125	19402	+
BmMITE-2	nscaf2655	220846	221123	+
BmMITE-2	nscaf2655	512116	512393	+
BmMITE-2	nscaf2655	938123	938401	+
BmMITE-2	nscaf2655	1192944	1193221	+
BmMITE-2	nscaf2655	1959961	1960237	+
BmMITE-2	nscaf2655	2737423	2737699	+
BmMITE-2	nscaf2655	3106830	3107105	+
BmMITE-2	nscaf2655	3213306	3213583	+
BmMITE-2	nscaf2655	3421608	3421885	+
BmMITE-2	nscaf2655	3474729	3475006	+
BmMITE-2	nscaf2589	228418	228694	+
BmMITE-2	nscaf2589	1241196	1241473	+
BmMITE-2	nscaf2589	1313960	1314237	+
BmMITE-2	nscaf2589	1443975	1444253	+
BmMITE-2	nscaf2589	2609708	2609986	+
BmMITE-2	nscaf2589	2806160	2806437	+
BmMITE-2	nscaf2589	3016604	3016881	+
BmMITE-2	nscaf2589	3103445	3103721	+
BmMITE-2	nscaf2589	3730528	3730806	+
BmMITE-2	nscaf2589	3815703	3815980	+
BmMITE-2	nscaf2589	4406714	4406991	+
BmMITE-2	nscaf2589	4477311	4477588	+
BmMITE-2	nscaf2589	4747641	4747918	+
BmMITE-2	nscaf2589	4857943	4858220	+
BmMITE-2	nscaf2511	33503	33780	+
BmMITE-2	nscaf2511	558613	558890	+
BmMITE-2	nscaf2511	1328671	1328948	+
BmMITE-2	nscaf2511	1591205	1592234	+
BmMITE-2	nscaf2511	2227559	2227836	+
BmMITE-2	nscaf2511	2772081	2772360	+
BmMITE-2	nscaf2511	2784501	2784777	+
BmMITE-2	nscaf2511	2812515	2812792	+
BmMITE-2	nscaf2511	3393508	3394217	+
BmMITE-2	nscaf2511	3852718	3852999	+
BmMITE-2	nscaf2511	4000109	4000386	+
BmMITE-2	nscaf2511	4939381	4939658	+
BmMITE-2	nscaf2511	5410402	5410679	+
BmMITE-2	nscaf2511	5505915	5506192	+

BmMITE-2	nscaf2511	5946320	5946597	+
BmMITE-2	nscaf2511	6296429	6296706	+
BmMITE-2	nscaf2204	212361	212638	+
BmMITE-2	nscaf2204	805621	805898	+
BmMITE-2	nscaf2204	982948	983225	+
BmMITE-2	nscaf2204	1552182	1552459	+
BmMITE-2	nscaf2204	1752943	1753220	+
BmMITE-2	nscaf2204	2591005	2591282	+
BmMITE-2	nscaf2204	2734629	2734906	+
BmMITE-2	nscaf2204	2808800	2809077	+
BmMITE-2	nscaf2204	2914832	2915109	+
BmMITE-2	nscaf2204	3646487	3646764	+
BmMITE-2	nscaf2204	3886952	3887229	+
BmMITE-2	nscaf2204	3960090	3960366	+
BmMITE-2	nscaf2204	4424101	4424378	+
BmMITE-2	nscaf2176	392013	392291	+
BmMITE-2	nscaf2176	774090	774367	+
BmMITE-2	nscaf2176	1055811	1056081	+
BmMITE-2	nscaf2176	1163059	1163336	+
BmMITE-2	nscaf2176	1256993	1257270	+
BmMITE-2	nscaf2176	1275291	1275568	+
BmMITE-2	nscaf2176	2569291	2569568	+
BmMITE-2	nscaf2176	2800014	2800291	+
BmMITE-2	nscaf2176	3356270	3356547	+
BmMITE-2	nscaf2176	3793371	3793648	+
BmMITE-2	nscaf2176	4113204	4113481	+
BmMITE-2	nscaf2176	4286980	4287257	+
BmMITE-2	nscaf2136	193783	194061	+
BmMITE-2	nscaf2136	487933	488210	+
BmMITE-2	nscaf2136	776419	776696	+
BmMITE-2	nscaf2136	950173	950450	+
BmMITE-2	nscaf2136	1719297	1719574	+
BmMITE-2	nscaf2136	2697716	2697993	+
BmMITE-2	nscaf2136	3265241	3265517	+
BmMITE-2	nscaf2136	3739312	3739589	+
BmMITE-2	nscaf2136	5012704	5012987	+
BmMITE-2	nscaf2136	7029981	7030258	+
BmMITE-2	nscaf2136	7056655	7056932	+
BmMITE-2	nscaf2136	8040357	8040634	+
BmMITE-2	nscaf2136	8268487	8268763	+
BmMITE-2	nscaf1898	1717561	1717838	+
BmMITE-2	nscaf1898	1771148	1771425	+
BmMITE-2	nscaf1898	2535223	2535500	+

BmMITE-2	nscaf1898	4017354	4017631	+
BmMITE-2	nscaf1898	4045963	4046241	+
BmMITE-2	nscaf1898	4221622	4221899	+
BmMITE-2	nscaf1898	4528905	4529182	+
BmMITE-2	nscaf1898	4691333	4691610	+
BmMITE-2	nscaf1898	5603196	5603473	+
BmMITE-2	nscaf1898	5658957	5659234	+
BmMITE-2	nscaf1898	5698316	5698593	+
BmMITE-2	nscaf1898	5732740	5733017	+
BmMITE-2	nscaf1898	7278044	7278321	+
BmMITE-2	nscaf1898	9128105	9128382	+
BmMITE-2	nscaf1898	9171788	9172065	+
BmMITE-2	nscaf1898	9252773	9253050	+
BmMITE-2	nscaf1898	9765754	9766031	+
BmMITE-2	nscaf1898	10135876	10136153	+
BmMITE-2	nscaf1898	10166809	10167086	+
BmMITE-2	nscaf1898	10440112	10440389	+
BmMITE-2	nscaf1898	10806978	10807255	+
BmMITE-2	nscaf1898	10953352	10953629	+
BmMITE-2	nscaf1898	11189744	11190021	+
BmMITE-2	nscaf1898	12398632	12398909	+
BmMITE-2	nscaf1898	12463504	12463781	+
BmMITE-2	nscaf1898	12483383	12483660	+
BmMITE-2	nscaf1898	12520438	12520715	+
BmMITE-2	nscaf1898	12553800	12554077	+
BmMITE-2	nscaf1898	12803848	12804125	+
BmMITE-2	nscaf1898	14220687	14220964	+
BmMITE-2	nscaf1898	14775041	14775317	+
BmMITE-2	nscaf1690	176432	176710	+
BmMITE-2	nscaf1690	301783	302060	+
BmMITE-2	nscaf1690	702900	703177	+
BmMITE-2	nscaf1690	849454	849731	+
BmMITE-2	nscaf1690	1034487	1034763	+
BmMITE-2	nscaf1690	1137857	1138134	+
BmMITE-2	nscaf1690	3091089	3091366	+
BmMITE-2	nscaf1690	3492477	3492754	+
BmMITE-2	nscaf1690	3632055	3632332	+
BmMITE-2	nscaf1690	4977526	4977803	+
BmMITE-2	nscaf1690	5166678	5166951	+
BmMITE-2	nscaf1690	5227180	5227457	+
BmMITE-2	nscaf1690	5271709	5271986	+
BmMITE-2	nscaf1690	5756284	5756559	+
BmMITE-2	nscaf1690	6056786	6057063	+

BmMITE-2	nscaf1690	6610338	6610615	+
BmMITE-2	nscaf1690	6666199	6666474	+
BmMITE-2	nscaf1690	7568783	7569060	+
BmMITE-2	nscaf1690	7942449	7942726	+
BmMITE-2	nscaf1681	16338	16615	+
BmMITE-2	nscaf1681	1331093	1331370	+
BmMITE-2	nscaf1681	1501448	1501724	+
BmMITE-2	nscaf1681	1928350	1928627	+
BmMITE-2	nscaf1681	2089116	2089393	+
BmMITE-2	nscaf1681	2289308	2289585	+
BmMITE-2	nscaf1681	2779212	2779493	+
BmMITE-2	nscaf1681	2986018	2986295	+
BmMITE-2	nscaf1681	4962192	4962464	+
BmMITE-2	nscaf1681	5424315	5424592	+
BmMITE-2	nscaf1681	5562195	5562472	+
BmMITE-2	nscaf1681	5787788	5788065	+
BmMITE-2	nscaf1681	5880410	5880686	+
BmMITE-2	nscaf3034	485286	485561	+
BmMITE-2	nscaf3034	2115138	2115415	+
BmMITE-2	nscaf3034	2457365	2457642	+
BmMITE-2	nscaf3034	2861139	2861414	+
BmMITE-2	nscaf3034	2942814	2943091	+
BmMITE-2	nscaf3034	3444262	3444539	+
BmMITE-2	nscaf3034	4013797	4014074	+
BmMITE-2	nscaf3034	5008414	5008691	+
BmMITE-2	nscaf2948	154979	155292	+
BmMITE-2	nscaf2948	366271	366548	+
BmMITE-2	nscaf2948	750957	751234	+
BmMITE-2	nscaf2948	1025482	1025759	+
BmMITE-2	nscaf2948	1196412	1196689	+
BmMITE-2	nscaf2948	1435760	1436037	+
BmMITE-2	nscaf2948	1700820	1701097	+
BmMITE-2	nscaf2948	1810048	1810323	+
BmMITE-2	scaffold31022	69	346	+
BmMITE-2	scaffold2499	1706	1983	+
BmMITE-2	scaffold1044	3730	4007	+
BmMITE-2	scaffold1031	9852	10129	+
BmMITE-2	nscaf98	885061	885337	+
BmMITE-2	nscaf98	975881	976158	+
BmMITE-2	nscaf98	1080315	1080592	+
BmMITE-2	nscaf98	1224364	1224641	+
BmMITE-2	nscaf98	1585410	1585687	+
BmMITE-2	nscaf463	68088	68365	+

BmMITE-2	nscaf463	1475708	1475985	+
BmMITE-2	nscaf463	1516711	1516988	+
BmMITE-2	nscaf463	1543766	1544043	+
BmMITE-2	nscaf3075	1205128	1205405	+
BmMITE-2	nscaf3072	585216	585493	+
BmMITE-2	nscaf3072	677842	678119	+
BmMITE-2	nscaf3072	696046	696323	+
BmMITE-2	nscaf3072	2100749	2101026	+
BmMITE-2	nscaf3072	2370007	2370284	+
BmMITE-2	nscaf3072	2528771	2529049	+
BmMITE-2	nscaf3072	2859556	2859833	+
BmMITE-2	nscaf3072	2960597	2960875	+
BmMITE-2	nscaf3063	819796	820073	+
BmMITE-2	nscaf3063	1079184	1079460	+
BmMITE-2	nscaf3063	1421412	1421689	+
BmMITE-2	nscaf3063	1511392	1511669	+
BmMITE-2	nscaf3063	2081880	2082156	+
BmMITE-2	nscaf3063	2253836	2254113	+
BmMITE-2	nscaf3063	2273702	2273979	+
BmMITE-2	nscaf3063	2514161	2514438	+
BmMITE-2	nscaf3063	2769146	2769423	+
BmMITE-2	nscaf3063	2799586	2799863	+
BmMITE-2	nscaf3063	2822172	2822449	+
BmMITE-2	nscaf3063	3299289	3299565	+
BmMITE-2	nscaf3063	3541440	3541717	+
BmMITE-2	nscaf3055	932562	932839	+
BmMITE-2	nscaf3055	1202432	1202709	+
BmMITE-2	nscaf3055	1316453	1316730	+
BmMITE-2	nscaf3041	862395	862672	+
BmMITE-2	nscaf3041	989442	989719	+
BmMITE-2	nscaf3040	1461276	1461569	+
BmMITE-2	nscaf3040	1705589	1705866	+
BmMITE-2	nscaf3040	2268279	2268556	+
BmMITE-2	nscaf3040	2381858	2382135	+
BmMITE-2	nscaf3040	2437761	2438038	+
BmMITE-2	nscaf3040	2521333	2521610	+
BmMITE-2	nscaf3040	2823790	2824066	+
BmMITE-2	nscaf3040	3535905	3536183	+
BmMITE-2	nscaf3040	3567647	3567924	+
BmMITE-2	nscaf3040	4662663	4662939	+
BmMITE-2	nscaf3027	925780	926056	+
BmMITE-2	nscaf3027	1903083	1903360	+
BmMITE-2	nscaf3027	3572456	3572734	+

BmMITE-2	nscaf3027	4080666	4080940	+
BmMITE-2	nscaf3027	5159288	5159565	+
BmMITE-2	nscaf3015	1445698	1445975	+
BmMITE-2	nscaf3015	1938371	1938647	+
BmMITE-2	nscaf3015	2442604	2442881	+
BmMITE-2	nscaf3015	3302962	3303239	+
BmMITE-2	nscaf3015	4247216	4247493	+
BmMITE-2	nscaf3015	4259408	4259685	+
BmMITE-2	nscaf3008	366221	366498	+
BmMITE-2	nscaf3008	674864	675141	+
BmMITE-2	nscaf3008	712360	712637	+
BmMITE-2	nscaf3008	881556	881833	+
BmMITE-2	nscaf2993	322677	322954	+
BmMITE-2	nscaf2993	446405	446681	+
BmMITE-2	nscaf2993	678203	678480	+
BmMITE-2	nscaf2993	729540	729824	+
BmMITE-2	nscaf2993	1152421	1152698	+
BmMITE-2	nscaf2993	1217757	1218034	+
BmMITE-2	nscaf2993	1473305	1473582	+
BmMITE-2	nscaf2993	1866511	1866788	+
BmMITE-2	nscaf2993	1987551	1987828	+
BmMITE-2	nscaf2993	2875042	2875319	+
BmMITE-2	nscaf2993	3212720	3212997	+
BmMITE-2	nscaf2993	3962634	3962911	+
BmMITE-2	nscaf2993	4811859	4812136	+
BmMITE-2	nscaf2993	4862908	4863190	+
BmMITE-2	nscaf2993	4926152	4926429	+
BmMITE-2	nscaf2993	5296426	5296703	+
BmMITE-2	nscaf2993	5525121	5525399	+
BmMITE-2	nscaf2993	5591113	5591390	+
BmMITE-2	nscaf2993	6047302	6047578	+
BmMITE-2	nscaf2993	6517383	6517660	+
BmMITE-2	nscaf2993	6751238	6751516	+
BmMITE-2	nscaf2993	6771983	6772266	+
BmMITE-2	nscaf2993	7810306	7810580	+
BmMITE-2	nscaf2993	8095025	8095302	+
BmMITE-2	nscaf2970	387268	387583	+
BmMITE-2	nscaf2970	444807	445084	+
BmMITE-2	nscaf2970	792696	792972	+
BmMITE-2	nscaf2970	938301	938579	+
BmMITE-2	nscaf2970	964195	964472	+
BmMITE-2	nscaf2970	1078129	1078406	+
BmMITE-2	nscaf2970	1553944	1554221	+

BmMITE-2	nscaf2970	1589557	1589834	+
BmMITE-2	nscaf2970	1912837	1913109	+
BmMITE-2	nscaf2970	2564358	2564635	+
BmMITE-2	nscaf2970	2637180	2637456	+
BmMITE-2	nscaf2970	2671461	2671738	+
BmMITE-2	nscaf2890	720760	721037	+
BmMITE-2	nscaf2890	949533	949810	+
BmMITE-2	nscaf2890	1304458	1304735	+
BmMITE-2	nscaf2883	678452	678729	+
BmMITE-2	nscaf2883	735679	735956	+
BmMITE-2	nscaf2883	2113116	2113393	+
BmMITE-2	nscaf2874	177977	178254	+
BmMITE-2	nscaf2874	189006	189282	+
BmMITE-2	nscaf2874	225759	226055	+
BmMITE-2	nscaf2874	308161	308438	+
BmMITE-2	nscaf2874	535086	535362	+
BmMITE-2	nscaf2852	185965	186242	+
BmMITE-2	nscaf2852	1931926	1932203	+
BmMITE-2	nscaf2852	2329394	2329671	+
BmMITE-2	nscaf2833	22817	23094	+
BmMITE-2	nscaf2833	38096	38373	+
BmMITE-2	nscaf2833	114353	114630	+
BmMITE-2	nscaf2833	241409	241686	+
BmMITE-2	nscaf2833	256860	257138	+
BmMITE-2	nscaf2833	450496	450772	+
BmMITE-2	nscaf2825	375649	375927	+
BmMITE-2	nscaf2825	419780	420056	+
BmMITE-2	nscaf2825	502090	502367	+
BmMITE-2	nscaf2815	344188	344465	+
BmMITE-2	nscaf2815	1532003	1532280	+
BmMITE-2	nscaf2815	1704603	1704880	+
BmMITE-2	nscaf2815	2106333	2106611	+
BmMITE-2	nscaf2815	2299161	2299438	+
BmMITE-2	nscaf2801	531670	531947	+
BmMITE-2	nscaf2797	501378	501655	+
BmMITE-2	nscaf2797	1069122	1069398	+
BmMITE-2	nscaf2797	1346359	1346636	+
BmMITE-2	nscaf2792	22811	23088	+
BmMITE-2	nscaf2681	49688	49965	+
BmMITE-2	nscaf2681	264840	265117	+
BmMITE-2	nscaf2681	307856	308133	+
BmMITE-2	nscaf2681	759192	759469	+
BmMITE-2	nscaf2681	912086	912363	+

BmMITE-2	nscaf2681	1160484	1160761	+
BmMITE-2	nscaf2674	281615	281892	+
BmMITE-2	nscaf2674	1077671	1077948	+
BmMITE-2	nscaf2674	1765545	1765822	+
BmMITE-2	nscaf2674	1826003	1826279	+
BmMITE-2	nscaf2674	1911107	1911384	+
BmMITE-2	nscaf2674	2112099	2112376	+
BmMITE-2	nscaf2674	2205853	2206130	+
BmMITE-2	nscaf2674	2686066	2686343	+
BmMITE-2	nscaf2674	2777920	2778197	+
BmMITE-2	nscaf2674	2972931	2973208	+
BmMITE-2	nscaf2674	3247905	3248226	+
BmMITE-2	nscaf2674	4437467	4437744	+
BmMITE-2	nscaf2674	5597979	5598256	+
BmMITE-2	nscaf2674	5627843	5628120	+
BmMITE-2	nscaf2674	5893300	5893577	+
BmMITE-2	nscaf2674	6537113	6537390	+
BmMITE-2	nscaf2674	6613313	6613590	+
BmMITE-2	nscaf2674	7740888	7741155	+
BmMITE-2	nscaf2674	7842927	7843204	+
BmMITE-2	nscaf2210	556874	557151	+
BmMITE-2	nscaf2210	1699199	1699476	+
BmMITE-2	nscaf2210	1748344	1748621	+
BmMITE-2	nscaf2210	1919650	1919927	+
BmMITE-2	nscaf2210	2700509	2700786	+
BmMITE-2	nscaf2210	3081000	3081277	+
BmMITE-2	nscaf2142	2215	2492	+
BmMITE-2	nscaf2937	98954	99230	+
BmMITE-2	nscaf2937	487946	488223	+
BmMITE-2	nscaf2937	945532	945809	+
BmMITE-2	nscaf2937	1049079	1049356	+
BmMITE-2	nscaf3079	23370	23647	+
BmMITE-2	nscaf3079	418233	418510	+
BmMITE-2	nscaf3079	767112	767392	+
BmMITE-2	nscaf3079	799178	799455	+
BmMITE-2	nscaf3079	981886	982163	+
BmMITE-2	nscaf3079	1071384	1071661	+
BmMITE-2	nscaf3079	1497993	1498270	+
BmMITE-2	scaffold874	6208	6485	+
BmMITE-2	scaffold790	395	672	+
BmMITE-2	scaffold33173	19	296	+
BmMITE-2	scaffold1275	3844	4121	+
BmMITE-2	nscaf736	5142	5419	+

BmMITE-2	nscaf736	31207	31484	+
BmMITE-2	nscaf3074	335430	335707	+
BmMITE-2	nscaf3074	498568	498845	+
BmMITE-2	nscaf3056	168100	168377	+
BmMITE-2	nscaf3056	293872	294149	+
BmMITE-2	nscaf3053	143410	143685	+
BmMITE-2	nscaf3053	585350	585627	+
BmMITE-2	nscaf3044	260144	260421	+
BmMITE-2	nscaf3044	670813	671090	+
BmMITE-2	nscaf3044	1527659	1527936	+
BmMITE-2	nscaf3013	205217	205494	+
BmMITE-2	nscaf3013	781094	781371	+
BmMITE-2	nscaf3013	1379991	1380268	+
BmMITE-2	nscaf3005	278413	278691	+
BmMITE-2	nscaf3005	1140801	1141078	+
BmMITE-2	nscaf2965	1809	2085	+
BmMITE-2	nscaf2965	196524	196802	+
BmMITE-2	nscaf2965	260571	260848	+
BmMITE-2	nscaf2916	157884	158161	+
BmMITE-2	nscaf2916	225428	225705	+
BmMITE-2	nscaf2905	159032	159309	+
BmMITE-2	nscaf2899	236817	237094	+
BmMITE-2	nscaf2899	413641	413918	+
BmMITE-2	nscaf2891	653674	653951	+
BmMITE-2	nscaf2891	1036822	1037099	+
BmMITE-2	nscaf2891	1937499	1937776	+
BmMITE-2	nscaf2891	2078442	2078719	+
BmMITE-2	nscaf2891	2228882	2229156	+
BmMITE-2	nscaf2891	2257702	2257979	+
BmMITE-2	nscaf2891	2310382	2310658	+
BmMITE-2	nscaf2891	2395770	2396047	+
BmMITE-2	nscaf2891	2555332	2555609	+
BmMITE-2	nscaf2891	2642848	2643125	+
BmMITE-2	nscaf2851	369280	369557	+
BmMITE-2	nscaf2851	924976	925253	+
BmMITE-2	nscaf2838	102280	102557	+
BmMITE-2	nscaf2838	373045	373322	+
BmMITE-2	nscaf2838	1663926	1664203	+
BmMITE-2	nscaf2838	1819634	1819911	+
BmMITE-2	nscaf2836	91756	92047	+
BmMITE-2	nscaf2836	131728	132005	+
BmMITE-2	nscaf2836	295232	295509	+
BmMITE-2	nscaf2836	485854	486131	+

BmMITE-2	nscaf2734	1226681	1226958	+
BmMITE-2	nscaf2734	1398133	1398410	+
BmMITE-2	nscaf2686	662873	663150	+
BmMITE-2	nscaf2686	1026658	1026935	+
BmMITE-2	nscaf2686	1047483	1047760	+
BmMITE-2	nscaf2686	1089854	1090133	+
BmMITE-2	nscaf2686	1108638	1108915	+
BmMITE-2	nscaf2564	22593	22870	+
BmMITE-2	nscaf2216	957203	957480	+
BmMITE-2	nscaf1485	4439	4716	+
BmMITE-2	nscaf1346	350	627	+
BmMITE-2	nscaf1289	20254	20531	+
BmMITE-2	nscaf1289	57563	57840	+
BmMITE-2	scaffold606	32424	32702	+
BmMITE-2	scaffold2204	82	359	+
BmMITE-2	nscaf3066	164859	165136	+
BmMITE-2	nscaf3066	292286	292563	+
BmMITE-2	nscaf3066	390268	390545	+
BmMITE-2	nscaf3066	1443073	1443350	+
BmMITE-2	nscaf3052	1070985	1071262	+
BmMITE-2	nscaf2971	298331	298608	+
BmMITE-2	nscaf2912	54674	54951	+
BmMITE-2	nscaf2912	757038	757315	+
BmMITE-2	nscaf2912	800790	801067	+
BmMITE-2	nscaf2912	1145372	1145644	+
BmMITE-2	nscaf2889	1556668	1556945	+
BmMITE-2	nscaf2889	1842482	1842759	+
BmMITE-2	nscaf2889	1981320	1981597	+
BmMITE-2	nscaf2889	2381683	2381959	+
BmMITE-2	nscaf2889	2734877	2735154	+
BmMITE-2	nscaf2819	9381	9658	+
BmMITE-2	nscaf2819	42131	42408	+
BmMITE-2	nscaf2814	52347	52624	+
BmMITE-2	nscaf125	136587	136864	+
BmMITE-2	nscaf3037	42871	43152	+
BmMITE-2	nscaf3037	63415	63693	+
BmMITE-2	nscaf2810	268988	269264	+
BmMITE-2	nscaf2810	380330	380600	+
BmMITE-2	nscaf2793	23102	23379	+
BmMITE-2	nscaf1705	805493	805769	+
BmMITE-2	nscaf1705	829072	829365	+
BmMITE-2	nscaf1705	925931	926208	+
BmMITE-2	nscaf1705	1058140	1058417	+

BmMITE-2	nscaf1705	1254852	1255129	+
BmMITE-2	scaffold783	12852	13129	+
BmMITE-2	scaffold752	30686	30960	+
BmMITE-2	scaffold719	19159	19436	+
BmMITE-2	scaffold37888	14	291	+
BmMITE-2	scaffold32526	300	577	+
BmMITE-2	scaffold2649	435	712	+
BmMITE-2	scaffold22769	366	643	+
BmMITE-2	scaffold1612	1244	1521	+
BmMITE-2	scaffold1504	2595	2872	+
BmMITE-2	scaffold1253	1886	2163	+
BmMITE-2	scaffold12143	125	402	+
BmMITE-2	scaffold1207	3128	3405	+
BmMITE-2	scaffold1135	4635	4912	+
BmMITE-2	scaffold1027	10372	10649	+
BmMITE-2	nscaf3138	10057	10334	+
BmMITE-2	nscaf3060	131574	131851	+
BmMITE-2	nscaf3002	19622	19900	+
BmMITE-2	nscaf3002	241406	241683	+
BmMITE-2	nscaf2990	238418	238695	+
BmMITE-2	nscaf2990	831365	831642	+
BmMITE-2	nscaf2962	105711	105988	+
BmMITE-2	nscaf2962	522652	522929	+
BmMITE-2	nscaf2962	590785	591062	+
BmMITE-2	nscaf2962	811647	811929	+
BmMITE-2	nscaf2924	251340	251653	+
BmMITE-2	nscaf2924	295639	295916	+
BmMITE-2	nscaf2924	451622	451899	+
BmMITE-2	nscaf2924	510140	510417	+
BmMITE-2	nscaf2813	169009	169286	+
BmMITE-2	nscaf2813	301850	302126	+
BmMITE-2	nscaf2813	767884	768160	+
BmMITE-2	nscaf2780	220304	220581	+
BmMITE-2	nscaf2766	1056037	1056314	+
BmMITE-2	nscaf1814	8910	9187	+
BmMITE-2	nscaf1732	3587	3864	+
BmMITE-2	nscaf2913	126135	126411	+
BmMITE-2	scaffold4941	483	760	+
BmMITE-2	scaffold33884	33	309	+
BmMITE-2	scaffold24468	15	292	+
BmMITE-2	scaffold21580	3	279	+
BmMITE-2	nscaf71	1730	2006	+
BmMITE-2	nscaf484	5622	5899	+

BmMITE-2	nscaf2961	370	647	+
BmMITE-2	nscaf2309	9811	10087	+
BmMITE-2	nscaf2309	20801	21078	+
BmMITE-2	nscaf3005	1505486	1506233	+
BmMITE-2	nscaf3098	655942	656327	+
BmMITE-2	nscaf2902	3476365	3476661	+
BmMITE-2	nscaf3027	2942374	2942669	+
BmMITE-2	nscaf2964	767208	767495	+
BmMITE-2	nscaf2823	3400869	3401156	+
BmMITE-2	nscaf2853	1241665	1241956	+
BmMITE-2	nscaf2930	3596113	3596391	+
BmMITE-2	nscaf3058	4030895	4031173	+
BmMITE-2	nscaf3026	4322541	4322819	+
BmMITE-2	nscaf2828	3320184	3320462	+
BmMITE-2	nscaf1705	1450524	1450802	+
BmMITE-2	nscaf3026	748984	749260	+
BmMITE-2	nscaf2951	677623	677899	+
BmMITE-2	nscaf2902	7403977	7404252	+
BmMITE-2	nscaf2829	414818	415096	+
BmMITE-2	nscaf2795	1086240	1086525	+
BmMITE-2	nscaf3040	3605381	3605907	+
BmMITE-2	nscaf2801	601010	601288	+
BmMITE-2	nscaf2931	697119	697394	+
BmMITE-2	nscaf2930	1678693	1678968	+
BmMITE-2	nscaf3058	887410	887686	+
BmMITE-2	nscaf2818	28502	28780	+
BmMITE-2	nscaf3035	1495893	1496155	+
BmMITE-2	nscaf2795	348994	349268	+
BmMITE-2	nscaf3034	3352284	3352562	+
BmMITE-2	nscaf2865	2720071	2720342	+
BmMITE-2	nscaf1681	1711076	1711353	+
BmMITE-2	nscaf2891	1475924	1476185	+
BmMITE-2	nscaf2686	726421	726699	+
BmMITE-2	nscaf2847	1397576	1397845	+
BmMITE-2	nscaf1690	6095112	6095394	+
BmMITE-2	nscaf2575	3158569	3158850	+
BmMITE-2	nscaf3099	3534610	3534885	+
BmMITE-2	nscaf3040	448987	449249	+
BmMITE-2	nscaf2930	5966178	5966447	+
BmMITE-2	nscaf2655	3722815	3723072	+
BmMITE-2	nscaf2848	55431	55744	+
BmMITE-2	nscaf2948	6685	6937	+
BmMITE-2	nscaf2964	3032320	3032587	+

BmMITE-2	nscaf2943	897871	898165	+
BmMITE-2	nscaf2766	1018957	1019216	+
BmMITE-2	nscaf2176	2393803	2394045	+
BmMITE-2	nscaf2136	3948394	3948649	+
BmMITE-2	nscaf1681	4345278	4345549	+
BmMITE-2	nscaf2826	186193	186431	+
BmMITE-2	nscaf2204	622690	622943	+
BmMITE-2	nscaf3063	2037735	2037964	+
BmMITE-2	nscaf2943	1297659	1297907	+
BmMITE-3	nscaf1690	780321	780016	-
BmMITE-3	nscaf2853	368266	367961	-
BmMITE-3	nscaf481	1297473	1297169	-
BmMITE-3	nscaf481	1193920	1193615	-
BmMITE-3	nscaf481	284378	284073	-
BmMITE-3	nscaf481	266046	265741	-
BmMITE-3	nscaf2888	310519	310214	-
BmMITE-3	nscaf2623	1502765	1502460	-
BmMITE-3	nscaf2964	3408662	3408357	-
BmMITE-3	nscaf2889	1987347	1987042	-
BmMITE-3	nscaf773	44025	43710	-
BmMITE-3	nscaf2855	3283638	3283329	-
BmMITE-3	nscaf2860	219914	219451	-
BmMITE-3	nscaf3072	750561	750099	-
BmMITE-3	nscaf2912	475884	475446	-
BmMITE-3	nscaf3034	5098254	5097792	-
BmMITE-3	nscaf2937	1240505	1240055	-
BmMITE-3	nscaf2916	489849	489581	-
BmMITE-3	nscaf1690	621120	621425	+
BmMITE-3	scaffold21516	204	509	+
BmMITE-3	nscaf2575	2124312	2124617	+
BmMITE-3	nscaf2623	528635	528938	+
BmMITE-3	nscaf3063	2083730	2084035	+
BmMITE-3	nscaf2330	113401	113706	+
BmMITE-3	nscaf2330	2405008	2405387	+
BmMITE-3	nscaf3031	314375	314678	+
BmMITE-3	nscaf2993	7687429	7687734	+
BmMITE-3	nscaf2851	1396512	1396817	+
BmMITE-3	nscaf3066	754291	754599	+
BmMITE-3	nscaf2847	1354984	1355283	+
BmMITE-3	nscaf2674	30325	30790	+
BmMITE-3	nscaf3079	1044941	1045407	+
BmMITE-3	nscaf2655	2696848	2697311	+

BmMITE-3	nscaf3053	256837	257211	+
BmMITE-4	nscaf2136	1494111	1493545	-
BmMITE-4	nscaf3026	1574967	1574401	-
BmMITE-4	nscaf2589	1130360	1129794	-
BmMITE-4	nscaf2891	1548858	1548272	-
BmMITE-4	nscaf2674	1858501	1857892	-
BmMITE-4	nscaf3132	5247	5832	+
BmMITE-4	nscaf2888	4017816	4018428	+
BmMITE-5	nscaf2330	3584998	3584543	-
BmMITE-5	nscaf2330	757189	756977	-
BmMITE-5	nscaf2912	54220	54007	-
BmMITE-5	nscaf2888	2803418	2803207	-
BmMITE-5	nscaf1898	14625552	14625341	-
BmMITE-5	nscaf2930	186629	186418	-
BmMITE-5	nscaf2986	268038	267827	-
BmMITE-5	nscaf2902	5796316	5796104	-
BmMITE-5	nscaf2902	383403	383192	-
BmMITE-5	nscaf2851	1553768	1553557	-
BmMITE-5	nscaf2795	3308148	3307936	-
BmMITE-5	nscaf2993	4769735	4769531	-
BmMITE-5	nscaf2993	1694253	1694042	-
BmMITE-5	nscaf2136	6747985	6747401	-
BmMITE-5	nscaf2136	6194885	6194675	-
BmMITE-5	nscaf2136	4952596	4952385	-
BmMITE-5	nscaf3094	50049	49838	-
BmMITE-5	nscaf3055	267111	266900	-
BmMITE-5	nscaf2931	1057889	1057678	-
BmMITE-5	nscaf2855	2611320	2611111	-
BmMITE-5	nscaf2828	291270	291060	-
BmMITE-5	nscaf2810	442363	442155	-
BmMITE-5	nscaf3040	2093113	2092901	-
BmMITE-5	nscaf3034	731458	731247	-
BmMITE-5	nscaf3008	930562	930350	-
BmMITE-5	nscaf3045	2156121	2155469	-
BmMITE-5	nscaf2176	538423	538219	-
BmMITE-5	scaffold418	343911	339644	-
BmMITE-5	nscaf2916	666029	665808	-
BmMITE-5	nscaf2931	18101	17433	-
BmMITE-5	nscaf3058	974966	973803	-
BmMITE-5	nscaf2674	6436365	6435713	-
BmMITE-5	nscaf2136	2594204	2593723	-

BmMITE-5	nscaf2951	1428857	1428456	-
BmMITE-5	nscaf2964	3998913	3998263	-
BmMITE-5	nscaf2987	426545	426360	-
BmMITE-5	nscaf2855	635332	634772	-
BmMITE-5	nscaf2793	59726	59134	-
BmMITE-5	nscaf2853	974043	972988	-
BmMITE-5	nscaf463	1522249	1522064	-
BmMITE-5	nscaf2204	427350	427553	+
BmMITE-5	nscaf2204	1075804	1076016	+
BmMITE-5	nscaf2575	648043	648695	+
BmMITE-5	nscaf2575	2065850	2066060	+
BmMITE-5	nscaf2575	2342029	2342233	+
BmMITE-5	nscaf2575	3492309	3492520	+
BmMITE-5	nscaf2943	3068893	3069104	+
BmMITE-5	nscaf3032	1164070	1164282	+
BmMITE-5	nscaf2953	522450	522661	+
BmMITE-5	nscaf1898	5994523	5994734	+
BmMITE-5	nscaf1898	7724644	7725294	+
BmMITE-5	nscaf2930	5766169	5766380	+
BmMITE-5	nscaf2795	2392006	2392217	+
BmMITE-5	nscaf2993	133856	134061	+
BmMITE-5	nscaf2993	4342538	4342748	+
BmMITE-5	nscaf2951	510767	510978	+
BmMITE-5	nscaf2823	2412996	2413207	+
BmMITE-5	nscaf3003	4493446	4493658	+
BmMITE-5	nscaf2891	1879954	1880158	+
BmMITE-5	nscaf2899	236393	236604	+
BmMITE-5	nscaf2818	434483	435073	+
BmMITE-5	nscaf3058	5008547	5008758	+
BmMITE-5	nscaf3038	25234	25447	+
BmMITE-5	nscaf2828	2919168	2919372	+
BmMITE-5	nscaf3079	1198865	1199072	+
BmMITE-5	nscaf3097	2777763	2777967	+
BmMITE-5	nscaf3052	356746	356952	+
BmMITE-5	nscaf2797	1302630	1302835	+
BmMITE-5	nscaf2789	60307	60958	+
BmMITE-5	nscaf3068	66922	68511	+
BmMITE-5	nscaf2983	956179	956728	+
BmMITE-5	nscaf2210	1471055	1471705	+
BmMITE-5	nscaf3003	4141728	4142371	+
BmMITE-5	nscaf2801	434406	434658	+
BmMITE-5	nscaf1705	718984	719175	+
BmMITE-5	nscaf1108	1360753	1361414	+

BmMITE-5	nscaf2986	364156	364588	+
BmMITE-5	nscaf2993	3026706	3027021	+
BmMITE-5	nscaf2655	302950	303146	+
BmMITE-5	nscaf1690	7823914	7824443	+
BmMITE-5	nscaf2818	2864798	2865398	+
BmMITE-5	nscaf3045	308978	309182	+
BmMITE-6	nscaf3007	71502	71233	-
BmMITE-6	nscaf2998	1430250	1429982	-
BmMITE-6	nscaf1690	5912276	5913025	+
BmMITE-6	nscaf2948	670561	670796	+
BmMITE-6	nscaf2889	3753673	3753921	+
BmMITE-6	nscaf2943	1891371	1891609	+
BmMITE-6	nscaf2993	6773428	6773691	+
BmMITE-6	nscaf2964	2562728	2563396	+
BmMITE-7	nscaf2136	718247	717699	-
BmMITE-7	nscaf2575	1982182	1981645	-
BmMITE-7	nscaf2888	8042794	8042257	-
BmMITE-7	nscaf2888	495189	494363	-
BmMITE-7	nscaf3055	795575	795041	-
BmMITE-7	nscaf2823	3763529	3762982	-
BmMITE-7	nscaf2767	2393514	2394061	+
BmMITE-7	nscaf1898	12405467	12406014	+
BmMITE-7	scaffold8193	178	715	+
BmMITE-7	nscaf3005	1107935	1108484	+
BmMITE-7	nscaf2829	3694950	3695499	+
BmMITE-7	nscaf1962	233674	234220	+
BmMITE-7	nscaf2828	3240525	3241937	+
BmMITE-8	nscaf2686	96069	95803	-
BmMITE-8	nscaf2823	2756104	2755840	-
BmMITE-8	nscaf3048	1006433	1006168	-
BmMITE-8	nscaf2853	3903764	3903499	-
BmMITE-8	nscaf2953	2038271	2038012	-
BmMITE-8	nscaf2931	1961038	1960781	-
BmMITE-8	nscaf2210	3863810	3863542	-
BmMITE-8	nscaf2176	2144099	2143836	-
BmMITE-8	scaffold42334	395	130	-
BmMITE-8	nscaf3027	1764322	1764057	-
BmMITE-8	nscaf2986	5258841	5258584	-
BmMITE-8	nscaf2964	954979	954714	-
BmMITE-8	nscaf2800	868447	868182	-

BmMITE-8	nscaf2511	3370261	3369994	-
BmMITE-8	nscaf1681	5160644	5159680	-
BmMITE-8	nscaf1681	4861834	4861570	-
BmMITE-8	nscaf2801	109958	109690	-
BmMITE-8	nscaf2330	2558640	2558374	-
BmMITE-8	nscaf2811	356793	356528	-
BmMITE-8	nscaf2780	84996	84731	-
BmMITE-8	nscaf2833	238748	238490	-
BmMITE-8	nscaf3040	1230754	1230512	-
BmMITE-8	nscaf2889	280317	280076	-
BmMITE-8	nscaf2795	648308	648067	-
BmMITE-8	nscaf1898	5658696	5658244	-
BmMITE-8	nscaf1898	3213624	3213011	-
BmMITE-8	nscaf2888	5237332	5235880	-
BmMITE-8	nscaf2176	3380962	3380722	-
BmMITE-8	nscaf2176	1796694	1796454	-
BmMITE-8	nscaf2176	1504381	1504140	-
BmMITE-8	nscaf2986	3283173	3282931	-
BmMITE-8	nscaf2930	5316992	5316755	-
BmMITE-8	nscaf2842	1277695	1277454	-
BmMITE-8	nscaf2398	713197	712949	-
BmMITE-8	nscaf2886	36288	36046	-
BmMITE-8	nscaf3099	3014688	3014448	-
BmMITE-8	nscaf3099	1478997	1478756	-
BmMITE-8	nscaf2993	7047736	7047495	-
BmMITE-8	nscaf2993	1473242	1472999	-
BmMITE-8	nscaf2891	2329046	2328766	-
BmMITE-8	nscaf98	1517506	1517263	-
BmMITE-8	nscaf2828	2383424	2383184	-
BmMITE-8	nscaf2655	300473	300235	-
BmMITE-8	nscaf2959	322120	321876	-
BmMITE-8	nscaf2767	4257987	4257750	-
BmMITE-8	nscaf2767	3247268	3247027	-
BmMITE-8	nscaf2767	555342	555100	-
BmMITE-8	nscaf2868	88358	88118	-
BmMITE-8	nscaf2655	2701581	2701339	-
BmMITE-8	nscaf2948	949926	949686	-
BmMITE-8	nscaf3103	1840	1221	-
BmMITE-8	nscaf2853	2618012	2617761	-
BmMITE-8	nscaf2529	2818011	2817754	-
BmMITE-8	nscaf2903	372383	372091	-
BmMITE-8	nscaf2964	618013	617774	-
BmMITE-8	nscaf2823	1613388	1613139	-

BmMITE-8	nscaf2865	5244134	5243885	-
BmMITE-8	nscaf2853	6016346	6016099	-
BmMITE-8	nscaf2815	766609	766359	-
BmMITE-8	nscaf2890	467300	467057	-
BmMITE-8	nscaf2930	2307714	2307471	-
BmMITE-8	nscaf2589	6655748	6655510	-
BmMITE-8	nscaf2852	2626878	2626607	-
BmMITE-8	nscaf2829	3902089	3901853	-
BmMITE-8	nscaf2529	3019651	3019411	-
BmMITE-8	nscaf2589	6560286	6560042	-
BmMITE-8	nscaf2210	1799442	1799178	-
BmMITE-8	nscaf3055	953638	953373	-
BmMITE-8	nscaf3044	1428845	1428580	-
BmMITE-8	nscaf2891	2559839	2559441	-
BmMITE-8	nscaf3031	2059990	2059725	-
BmMITE-8	nscaf2686	109764	110029	+
BmMITE-8	nscaf1690	3563357	3563622	+
BmMITE-8	nscaf2899	429665	429930	+
BmMITE-8	nscaf2795	2554483	2554748	+
BmMITE-8	nscaf3079	1464224	1464487	+
BmMITE-8	nscaf3066	1030959	1031224	+
BmMITE-8	nscaf2136	1889498	1889762	+
BmMITE-8	nscaf2136	3352673	3352934	+
BmMITE-8	nscaf2937	234869	235135	+
BmMITE-8	nscaf2902	264741	265004	+
BmMITE-8	nscaf2902	8296195	8296459	+
BmMITE-8	nscaf2176	1385089	1385354	+
BmMITE-8	nscaf3015	3065020	3065287	+
BmMITE-8	nscaf2986	4264644	4264909	+
BmMITE-8	nscaf2964	4009688	4009946	+
BmMITE-8	nscaf2827	2022729	2022994	+
BmMITE-8	nscaf481	408784	409051	+
BmMITE-8	nscaf2916	151005	151273	+
BmMITE-8	nscaf2916	241846	242111	+
BmMITE-8	nscaf2330	1995033	1995297	+
BmMITE-8	nscaf3072	565558	565823	+
BmMITE-8	nscaf3063	2687245	2687510	+
BmMITE-8	nscaf2589	1229161	1229426	+
BmMITE-8	nscaf2589	1895483	1895744	+
BmMITE-8	nscaf2815	1838794	1839059	+
BmMITE-8	nscaf2935	57553	57822	+
BmMITE-8	nscaf2959	318009	318266	+
BmMITE-8	nscaf1289	89451	89716	+

BmMITE-8	nscaf3008	544572	544872	+
BmMITE-8	nscaf3026	3019358	3019605	+
BmMITE-8	nscaf2686	343649	343885	+
BmMITE-8	nscaf2795	740037	740276	+
BmMITE-8	nscaf2136	4162021	4162262	+
BmMITE-8	nscaf2136	4797458	4797699	+
BmMITE-8	nscaf1898	1960552	1960794	+
BmMITE-8	nscaf1898	3879166	3879405	+
BmMITE-8	nscaf3034	2456843	2457086	+
BmMITE-8	nscaf2902	253526	253767	+
BmMITE-8	nscaf2902	6706845	6707085	+
BmMITE-8	nscaf2888	5237090	5237332	+
BmMITE-8	nscaf2176	4222433	4222678	+
BmMITE-8	nscaf2800	1554035	1554276	+
BmMITE-8	nscaf481	1254349	1254590	+
BmMITE-8	nscaf2983	2004487	2004963	+
BmMITE-8	nscaf2930	3301301	3301541	+
BmMITE-8	nscaf2930	5941468	5941709	+
BmMITE-8	nscaf3058	2405348	2405596	+
BmMITE-8	nscaf3058	5629623	5629864	+
BmMITE-8	nscaf3058	7214148	7214394	+
BmMITE-8	nscaf3035	233758	234000	+
BmMITE-8	nscaf2789	915858	916096	+
BmMITE-8	nscaf3063	766784	767024	+
BmMITE-8	nscaf2948	1081728	1081969	+
BmMITE-8	nscaf2204	2580514	2580756	+
BmMITE-8	nscaf2770	2148435	2148676	+
BmMITE-8	nscaf3031	4402302	4402543	+
BmMITE-8	nscaf2828	5299962	5300203	+
BmMITE-8	nscaf3032	1879688	1879932	+
BmMITE-8	nscaf2873	128249	128487	+
BmMITE-8	nscaf2216	362700	362937	+
BmMITE-8	nscaf2529	282132	282379	+
BmMITE-8	nscaf1071	48512	48754	+
BmMITE-8	nscaf2883	2561982	2562224	+
BmMITE-8	nscaf3026	4183544	4183794	+
BmMITE-8	nscaf2891	1824262	1824504	+
BmMITE-8	nscaf2851	78156	78410	+
BmMITE-8	nscaf2795	936384	936608	+
BmMITE-8	nscaf2825	657706	657948	+
BmMITE-8	nscaf3071	609417	609659	+
BmMITE-8	nscaf2965	50243	50484	+
BmMITE-8	nscaf3038	165683	165948	+

BmMITE-8	nscaf2986	4595644	4595900	+
BmMITE-8	nscaf2986	4977005	4977270	+
BmMITE-8	nscaf2279	529925	530189	+
BmMITE-8	nscaf2847	5342177	5342445	+
BmMITE-8	nscaf2855	2391650	2391914	+
BmMITE-9	nscaf2767	28859	28442	-
BmMITE-9	nscaf2931	1566441	1566024	-
BmMITE-9	nscaf2931	698270	697853	-
BmMITE-9	nscaf2931	164054	163637	-
BmMITE-9	nscaf2770	540647	540230	-
BmMITE-9	nscaf2902	1515987	1515570	-
BmMITE-9	nscaf2833	494802	494385	-
BmMITE-9	nscaf3026	5974703	5974286	-
BmMITE-9	nscaf2655	435404	434987	-
BmMITE-9	nscaf3031	3305911	3305500	-
BmMITE-9	nscaf3031	1297920	1297503	-
BmMITE-9	nscaf2822	107268	106851	-
BmMITE-9	nscaf3062	49905	49488	-
BmMITE-9	nscaf3027	3901680	3901263	-
BmMITE-9	nscaf2886	899807	899390	-
BmMITE-9	nscaf2818	2929794	2929260	-
BmMITE-9	nscaf2575	3852336	3851885	-
BmMITE-9	nscaf2575	681611	681194	-
BmMITE-9	nscaf3005	1954511	1954095	-
BmMITE-9	nscaf2876	702651	702234	-
BmMITE-9	nscaf2847	3723801	3723384	-
BmMITE-9	nscaf2847	1054959	1054547	-
BmMITE-9	nscaf2800	2889267	2888852	-
BmMITE-9	nscaf2855	5260625	5260208	-
BmMITE-9	nscaf2855	2508293	2507876	-
BmMITE-9	nscaf2855	2172151	2171730	-
BmMITE-9	nscaf3097	1476425	1476011	-
BmMITE-9	nscaf2686	665683	665265	-
BmMITE-9	nscaf1071	695674	695250	-
BmMITE-9	nscaf3015	984821	984409	-
BmMITE-9	nscaf2674	2031050	2030633	-
BmMITE-9	nscaf2853	590080	589664	-
BmMITE-9	nscaf2136	136380	135845	-
BmMITE-9	nscaf3069	223685	222856	-
BmMITE-9	nscaf1681	2683944	2683516	-
BmMITE-9	nscaf2216	953752	953349	-
BmMITE-9	nscaf2795	3194225	3193386	-

BmMITE-9	nscaf2888	7993529	7993133	-
BmMITE-9	nscaf2903	421284	420888	-
BmMITE-9	nscaf3026	4971657	4970817	-
BmMITE-9	nscaf2575	3712449	3712051	-
BmMITE-9	nscaf2964	3304924	3304530	-
BmMITE-9	nscaf1289	67552	66754	-
BmMITE-9	nscaf2829	1554486	1553897	-
BmMITE-9	nscaf2883	2352614	2352264	-
BmMITE-9	nscaf2767	2291000	2291417	+
BmMITE-9	scaffold41472	31	448	+
BmMITE-9	scaffold29595	144	561	+
BmMITE-9	nscaf2176	3049816	3050233	+
BmMITE-9	nscaf3003	2211779	2212199	+
BmMITE-9	nscaf3003	4448604	4449021	+
BmMITE-9	nscaf2833	600043	600451	+
BmMITE-9	nscaf1108	383511	383928	+
BmMITE-9	nscaf3052	1240464	1240881	+
BmMITE-9	nscaf3026	4091931	4092348	+
BmMITE-9	nscaf2970	2004078	2004495	+
BmMITE-9	nscaf2860	2863811	2864228	+
BmMITE-9	nscaf2655	2772862	2773279	+
BmMITE-9	nscaf3031	4852274	4852692	+
BmMITE-9	nscaf2957	44262	44680	+
BmMITE-9	nscaf2888	2297778	2298192	+
BmMITE-9	nscaf2828	3878992	3879409	+
BmMITE-9	nscaf2529	2010098	2010515	+
BmMITE-9	nscaf2529	2531389	2531795	+
BmMITE-9	nscaf2398	761510	761927	+
BmMITE-9	nscaf3034	2344064	2344481	+
BmMITE-9	nscaf3034	3970620	3971037	+
BmMITE-9	nscaf2330	4231708	4232125	+
BmMITE-9	nscaf2793	92228	92639	+
BmMITE-9	scaffold921	4991	5408	+
BmMITE-9	nscaf1898	7523773	7524190	+
BmMITE-9	scaffold1197	4698	5121	+
BmMITE-9	nscaf1071	1156450	1156899	+
BmMITE-9	nscaf2053	588343	588760	+
BmMITE-9	nscaf3022	836702	837123	+
BmMITE-9	nscaf3093	23221	23635	+
BmMITE-9	nscaf481	1243506	1243923	+
BmMITE-9	nscaf2674	3973407	3973817	+
BmMITE-9	nscaf2964	1710131	1710548	+
BmMITE-9	nscaf2964	3294250	3295074	+

BmMITE-9	nscaf2859	271294	271704	+
BmMITE-9	nscaf2797	48121	48538	+
BmMITE-9	nscaf2901	1430231	1430640	+
BmMITE-9	nscaf3115	24847	25269	+
BmMITE-9	nscaf2902	1646595	1646977	+
BmMITE-9	nscaf3040	3576364	3576825	+
BmMITE-9	nscaf2951	627092	627483	+
BmMITE-9	nscaf2053	608938	609312	+
BmMITE-10	nscaf2964	668875	668618	-
BmMITE-10	nscaf3099	2813734	2813473	-
BmMITE-10	nscaf2943	3809969	3809716	-
BmMITE-10	nscaf2943	3315759	3315221	-
BmMITE-10	nscaf2930	6152942	6152688	-
BmMITE-10	nscaf2204	2652106	2651852	-
BmMITE-10	scaffold1566	4291	4037	-
BmMITE-10	nscaf3072	2986867	2986613	-
BmMITE-10	nscaf2998	1618473	1618219	-
BmMITE-10	nscaf2852	979284	979029	-
BmMITE-10	nscaf2589	1075169	1074915	-
BmMITE-10	nscaf2136	3459841	3459588	-
BmMITE-10	nscaf2822	414762	414505	-
BmMITE-10	nscaf2674	4031043	4030790	-
BmMITE-10	nscaf2210	4967496	4967243	-
BmMITE-10	nscaf2210	4210648	4210393	-
BmMITE-10	nscaf2888	2407943	2407611	-
BmMITE-10	nscaf2828	4357925	4357673	-
BmMITE-10	nscaf2828	2713363	2713106	-
BmMITE-10	nscaf2948	280020	279263	-
BmMITE-10	nscaf3026	5999470	5999216	-
BmMITE-10	nscaf2842	1491238	1490977	-
BmMITE-10	nscaf2655	2801296	2801037	-
BmMITE-10	nscaf2655	2494638	2494384	-
BmMITE-10	nscaf2797	270456	270202	-
BmMITE-10	scaffold5716	583	328	-
BmMITE-10	nscaf2993	6547744	6547493	-
BmMITE-10	nscaf2868	216099	215839	-
BmMITE-10	nscaf2529	2737353	2737101	-
BmMITE-10	nscaf3003	778901	778647	-
BmMITE-10	nscaf2681	235307	235051	-
BmMITE-10	scaffold3451	628	380	-
BmMITE-10	nscaf3055	525796	525433	-
BmMITE-10	nscaf2398	392783	392525	-

BmMITE-10	nscaf2789	480195	479945	-
BmMITE-10	nscaf3099	2587484	2587226	-
BmMITE-10	nscaf2883	1880693	1880440	-
BmMITE-10	nscaf2511	4378401	4378148	-
BmMITE-10	nscaf1690	3028722	3028470	-
BmMITE-10	nscaf2827	822460	822204	-
BmMITE-10	nscaf3062	203544	203290	-
BmMITE-10	nscaf2795	4057950	4057694	-
BmMITE-10	nscaf2983	1221794	1221536	-
BmMITE-10	nscaf2815	599068	598814	-
BmMITE-10	nscaf2681	596179	595926	-
BmMITE-10	nscaf2902	8209781	8209528	-
BmMITE-10	nscaf3063	333096	332835	-
BmMITE-10	nscaf1681	1990107	1989858	-
BmMITE-10	nscaf2842	69049	68795	-
BmMITE-10	nscaf2770	533400	532888	-
BmMITE-10	nscaf1485	19978	19699	-
BmMITE-10	nscaf2674	4424882	4424644	-
BmMITE-10	nscaf2734	1545817	1545216	-
BmMITE-10	nscaf3099	3576305	3576048	-
BmMITE-10	nscaf1108	3218334	3217626	-
BmMITE-10	nscaf3099	2824053	2823799	-
BmMITE-10	nscaf2902	3771325	3771068	-
BmMITE-10	nscaf2852	917183	916926	-
BmMITE-10	nscaf2797	1056561	1056194	-
BmMITE-10	nscaf2855	6197895	6197653	-
BmMITE-10	nscaf3003	2541503	2541249	-
BmMITE-10	nscaf1898	280556	280303	-
BmMITE-10	nscaf3058	8537084	8536841	-
BmMITE-10	nscaf2655	3226749	3226499	-
BmMITE-10	nscaf3026	6393639	6393114	-
BmMITE-10	nscaf3034	844089	843826	-
BmMITE-10	nscaf2970	1196012	1195750	-
BmMITE-10	nscaf3008	744094	743838	-
BmMITE-10	nscaf3058	3629105	3628847	-
BmMITE-10	nscaf2674	7774959	7774708	-
BmMITE-10	nscaf2930	1939308	1939074	-
BmMITE-10	nscaf2826	524556	524318	-
BmMITE-10	nscaf2800	499199	498970	-
BmMITE-10	nscaf2964	1887113	1886865	-
BmMITE-10	nscaf3063	3319691	3319461	-
BmMITE-10	nscaf2828	5851183	5850948	-
BmMITE-10	nscaf3099	1710300	1710557	+

BmMITE-10	nscaf2901	300933	301189	+
BmMITE-10	nscaf2943	3415227	3415481	+
BmMITE-10	nscaf2883	2697518	2697772	+
BmMITE-10	nscaf2836	446870	447122	+
BmMITE-10	nscaf3072	863074	863330	+
BmMITE-10	nscaf2902	71428	71681	+
BmMITE-10	nscaf2589	2368944	2369202	+
BmMITE-10	nscaf1898	9806494	9806749	+
BmMITE-10	nscaf3027	4727162	4727418	+
BmMITE-10	nscaf3027	4747555	4747809	+
BmMITE-10	nscaf2986	1629546	1629798	+
BmMITE-10	nscaf2986	5495489	5495742	+
BmMITE-10	nscaf2767	2402085	2402328	+
BmMITE-10	nscaf2674	1352445	1352698	+
BmMITE-10	nscaf2210	1879344	1879595	+
BmMITE-10	nscaf1681	5742771	5743026	+
BmMITE-10	nscaf2828	2339045	2339310	+
BmMITE-10	nscaf2828	3869602	3869854	+
BmMITE-10	nscaf2828	4545439	4545692	+
BmMITE-10	nscaf3026	4583567	4583821	+
BmMITE-10	nscaf2970	2279026	2279311	+
BmMITE-10	nscaf3034	4747163	4747489	+
BmMITE-10	nscaf3031	993946	994211	+
BmMITE-10	nscaf2993	258563	258816	+
BmMITE-10	nscaf2865	5407140	5407393	+
BmMITE-10	nscaf3022	892431	892688	+
BmMITE-10	nscaf2983	2717823	2718334	+
BmMITE-10	nscaf2900	206605	206858	+
BmMITE-10	nscaf2818	717739	717989	+
BmMITE-10	nscaf3079	1610784	1611038	+
BmMITE-10	nscaf3097	414874	415252	+
BmMITE-10	nscaf3058	8223956	8224214	+
BmMITE-10	nscaf3026	1511975	1512228	+
BmMITE-10	nscaf3022	144630	144889	+
BmMITE-10	nscaf1108	1704541	1704799	+
BmMITE-10	nscaf2893	52548	52801	+
BmMITE-10	nscaf2868	776487	777124	+
BmMITE-10	nscaf2943	2528936	2529190	+
BmMITE-10	nscaf2943	3369369	3369623	+
BmMITE-10	nscaf2828	431975	432238	+
BmMITE-10	nscaf2983	2737644	2737898	+
BmMITE-10	nscaf2818	3112588	3112844	+
BmMITE-10	scaffold1536	1179	1432	+

BmMITE-10	nscaf2838	242060	242322	+
BmMITE-10	nscaf3022	491031	491293	+
BmMITE-10	nscaf3063	1497452	1497703	+
BmMITE-10	nscaf2943	3838298	3838556	+
BmMITE-10	nscaf3058	7215959	7216235	+
BmMITE-10	nscaf2853	5228689	5228926	+
BmMITE-10	nscaf1690	48746	48981	+
BmMITE-10	nscaf2789	1396821	1397344	+
BmMITE-10	nscaf2889	3800144	3800394	+
BmMITE-10	nscaf481	848292	848532	+
BmMITE-10	nscaf2204	2449901	2450584	+
BmMITE-10	nscaf3099	4633290	4633544	+
BmMITE-10	nscaf2902	524154	524410	+
BmMITE-10	nscaf2826	734216	734760	+
BmMITE-10	nscaf3072	1224688	1225670	+
BmMITE-10	nscaf2902	3835487	3835732	+
BmMITE-10	nscaf3040	2880024	2880268	+
BmMITE-10	nscaf3079	1420199	1420447	+
BmMITE-10	nscaf3015	1546198	1546467	+
BmMITE-10	nscaf2883	2457405	2457870	+
BmMITE-10	nscaf2822	446253	446508	+
BmMITE-10	nscaf2888	5867793	5868054	+
BmMITE-10	nscaf2888	9381257	9381510	+
BmMITE-10	nscaf2853	1609218	1609471	+
BmMITE-10	nscaf2529	4761653	4761908	+
BmMITE-10	nscaf3078	64078	64320	+
BmMITE-10	nscaf1690	5128122	5128369	+
BmMITE-10	nscaf2681	228066	228310	+
BmMITE-10	nscaf2829	1568309	1568813	+
BmMITE-10	nscaf2954	38675	39166	+
BmMITE-10	nscaf2136	4211850	4212106	+
BmMITE-10	nscaf2865	170521	170800	+
BmMITE-10	nscaf2825	211941	212192	+
BmMITE-10	nscaf2930	5457405	5457637	+
BmMITE-10	nscaf2800	2079339	2079581	+
BmMITE-10	nscaf2951	368195	368791	+
BmMITE-10	nscaf3044	1381309	1381545	+
BmMITE-10	nscaf3099	3808044	3808430	+
BmMITE-10	scaffold9919	90	351	+
BmMITE-11	nscaf3066	669814	669623	-
BmMITE-11	scaffold3087	592	401	-
BmMITE-11	nscaf3093	723006	722815	-

BmMITE-11	nscaf3093	709447	709256	-
BmMITE-11	nscaf481	415512	415320	-
BmMITE-11	scaffold9485	685	497	-
BmMITE-11	scaffold20124	252	64	-
BmMITE-11	nscaf2136	1447203	1447440	+
BmMITE-11	nscaf2937	353297	353487	+
BmMITE-11	nscaf2943	2462615	2462807	+
BmMITE-11	nscaf2943	3782357	3782546	+
BmMITE-11	nscaf2951	1061258	1061541	+
BmMITE-11	nscaf2210	675121	675311	+
BmMITE-11	nscaf87	23046	23237	+
BmMITE-11	scaffold5517	285	474	+
BmMITE-12	scaffold32891	304	95	-
BmMITE-12	nscaf3097	3167559	3167354	-
BmMITE-12	nscaf3097	318924	318719	-
BmMITE-12	nscaf3027	1522245	1522040	-
BmMITE-12	nscaf2136	7696272	7696067	-
BmMITE-12	nscaf1690	6254341	6254136	-
BmMITE-12	scaffold21700	421	212	-
BmMITE-12	nscaf2855	5640452	5640247	-
BmMITE-12	nscaf2847	3396128	3395923	-
BmMITE-12	nscaf3026	3680543	3680338	-
BmMITE-12	nscaf2828	33718	33509	-
BmMITE-12	nscaf2891	832267	832062	-
BmMITE-12	nscaf2883	2602705	2602500	-
BmMITE-12	nscaf2883	2200860	2200651	-
BmMITE-12	nscaf481	431279	431074	-
BmMITE-12	scaffold30670	321	112	-
BmMITE-12	scaffold2810	1075	866	-
BmMITE-12	nscaf3034	938658	938451	-
BmMITE-12	nscaf2983	405849	405640	-
BmMITE-12	nscaf3058	8198306	8198101	-
BmMITE-12	nscaf3058	3680273	3680064	-
BmMITE-12	nscaf2924	669280	669072	-
BmMITE-12	nscaf2842	2083870	2083662	-
BmMITE-12	scaffold9887	631	426	-
BmMITE-12	scaffold27327	433	228	-
BmMITE-12	scaffold26884	364	159	-
BmMITE-12	nscaf2993	6929778	6929573	-
BmMITE-12	nscaf2888	5824539	5824334	-
BmMITE-12	nscaf2210	1723356	1723151	-
BmMITE-12	scaffold18659	289	83	-

BmMITE-12	nscaf3045	2477928	2477723	-
BmMITE-12	nscaf2931	1797562	1797357	-
BmMITE-12	nscaf2930	4123957	4123752	-
BmMITE-12	nscaf2886	989402	989197	-
BmMITE-12	nscaf3099	1990275	1990070	-
BmMITE-12	nscaf2204	2285	2080	-
BmMITE-12	scaffold19109	255	50	-
BmMITE-12	nscaf2810	341412	341207	-
BmMITE-12	nscaf2953	13204	12999	-
BmMITE-12	nscaf2853	5791658	5791447	-
BmMITE-12	nscaf2889	2986030	2985827	-
BmMITE-12	nscaf3075	1275626	1275421	-
BmMITE-12	nscaf2948	1197993	1197789	-
BmMITE-12	scaffold26825	276	69	-
BmMITE-12	nscaf2511	348149	347947	-
BmMITE-12	nscaf2902	563456	563251	-
BmMITE-12	nscaf2829	2583884	2583680	-
BmMITE-12	nscaf3033	130113	129914	-
BmMITE-12	nscaf98	789984	789781	-
BmMITE-12	nscaf3098	882286	882082	-
BmMITE-12	nscaf2998	766750	766555	-
BmMITE-12	nscaf3027	1367374	1367162	-
BmMITE-12	nscaf2890	695622	695421	-
BmMITE-12	nscaf2136	3010006	3009797	-
BmMITE-12	nscaf2136	1603659	1603450	-
BmMITE-12	nscaf1690	4546076	4545868	-
BmMITE-12	scaffold20573	364	155	-
BmMITE-12	nscaf2847	4719502	4719292	-
BmMITE-12	nscaf3026	3857172	3856963	-
BmMITE-12	scaffold3149	938	731	-
BmMITE-12	nscaf3097	192441	192650	+
BmMITE-12	scaffold27574	228	437	+
BmMITE-12	nscaf2937	347670	347879	+
BmMITE-12	nscaf2890	1334940	1335149	+
BmMITE-12	nscaf2865	3024146	3024355	+
BmMITE-12	nscaf2865	5085344	5085550	+
BmMITE-12	nscaf2789	633925	634134	+
BmMITE-12	nscaf2770	2282068	2282277	+
BmMITE-12	nscaf2674	4177887	4178096	+
BmMITE-12	nscaf2674	5823210	5823419	+
BmMITE-12	nscaf2136	7012110	7012310	+
BmMITE-12	nscaf3066	1228220	1228428	+
BmMITE-12	nscaf3056	101619	101828	+

BmMITE-12	nscaf2855	5265741	5265953	+
BmMITE-12	nscaf2847	768558	768763	+
BmMITE-12	nscaf2847	6477151	6477352	+
BmMITE-12	scaffold416	284384	284593	+
BmMITE-12	scaffold27429	242	451	+
BmMITE-12	nscaf2828	575584	575785	+
BmMITE-12	scaffold757	310	519	+
BmMITE-12	scaffold17214	343	552	+
BmMITE-12	nscaf2891	579328	579537	+
BmMITE-12	nscaf2891	1093095	1093304	+
BmMITE-12	nscaf1898	6515561	6515772	+
BmMITE-12	nscaf2795	1702198	1702403	+
BmMITE-12	nscaf3079	738098	738306	+
BmMITE-12	scaffold693	7043	7252	+
BmMITE-12	scaffold17355	223	432	+
BmMITE-12	nscaf2983	1240494	1240703	+
BmMITE-12	scaffold26958	166	371	+
BmMITE-12	nscaf2575	3064535	3064740	+
BmMITE-12	scaffold6547	282	490	+
BmMITE-12	nscaf3058	3295346	3295551	+
BmMITE-12	scaffold36870	277	482	+
BmMITE-12	scaffold25968	427	632	+
BmMITE-12	scaffold22940	421	626	+
BmMITE-12	nscaf2993	4027810	4028015	+
BmMITE-12	nscaf2888	6058940	6059139	+
BmMITE-12	nscaf2943	798028	798233	+
BmMITE-12	nscaf1705	871052	871260	+
BmMITE-12	scaffold14101	242	447	+
BmMITE-12	nscaf2910	282757	282962	+
BmMITE-12	scaffold32679	283	488	+
BmMITE-12	scaffold19476	240	445	+
BmMITE-12	nscaf2655	1161818	1162023	+
BmMITE-12	nscaf2529	1907302	1907507	+
BmMITE-12	nscaf2529	2222287	2222492	+
BmMITE-12	nscaf2216	698678	698883	+
BmMITE-12	scaffold18798	236	440	+
BmMITE-12	nscaf3075	120904	121113	+
BmMITE-12	nscaf3035	1446690	1446893	+
BmMITE-12	nscaf2808	22836	23044	+
BmMITE-12	nscaf2330	3817082	3817286	+
BmMITE-12	scaffold32593	340	549	+
BmMITE-12	nscaf3041	693583	693792	+
BmMITE-12	nscaf2766	709507	709716	+

BmMITE-12	nscaf3106	237236	237440	+
BmMITE-12	nscaf2210	989499	989687	+
BmMITE-12	nscaf2136	5394346	5394541	+
BmMITE-12	nscaf3013	889521	889730	+
BmMITE-12	nscaf3027	671548	671756	+
BmMITE-12	nscaf2674	4124081	4124290	+
BmMITE-12	nscaf2795	466893	467108	+
BmMITE-12	nscaf3079	68708	68920	+
BmMITE-12	nscaf2589	5278497	5278713	+
BmMITE-13	nscaf3026	6491184	6490922	-
BmMITE-13	nscaf3026	4527408	4527152	-
BmMITE-13	nscaf3026	2463663	2463401	-
BmMITE-13	nscaf2954	51407	51139	-
BmMITE-13	nscaf2859	1775447	1775185	-
BmMITE-13	nscaf2855	2914399	2914145	-
BmMITE-13	nscaf2847	7847158	7846896	-
BmMITE-13	nscaf2847	6530742	6530477	-
BmMITE-13	nscaf2847	4050011	4049749	-
BmMITE-13	nscaf2847	2377327	2377066	-
BmMITE-13	nscaf2829	2951473	2951211	-
BmMITE-13	nscaf2767	3894986	3894724	-
BmMITE-13	nscaf2767	3540322	3540066	-
BmMITE-13	nscaf1898	15554661	15554397	-
BmMITE-13	nscaf1898	9744640	9744378	-
BmMITE-13	nscaf3055	262024	261763	-
BmMITE-13	nscaf3027	4488195	4487933	-
BmMITE-13	nscaf3027	1747306	1747044	-
BmMITE-13	nscaf3027	845802	845540	-
BmMITE-13	nscaf3027	78392	78130	-
BmMITE-13	nscaf2930	3772750	3772487	-
BmMITE-13	nscaf2930	1703749	1703487	-
BmMITE-13	nscaf2681	844345	844083	-
BmMITE-13	nscaf2589	5929292	5929032	-
BmMITE-13	nscaf2589	2477611	2477348	-
BmMITE-13	nscaf2529	3534760	3534499	-
BmMITE-13	nscaf2912	1277151	1276889	-
BmMITE-13	nscaf2912	998611	998344	-
BmMITE-13	nscaf2902	10847797	10847534	-
BmMITE-13	nscaf2902	5942553	5942291	-
BmMITE-13	nscaf2902	4176524	4176260	-
BmMITE-13	nscaf2891	2671637	2671375	-
BmMITE-13	nscaf2136	604555	604291	-

BmMITE-13	nscaf2822	234654	234391	-
BmMITE-13	nscaf481	1067367	1067105	-
BmMITE-13	nscaf3099	3826240	3825981	-
BmMITE-13	nscaf3099	2859445	2859183	-
BmMITE-13	nscaf3008	344387	344125	-
BmMITE-13	nscaf2986	3187489	3187227	-
BmMITE-13	nscaf2888	3706925	3706662	-
BmMITE-13	nscaf2888	1027289	1027027	-
BmMITE-13	nscaf2853	5939297	5939035	-
BmMITE-13	nscaf2970	2298523	2298263	-
BmMITE-13	nscaf2655	3663622	3663357	-
BmMITE-13	nscaf3007	72998	72736	-
BmMITE-13	nscaf2832	52092	51830	-
BmMITE-13	nscaf2575	2143944	2143682	-
BmMITE-13	nscaf2575	574865	574602	-
BmMITE-13	nscaf2053	574565	574303	-
BmMITE-13	scaffold906	4219	3957	-
BmMITE-13	scaffold3445	779	518	-
BmMITE-13	nscaf1705	976516	976254	-
BmMITE-13	nscaf1681	1700674	1700413	-
BmMITE-13	nscaf1516	23114	22857	-
BmMITE-13	nscaf2674	719605	719341	-
BmMITE-13	nscaf3035	1769700	1769434	-
BmMITE-13	nscaf3089	685744	685481	-
BmMITE-13	nscaf3031	804054	803761	-
BmMITE-13	nscaf2210	3109543	3109286	-
BmMITE-13	nscaf2210	3093949	3093693	-
BmMITE-13	nscaf2210	1422497	1422234	-
BmMITE-13	nscaf2937	436831	436573	-
BmMITE-13	nscaf2511	567067	566807	-
BmMITE-13	nscaf3138	39916	39657	-
BmMITE-13	nscaf1071	324147	323884	-
BmMITE-13	nscaf2176	4180800	4180531	-
BmMITE-13	nscaf2176	352814	352554	-
BmMITE-13	scaffold13278	566	309	-
BmMITE-13	nscaf3093	257426	257164	-
BmMITE-13	nscaf2983	2942992	2942728	-
BmMITE-13	nscaf2983	2490901	2490637	-
BmMITE-13	nscaf2931	1606607	1606347	-
BmMITE-13	scaffold4309	733	471	-
BmMITE-13	nscaf3056	399962	399697	-
BmMITE-13	scaffold1420	4596	4332	-
BmMITE-13	nscaf2825	240740	240479	-

BmMITE-13	scaffold713	34953	34693	-
BmMITE-13	nscaf3015	889447	889226	-
BmMITE-13	nscaf2686	168097	168359	+
BmMITE-13	nscaf2330	3632582	3632846	+
BmMITE-13	nscaf2330	4280317	4280579	+
BmMITE-13	nscaf2330	4331897	4332162	+
BmMITE-13	nscaf3040	696458	696720	+
BmMITE-13	nscaf3040	905435	905696	+
BmMITE-13	nscaf2964	293146	293408	+
BmMITE-13	nscaf2964	1964837	1965093	+
BmMITE-13	nscaf2855	2979812	2980066	+
BmMITE-13	nscaf2855	4890134	4890391	+
BmMITE-13	nscaf2855	5130584	5130849	+
BmMITE-13	nscaf2855	5594833	5595095	+
BmMITE-13	nscaf2855	5633437	5633699	+
BmMITE-13	nscaf2847	7337345	7337606	+
BmMITE-13	nscaf2847	7923752	7924021	+
BmMITE-13	nscaf2829	1375927	1376189	+
BmMITE-13	nscaf2767	122701	122960	+
BmMITE-13	nscaf2767	1740992	1741254	+
BmMITE-13	nscaf2767	2154160	2154422	+
BmMITE-13	nscaf1898	5245083	5245343	+
BmMITE-13	nscaf1898	11721288	11721553	+
BmMITE-13	nscaf3034	653057	653318	+
BmMITE-13	nscaf3034	979957	980218	+
BmMITE-13	nscaf3034	1770195	1770461	+
BmMITE-13	nscaf3034	1925688	1925954	+
BmMITE-13	nscaf3027	4845907	4846167	+
BmMITE-13	nscaf2951	789912	790174	+
BmMITE-13	nscaf2780	237046	237308	+
BmMITE-13	nscaf2589	2623074	2623337	+
BmMITE-13	nscaf2589	3542164	3542431	+
BmMITE-13	nscaf2589	5010957	5011219	+
BmMITE-13	nscaf2529	4972567	4973050	+
BmMITE-13	nscaf2529	5088986	5089248	+
BmMITE-13	nscaf1690	3112536	3112800	+
BmMITE-13	nscaf3003	1594488	1594750	+
BmMITE-13	nscaf3003	4536618	4536874	+
BmMITE-13	nscaf2902	2769259	2769523	+
BmMITE-13	nscaf2902	7332496	7332760	+
BmMITE-13	nscaf2883	564474	564736	+
BmMITE-13	nscaf2883	705178	705440	+
BmMITE-13	nscaf2883	1008558	1008813	+

BmMITE-13	nscaf2876	1399798	1400060	+
BmMITE-13	nscaf2136	1432932	1433194	+
BmMITE-13	nscaf3058	765102	765359	+
BmMITE-13	nscaf3058	4944555	4944817	+
BmMITE-13	nscaf3058	6934855	6935118	+
BmMITE-13	nscaf3058	7404494	7404760	+
BmMITE-13	nscaf481	658812	659074	+
BmMITE-13	nscaf3099	726151	726413	+
BmMITE-13	nscaf3099	2225675	2225939	+
BmMITE-13	nscaf3099	4700441	4700706	+
BmMITE-13	nscaf2919	22524	22786	+
BmMITE-13	nscaf2888	2927721	2927975	+
BmMITE-13	nscaf2888	7267400	7267661	+
BmMITE-13	nscaf2853	2713922	2714179	+
BmMITE-13	nscaf2970	202421	202686	+
BmMITE-13	nscaf2655	3420373	3420636	+
BmMITE-13	nscaf2889	2133705	2133967	+
BmMITE-13	nscaf2575	1174348	1174603	+
BmMITE-13	scaffold40398	99	362	+
BmMITE-13	scaffold720	3545	3806	+
BmMITE-13	scaffold3800	146	407	+
BmMITE-13	nscaf3079	1490706	1490967	+
BmMITE-13	nscaf2823	977866	978128	+
BmMITE-13	nscaf2674	2836921	2837182	+
BmMITE-13	nscaf3035	1671200	1671466	+
BmMITE-13	nscaf3031	1530867	1531130	+
BmMITE-13	nscaf3075	668969	669231	+
BmMITE-13	nscaf2770	997610	997874	+
BmMITE-13	nscaf2511	5618901	5619164	+
BmMITE-13	scaffold4122	2	259	+
BmMITE-13	nscaf2885	5716	5977	+
BmMITE-13	nscaf3066	84206	84478	+
BmMITE-13	nscaf2882	17753	18015	+
BmMITE-13	nscaf3062	312815	313085	+
BmMITE-13	nscaf3098	1846365	1846623	+
BmMITE-13	nscaf3063	1237525	1237788	+
BmMITE-13	nscaf2818	1665049	1665306	+
BmMITE-13	nscaf2204	37736	37998	+
BmMITE-13	scaffold882	10318	10585	+
BmMITE-13	nscaf2838	889237	889499	+
BmMITE-13	nscaf1108	773186	773863	+
BmMITE-13	nscaf1987	36602	36876	+
BmMITE-13	nscaf2734	1719236	1719516	+

BmMITE-13	nscaf1108	1683234	1685071	+
BmMITE-13	nscaf1962	70372	70617	+
BmMITE-13	nscaf2176	825756	826006	+
BmMITE-13	nscaf2589	5784051	5784298	+
BmMITE-13	nscaf2589	5770640	5770868	+
BmMITE-14	nscaf3097	2459961	2459531	-
BmMITE-14	nscaf2847	975142	974702	-
BmMITE-14	nscaf2813	382381	381930	-
BmMITE-14	nscaf2204	4020253	4019369	-
BmMITE-14	nscaf2855	3903328	3902719	-
BmMITE-14	nscaf2204	1471218	1470840	-
BmMITE-14	nscaf2855	4909656	4909283	-
BmMITE-14	nscaf3074	481547	481174	-
BmMITE-14	nscaf2902	10787546	10787168	-
BmMITE-14	nscaf2865	5153339	5153781	+
BmMITE-14	nscaf2962	1090035	1090475	+
BmMITE-14	nscaf3079	318812	319254	+
BmMITE-14	nscaf3099	1038952	1039373	+
BmMITE-14	nscaf2962	655877	656247	+
BmMITE-14	nscaf3075	606862	607238	+
BmMITE-14	nscaf3075	647149	647527	+
BmMITE-14	nscaf2681	613168	613544	+
BmMITE-14	nscaf1108	591990	592358	+
BmMITE-14	nscaf2655	108992	109365	+
BmMITE-14	nscaf2902	6414910	6415279	+
BmMITE-14	nscaf2053	44837	45208	+
BmMITE-14	nscaf2891	1677326	1677671	+
BmMITE-15	nscaf1071	129794	130807	+
BmMITE-15	nscaf1690	4899025	4899402	-
BmMITE-15	nscaf2575	4252020	4252397	+
BmMITE-15	nscaf2655	896197	896574	+
BmMITE-15	nscaf2809	71341	72361	+
BmMITE-15	nscaf2853	5899987	5901009	-
BmMITE-15	nscaf2930	1317309	1317686	-
BmMITE-15	nscaf2998	133474	134503	-
BmMITE-15	nscaf3098	864409	867781	+
BmMITE-16	nscaf2815	2422460	2422169	-
BmMITE-16	nscaf2674	898198	897912	-
BmMITE-16	nscaf2823	1530336	1530051	-
BmMITE-16	nscaf3031	2893662	2893376	-

BmMITE-16	nscaf2511	4685897	4685611	-
BmMITE-16	nscaf3162	28764	27738	-
BmMITE-16	nscaf3026	3752670	3752242	-
BmMITE-16	nscaf3031	354315	354179	-
BmMITE-16	nscaf2916	60686	60197	-
BmMITE-16	nscaf2902	78401	78003	-
BmMITE-16	nscaf2986	4960522	4960282	-
BmMITE-16	nscaf1898	2516455	2516719	+
BmMITE-16	nscaf2818	1127168	1127725	+
BmMITE-16	nscaf1898	15391039	15391587	+
BmMITE-16	nscaf2789	1205967	1206537	+
BmMITE-16	nscaf2210	1350346	1350903	+
BmMITE-16	nscaf3026	6548794	6549079	+
BmMITE-16	nscaf3015	4516344	4516631	+
BmMITE-16	nscaf2828	2201913	2202198	+
BmMITE-16	nscaf3013	771538	771825	+
BmMITE-16	nscaf2136	3043846	3044134	+
BmMITE-16	nscaf2852	2379906	2380187	+
BmMITE-16	nscaf2855	4746276	4746578	+
BmMITE-16	scaffold672	11377	11666	+
BmMITE-16	nscaf2511	3155219	3155451	+
BmMITE-17	nscaf2977	73006	72521	-
BmMITE-17	nscaf2828	3949045	3948541	-
BmMITE-17	nscaf2930	1804860	1804375	-
BmMITE-17	nscaf2789	1229988	1229505	-
BmMITE-17	nscaf2891	2325788	2325297	-
BmMITE-17	nscaf3048	717475	716999	-
BmMITE-17	nscaf3063	2214006	2213513	-
BmMITE-17	nscaf2964	1891303	1890842	-
BmMITE-17	nscaf2853	5266867	5266377	-
BmMITE-17	nscaf2891	2566816	2566364	-
BmMITE-17	nscaf2905	327458	326977	-
BmMITE-17	nscaf2053	427659	427178	-
BmMITE-17	nscaf2204	1451907	1451439	-
BmMITE-17	nscaf3003	491161	491654	+
BmMITE-17	nscaf2983	1391749	1392237	+
BmMITE-17	nscaf1705	1053629	1054116	+
BmMITE-17	nscaf2888	1766871	1767357	+
BmMITE-17	nscaf2887	1190641	1191127	+
BmMITE-17	nscaf3055	482337	482799	+
BmMITE-17	nscaf2993	7542766	7543230	+
BmMITE-17	nscaf2589	3913639	3914129	+

BmMITE-17	nscaf1898	12540615	12541450	+

	Fragmentary copies			
**Family**	**Nscaf**	**start**	**end**	**strand**
BmMITE-1	nscaf2330	4100955	4100701	-
BmMITE-1	nscaf2902	4766319	4766061	-
BmMITE-1	nscaf2655	352949	352695	-
BmMITE-1	nscaf2855	5076958	5076412	-
BmMITE-1	nscaf3097	299574	299827	+
BmMITE-1	nscaf2176	3047376	3047625	+
BmMITE-1	nscaf2176	2642687	2642433	-
BmMITE-1	nscaf3040	3582297	3582043	-
BmMITE-1	nscaf2789	1148804	1148551	-
BmMITE-1	nscaf2836	526094	526347	+
BmMITE-1	nscaf2852	1397665	1397401	-
BmMITE-1	nscaf2829	4121531	4121787	+
BmMITE-1	nscaf2986	2777697	2777951	+
BmMITE-1	nscaf3040	966984	967253	+
BmMITE-1	nscaf2953	2241696	2241949	+
BmMITE-1	nscaf3075	563878	563625	-
BmMITE-1	nscaf2853	3909877	3909622	-
BmMITE-1	nscaf2789	140150	139893	-
BmMITE-1	nscaf3070	32123	31871	-
BmMITE-1	nscaf2889	3622381	3622627	+
BmMITE-1	nscaf3098	494083	493838	-
BmMITE-1	nscaf3097	318582	318328	-
BmMITE-1	nscaf2511	3648053	3648307	+
BmMITE-1	nscaf3058	3324869	3325122	+
BmMITE-1	nscaf2527	753	975	+
BmMITE-1	nscaf2979	104524	104271	-
BmMITE-1	nscaf2136	2673275	2673023	-
BmMITE-1	nscaf2855	1123296	1123549	+
BmMITE-1	nscaf2930	4836967	4837223	+
BmMITE-1	nscaf3040	223673	223925	+
BmMITE-1	nscaf2511	5037573	5037797	+
BmMITE-1	nscaf2823	1007573	1007323	-
BmMITE-1	nscaf2986	5668557	5668803	+
BmMITE-1	nscaf2855	2411902	2412153	+
BmMITE-1	nscaf1681	1031906	1032154	+
BmMITE-1	nscaf3097	121118	120865	-
BmMITE-1	nscaf2589	5148146	5148393	+
BmMITE-1	nscaf2931	1031784	1031538	-
BmMITE-1	nscaf3034	2146917	2147162	+
BmMITE-1	nscaf2779	6638	6877	+

BmMITE-1	nscaf3022	642751	642995	+
BmMITE-1	nscaf2865	5151457	5151705	+
BmMITE-1	nscaf2986	4360561	4360313	-
BmMITE-1	nscaf2902	906500	906251	-
BmMITE-1	nscaf2655	1680266	1680514	+
BmMITE-1	nscaf2829	955989	956236	+
BmMITE-1	nscaf2888	6196872	6196621	-
BmMITE-1	nscaf2836	94162	94507	+
BmMITE-1	nscaf2931	1301315	1301069	-
BmMITE-1	nscaf2964	3524745	3525419	+
BmMITE-1	nscaf2865	2312123	2311875	-
BmMITE-1	nscaf2902	10448794	10449047	+
BmMITE-1	nscaf2734	1337716	1337964	+
BmMITE-1	nscaf3015	1802418	1802664	+
BmMITE-1	nscaf3058	2620383	2620133	-
BmMITE-1	scaffold1241	3657	3407	-
BmMITE-1	nscaf463	1227642	1227395	-
BmMITE-1	nscaf1108	2120753	2120996	+
BmMITE-1	nscaf2827	1267132	1266887	-
BmMITE-1	nscaf2930	3259498	3258981	-
BmMITE-1	nscaf3058	4296922	4296679	-
BmMITE-1	nscaf1690	6992897	6993142	+
BmMITE-1	nscaf3058	4820226	4819983	-
BmMITE-1	nscaf2813	704156	703913	-
BmMITE-1	nscaf3058	3862751	3862990	+
BmMITE-1	nscaf2860	986624	986861	+
BmMITE-1	nscaf3099	3151398	3151161	-
BmMITE-1	nscaf3056	780678	780443	-
BmMITE-1	nscaf2943	858114	858337	+
BmMITE-1	nscaf2828	5105187	5104965	-
BmMITE-1	nscaf2948	1411444	1411664	+
BmMITE-1	nscaf3003	2344203	2344427	+
BmMITE-1	nscaf3026	6502231	6501549	-
BmMITE-1	nscaf3072	825009	825267	+
BmMITE-1	nscaf2800	1840188	1840442	+
BmMITE-1	nscaf2800	2769000	2768746	-
BmMITE-1	nscaf3097	1734486	1734232	-
BmMITE-1	nscaf2589	2899054	2898800	-
BmMITE-1	nscaf2511	3854364	3854109	-
BmMITE-1	nscaf2511	2944983	2944728	-
BmMITE-1	nscaf3099	3360709	3360964	+
BmMITE-1	nscaf3078	3707	3453	-
BmMITE-1	nscaf2216	1151954	1152202	+

BmMITE-1	nscaf3005	2076168	2075914	-
BmMITE-1	nscaf2204	1478787	1479041	+
BmMITE-1	nscaf1898	15207766	15207514	-
BmMITE-1	nscaf2589	3863958	3864212	+
BmMITE-1	nscaf2993	4814968	4814713	-
BmMITE-1	nscaf2136	4849079	4848824	-
BmMITE-1	nscaf2589	3733784	3733530	-
BmMITE-1	nscaf2511	2423350	2423604	+
BmMITE-1	nscaf3055	1101116	1101602	+
BmMITE-1	nscaf2995	294216	293962	-
BmMITE-1	nscaf2330	932834	933085	+
BmMITE-1	nscaf1681	1128772	1128519	-
BmMITE-1	nscaf2818	1576890	1576636	-
BmMITE-1	nscaf2829	2224918	2225423	+
BmMITE-1	nscaf2902	397304	397557	+
BmMITE-1	nscaf2993	5489590	5489328	-
BmMITE-1	nscaf2993	7023437	7023691	+
BmMITE-1	nscaf2827	2200587	2200336	-
BmMITE-1	nscaf3003	2626264	2626516	+
BmMITE-1	nscaf2825	493674	493926	+
BmMITE-1	nscaf3075	1068681	1068429	-
BmMITE-1	nscaf3034	3574426	3574677	+
BmMITE-1	nscaf2943	964910	965159	+
BmMITE-1	nscaf2889	2847380	2847141	-
BmMITE-1	nscaf2847	4622902	4623144	+
BmMITE-1	nscaf2855	2077736	2077981	+
BmMITE-1	nscaf1108	1396627	1396383	-
BmMITE-1	nscaf2575	2340479	2340702	+
BmMITE-1	nscaf3072	2421406	2421629	+
BmMITE-1	nscaf2970	1573740	1573964	+
BmMITE-1	nscaf481	1362861	1362637	-
BmMITE-1	nscaf3013	518752	518975	+
BmMITE-1	nscaf2902	6357309	6357087	-
BmMITE-2	nscaf3003	1899139	1899415	+
BmMITE-2	nscaf2931	316005	316280	+
BmMITE-2	nscaf2887	1746767	1747042	+
BmMITE-2	nscaf2781	46712	46993	+
BmMITE-2	nscaf2767	3751922	3751646	-
BmMITE-2	nscaf2529	4737282	4737006	-
BmMITE-2	nscaf2330	3613710	3613434	-
BmMITE-2	nscaf2888	7971386	7971662	+
BmMITE-2	nscaf2953	1899002	1899278	+

BmMITE-2	nscaf3099	280405	280681	+
BmMITE-2	nscaf3099	3103925	3103654	-
BmMITE-2	nscaf3099	1921328	1921052	-
BmMITE-2	nscaf3058	8149989	8149714	-
BmMITE-2	nscaf3058	3028734	3028458	-
BmMITE-2	nscaf3035	342984	345064	+
BmMITE-2	nscaf3032	885617	885893	+
BmMITE-2	nscaf3031	3478115	3477840	-
BmMITE-2	nscaf3026	4363607	4363331	-
BmMITE-2	nscaf2902	291137	291413	+
BmMITE-2	nscaf2902	2385098	2385374	+
BmMITE-2	nscaf2902	3458591	3458867	+
BmMITE-2	nscaf2902	340751	340475	-
BmMITE-2	nscaf2876	1549333	1549057	-
BmMITE-2	nscaf2855	6784757	6785033	+
BmMITE-2	nscaf2855	3459830	3459554	-
BmMITE-2	nscaf2829	113126	113402	+
BmMITE-2	nscaf2829	391286	391562	+
BmMITE-2	nscaf2829	2817534	2817260	-
BmMITE-2	nscaf2828	5602071	5602347	+
BmMITE-2	nscaf2823	2977294	2977571	+
BmMITE-2	nscaf2823	4062952	4063228	+
BmMITE-2	nscaf2795	108825	109100	+
BmMITE-2	nscaf2795	3789305	3789581	+
BmMITE-2	nscaf2770	25786	25510	-
BmMITE-2	nscaf2655	534794	535070	+
BmMITE-2	nscaf2511	4282915	4283191	+
BmMITE-2	nscaf2204	193565	193841	+
BmMITE-2	nscaf2136	7598990	7599266	+
BmMITE-2	nscaf2136	5435667	5435391	-
BmMITE-2	nscaf1898	4548607	4548883	+
BmMITE-2	nscaf1898	8953013	8953289	+
BmMITE-2	nscaf1690	588240	588515	+
BmMITE-2	nscaf3072	2983346	2983622	+
BmMITE-2	nscaf3063	818529	818253	-
BmMITE-2	nscaf3055	1429546	1429270	-
BmMITE-2	nscaf3040	981441	981122	-
BmMITE-2	nscaf3027	5027312	5027587	+
BmMITE-2	nscaf2993	7714949	7714673	-
BmMITE-2	nscaf2993	1187419	1186668	-
BmMITE-2	nscaf2883	1070734	1070458	-
BmMITE-2	nscaf2801	525000	524724	-
BmMITE-2	nscaf2797	554600	554876	+

BmMITE-2	nscaf2674	1216920	1217196	+
BmMITE-2	nscaf2674	793895	793618	-
BmMITE-2	nscaf2916	672052	672328	+
BmMITE-2	nscaf2838	2413413	2413137	-
BmMITE-2	nscaf2836	386045	386321	+
BmMITE-2	nscaf2216	1502782	1503058	+
BmMITE-2	scaffold584	42425	42701	+
BmMITE-2	nscaf3131	14733	14457	-
BmMITE-2	nscaf2556	309750	310026	+
BmMITE-2	nscaf2360	4854	5130	+
BmMITE-2	nscaf2962	1024112	1023836	-
BmMITE-2	nscaf2766	48809	48533	-
BmMITE-2	nscaf2529	5268777	5268503	-
BmMITE-2	nscaf2330	2155069	2155343	+
BmMITE-2	nscaf2655	179996	180269	+
BmMITE-2	nscaf2888	8998849	8995339	-
BmMITE-2	nscaf3034	2309859	2310133	+
BmMITE-2	nscaf3035	1447828	1448092	+
BmMITE-2	nscaf2986	3970403	3970678	+
BmMITE-2	nscaf2931	991423	991698	+
BmMITE-2	nscaf2847	7125735	7125460	-
BmMITE-2	nscaf2767	1769871	1769596	-
BmMITE-2	nscaf2529	5780431	5780156	-
BmMITE-2	nscaf2330	2036975	2037251	+
BmMITE-2	nscaf2053	105248	105523	+
BmMITE-2	nscaf2053	245841	245566	-
BmMITE-2	nscaf1108	368797	369072	+
BmMITE-2	nscaf1071	296226	295952	-
BmMITE-2	nscaf2888	997712	997987	+
BmMITE-2	nscaf2888	2215759	2216034	+
BmMITE-2	nscaf2865	3832289	3832564	+
BmMITE-2	nscaf2575	2290238	2290513	+
BmMITE-2	nscaf2575	2617294	2617020	-
BmMITE-2	nscaf3031	2287884	2288159	+
BmMITE-2	nscaf3031	3094945	3094670	-
BmMITE-2	nscaf3026	1736362	1736636	+
BmMITE-2	nscaf3026	6559596	6559322	-
BmMITE-2	nscaf2998	505189	504914	-
BmMITE-2	nscaf2983	1427386	1427661	+
BmMITE-2	nscaf2964	4554739	4555014	+
BmMITE-2	nscaf2964	3784858	3784582	-
BmMITE-2	nscaf2964	1470074	1469800	-
BmMITE-2	nscaf2943	1939122	1939397	+

BmMITE-2	nscaf2902	6005697	6005422	-
BmMITE-2	nscaf2828	983018	982743	-
BmMITE-2	nscaf2822	598355	598630	+
BmMITE-2	nscaf2800	1693179	1693455	+
BmMITE-2	nscaf2800	655726	655451	-
BmMITE-2	nscaf2655	359770	359495	-
BmMITE-2	nscaf2589	3856663	3856938	+
BmMITE-2	nscaf2511	5622634	5622359	-
BmMITE-2	nscaf2511	4457014	4456740	-
BmMITE-2	nscaf2204	2946755	2946480	-
BmMITE-2	nscaf2176	1601687	1601962	+
BmMITE-2	nscaf2176	3035427	3035155	-
BmMITE-2	nscaf2136	413648	413922	+
BmMITE-2	nscaf1898	6446197	6446472	+
BmMITE-2	nscaf1898	7700092	7700367	+
BmMITE-2	nscaf1898	7940126	7940401	+
BmMITE-2	nscaf1681	1587424	1587149	-
BmMITE-2	nscaf3034	2773860	2772478	-
BmMITE-2	nscaf3027	5002008	5001733	-
BmMITE-2	nscaf3027	4318335	4318060	-
BmMITE-2	nscaf2993	1470308	1470033	-
BmMITE-2	nscaf2833	571163	570888	-
BmMITE-2	nscaf2836	453688	453962	+
BmMITE-2	nscaf2686	1046056	1045781	-
BmMITE-2	nscaf2216	1172015	1171740	-
BmMITE-2	nscaf716	35894	36169	+
BmMITE-2	nscaf2819	129490	129755	+
BmMITE-2	nscaf3084	5001	4726	-
BmMITE-2	nscaf3091	1	274	+
BmMITE-2	nscaf2855	4676033	4675759	-
BmMITE-2	nscaf2847	5429095	5429367	+
BmMITE-2	nscaf2888	3403621	3403358	-
BmMITE-2	nscaf2842	1532988	1532712	-
BmMITE-2	nscaf1071	220485	220208	-
BmMITE-2	nscaf3131	59758	60017	+
BmMITE-2	nscaf2795	1380147	1376444	-
BmMITE-2	nscaf1898	2228774	2228526	-
BmMITE-2	nscaf2983	1973331	1973107	-
BmMITE-2	nscaf2529	2340064	2340338	+
BmMITE-2	nscaf2330	748886	748604	-
BmMITE-2	nscaf2888	8151504	8151230	-
BmMITE-2	nscaf2998	29013	29287	+
BmMITE-2	nscaf2964	1032534	1032808	+

BmMITE-2	nscaf2964	4565024	4565297	+
BmMITE-2	nscaf2902	9729239	9728965	-
BmMITE-2	nscaf2204	3111587	3111861	+
BmMITE-2	nscaf2993	297411	297685	+
BmMITE-2	nscaf3079	1602833	1603107	+
BmMITE-2	scaffold1494	800	526	-
BmMITE-2	nscaf2913	59930	59656	-
BmMITE-2	nscaf2912	696193	695920	-
BmMITE-2	nscaf2902	9526624	9526399	-
BmMITE-2	nscaf2931	1299316	1299044	-
BmMITE-2	nscaf2930	2658282	2658010	-
BmMITE-2	nscaf2330	4271568	4271845	+
BmMITE-2	nscaf1108	1851321	1851048	-
BmMITE-2	nscaf481	139334	139061	-
BmMITE-2	nscaf3058	4529245	4528972	-
BmMITE-2	nscaf3026	951661	951388	-
BmMITE-2	nscaf2951	1305569	1305841	+
BmMITE-2	nscaf2903	1566487	1566760	+
BmMITE-2	nscaf2828	3033199	3033472	+
BmMITE-2	nscaf1898	7624594	7624321	-
BmMITE-2	nscaf1898	4265623	4265350	-
BmMITE-2	nscaf3040	4240998	4240725	-
BmMITE-2	nscaf2852	350881	350608	-
BmMITE-2	nscaf2797	1202742	1202469	-
BmMITE-2	nscaf2851	1724262	1723989	-
BmMITE-2	nscaf2888	7504108	7504381	+
BmMITE-2	nscaf3027	830569	830297	-
BmMITE-2	nscaf2993	7568791	7569064	+
BmMITE-2	nscaf2855	6536744	6536475	-
BmMITE-2	nscaf3010	15331	15060	-
BmMITE-2	nscaf1681	4920332	4920061	-
BmMITE-2	nscaf2912	1410771	1411044	+
BmMITE-2	nscaf2815	1447810	1448045	+
BmMITE-2	nscaf2575	2424129	2424399	+
BmMITE-2	nscaf2993	854388	854657	+
BmMITE-2	nscaf2575	1583076	1582807	-
BmMITE-2	nscaf2204	2006462	2006194	-
BmMITE-2	nscaf1681	2402472	2402203	-
BmMITE-2	nscaf98	138270	138538	+
BmMITE-2	nscaf3015	4522597	4522865	+
BmMITE-2	nscaf2993	3995173	3995443	+
BmMITE-2	nscaf3045	266725	266992	+
BmMITE-2	nscaf3026	2643087	2643355	+

BmMITE-2	nscaf2589	1167637	1167905	+
BmMITE-2	nscaf2990	188319	188014	-
BmMITE-2	nscaf2924	46326	46064	-
BmMITE-2	nscaf2930	5471446	5471713	+
BmMITE-2	nscaf2827	1618681	1618948	+
BmMITE-2	nscaf2823	2859896	2859629	-
BmMITE-2	nscaf2890	708086	708353	+
BmMITE-2	nscaf2686	996354	996918	+
BmMITE-2	nscaf2511	5186226	5186492	+
BmMITE-2	nscaf1806	29933	30199	+
BmMITE-2	nscaf1108	863631	863896	+
BmMITE-2	nscaf2903	1008466	1008731	+
BmMITE-2	nscaf1681	4583507	4583772	+
BmMITE-2	nscaf3040	2756960	2756695	-
BmMITE-2	nscaf3005	2179047	2178782	-
BmMITE-2	nscaf2176	3108410	3108146	-
BmMITE-2	nscaf2847	8574220	8574487	+
BmMITE-2	scaffold10013	403	666	+
BmMITE-2	scaffold9350	777	514	-
BmMITE-2	nscaf2796	107702	107443	-
BmMITE-2	nscaf2902	3908954	3909215	+
BmMITE-2	nscaf2828	1219778	1219516	-
BmMITE-2	nscaf2883	1418773	1419036	+
BmMITE-2	nscaf2937	784948	785208	+
BmMITE-2	nscaf2951	1551649	1551910	+
BmMITE-2	nscaf2511	1164740	1164479	-
BmMITE-2	nscaf2797	1049646	1049907	+
BmMITE-2	nscaf2176	2909127	2908873	-
BmMITE-2	nscaf2770	1138046	1138304	+
BmMITE-2	nscaf2887	1948192	1948448	+
BmMITE-2	nscaf2674	2878317	2878572	+
BmMITE-2	nscaf2815	454441	454696	+
BmMITE-2	nscaf3052	200228	200486	+
BmMITE-2	nscaf1690	1710379	1710127	-
BmMITE-2	nscaf2828	5019824	5019575	-
BmMITE-2	nscaf2931	748335	748087	-
BmMITE-2	nscaf3045	432149	431900	-
BmMITE-2	nscaf2865	4204818	4204570	-
BmMITE-2	nscaf3072	2564472	2564224	-
BmMITE-2	nscaf2993	4469199	4468951	-
BmMITE-2	nscaf1962	126777	126529	-
BmMITE-2	nscaf3058	4158244	4157997	-
BmMITE-2	nscaf2842	1365205	1364958	-

BmMITE-2	nscaf3034	3426544	3426791	+
BmMITE-2	nscaf3003	2609028	2609274	+
BmMITE-2	nscaf2176	2737698	2737944	+
BmMITE-2	nscaf2556	469564	469808	+
BmMITE-2	nscaf3040	1219975	1220214	+
BmMITE-2	nscaf2930	2958434	2958672	+
BmMITE-2	nscaf2853	246606	246369	-
BmMITE-2	nscaf2655	3624953	3625185	+
BmMITE-2	nscaf2847	29889	29662	-
BmMITE-2	nscaf3026	2128047	2128278	+
BmMITE-2	nscaf3032	2117482	2117254	-
BmMITE-2	nscaf3055	1587776	1588000	+
BmMITE-2	nscaf2912	1228884	1228661	-
BmMITE-2	nscaf3003	1601765	1602041	+
BmMITE-2	nscaf3003	3950678	3950954	+
BmMITE-2	nscaf2986	2886385	2886109	-
BmMITE-2	nscaf2930	2586887	2586611	-
BmMITE-2	nscaf2818	1211743	1212019	+
BmMITE-2	nscaf2818	3126394	3126670	+
BmMITE-2	nscaf2818	2341336	2341060	-
BmMITE-2	nscaf2818	1090142	1089866	-
BmMITE-2	nscaf2818	960584	960308	-
BmMITE-2	nscaf2529	315154	315430	+
BmMITE-2	nscaf2053	304593	304869	+
BmMITE-2	nscaf2053	357392	357669	+
BmMITE-2	scaffold24948	619	343	-
BmMITE-2	nscaf2888	947589	947865	+
BmMITE-2	nscaf2888	8003119	8003395	+
BmMITE-2	nscaf2888	2210412	2210136	-
BmMITE-2	nscaf2865	650659	650935	+
BmMITE-2	nscaf2865	2267483	2267759	+
BmMITE-2	nscaf2865	3317100	3316824	-
BmMITE-2	nscaf3035	1613611	1613887	+
BmMITE-2	nscaf3031	624883	624607	-
BmMITE-2	nscaf2964	3720906	3721183	+
BmMITE-2	nscaf2964	4524430	4524154	-
BmMITE-2	nscaf2943	2095619	2095343	-
BmMITE-2	nscaf2927	34928	34652	-
BmMITE-2	nscaf2902	2652413	2652137	-
BmMITE-2	nscaf2855	2622158	2621882	-
BmMITE-2	nscaf2853	2154859	2155135	+
BmMITE-2	nscaf2828	5759579	5759854	+
BmMITE-2	nscaf2823	520441	520717	+

BmMITE-2	nscaf2800	1726338	1726062	-
BmMITE-2	nscaf2770	976151	976427	+
BmMITE-2	nscaf2770	1100930	1101206	+
BmMITE-2	nscaf2589	3251301	3251025	-
BmMITE-2	nscaf2511	3323218	3323494	+
BmMITE-2	nscaf2204	4006089	4006364	+
BmMITE-2	nscaf2204	4384078	4383801	-
BmMITE-2	nscaf2136	1188001	1188277	+
BmMITE-2	nscaf1898	11432139	11432414	+
BmMITE-2	nscaf1681	1913530	1913806	+
BmMITE-2	nscaf1681	901748	901472	-
BmMITE-2	nscaf3034	3906660	3906385	-
BmMITE-2	nscaf2948	2732314	2732590	+
BmMITE-2	nscaf463	817191	817467	+
BmMITE-2	nscaf3075	1155626	1155902	+
BmMITE-2	nscaf3041	1070798	1070522	-
BmMITE-2	nscaf3027	443547	443821	+
BmMITE-2	nscaf3027	2902134	2901858	-
BmMITE-2	nscaf3008	1932599	1932325	-
BmMITE-2	nscaf3008	696643	696367	-
BmMITE-2	nscaf2852	325789	325513	-
BmMITE-2	nscaf2833	322855	323135	+
BmMITE-2	nscaf2797	927765	928041	+
BmMITE-2	nscaf2792	9634	9910	+
BmMITE-2	nscaf2674	6633014	6633290	+
BmMITE-2	nscaf2210	1938303	1938579	+
BmMITE-2	scaffold1358	5908	5632	-
BmMITE-2	nscaf2937	1270290	1270014	-
BmMITE-2	nscaf2937	543349	543073	-
BmMITE-2	nscaf3044	1363307	1363032	-
BmMITE-2	nscaf2899	651524	651248	-
BmMITE-2	nscaf2891	684245	684511	+
BmMITE-2	nscaf2836	460449	460174	-
BmMITE-2	nscaf3066	186291	186572	+
BmMITE-2	nscaf3066	1029456	1029180	-
BmMITE-2	nscaf2912	399574	399850	+
BmMITE-2	nscaf2889	735544	735820	+
BmMITE-2	nscaf2793	36950	37225	+
BmMITE-2	nscaf3101	88555	88831	+
BmMITE-2	nscaf2210	819062	819337	+
BmMITE-2	nscaf1108	1198156	1197881	-
BmMITE-2	nscaf2888	3627571	3627843	+
BmMITE-2	nscaf2888	7579300	7579025	-

BmMITE-2	nscaf2575	2131046	2131321	+
BmMITE-2	nscaf481	744514	744789	+
BmMITE-2	nscaf3031	4538609	4538334	-
BmMITE-2	nscaf3031	507067	506792	-
BmMITE-2	nscaf2964	350356	350630	+
BmMITE-2	nscaf2943	1388130	1387856	-
BmMITE-2	nscaf2943	366885	366610	-
BmMITE-2	nscaf2902	1546429	1546704	+
BmMITE-2	nscaf2902	9747457	9747732	+
BmMITE-2	nscaf2795	2608943	2608669	-
BmMITE-2	nscaf1898	2927786	2927512	-
BmMITE-2	nscaf1690	3666655	3666380	-
BmMITE-2	nscaf2993	2930128	2929854	-
BmMITE-2	nscaf2883	544546	544272	-
BmMITE-2	nscaf2852	1640527	1640253	-
BmMITE-2	nscaf2902	1310936	1311177	+
BmMITE-2	nscaf2887	730645	730371	-
BmMITE-2	nscaf2623	294364	294638	+
BmMITE-2	nscaf2953	748173	748451	+
BmMITE-2	nscaf2953	714800	714526	-
BmMITE-2	nscaf2943	1965394	1965120	-
BmMITE-2	nscaf2855	4523265	4522993	-
BmMITE-2	nscaf2204	2667020	2667294	+
BmMITE-2	nscaf3015	2241903	2242177	+
BmMITE-2	nscaf2970	2253477	2253201	-
BmMITE-2	nscaf2970	1206488	1206213	-
BmMITE-2	nscaf2852	60837	61111	+
BmMITE-2	nscaf2734	1731783	1732057	+
BmMITE-2	nscaf2886	1111252	1111525	+
BmMITE-2	nscaf2827	1467350	1467623	+
BmMITE-2	nscaf2888	768578	768851	+
BmMITE-2	nscaf2953	1102769	1102496	-
BmMITE-2	nscaf3097	431607	431879	+
BmMITE-2	nscaf3062	623569	623298	-
BmMITE-2	nscaf3058	7529960	7529687	-
BmMITE-2	nscaf2998	879444	879721	+
BmMITE-2	nscaf2901	1446102	1445829	-
BmMITE-2	nscaf2860	2183622	2183895	+
BmMITE-2	nscaf2829	1699763	1699491	-
BmMITE-2	nscaf2822	1745361	1745634	+
BmMITE-2	nscaf2770	1015951	1015678	-
BmMITE-2	nscaf2770	707621	707349	-
BmMITE-2	nscaf2511	4563237	4563510	+

BmMITE-2	nscaf2176	2502735	2503008	+
BmMITE-2	nscaf2993	205911	206184	+
BmMITE-2	nscaf2970	638699	638426	-
BmMITE-2	nscaf2797	992518	992791	+
BmMITE-2	nscaf125	117635	117908	+
BmMITE-2	nscaf2828	2169799	2170067	+
BmMITE-2	nscaf2330	1596430	1596700	+
BmMITE-2	nscaf2176	833732	833463	-
BmMITE-2	nscaf2851	334276	334008	-
BmMITE-2	nscaf2815	567400	567130	-
BmMITE-2	nscaf2912	348015	347745	-
BmMITE-2	scaffold8113	271	1	-
BmMITE-2	nscaf2930	2287210	2286942	-
BmMITE-2	nscaf2847	2965445	2965712	+
BmMITE-2	nscaf2767	3309606	3309339	-
BmMITE-2	nscaf2529	791897	792164	+
BmMITE-2	nscaf2852	33717	33450	-
BmMITE-2	nscaf2891	1551696	1551962	+
BmMITE-2	nscaf2686	799909	799637	-
BmMITE-2	nscaf2136	323221	322955	-
BmMITE-2	nscaf3097	902522	902787	+
BmMITE-2	nscaf3035	516140	515875	-
BmMITE-2	nscaf2901	1532677	1532412	-
BmMITE-2	nscaf2993	5187612	5187877	+
BmMITE-2	nscaf1898	9261792	9261538	-
BmMITE-2	nscaf2759	296680	296408	-
BmMITE-2	nscaf1108	780234	780499	+
BmMITE-2	nscaf3027	69806	69544	-
BmMITE-2	nscaf3075	1066539	1066281	-
BmMITE-2	nscaf2953	1322695	1323830	+
BmMITE-2	nscaf3035	1696327	1696072	-
BmMITE-2	nscaf2902	5427290	5427030	-
BmMITE-2	nscaf1690	6321360	6321615	+
BmMITE-2	nscaf2855	4613261	4613007	-
BmMITE-2	nscaf2589	556217	555968	-
BmMITE-2	nscaf2674	4234901	4234648	-
BmMITE-2	nscaf3045	1356350	1356604	+
BmMITE-2	nscaf2931	1713112	1712861	-
BmMITE-2	nscaf2865	3875563	3875818	+
BmMITE-2	nscaf3097	3191750	3191496	-
BmMITE-2	nscaf2964	4460290	4460542	+
BmMITE-2	nscaf2204	1208399	1208652	+
BmMITE-2	nscaf2964	3456856	3456605	-

BmMITE-2	nscaf2986	4372540	4372312	-
BmMITE-2	nscaf2853	2193510	2193737	+
BmMITE-2	nscaf2842	332712	332938	+
BmMITE-2	nscaf2204	4028201	4028429	+
BmMITE-2	nscaf2204	1495448	1495220	-
BmMITE-2	nscaf1690	622510	622737	+
BmMITE-2	nscaf3063	2124578	2124806	+
BmMITE-2	nscaf3027	1507740	1507512	-
BmMITE-2	nscaf2883	1969632	1969860	+
BmMITE-2	nscaf2874	73264	73492	+
BmMITE-2	nscaf1289	83618	83846	+
BmMITE-2	nscaf2886	758741	758967	+
BmMITE-2	nscaf1108	688585	688811	+
BmMITE-2	nscaf3058	6767610	6767384	-
BmMITE-2	nscaf2983	2733686	2733460	-
BmMITE-2	nscaf2902	5191492	5191718	+
BmMITE-2	nscaf2795	1255543	1255769	+
BmMITE-2	nscaf1898	11002684	11002458	-
BmMITE-2	nscaf1690	7425866	7426092	+
BmMITE-2	nscaf2970	1366910	1366684	-
BmMITE-2	nscaf2838	296372	296146	-
BmMITE-2	nscaf2889	1504844	1504618	-
BmMITE-2	nscaf2674	1738674	1738449	-

BmMITE-3	3	306	nscaf3031	3418522	3418054
BmMITE-3	4	306	nscaf2781	163665	163364
BmMITE-3	13	306	nscaf2888	9518983	9519442
BmMITE-3	16	306	nscaf3031	4755653	4755219
BmMITE-3	16	306	nscaf2767	3554392	3554833
BmMITE-3	1	304	nscaf3053	602626	602166
BmMITE-3	1	303	nscaf2912	655591	656067
BmMITE-3	1	303	nscaf3034	2011244	2011720
BmMITE-3	1	294	nscaf2781	73467	74514
BmMITE-3	1	290	nscaf2529	5720890	5720601
BmMITE-3	1	290	nscaf3058	3549700	3550138
BmMITE-3	1	290	nscaf3005	2242404	2241964

BmMITE-4	11	567	nscaf2822	347932	348723
BmMITE-4	16	555	nscaf3032	35987	36546
BmMITE-4	22	567	scaffold348	1	566
BmMITE-4	61	567	scaffold295	1	527
BmMITE-4	94	567	scaffold299	1	477
BmMITE-4	95	567	nscaf3035	28198	28636

BmMITE-4	105	565	scaffold164	1	461
BmMITE-4	1	532	scaffold337	46	577
BmMITE-4	1	505	nscaf2948	2574444	2574948
BmMITE-5	2	213	nscaf2204	961814	962024
BmMITE-5	2	213	nscaf2930	4814955	4815158
BmMITE-5	2	213	nscaf2136	4179453	4179663
BmMITE-5	2	213	nscaf2971	97165	97375
BmMITE-5	3	213	nscaf2860	2122186	2121976
BmMITE-5	3	213	nscaf2511	779111	778902
BmMITE-5	3	213	nscaf3058	4957372	4958024
BmMITE-5	4	213	nscaf2930	2625781	2625573
BmMITE-5	4	213	nscaf2852	1695069	1695277
BmMITE-5	4	213	scaffold433	29	237
BmMITE-5	4	213	nscaf3048	812828	813036
BmMITE-5	4	213	nscaf2891	2656529	2656321
BmMITE-5	4	213	nscaf2529	4426624	4426298
BmMITE-5	5	213	nscaf2943	759865	760072
BmMITE-5	5	213	nscaf3003	368078	367871
BmMITE-5	5	213	nscaf3072	1706723	1709546
BmMITE-5	8	213	nscaf2964	3783983	3785234
BmMITE-5	13	213	nscaf2888	2776663	2776464
BmMITE-5	18	213	nscaf2847	1657642	1657448
BmMITE-5	18	210	nscaf2823	34370	34555
BmMITE-5	20	213	nscaf2827	830196	830003
BmMITE-5	20	213	nscaf2833	175425	175233
BmMITE-5	24	213	nscaf2053	364557	364745
BmMITE-5	28	213	nscaf3052	997828	997644
BmMITE-5	29	213	nscaf2852	1525504	1525688
BmMITE-5	32	213	nscaf2828	5971682	5971071
BmMITE-5	43	213	nscaf3045	1141254	1141423
BmMITE-5	1	212	nscaf2136	2805711	2805921
BmMITE-5	1	212	nscaf2818	1133381	1133590
BmMITE-5	1	212	nscaf1962	478455	479107
BmMITE-5	1	212	nscaf3031	2070132	2070776
BmMITE-5	1	212	nscaf2930	5584643	5584123
BmMITE-5	1	211	nscaf2888	9765710	9765500
BmMITE-5	1	211	nscaf1898	9006989	9007198
BmMITE-5	1	210	nscaf2767	4066867	4066666
BmMITE-5	1	210	nscaf44	6435	5785
BmMITE-5	1	210	nscaf3144	47437	47628
BmMITE-5	1	209	nscaf2943	1271084	1270879
BmMITE-5	1	209	nscaf1690	6787798	6787591

BmMITE-5	1	209	nscaf2053	97766	98407
BmMITE-5	1	209	nscaf2847	5099665	5099454
BmMITE-5	1	209	nscaf2529	4346404	4346204
BmMITE-5	1	209	nscaf2954	290068	289609
BmMITE-5	1	207	nscaf2511	2563204	2563402
BmMITE-5	1	207	nscaf3058	8809454	8807834
BmMITE-5	1	206	nscaf2575	4382272	4381631
BmMITE-5	1	204	nscaf2674	7526584	7526773
BmMITE-5	1	194	nscaf2883	1464233	1464426
BmMITE-5	1	187	scaffold547	106186	106371
BmMITE-5	1	182	nscaf2855	722475	722295
BmMITE-5	1	182	nscaf3058	7328543	7328724
BmMITE-5	1	173	nscaf2204	4402050	4401880
BmMITE-5	1	172	nscaf2983	943673	943502
BmMITE-6	3	221	nscaf2953	2207197	2206980
BmMITE-6	5	242	nscaf2880	77575	76040
BmMITE-6	5	267	nscaf2818	1017602	1017796
BmMITE-6	1	266	scaffold334	860	137
BmMITE-6	1	265	nscaf2865	1979853	1979539
BmMITE-6	1	242	nscaf2575	1701390	1701148
BmMITE-6	1	242	nscaf2833	93849	94186
BmMITE-6	1	236	nscaf3058	6814774	6814538
BmMITE-6	1	236	nscaf2882	241935	242171
BmMITE-6	1	236	nscaf2986	5627964	5627667
BmMITE-6	1	235	nscaf2902	6470694	6470923
BmMITE-7	5	548	nscaf2176	2056937	2059755
BmMITE-7	9	534	scaffold389	525	1
BmMITE-7	62	548	scaffold590	917	441
BmMITE-7	1	542	scaffold584	88331	87799
BmMITE-7	1	536	nscaf2529	1607433	1606877
BmMITE-7	1	474	scaffold385	474	1
BmMITE-7	1	464	nscaf2204	3885758	3886637
BmMITE-8	2	266	nscaf3026	796450	796714
BmMITE-8	2	266	nscaf3026	2066363	2066099
BmMITE-8	2	266	nscaf2890	1379220	1379485
BmMITE-8	2	266	nscaf3040	2326299	2326563
BmMITE-8	2	266	nscaf3040	2767273	2767010
BmMITE-8	2	266	nscaf3040	1661219	1660959
BmMITE-8	2	266	nscaf2903	1397782	1397523
BmMITE-8	2	266	nscaf2865	3281052	3281314

BmMITE-8	2	266	nscaf2865	4756386	4756650
BmMITE-8	2	266	nscaf2865	3959858	3959595
BmMITE-8	2	266	nscaf2829	121669	121928
BmMITE-8	2	266	nscaf2823	1601345	1601613
BmMITE-8	2	266	nscaf2823	4038081	4038345
BmMITE-8	2	266	nscaf1690	499944	500208
BmMITE-8	2	266	nscaf1690	3100539	3100261
BmMITE-8	2	266	nscaf1690	895066	894802
BmMITE-8	2	266	nscaf2889	2313398	2313662
BmMITE-8	2	266	nscaf2889	3242639	3242374
BmMITE-8	2	266	nscaf2795	487265	487529
BmMITE-8	2	266	nscaf2795	381029	380767
BmMITE-8	2	266	nscaf3066	823313	823577
BmMITE-8	2	266	nscaf2804	101405	101670
BmMITE-8	2	266	nscaf2136	2781095	2783471
BmMITE-8	2	266	nscaf2136	6449600	6449864
BmMITE-8	2	266	nscaf2136	7421182	7421446
BmMITE-8	2	266	nscaf1898	12362192	12362456
BmMITE-8	2	266	nscaf3093	714310	714046
BmMITE-8	2	266	nscaf3034	716709	716972
BmMITE-8	2	266	nscaf2953	2814880	2814610
BmMITE-8	2	266	nscaf2888	3381367	3383479
BmMITE-8	2	266	nscaf2888	7985910	7985646
BmMITE-8	2	266	nscaf2888	6888751	6888483
BmMITE-8	2	266	nscaf2818	2691098	2690834
BmMITE-8	2	266	nscaf2818	2416380	2416077
BmMITE-8	2	266	nscaf2210	3282379	3282642
BmMITE-8	2	266	nscaf2210	3547066	3547330
BmMITE-8	2	266	nscaf2210	4585652	4585915
BmMITE-8	2	266	nscaf2210	3318502	3318239
BmMITE-8	2	266	nscaf2176	2379249	2379506
BmMITE-8	2	266	nscaf3015	2228933	2228668
BmMITE-8	2	266	nscaf3015	484098	483834
BmMITE-8	2	266	nscaf2964	2144699	2144436
BmMITE-8	2	266	nscaf2964	1592288	1592024
BmMITE-8	2	266	nscaf2964	368456	368192
BmMITE-8	2	266	nscaf2827	24140	23876
BmMITE-8	2	266	nscaf2800	1828118	1828383
BmMITE-8	2	266	nscaf481	779529	779788
BmMITE-8	2	266	nscaf3089	438613	438347
BmMITE-8	2	266	nscaf3058	3018208	3018466
BmMITE-8	2	266	nscaf3058	6683568	6683305
BmMITE-8	2	266	nscaf2819	115690	115428

BmMITE-8	2	266	nscaf2279	539403	539138
BmMITE-8	2	266	nscaf3044	1310845	1310581
BmMITE-8	2	266	nscaf2951	1484318	1484054
BmMITE-8	2	266	nscaf2886	186894	187157
BmMITE-8	2	266	nscaf2886	980348	980612
BmMITE-8	2	266	nscaf2883	94417	94152
BmMITE-8	2	266	nscaf3063	1528415	1528150
BmMITE-8	2	266	nscaf2822	2143253	2142994
BmMITE-8	2	266	nscaf2589	5138987	5138723
BmMITE-8	2	266	nscaf2770	484434	484170
BmMITE-8	2	266	scaffold496	692	957
BmMITE-8	2	266	nscaf3106	164671	164936
BmMITE-8	2	266	nscaf98	1017040	1016777
BmMITE-8	2	266	nscaf3076	11239	11503
BmMITE-8	2	266	nscaf3031	1554854	1555118
BmMITE-8	2	266	nscaf3031	144965	144703
BmMITE-8	2	266	nscaf2828	3304135	3303878
BmMITE-8	2	266	nscaf2655	1602009	1602274
BmMITE-8	2	266	nscaf2655	3692077	3692341
BmMITE-8	2	266	nscaf2552	48839	48575
BmMITE-8	2	266	nscaf2962	286570	286300
BmMITE-8	2	266	nscaf2847	4824029	4823768
BmMITE-8	2	266	nscaf2887	2014648	2014910
BmMITE-8	2	266	scaffold139	5403	5132
BmMITE-8	2	266	nscaf1336	2083	1819
BmMITE-8	2	266	nscaf3053	197072	196808
BmMITE-8	2	266	nscaf3032	75104	74839
BmMITE-8	2	266	scaffold148	3554	3292
BmMITE-8	2	266	nscaf463	382031	382295
BmMITE-8	2	266	nscaf2825	445085	444817
BmMITE-8	2	266	nscaf2912	919859	919595
BmMITE-8	2	266	nscaf2855	3297334	3297598
BmMITE-8	2	266	nscaf2855	4738485	4738757
BmMITE-8	2	266	nscaf2408	2654	2913
BmMITE-8	2	266	nscaf2972	11190	10922
BmMITE-8	2	266	scaffold547	45945	45681
BmMITE-8	2	266	nscaf3078	23700	23436
BmMITE-8	2	266	nscaf3013	936861	936592
BmMITE-8	2	265	nscaf3026	3256551	3256806
BmMITE-8	2	265	nscaf3026	3748258	3748520
BmMITE-8	2	265	nscaf3026	4900755	4901019
BmMITE-8	2	265	nscaf3026	5265787	5265525
BmMITE-8	2	265	nscaf3026	3991465	3991202

BmMITE-8	2	265	nscaf3026	3843088	3842825
BmMITE-8	2	265	nscaf3040	4324755	4324500
BmMITE-8	2	265	nscaf3040	3575867	3575605
BmMITE-8	2	265	nscaf2865	3907891	3908687
BmMITE-8	2	265	nscaf2865	4550641	4550903
BmMITE-8	2	265	nscaf2865	1968164	1967901
BmMITE-8	2	265	nscaf2829	1663937	1664199
BmMITE-8	2	265	nscaf2823	2607186	2607457
BmMITE-8	2	265	scaffold377	501	238
BmMITE-8	2	265	nscaf2623	776912	777174
BmMITE-8	2	265	nscaf2623	764757	764494
BmMITE-8	2	265	nscaf1690	6037595	6037858
BmMITE-8	2	265	nscaf1690	7275513	7275778
BmMITE-8	2	265	nscaf1690	2256095	2255832
BmMITE-8	2	265	nscaf1108	451931	452194
BmMITE-8	2	265	nscaf1108	1769329	1769591
BmMITE-8	2	265	nscaf1108	2149592	2149330
BmMITE-8	2	265	nscaf2899	640587	640324
BmMITE-8	2	265	nscaf2795	2584593	2584856
BmMITE-8	2	265	nscaf2795	4082170	4082437
BmMITE-8	2	265	nscaf3079	1431640	1431902
BmMITE-8	2	265	nscaf3079	614798	614533
BmMITE-8	2	265	nscaf3066	117995	118255
BmMITE-8	2	265	nscaf2970	859863	860124
BmMITE-8	2	265	nscaf2970	1062473	1062204
BmMITE-8	2	265	nscaf2970	993349	993085
BmMITE-8	2	265	nscaf2136	1612718	1612981
BmMITE-8	2	265	nscaf3034	4007479	4007742
BmMITE-8	2	265	nscaf3003	1327737	1328019
BmMITE-8	2	265	nscaf2953	735370	735107
BmMITE-8	2	265	nscaf2888	3518031	3518294
BmMITE-8	2	265	nscaf2888	6777525	6777786
BmMITE-8	2	265	nscaf2888	6820330	6820593
BmMITE-8	2	265	nscaf2888	3624331	3624068
BmMITE-8	2	265	nscaf2818	1321224	1321487
BmMITE-8	2	265	scaffold915	148	411
BmMITE-8	2	265	scaffold896	14919	15181
BmMITE-8	2	265	nscaf3098	819538	819801
BmMITE-8	2	265	nscaf3098	271962	271697
BmMITE-8	2	265	nscaf3033	390770	391033
BmMITE-8	2	265	nscaf2210	4090558	4090821
BmMITE-8	2	265	nscaf2210	1169643	1169380
BmMITE-8	2	265	nscaf3055	884747	884484

BmMITE-8	2	265	nscaf2876	124891	124627
BmMITE-8	2	265	nscaf2986	2685425	2685688
BmMITE-8	2	265	nscaf2986	4698660	4698924
BmMITE-8	2	265	nscaf2986	4034889	4034632
BmMITE-8	2	265	nscaf2964	1750529	1750792
BmMITE-8	2	265	nscaf2964	3483459	3483721
BmMITE-8	2	265	nscaf2964	4678184	4677928
BmMITE-8	2	265	nscaf2964	1422992	1422618
BmMITE-8	2	265	nscaf2827	889043	889306
BmMITE-8	2	265	nscaf2827	1334544	1334811
BmMITE-8	2	265	nscaf1705	273714	273451
BmMITE-8	2	265	nscaf2674	3550673	3550410
BmMITE-8	2	265	nscaf2511	6435523	6435787
BmMITE-8	2	265	nscaf2511	5692121	5691858
BmMITE-8	2	265	nscaf1681	2471595	2471333
BmMITE-8	2	265	nscaf2983	825088	825347
BmMITE-8	2	265	nscaf2983	1421171	1421437
BmMITE-8	2	265	nscaf2983	3331639	3331378
BmMITE-8	2	265	nscaf2983	3264254	3263991
BmMITE-8	2	265	nscaf2983	3208799	3208534
BmMITE-8	2	265	nscaf2983	605332	605070
BmMITE-8	2	265	nscaf2930	5437944	5437681
BmMITE-8	2	265	nscaf2930	1820752	1820490
BmMITE-8	2	265	nscaf2916	526893	526630
BmMITE-8	2	265	nscaf2901	1073384	1073121
BmMITE-8	2	265	nscaf2901	276574	276310
BmMITE-8	2	265	nscaf2901	88190	87896
BmMITE-8	2	265	nscaf2801	213512	213773
BmMITE-8	2	265	nscaf2398	47640	47377
BmMITE-8	2	265	nscaf3058	7660729	7660991
BmMITE-8	2	265	nscaf3058	5103511	5103248
BmMITE-8	2	265	nscaf3058	4775514	4775257
BmMITE-8	2	265	nscaf3058	897677	897415
BmMITE-8	2	265	nscaf2330	351425	351688
BmMITE-8	2	265	nscaf2330	3620667	3620403
BmMITE-8	2	265	nscaf2279	7198	7462
BmMITE-8	2	265	nscaf3072	2307955	2307692
BmMITE-8	2	265	nscaf2886	837227	837488
BmMITE-8	2	265	nscaf2789	217086	217349
BmMITE-8	2	265	nscaf2789	575068	575335
BmMITE-8	2	265	nscaf2789	656940	657204
BmMITE-8	2	265	nscaf2789	944580	944844
BmMITE-8	2	265	nscaf2789	1285433	1285698

BmMITE-8	2	265	nscaf3099	203354	203619
BmMITE-8	2	265	nscaf3099	1318245	1318507
BmMITE-8	2	265	nscaf3099	4044600	4044862
BmMITE-8	2	265	nscaf3099	4533115	4532852
BmMITE-8	2	265	nscaf3099	2086759	2086494
BmMITE-8	2	265	nscaf3097	425366	425630
BmMITE-8	2	265	nscaf3063	3172845	3172582
BmMITE-8	2	265	nscaf3063	2225048	2224786
BmMITE-8	2	265	nscaf3063	237850	237587
BmMITE-8	2	265	nscaf3054	32070	32338
BmMITE-8	2	265	nscaf3054	42990	42727
BmMITE-8	2	265	nscaf2993	1779332	1779591
BmMITE-8	2	265	nscaf2993	7404913	7404650
BmMITE-8	2	265	nscaf2948	348338	348602
BmMITE-8	2	265	nscaf2948	1002882	1002619
BmMITE-8	2	265	nscaf2822	1573574	1573837
BmMITE-8	2	265	nscaf2589	5255880	5256141
BmMITE-8	2	265	nscaf2589	915852	915589
BmMITE-8	2	265	nscaf2204	3530035	3530297
BmMITE-8	2	265	nscaf2204	4276521	4276784
BmMITE-8	2	265	nscaf2860	1017921	1018182
BmMITE-8	2	265	nscaf3031	5249148	5249407
BmMITE-8	2	265	nscaf3031	5830571	5827541
BmMITE-8	2	265	nscaf3031	5020602	5020340
BmMITE-8	2	265	nscaf3031	2476238	2475981
BmMITE-8	2	265	nscaf2828	1831688	1831951
BmMITE-8	2	265	nscaf2655	453986	454249
BmMITE-8	2	265	scaffold818	4729	4992
BmMITE-8	2	265	scaffold352	304	565
BmMITE-8	2	265	nscaf3104	199976	200239
BmMITE-8	2	265	nscaf2847	3287196	3286932
BmMITE-8	2	265	nscaf2847	1205040	1204778
BmMITE-8	2	265	nscaf2943	2949862	2949602
BmMITE-8	2	265	scaffold693	47353	47616
BmMITE-8	2	265	nscaf3053	364890	365153
BmMITE-8	2	265	nscaf2987	443772	443509
BmMITE-8	2	265	nscaf2813	905527	905787
BmMITE-8	2	265	nscaf2457	302	39
BmMITE-8	2	265	nscaf916	8521	8785
BmMITE-8	2	265	nscaf2959	230504	230243
BmMITE-8	2	265	nscaf3074	169496	169759
BmMITE-8	2	265	nscaf3045	2211339	2205460
BmMITE-8	2	265	scaffold632	90820	91081

BmMITE-8	2	265	nscaf3041	421647	421911
BmMITE-8	2	265	nscaf2912	894792	894530
BmMITE-8	2	265	nscaf2855	2638703	2638964
BmMITE-8	2	265	nscaf2767	3553569	3553307
BmMITE-8	2	265	nscaf2564	70475	70212
BmMITE-8	2	265	nscaf2053	183366	183106
BmMITE-8	2	265	nscaf2797	516055	515800
BmMITE-8	2	265	nscaf3090	48998	48735
BmMITE-8	2	265	nscaf3037	61872	62143
BmMITE-8	2	265	nscaf3071	585262	584997
BmMITE-8	2	265	nscaf2852	796937	797208
BmMITE-8	2	265	nscaf2529	824042	823664
BmMITE-8	2	265	scaffold166	1974	2239
BmMITE-8	2	265	nscaf2954	31811	32366
BmMITE-8	2	265	nscaf2894	3606	3957
BmMITE-8	2	265	nscaf3003	296330	296039
BmMITE-8	2	265	nscaf2575	3492072	3492551
BmMITE-8	2	265	nscaf2965	233265	234078
BmMITE-8	2	263	nscaf2623	450805	450304
BmMITE-8	2	265	nscaf2511	1994528	1995087
BmMITE-8	2	263	nscaf2836	646493	638537
BmMITE-8	2	262	nscaf3026	4723313	4723054
BmMITE-8	2	262	nscaf2865	3445726	3445466
BmMITE-8	2	262	nscaf2853	5286185	5285925
BmMITE-8	2	262	nscaf2931	817312	817052
BmMITE-8	2	265	nscaf2888	4598231	4598513
BmMITE-8	2	262	nscaf2888	1983995	1983741
BmMITE-8	2	262	scaffold385	401	141
BmMITE-8	2	266	nscaf2948	703477	704114
BmMITE-8	2	262	nscaf2822	2078396	2078129
BmMITE-8	2	262	nscaf2851	1379188	1379448
BmMITE-8	2	262	nscaf2943	244436	244175
BmMITE-8	2	262	nscaf2855	5907024	5907284
BmMITE-8	2	263	nscaf2865	1030558	1030289
BmMITE-8	2	261	nscaf3003	3732363	3732104
BmMITE-8	2	261	nscaf2868	63313	63571
BmMITE-8	2	260	nscaf2795	2827536	2827794
BmMITE-8	2	266	nscaf2801	148483	148236
BmMITE-8	2	260	nscaf2859	193642	193900
BmMITE-8	2	266	nscaf2855	2945330	2945578
BmMITE-8	2	265	nscaf2865	2201630	2201383
BmMITE-8	2	259	nscaf2829	1698690	1698433
BmMITE-8	2	265	nscaf2795	3740702	3740458

BmMITE-8	2	265	nscaf2136	2704713	2704467
BmMITE-8	2	265	nscaf2964	2278809	2279056
BmMITE-8	2	265	nscaf2964	1497052	1496806
BmMITE-8	2	265	nscaf2993	379845	379597
BmMITE-8	2	259	nscaf2980	223599	223342
BmMITE-8	2	265	nscaf2828	1704756	1705002
BmMITE-8	2	265	nscaf736	22554	22801
BmMITE-8	2	257	nscaf2330	3490335	3490079
BmMITE-8	2	262	nscaf3058	1468993	1468749
BmMITE-8	2	265	nscaf3026	3748520	3747996
BmMITE-8	2	262	nscaf2623	506181	506829
BmMITE-8	2	255	nscaf2853	594413	594671
BmMITE-8	2	265	nscaf3003	362886	362635
BmMITE-8	2	261	nscaf2888	6229405	6229163
BmMITE-8	2	265	nscaf2827	945137	944883
BmMITE-8	2	265	nscaf2176	939980	940231
BmMITE-8	2	266	nscaf2674	909188	909425
BmMITE-8	2	266	nscaf3072	1570344	1569972
BmMITE-8	2	265	nscaf1690	6615124	6615363
BmMITE-8	2	265	nscaf1690	2078780	2078540
BmMITE-8	2	266	nscaf2970	24590	24350
BmMITE-8	2	253	nscaf3038	131226	131477
BmMITE-8	2	265	nscaf3027	1464023	1463785
BmMITE-8	2	266	nscaf3026	168207	168446
BmMITE-8	2	266	nscaf3026	5738907	5738666
BmMITE-8	2	266	nscaf2829	4200894	4200653
BmMITE-8	2	266	nscaf2829	1974249	1974014
BmMITE-8	2	266	nscaf2829	1759219	1758980
BmMITE-8	2	266	nscaf2829	1507872	1507631
BmMITE-8	2	266	nscaf2623	624282	624522
BmMITE-8	2	266	nscaf1690	623421	623662
BmMITE-8	2	266	nscaf1690	1583168	1583458
BmMITE-8	2	266	nscaf1108	1220819	1220580
BmMITE-8	2	266	nscaf3066	130609	130849
BmMITE-8	2	266	nscaf2970	722841	723081
BmMITE-8	2	266	nscaf2136	5221267	5221505
BmMITE-8	2	266	nscaf2136	3448763	3448523
BmMITE-8	2	266	nscaf2136	1480218	1479978
BmMITE-8	2	266	nscaf1898	7171348	7171588
BmMITE-8	2	266	nscaf1898	13311540	13311780
BmMITE-8	2	266	nscaf1898	15218150	15218389
BmMITE-8	2	266	nscaf1898	9778488	9778247
BmMITE-8	2	266	nscaf3003	4443572	4444253

BmMITE-8	2	266	nscaf3003	4493235	4493962
BmMITE-8	2	266	nscaf2937	341446	341206
BmMITE-8	2	266	nscaf2902	3800204	3800599
BmMITE-8	2	266	nscaf2818	2345293	2345533
BmMITE-8	2	266	nscaf2818	2653164	2653402
BmMITE-8	2	266	nscaf2818	2834134	2834370
BmMITE-8	2	266	nscaf3038	45818	46059
BmMITE-8	2	266	nscaf3033	218118	217880
BmMITE-8	2	266	nscaf2210	2563855	2563615
BmMITE-8	2	266	nscaf3055	1652130	1652371
BmMITE-8	2	266	nscaf3055	1931418	1931659
BmMITE-8	2	266	nscaf2986	369405	369645
BmMITE-8	2	266	nscaf2986	2865793	2865554
BmMITE-8	2	266	nscaf2964	2155105	2155344
BmMITE-8	2	266	nscaf2964	3798157	3793681
BmMITE-8	2	266	nscaf2827	2268433	2268189
BmMITE-8	2	252	nscaf2800	2773709	2773458
BmMITE-8	2	266	nscaf2800	412169	411928
BmMITE-8	2	266	nscaf2674	3591846	3591607
BmMITE-8	2	266	nscaf2983	859948	860190
BmMITE-8	2	266	nscaf2398	226259	226874
BmMITE-8	2	266	nscaf3058	1035045	1035291
BmMITE-8	2	266	nscaf3072	2551691	2551928
BmMITE-8	2	266	nscaf3099	3754228	3754468
BmMITE-8	2	266	nscaf3099	2303767	2303535
BmMITE-8	2	266	nscaf3063	2261834	2261595
BmMITE-8	2	266	nscaf2993	4188131	4188371
BmMITE-8	2	266	nscaf2589	233579	233341
BmMITE-8	2	266	nscaf2204	50686	50443
BmMITE-8	2	266	nscaf2860	1639098	1638859
BmMITE-8	2	266	nscaf3076	38137	37898
BmMITE-8	2	266	nscaf2828	4165349	4165106
BmMITE-8	2	266	nscaf2828	4061101	4060862
BmMITE-8	2	266	nscaf2828	1305905	1305673
BmMITE-8	2	266	nscaf2655	3455109	3454869
BmMITE-8	2	266	nscaf3104	267938	268177
BmMITE-8	2	266	nscaf2847	1701751	1701991
BmMITE-8	2	266	nscaf2847	2069631	2069870
BmMITE-8	2	266	nscaf2943	2624615	2624857
BmMITE-8	2	266	nscaf2943	3680481	3680241
BmMITE-8	2	266	nscaf2943	367784	367540
BmMITE-8	2	266	nscaf3074	688901	689135
BmMITE-8	2	266	nscaf2855	3016292	3016052

BmMITE-8	2	266	nscaf2767	2801405	2801645
BmMITE-8	2	266	nscaf2767	2531694	2531452
BmMITE-8	2	266	nscaf3062	164265	164499
BmMITE-8	2	266	nscaf2529	2450817	2451056
BmMITE-8	2	266	nscaf2836	462448	462688
BmMITE-8	2	265	nscaf3005	2041583	2041822
BmMITE-8	2	266	nscaf125	232451	232213
BmMITE-8	2	266	scaffold114	7898	7659
BmMITE-8	2	266	nscaf2998	1599943	1600194
BmMITE-8	2	266	nscaf2995	222202	221961
BmMITE-8	2	265	nscaf3026	1204986	1205224
BmMITE-8	2	265	nscaf3026	6351247	6351007
BmMITE-8	2	265	nscaf2890	1461645	1461885
BmMITE-8	2	265	nscaf3040	2162928	2163168
BmMITE-8	2	265	nscaf3040	4645156	4644917
BmMITE-8	2	265	nscaf2903	881154	880916
BmMITE-8	2	265	nscaf2865	3691818	3692057
BmMITE-8	2	265	nscaf2829	3782074	3782313
BmMITE-8	2	265	nscaf2829	4074621	4074383
BmMITE-8	2	265	nscaf2829	639675	639442
BmMITE-8	2	265	nscaf1690	166744	166982
BmMITE-8	2	265	nscaf1690	3521830	3521591
BmMITE-8	2	265	nscaf1108	2174758	2174997
BmMITE-8	2	265	nscaf2889	2872587	2872349
BmMITE-8	2	265	nscaf2889	2582718	2582355
BmMITE-8	2	265	nscaf2795	22122	22361
BmMITE-8	2	265	nscaf3066	594476	594716
BmMITE-8	2	265	nscaf2853	3176100	3176341
BmMITE-8	2	265	nscaf2853	6307427	6307667
BmMITE-8	2	265	nscaf2136	440754	440993
BmMITE-8	2	265	nscaf2136	5904886	5905125
BmMITE-8	2	265	nscaf2136	1981622	1981383
BmMITE-8	2	265	nscaf1898	1992928	1993167
BmMITE-8	2	265	nscaf1898	3365376	3365617
BmMITE-8	2	265	nscaf1898	7010541	7010781
BmMITE-8	2	265	nscaf1898	7379232	7379469
BmMITE-8	2	265	nscaf1898	8723931	8724171
BmMITE-8	2	265	nscaf1898	11503619	11503380
BmMITE-8	2	265	nscaf3034	1372876	1373112
BmMITE-8	2	265	nscaf3034	2445992	2446234
BmMITE-8	2	265	nscaf3034	2497495	2497256
BmMITE-8	2	265	nscaf3003	1758336	1758579
BmMITE-8	2	265	nscaf3003	2327263	2327032

BmMITE-8	2	265	nscaf2953	2849958	2847000
BmMITE-8	2	265	nscaf2902	7017593	7017833
BmMITE-8	2	265	nscaf2902	10023167	10023406
BmMITE-8	2	265	nscaf2902	968343	968106
BmMITE-8	2	265	nscaf2888	2376077	2376316
BmMITE-8	2	265	nscaf2888	6273470	6273227
BmMITE-8	2	265	nscaf2818	1789874	1790114
BmMITE-8	2	265	nscaf2818	1368528	1368289
BmMITE-8	2	265	nscaf3098	2048142	2048390
BmMITE-8	2	265	nscaf2210	3935411	3935171
BmMITE-8	2	265	nscaf2176	4045348	4045587
BmMITE-8	2	265	nscaf2176	4114810	4114573
BmMITE-8	2	266	nscaf2176	1988631	1988392
BmMITE-8	2	265	nscaf3027	3754548	3754786
BmMITE-8	2	265	nscaf3015	4396076	4396313
BmMITE-8	2	265	nscaf2800	1029610	1029848
BmMITE-8	2	265	nscaf2800	1736829	1737073
BmMITE-8	2	265	nscaf2674	5692088	5691850
BmMITE-8	2	265	nscaf2674	5088013	5087773
BmMITE-8	2	265	nscaf2674	1543632	1543392
BmMITE-8	2	265	nscaf2511	5512006	5512245
BmMITE-8	2	265	nscaf2511	1463349	1463104
BmMITE-8	2	265	nscaf1681	5573679	5573440
BmMITE-8	2	265	nscaf2930	3620293	3620531
BmMITE-8	2	265	nscaf2901	1406403	1406641
BmMITE-8	2	265	nscaf3058	6229870	6230115
BmMITE-8	2	265	nscaf3058	8330701	8330940
BmMITE-8	2	265	nscaf3058	3459077	3458720
BmMITE-8	2	265	nscaf2819	5726	5487
BmMITE-8	2	265	nscaf2279	159232	159468
BmMITE-8	2	265	nscaf3072	1162919	1163153
BmMITE-8	2	265	nscaf3044	112043	111802
BmMITE-8	2	265	nscaf3099	625223	625462
BmMITE-8	2	265	nscaf3099	4337786	4337543
BmMITE-8	2	265	nscaf3097	810067	810305
BmMITE-8	2	265	nscaf3097	58232	57778
BmMITE-8	2	265	nscaf3063	416187	416427
BmMITE-8	2	265	nscaf3063	2914764	2915005
BmMITE-8	2	265	nscaf3063	3123132	3123373
BmMITE-8	2	265	nscaf3063	2791600	2791367
BmMITE-8	2	265	nscaf3063	573429	573189
BmMITE-8	2	265	nscaf2993	5826049	5825807
BmMITE-8	2	265	nscaf2993	309230	308992

BmMITE-8	2	251	nscaf2589	1453638	1453890
BmMITE-8	2	265	nscaf2589	4732654	4732894
BmMITE-8	2	265	nscaf2204	4094892	4094652
BmMITE-8	2	265	nscaf2204	3617044	3616805
BmMITE-8	2	265	nscaf2204	2947121	2946878
BmMITE-8	2	265	nscaf2860	924492	924739
BmMITE-8	2	265	nscaf2770	1268835	1269074
BmMITE-8	2	265	nscaf2891	2523509	2523272
BmMITE-8	2	266	nscaf98	982437	982197
BmMITE-8	2	265	nscaf3031	6227372	6227612
BmMITE-8	2	265	nscaf3031	2509566	2509326
BmMITE-8	2	265	nscaf2828	871658	871897
BmMITE-8	2	265	nscaf2828	1190990	1191228
BmMITE-8	2	265	nscaf2655	1627691	1627932
BmMITE-8	2	265	nscaf2655	3403175	3403414
BmMITE-8	2	265	nscaf2655	3168321	3168083
BmMITE-8	2	265	nscaf2655	2786618	2786044
BmMITE-8	2	265	nscaf2887	1659400	1659640
BmMITE-8	2	265	nscaf2887	1319310	1319068
BmMITE-8	2	265	nscaf2943	1710837	1711077
BmMITE-8	2	265	nscaf2943	1800380	1800623
BmMITE-8	2	265	nscaf2943	3473730	3473492
BmMITE-8	2	265	nscaf3053	414591	414828
BmMITE-8	2	265	nscaf2813	355998	356237
BmMITE-8	2	265	nscaf3032	486155	485917
BmMITE-8	2	265	nscaf3074	334811	335049
BmMITE-8	2	265	nscaf3074	3455	3216
BmMITE-8	2	265	nscaf2855	839088	839329
BmMITE-8	2	265	nscaf2855	1953648	1953891
BmMITE-8	2	265	nscaf2767	1661802	1662043
BmMITE-8	2	265	nscaf2767	3633705	3633943
BmMITE-8	2	265	nscaf2575	1840662	1840901
BmMITE-8	2	265	nscaf2575	2884678	2884441
BmMITE-8	2	265	nscaf2833	526398	526159
BmMITE-8	2	265	nscaf2852	2511567	2511328
BmMITE-8	2	266	nscaf3013	764135	763604
BmMITE-8	2	265	nscaf2529	1861940	1862180
BmMITE-8	2	265	nscaf2836	60326	60087
BmMITE-8	2	266	nscaf3005	1231970	1231730
BmMITE-8	2	265	nscaf3131	33949	33712
BmMITE-8	2	266	nscaf125	230662	230972
BmMITE-8	2	265	nscaf3008	189195	189434
BmMITE-8	2	265	scaffold319	42	281

BmMITE-8	2	265	nscaf2838	677500	677262
BmMITE-8	2	265	scaffold179	348	109
BmMITE-8	2	265	nscaf2998	955364	955125
BmMITE-8	2	265	nscaf2998	742493	742256
BmMITE-8	2	264	nscaf3040	4304445	4304683
BmMITE-8	2	265	nscaf1690	1864938	1864694
BmMITE-8	2	266	nscaf1898	15374987	15375258
BmMITE-8	2	265	nscaf1898	13372910	13372668
BmMITE-8	2	264	nscaf1898	11882051	11881812
BmMITE-8	2	266	nscaf3098	239854	239408
BmMITE-8	2	265	nscaf2827	1389718	1389956
BmMITE-8	2	265	nscaf1705	26895	26656
BmMITE-8	2	265	nscaf2674	6332037	6332274
BmMITE-8	2	265	nscaf2330	2746897	2747492
BmMITE-8	2	264	nscaf2948	1027802	1027563
BmMITE-8	2	265	nscaf2847	2300325	2300561
BmMITE-8	2	265	nscaf463	1066667	1066213
BmMITE-8	2	265	nscaf2825	631857	631614
BmMITE-8	2	264	nscaf2855	2201059	2201298
BmMITE-8	2	265	nscaf2855	4004710	4004950
BmMITE-8	2	264	nscaf2529	5521518	5521748
BmMITE-8	2	264	nscaf2838	1197658	1197421
BmMITE-8	2	264	scaffold280	240	1
BmMITE-8	2	263	nscaf2136	3115354	3115591
BmMITE-8	2	265	nscaf1898	5583941	5584337
BmMITE-8	2	265	nscaf3034	4470368	4470128
BmMITE-8	2	266	nscaf2902	10794405	10794647
BmMITE-8	2	265	nscaf2902	591065	590826
BmMITE-8	2	263	nscaf2818	441526	441767
BmMITE-8	2	265	nscaf2674	5224834	5224596
BmMITE-8	2	263	nscaf2330	2251364	2251603
BmMITE-8	2	264	nscaf3099	2799182	2798944
BmMITE-8	2	263	nscaf3054	45966	46201
BmMITE-8	2	263	nscaf2993	1903429	1903190
BmMITE-8	2	263	nscaf2891	213367	213603
BmMITE-8	2	265	nscaf2962	679057	678815
BmMITE-8	2	265	nscaf2943	2696359	2695849
BmMITE-8	2	266	nscaf2868	1420006	1420243
BmMITE-8	2	265	nscaf3026	3244234	3243994
BmMITE-8	2	262	nscaf3040	2051018	2051255
BmMITE-8	2	262	nscaf2823	122187	122424
BmMITE-8	2	265	nscaf3066	1207059	1207304
BmMITE-8	2	265	nscaf1898	8048854	8049097

BmMITE-8	2	262	nscaf3034	995261	995025
BmMITE-8	2	266	nscaf2931	1978049	1977805
BmMITE-8	2	262	nscaf2888	1281311	1281075
BmMITE-8	2	262	nscaf3033	55108	54872
BmMITE-8	2	266	nscaf2210	3985309	3985827
BmMITE-8	2	266	nscaf2674	2727023	2727262
BmMITE-8	2	262	nscaf2674	2215952	2215717
BmMITE-8	2	265	nscaf2330	1958173	1958412
BmMITE-8	2	262	nscaf2951	482971	482733
BmMITE-8	2	265	nscaf2589	3200396	3200157
BmMITE-8	2	265	nscaf3031	6129541	6129821
BmMITE-8	2	266	nscaf2847	5742785	5742545
BmMITE-8	2	262	nscaf2943	412471	412235
BmMITE-8	2	262	nscaf2987	334701	334937
BmMITE-8	2	262	nscaf3032	1640281	1640046
BmMITE-8	2	262	nscaf2216	1219328	1219564
BmMITE-8	2	266	scaffold349	587	69
BmMITE-8	2	265	nscaf3003	2339853	2340104
BmMITE-8	2	265	nscaf2902	10658786	10659026
BmMITE-8	2	265	nscaf2986	1703627	1703386
BmMITE-8	2	262	nscaf3099	4486882	4486645
BmMITE-8	2	265	nscaf2993	2195358	2195595
BmMITE-8	2	261	nscaf2993	7685847	7686084
BmMITE-8	2	266	nscaf3031	667190	666951
BmMITE-8	2	261	nscaf2962	391304	391538
BmMITE-8	2	261	nscaf3008	458660	458894
BmMITE-8	2	261	scaffold426	134	370
BmMITE-8	2	260	nscaf2889	1570358	1570841
BmMITE-8	2	246	nscaf3089	255968	256212
BmMITE-8	2	266	nscaf2623	483001	482633
BmMITE-8	2	262	nscaf3003	3247114	3246877
BmMITE-8	2	265	nscaf2800	2569424	2569949
BmMITE-8	2	259	nscaf2851	746591	746356
BmMITE-8	2	262	nscaf2905	312784	312540
BmMITE-8	2	265	nscaf2686	481624	481384
BmMITE-8	2	266	nscaf2889	3179512	3179775
BmMITE-8	2	266	nscaf2853	4610890	4611128
BmMITE-8	2	262	nscaf1898	15550563	15550802
BmMITE-8	2	260	nscaf3003	3476703	3477090
BmMITE-8	2	265	nscaf2398	71268	71528
BmMITE-8	2	263	nscaf3031	77279	77039
BmMITE-8	2	263	nscaf2813	522849	523239
BmMITE-8	2	244	nscaf3068	214989	215232

BmMITE-8	2	265	nscaf2953	2898225	2897511
BmMITE-8	2	265	nscaf2964	3312892	3312652
BmMITE-8	2	265	nscaf2330	397553	397312
BmMITE-8	2	256	nscaf2890	381349	381580
BmMITE-8	2	266	nscaf1898	12987014	12986636
BmMITE-8	2	265	nscaf3035	1087674	1087916
BmMITE-8	2	266	nscaf3079	914875	915130
BmMITE-8	2	265	nscaf2888	7078113	7078370
BmMITE-8	2	265	nscaf2888	7871493	7871255
BmMITE-8	2	265	nscaf2847	7657915	7658191
BmMITE-8	2	265	nscaf2797	189402	188736
BmMITE-8	2	265	scaffold657	2668	2903
BmMITE-8	2	239	nscaf3099	2161881	2162118
BmMITE-8	2	266	nscaf2889	1708177	1708424
BmMITE-8	2	265	nscaf2847	2441396	2441141
BmMITE-8	2	265	nscaf2053	435637	435886
BmMITE-8	2	237	nscaf2964	2057980	2058215
BmMITE-8	2	266	nscaf2943	1655192	1655417
BmMITE-8	2	265	nscaf1898	14415502	14415735
BmMITE-8	2	265	nscaf2888	8060160	8059918
BmMITE-8	2	250	nscaf2943	3367536	3367314
BmMITE-8	2	265	nscaf3031	997408	997165
BmMITE-8	2	234	nscaf2855	4415365	4415600
BmMITE-8	2	234	nscaf684	13614	13845
BmMITE-8	2	234	nscaf3090	257896	258128
BmMITE-8	2	266	nscaf2136	5105983	5105750
BmMITE-8	2	247	nscaf3031	5666767	5666989
BmMITE-8	2	266	nscaf2983	1282099	1282319
BmMITE-8	2	232	nscaf3058	2906692	2906923
BmMITE-8	2	246	nscaf2828	5286354	5286135
BmMITE-8	2	266	nscaf2575	1497413	1497157
BmMITE-8	2	231	nscaf3048	1382454	1382683
BmMITE-8	2	259	nscaf1898	4101723	4101966
BmMITE-8	2	245	nscaf3035	647355	647137
BmMITE-8	2	249	nscaf3071	944793	944549
BmMITE-8	2	266	nscaf2801	493638	493407
BmMITE-8	2	266	nscaf3099	3098268	3098485
BmMITE-8	2	265	nscaf2953	1135284	1135058
BmMITE-8	2	229	nscaf2964	1925111	1925338
BmMITE-8	2	265	nscaf3097	432390	432156
BmMITE-8	2	265	nscaf2993	6245264	6245051
BmMITE-8	2	265	nscaf2964	297377	297125
BmMITE-8	2	265	nscaf2883	1834419	1834725

BmMITE-8	2	241	nscaf2828	3185447	3185662
BmMITE-8	2	266	nscaf3003	490714	490187
BmMITE-8	2	265	nscaf2943	3190513	3190900
BmMITE-8	2	266	nscaf3027	180384	180624
BmMITE-8	2	265	nscaf3015	4080736	4080971
BmMITE-8	2	266	nscaf2964	474510	474251
BmMITE-8	2	266	nscaf3040	4072052	4072283
BmMITE-8	2	265	nscaf3026	4254544	4254311
BmMITE-8	2	266	nscaf2829	4188195	4187958
BmMITE-8	2	266	nscaf2853	2761980	2761740
BmMITE-8	2	266	nscaf1898	8130377	8130140
BmMITE-8	2	266	nscaf2888	9505511	9505273
BmMITE-8	2	266	nscaf3055	1351451	1351694
BmMITE-8	2	236	nscaf2789	852790	852135
BmMITE-8	2	265	nscaf2589	4444666	4444898
BmMITE-8	2	266	nscaf98	461652	461892
BmMITE-8	2	266	nscaf2529	2454861	2454622
BmMITE-8	2	265	nscaf3026	978396	978639
BmMITE-8	2	265	nscaf3040	4603171	4602932
BmMITE-8	2	265	nscaf2970	490885	491125
BmMITE-8	2	265	nscaf2853	4900724	4900488
BmMITE-8	2	265	nscaf2136	2878058	2877826
BmMITE-8	2	237	nscaf2136	2199216	2199005
BmMITE-8	2	266	nscaf2902	4584887	4584665
BmMITE-8	2	266	nscaf2986	3993634	3993093
BmMITE-8	2	265	nscaf2964	234440	234678
BmMITE-8	2	264	nscaf2589	5036138	5036379
BmMITE-8	2	265	nscaf2770	1255631	1255870
BmMITE-8	2	265	nscaf2655	1925698	1925003
BmMITE-8	2	265	nscaf1681	4323766	4323527
BmMITE-8	2	264	nscaf3099	34829	34592
BmMITE-8	2	266	nscaf2891	1096716	1096483
BmMITE-8	2	266	nscaf2902	10922640	10922403
BmMITE-8	2	265	nscaf2902	6907448	6907207
BmMITE-8	2	266	nscaf2210	3220355	3220117
BmMITE-8	2	233	nscaf3099	2681660	2681457
BmMITE-8	2	263	nscaf2943	354988	354754
BmMITE-8	2	265	nscaf1108	1014483	1014246
BmMITE-8	2	265	nscaf2655	366870	366632
BmMITE-8	2	265	nscaf2829	2399537	2399296
BmMITE-8	2	231	nscaf2823	4316721	4316930
BmMITE-8	2	261	nscaf2795	2248372	2248140
BmMITE-8	2	260	nscaf3048	1251114	1250858

BmMITE-8	2	231	nscaf2818	346536	346333
BmMITE-8	2	265	nscaf2589	6048418	6048784
BmMITE-8	2	265	nscaf2780	219419	219180
BmMITE-8	2	265	scaffold861	5392	5153
BmMITE-8	2	265	scaffold690	19750	19511
BmMITE-8	2	266	nscaf2903	912011	912250
BmMITE-8	2	265	nscaf2865	440596	440825
BmMITE-8	2	261	nscaf1705	1120115	1120351
BmMITE-8	2	265	nscaf2943	1072591	1072827
BmMITE-8	2	266	nscaf2943	2330259	2330496
BmMITE-8	2	265	nscaf3005	1006161	1005924
BmMITE-8	2	265	nscaf1690	794347	791124
BmMITE-8	2	266	nscaf3034	255222	255450
BmMITE-8	2	266	nscaf2883	2494240	2493977
BmMITE-8	2	266	nscaf2575	2571635	2571394
BmMITE-8	3	266	nscaf2902	10978062	10977797
BmMITE-8	3	266	nscaf2964	4256545	4256282
BmMITE-8	3	266	nscaf2828	5565546	5565809
BmMITE-8	3	265	nscaf2931	2121655	2121916
BmMITE-8	3	265	nscaf2876	299757	300019
BmMITE-8	3	265	nscaf2986	2705267	2705528
BmMITE-8	3	265	nscaf3099	3951635	3951899
BmMITE-8	3	261	nscaf3040	2830080	2830664
BmMITE-8	3	261	nscaf3040	4265566	4265828
BmMITE-8	3	261	nscaf2889	1115624	1115366
BmMITE-8	3	261	nscaf2210	4355897	4356154
BmMITE-8	3	261	nscaf3097	569978	570238
BmMITE-8	3	261	nscaf2993	3524831	3525089
BmMITE-8	3	261	nscaf2810	37666	40098
BmMITE-8	3	261	nscaf3090	154280	154022
BmMITE-8	3	266	nscaf2829	3316479	3316240
BmMITE-8	3	266	nscaf2828	3857159	3857398
BmMITE-8	3	265	nscaf2899	54732	54465
BmMITE-8	3	265	nscaf2853	5591797	5592031
BmMITE-8	3	265	nscaf2800	1747723	1747489
BmMITE-8	3	266	nscaf3031	3911852	3911203
BmMITE-8	3	265	nscaf2847	7931712	7931475
BmMITE-8	3	265	nscaf3005	1749273	1749043
BmMITE-8	3	266	nscaf2902	9593544	9593929
BmMITE-8	3	263	nscaf3072	971251	971487
BmMITE-8	3	266	nscaf2970	688752	689008
BmMITE-8	3	265	nscaf2912	765382	765621
BmMITE-8	3	261	nscaf2759	948233	948467

BmMITE-8	3	261	nscaf2902	7237832	7237605
BmMITE-8	3	261	nscaf3055	54572	54339
BmMITE-8	3	261	nscaf2964	4819460	4819224
BmMITE-8	3	261	nscaf2828	1022266	1022034
BmMITE-8	3	265	nscaf3040	371200	371552
BmMITE-8	3	266	nscaf2827	319817	319553
BmMITE-8	3	260	nscaf3058	5417619	5417852
BmMITE-8	3	260	nscaf3045	1451762	1451534
BmMITE-8	3	261	nscaf2889	3820595	3820361
BmMITE-8	3	259	nscaf2888	8984994	8984764
BmMITE-8	3	261	nscaf2912	873106	872874
BmMITE-8	3	260	nscaf2767	1450914	1451147
BmMITE-8	3	265	nscaf2912	1396276	1396522
BmMITE-8	3	221	nscaf3071	96755	96972
BmMITE-8	3	261	nscaf2589	1758667	1758431
BmMITE-8	4	266	nscaf2883	1870759	1870504
BmMITE-8	4	266	nscaf479	5942	6203
BmMITE-8	4	265	nscaf2865	3852902	3852641
BmMITE-8	4	265	nscaf2829	4241090	4240830
BmMITE-8	4	265	nscaf2829	4155348	4155087
BmMITE-8	4	265	nscaf2953	2017058	2016797
BmMITE-8	4	265	nscaf3097	1898392	1898131
BmMITE-8	4	264	nscaf2800	199844	199585
BmMITE-8	4	265	nscaf3003	1897000	1897925
BmMITE-8	4	263	nscaf2953	3289919	3289660
BmMITE-8	4	262	nscaf1690	1269757	1270013
BmMITE-8	4	262	nscaf1690	516704	516446
BmMITE-8	4	262	nscaf3098	1612084	1611826
BmMITE-8	4	262	nscaf2176	3034685	3034429
BmMITE-8	4	262	nscaf3015	187867	188119
BmMITE-8	4	262	nscaf2901	910906	911165
BmMITE-8	4	262	nscaf2993	2104479	2104220
BmMITE-8	4	262	nscaf2948	1587743	1588003
BmMITE-8	4	262	nscaf2860	1116477	1116219
BmMITE-8	4	262	nscaf2780	10360	10102
BmMITE-8	4	262	nscaf3031	5099905	5100161
BmMITE-8	4	262	nscaf2655	2896732	2896475
BmMITE-8	4	262	nscaf818	10641	10899
BmMITE-8	4	262	nscaf3045	744010	744268
BmMITE-8	4	262	nscaf2767	2611658	2611919
BmMITE-8	4	261	nscaf3040	4389327	4389070
BmMITE-8	4	262	nscaf3031	599247	598974
BmMITE-8	4	260	nscaf3034	456823	456563

BmMITE-8	4	262	nscaf3058	8601871	8602310
BmMITE-8	4	258	nscaf2990	889356	889613
BmMITE-8	4	261	nscaf2767	75165	74924
BmMITE-8	4	261	scaffold128	1656	1415
BmMITE-8	4	262	nscaf2853	4558356	4560047
BmMITE-8	4	266	nscaf2795	763644	763881
BmMITE-8	4	266	nscaf3003	3893902	3893665
BmMITE-8	4	266	nscaf2822	1028813	1029439
BmMITE-8	4	266	nscaf2529	4675991	4675753
BmMITE-8	4	266	nscaf2865	496052	495790
BmMITE-8	4	265	nscaf2511	3159464	3159700
BmMITE-8	4	265	nscaf3058	1312653	1312417
BmMITE-8	4	265	nscaf2330	542458	542699
BmMITE-8	4	265	nscaf2330	3167820	3168058
BmMITE-8	4	265	nscaf3054	21527	21290
BmMITE-8	4	265	nscaf2655	976978	977215
BmMITE-8	4	265	nscaf2852	1735855	1736095
BmMITE-8	4	264	nscaf2865	5483029	5482794
BmMITE-8	4	265	nscaf2986	1946398	1946636
BmMITE-8	4	262	nscaf2829	3742196	3741694
BmMITE-8	4	262	nscaf2930	3412523	3412288
BmMITE-8	4	262	nscaf2789	788481	788078
BmMITE-8	4	262	nscaf2828	2244707	2244474
BmMITE-8	4	266	nscaf2852	1777008	1776736
BmMITE-8	4	262	nscaf125	10306	214
BmMITE-8	4	261	nscaf2931	705021	704785
BmMITE-8	4	261	nscaf2876	810869	811101
BmMITE-8	4	262	nscaf3027	842660	843101
BmMITE-8	4	261	nscaf3058	2052410	2052174
BmMITE-8	4	259	nscaf3072	1770475	1770241
BmMITE-8	4	262	nscaf2827	2085791	2086027
BmMITE-8	4	266	nscaf2902	10599614	10599102
BmMITE-8	4	242	nscaf2818	3069938	3070594
BmMITE-8	4	258	nscaf3072	2478848	2479119
BmMITE-8	4	237	nscaf2948	660161	660393
BmMITE-8	4	250	nscaf2986	714475	714698
BmMITE-8	4	262	nscaf2823	2674149	2674387
BmMITE-8	4	265	nscaf2136	3221121	3220895
BmMITE-8	4	265	nscaf3058	7961553	7961313
BmMITE-8	4	234	nscaf3062	16061	16267
BmMITE-8	4	262	nscaf2136	6851184	6851403
BmMITE-8	4	262	nscaf2931	353537	353303
BmMITE-8	5	266	nscaf2889	3036395	3036134

BmMITE-8	5	266	nscaf1898	8911218	8911479
BmMITE-8	5	266	nscaf2888	6676442	6676181
BmMITE-8	5	266	nscaf2986	2059863	2059602
BmMITE-8	5	266	nscaf2964	2327728	2326817
BmMITE-8	5	266	nscaf1705	1344634	1344374
BmMITE-8	5	266	nscaf2330	2229154	2229416
BmMITE-8	5	266	nscaf2993	4784013	4783752
BmMITE-8	5	266	nscaf2655	2391371	2391109
BmMITE-8	5	265	nscaf2853	2238770	2239032
BmMITE-8	5	265	nscaf3034	2969452	2969192
BmMITE-8	5	265	nscaf3034	2279510	2279250
BmMITE-8	5	265	nscaf2800	1458099	1457839
BmMITE-8	5	265	nscaf1681	3915257	3914998
BmMITE-8	5	265	nscaf2943	2926941	2926681
BmMITE-8	5	265	nscaf3068	69852	70112
BmMITE-8	5	265	nscaf2855	4596476	4596213
BmMITE-8	5	263	nscaf3026	107503	107245
BmMITE-8	5	263	nscaf1690	1335956	1336208
BmMITE-8	5	263	nscaf1690	2688504	2688246
BmMITE-8	5	263	nscaf2853	765907	766165
BmMITE-8	5	263	nscaf2136	5672345	5672087
BmMITE-8	5	263	nscaf1898	12130685	12130941
BmMITE-8	5	263	nscaf2902	4161202	4160944
BmMITE-8	5	266	nscaf2176	395918	396656
BmMITE-8	5	263	nscaf2511	3519145	3519403
BmMITE-8	5	263	nscaf2511	3458922	3458664
BmMITE-8	5	263	nscaf3072	1539259	1539517
BmMITE-8	5	263	nscaf3072	930434	930176
BmMITE-8	5	263	nscaf3097	491134	491394
BmMITE-8	5	263	nscaf2810	361441	361183
BmMITE-8	5	263	nscaf3031	4465680	4465396
BmMITE-8	5	263	nscaf2873	80858	81117
BmMITE-8	5	263	nscaf2767	823738	823480
BmMITE-8	5	263	nscaf2529	4660330	4660591
BmMITE-8	5	262	nscaf2686	1011617	1011360
BmMITE-8	5	262	nscaf2829	4092827	4092569
BmMITE-8	5	262	nscaf1690	1033403	1033146
BmMITE-8	5	262	nscaf2970	2331808	2332065
BmMITE-8	5	262	nscaf2136	4564408	4564668
BmMITE-8	5	262	nscaf1898	5882648	5882905
BmMITE-8	5	262	nscaf2210	3426246	3425989
BmMITE-8	5	262	scaffold426	329	72
BmMITE-8	5	262	nscaf2964	1205799	1205543

BmMITE-8	5	262	nscaf481	1195782	1196038
BmMITE-8	5	262	nscaf3035	1502586	1502328
BmMITE-8	5	262	nscaf3031	5229471	5229212
BmMITE-8	5	262	nscaf2655	1789676	1789421
BmMITE-8	5	262	scaffold265	25	283
BmMITE-8	5	261	nscaf2901	426570	426826
BmMITE-8	5	262	nscaf2853	4569450	4569144
BmMITE-8	5	257	nscaf2330	184691	184943
BmMITE-8	5	265	nscaf2993	1066571	1066847
BmMITE-8	5	254	nscaf3031	1767005	1766757
BmMITE-8	5	266	nscaf2903	1281696	1281933
BmMITE-8	5	252	nscaf2853	6223380	6223627
BmMITE-8	5	266	nscaf2902	3486397	3486633
BmMITE-8	5	266	nscaf3099	2641772	2642011
BmMITE-8	5	266	nscaf2822	1489264	1489511
BmMITE-8	5	266	nscaf2797	600927	600691
BmMITE-8	5	265	nscaf1898	2259614	2259850
BmMITE-8	5	265	nscaf3003	2546269	2546505
BmMITE-8	5	265	nscaf2210	4717555	4717792
BmMITE-8	5	265	nscaf2210	4892038	4891802
BmMITE-8	5	251	scaffold389	247	1
BmMITE-8	5	265	nscaf2176	3969449	3969213
BmMITE-8	5	265	nscaf2800	598165	598401
BmMITE-8	5	265	nscaf2674	6123831	6124066
BmMITE-8	5	265	nscaf1681	1231080	1231316
BmMITE-8	5	265	nscaf3044	1286331	1286094
BmMITE-8	5	265	nscaf2204	3051408	3051645
BmMITE-8	5	265	nscaf98	364988	364752
BmMITE-8	5	265	nscaf2868	330889	331127
BmMITE-8	5	265	nscaf2529	2292355	2292122
BmMITE-8	5	265	scaffold120	190	426
BmMITE-8	5	264	nscaf2589	3390489	3390254
BmMITE-8	5	265	nscaf2998	1468381	1468618
BmMITE-8	5	263	nscaf2795	1924636	1924869
BmMITE-8	5	263	nscaf2970	2660102	2661045
BmMITE-8	5	263	nscaf2931	1496724	1496958
BmMITE-8	5	263	nscaf2902	7928316	7928551
BmMITE-8	5	263	nscaf2964	496329	496096
BmMITE-8	5	263	nscaf3058	1814231	1808594
BmMITE-8	5	263	nscaf3031	3775397	3775163
BmMITE-8	5	263	nscaf2943	2265420	2265186
BmMITE-8	5	263	nscaf3032	114390	114157
BmMITE-8	5	263	nscaf2852	1076443	1076210

BmMITE-8	5	263	nscaf3075	320502	320275
BmMITE-8	5	262	nscaf2865	3997747	3997514
BmMITE-8	5	262	nscaf2823	4091498	4091265
BmMITE-8	5	265	nscaf1690	1073558	1073794
BmMITE-8	5	262	nscaf2136	6543301	6543067
BmMITE-8	5	262	nscaf1898	9226157	9225926
BmMITE-8	5	262	nscaf2902	10972239	10972468
BmMITE-8	5	262	nscaf2902	4553262	4553032
BmMITE-8	5	262	nscaf2818	1475334	1475099
BmMITE-8	5	262	nscaf2818	114870	114636
BmMITE-8	5	262	nscaf2800	1556281	1556048
BmMITE-8	5	262	nscaf2842	1826360	1826593
BmMITE-8	5	262	nscaf3044	1456790	1457024
BmMITE-8	5	262	nscaf2993	7024572	7024804
BmMITE-8	5	262	nscaf2905	157494	157259
BmMITE-8	5	261	nscaf2890	1119395	1119627
BmMITE-8	5	263	nscaf2827	398006	397499
BmMITE-8	5	265	nscaf3022	751072	750837
BmMITE-8	5	260	nscaf2983	707261	707493
BmMITE-8	5	262	nscaf2204	3668930	3669164
BmMITE-8	5	259	nscaf3040	840235	840002
BmMITE-8	5	262	nscaf1898	12061357	12061123
BmMITE-8	5	259	nscaf3003	4359532	4359763
BmMITE-8	5	259	nscaf2876	1137762	1137992
BmMITE-8	5	263	nscaf2986	2114803	2114569
BmMITE-8	5	262	nscaf3058	5640434	5640667
BmMITE-8	5	258	nscaf2795	2033816	2033586
BmMITE-8	5	257	nscaf3058	3754851	3754620
BmMITE-8	5	259	nscaf2886	432276	432508
BmMITE-8	5	261	nscaf3026	2227863	2229095
BmMITE-8	5	256	nscaf2847	3817838	3818069
BmMITE-8	5	237	nscaf98	1673850	1674059
BmMITE-8	5	265	nscaf2903	599799	599128
BmMITE-8	5	265	nscaf2204	4020734	4020971
BmMITE-8	5	265	nscaf3031	237280	237044
BmMITE-8	5	263	nscaf2829	3027338	3027104
BmMITE-8	5	263	nscaf3027	5111638	5111394
BmMITE-8	5	233	nscaf2210	4670602	4670806
BmMITE-8	5	262	nscaf2674	3715679	3715934
BmMITE-8	5	265	nscaf3008	1888251	1888016
BmMITE-8	6	266	nscaf2905	466852	466592
BmMITE-8	6	265	nscaf3038	76529	69587
BmMITE-8	6	265	nscaf2986	602084	602342

BmMITE-8	6	265	nscaf2204	2560923	2560672
BmMITE-8	6	264	nscaf3015	1051968	1052226
BmMITE-8	6	264	nscaf2964	683631	683889
BmMITE-8	6	264	nscaf3058	3967768	3967510
BmMITE-8	6	264	nscaf2330	3609345	3609088
BmMITE-8	6	263	nscaf2136	1528697	1528954
BmMITE-8	6	263	scaffold876	11887	12144
BmMITE-8	6	262	nscaf2903	1441183	1440924
BmMITE-8	6	262	nscaf1690	7290504	7290239
BmMITE-8	6	262	nscaf2953	2934037	2934292
BmMITE-8	6	262	nscaf2937	791050	791306
BmMITE-8	6	262	nscaf2818	1650753	1651009
BmMITE-8	6	262	nscaf1705	578004	578259
BmMITE-8	6	262	nscaf3058	8734486	8734230
BmMITE-8	6	262	nscaf3072	458626	458367
BmMITE-8	6	262	nscaf2943	2071008	2070752
BmMITE-8	6	262	scaffold655	80186	79932
BmMITE-8	6	262	nscaf1236	10511	10766
BmMITE-8	6	262	scaffold288	966	1221
BmMITE-8	6	265	nscaf1681	2089444	2088905
BmMITE-8	6	261	nscaf2842	60170	60425
BmMITE-8	6	261	nscaf2847	3587684	3587939
BmMITE-8	6	260	nscaf1898	2534825	2534570
BmMITE-8	6	260	nscaf2868	47881	48417
BmMITE-8	6	262	nscaf2511	3133727	3134291
BmMITE-8	6	266	nscaf3034	2861584	2861821
BmMITE-8	6	266	nscaf3003	3432440	3432675
BmMITE-8	6	265	nscaf1898	12742015	12741780
BmMITE-8	6	265	nscaf3053	148805	148563
BmMITE-8	6	264	nscaf2827	1462656	1462424
BmMITE-8	6	264	nscaf3041	1643396	1643162
BmMITE-8	6	264	nscaf2990	393204	392966
BmMITE-8	6	264	nscaf3040	447366	447600
BmMITE-8	6	266	nscaf2889	1525332	1525097
BmMITE-8	6	262	nscaf2795	556489	556720
BmMITE-8	6	265	nscaf3093	590598	590363
BmMITE-8	6	262	nscaf2829	2323773	2323541
BmMITE-8	6	261	nscaf3079	280025	279797
BmMITE-8	6	262	nscaf2937	1437655	1437887
BmMITE-8	6	264	nscaf2847	3018667	3018433
BmMITE-8	6	257	nscaf2136	97300	97524
BmMITE-8	6	264	nscaf2589	6114139	6113884
BmMITE-8	6	235	scaffold107	518	750

BmMITE-8	6	265	nscaf3035	797658	798159
BmMITE-8	6	228	nscaf2883	2026090	2026312
BmMITE-8	6	266	nscaf2962	695178	694943
BmMITE-8	7	266	nscaf2983	2199523	2199782
BmMITE-8	7	266	scaffold672	1	260
BmMITE-8	7	265	nscaf2853	1776933	1777191
BmMITE-8	7	265	nscaf2800	1617239	1616980
BmMITE-8	7	265	scaffold298	424	166
BmMITE-8	7	262	nscaf2827	724149	723905
BmMITE-8	7	266	nscaf2852	1978124	1978358
BmMITE-8	7	266	nscaf2838	1808464	1808698
BmMITE-8	7	265	nscaf3044	1266054	1265818
BmMITE-8	7	265	nscaf2905	443152	443385
BmMITE-8	7	262	nscaf2829	2054832	2055063
BmMITE-8	7	262	nscaf1898	14922146	14921916
BmMITE-8	7	236	nscaf2810	181578	181373
BmMITE-8	8	266	nscaf2993	2885229	2884972
BmMITE-8	8	266	nscaf2943	3801414	3801160
BmMITE-8	8	265	nscaf3003	4109058	4109316
BmMITE-8	8	265	nscaf2847	2512279	2512536
BmMITE-8	8	262	nscaf2176	3874088	3873834
BmMITE-8	8	262	nscaf2828	5182592	5182338
BmMITE-8	8	261	nscaf2868	226015	225755
BmMITE-8	8	266	nscaf2852	2841248	2841490
BmMITE-8	8	265	nscaf1898	11054461	11053972
BmMITE-8	8	237	nscaf3003	4079441	4079213
BmMITE-8	8	251	nscaf2891	1671960	1672173
BmMITE-8	8	249	nscaf2828	1665319	1665099
BmMITE-8	8	233	nscaf2210	4560744	4560968
BmMITE-8	8	266	nscaf2912	345206	344970
BmMITE-8	8	260	nscaf2575	1381884	1382116
BmMITE-8	9	261	nscaf2846	95098	94848
BmMITE-8	9	265	nscaf3026	2584084	2584314
BmMITE-8	9	266	nscaf2951	2585	2355
BmMITE-8	9	264	nscaf3032	629523	629293
BmMITE-8	9	262	nscaf3097	254362	254589
BmMITE-8	9	262	nscaf3062	339161	338942
BmMITE-8	10	266	nscaf2851	1415492	1415236
BmMITE-8	10	266	nscaf2847	7234397	7234141
BmMITE-8	10	264	scaffold168	1	255
BmMITE-8	10	261	nscaf2822	648882	649127
BmMITE-8	10	259	nscaf2674	6023554	6023787
BmMITE-8	10	266	nscaf2964	2844235	2844467

BmMITE-8	10	266	nscaf3032	927300	927532
BmMITE-8	10	261	nscaf2902	9534381	9534155
BmMITE-8	10	261	nscaf3099	2236722	2236493
BmMITE-8	11	266	scaffold240	1	256
BmMITE-8	11	266	nscaf3040	2095093	2094862
BmMITE-8	11	262	nscaf2948	1215437	1215665
BmMITE-8	11	261	nscaf2833	425432	425658
BmMITE-8	12	265	nscaf3038	116712	116456
BmMITE-8	12	265	nscaf1681	1658006	1658258
BmMITE-8	12	262	scaffold320	1	251
BmMITE-8	12	261	nscaf2891	1063559	1063310
BmMITE-8	12	265	nscaf2330	3990852	3990623
BmMITE-8	12	265	nscaf2961	89626	89395
BmMITE-8	12	244	nscaf2970	970913	970705
BmMITE-8	13	262	nscaf2948	2395291	2395067
BmMITE-8	14	265	nscaf2827	2156391	2156139
BmMITE-8	14	265	nscaf1108	2433150	2432920
BmMITE-8	15	265	nscaf2330	1744199	1743948
BmMITE-8	15	262	nscaf1898	3527942	3527701
BmMITE-8	15	258	nscaf2575	3435139	3434896
BmMITE-8	15	266	nscaf2279	16079	15851
BmMITE-8	16	265	nscaf2990	532175	532397
BmMITE-8	17	265	scaffold430	1	249
BmMITE-8	18	263	nscaf3058	6506901	6506682
BmMITE-8	19	265	nscaf2529	2538698	2538947
BmMITE-8	20	265	nscaf2888	1443809	1444054
BmMITE-8	20	266	nscaf2853	1933710	1933931
BmMITE-8	20	266	nscaf2986	1024158	1024380
BmMITE-8	20	265	nscaf2865	4402185	4402445
BmMITE-8	21	265	nscaf2852	2227345	2227126
BmMITE-8	25	265	nscaf2860	1978650	1978410
BmMITE-8	26	265	nscaf2204	3504128	3504362
BmMITE-8	26	265	nscaf2914	60832	60590
BmMITE-8	26	263	nscaf2136	6023191	6022954
BmMITE-8	26	263	scaffold428	2	239
BmMITE-8	26	266	nscaf2829	3115420	3115635
BmMITE-8	26	266	nscaf2529	1476704	1476490
BmMITE-8	26	265	nscaf2865	618394	618179
BmMITE-8	26	265	nscaf2828	5828477	5828693
BmMITE-8	26	263	nscaf2136	570477	570689
BmMITE-8	26	262	nscaf1690	5897747	5898103
BmMITE-8	27	265	nscaf3097	1253888	1254124
BmMITE-8	27	265	scaffold416	457	219

BmMITE-8	27	266	nscaf2828	2356492	2356274
BmMITE-8	27	265	nscaf2953	1121577	1121363
BmMITE-8	28	265	nscaf2888	893240	893453
BmMITE-8	29	265	nscaf3080	7891	7678
BmMITE-8	30	266	nscaf3098	472677	472887
BmMITE-8	30	266	nscaf2810	523170	523385
BmMITE-8	30	265	nscaf3045	1131018	1131230
BmMITE-8	30	265	nscaf2529	1432110	1431899
BmMITE-8	30	262	nscaf2970	1774992	1775199
BmMITE-8	31	265	nscaf2136	5067474	5067264
BmMITE-8	31	262	nscaf2948	2059997	2059790
BmMITE-8	32	265	nscaf3140	18422	18655
BmMITE-8	32	265	nscaf3048	289295	289505
BmMITE-8	34	265	nscaf2564	360317	360525
BmMITE-8	35	265	nscaf1690	890559	890789
BmMITE-8	35	265	nscaf2993	2146341	2146571
BmMITE-8	35	265	nscaf2053	96051	95822
BmMITE-8	36	266	nscaf2853	6540140	6539933
BmMITE-8	38	265	scaffold408	1	228
BmMITE-8	38	265	nscaf481	1157694	1157897
BmMITE-8	39	262	nscaf2815	1128298	1128061
BmMITE-8	39	265	nscaf1898	1345304	1345507
BmMITE-8	41	266	nscaf3003	2274951	2274726
BmMITE-8	45	262	scaffold316	47810	48028
BmMITE-8	46	265	scaffold392	518	299
BmMITE-8	50	266	scaffold413	1	217
BmMITE-8	51	266	nscaf2366	26022	25803
BmMITE-8	51	265	nscaf2216	653607	653821
BmMITE-8	51	265	scaffold331	9	223
BmMITE-8	52	265	nscaf463	1591331	1591118
BmMITE-8	52	265	nscaf2529	3857567	3857780
BmMITE-8	54	266	nscaf3079	519258	519470
BmMITE-8	54	266	nscaf2176	1391295	1391083
BmMITE-8	1	265	nscaf3026	3959824	3960087
BmMITE-8	1	265	nscaf3026	4052456	4052720
BmMITE-8	1	265	nscaf3026	4972313	4972055
BmMITE-8	1	265	nscaf3026	4961413	4961150
BmMITE-8	1	265	nscaf3026	4126087	4125824
BmMITE-8	1	265	nscaf3026	981799	981531
BmMITE-8	1	265	nscaf2890	1305123	1305393
BmMITE-8	1	265	nscaf3043	165409	165673
BmMITE-8	1	265	nscaf3040	3337988	3338252
BmMITE-8	1	265	nscaf2903	1177372	1177636

BmMITE-8	1	265	nscaf2903	1301180	1300919
BmMITE-8	1	265	nscaf2865	4170260	4170793
BmMITE-8	1	265	nscaf2829	3417994	3418255
BmMITE-8	1	265	nscaf2829	1747631	1747370
BmMITE-8	1	265	nscaf2829	1431644	1431381
BmMITE-8	1	265	nscaf2829	1350530	1350266
BmMITE-8	1	265	nscaf2823	2454079	2454343
BmMITE-8	1	265	nscaf2623	574904	575167
BmMITE-8	1	265	nscaf1690	4494403	4494668
BmMITE-8	1	265	nscaf1690	2044555	2044294
BmMITE-8	1	265	scaffold406	205	469
BmMITE-8	1	265	nscaf2795	347859	348123
BmMITE-8	1	265	nscaf2795	2381996	2382261
BmMITE-8	1	265	nscaf2795	2990199	2989935
BmMITE-8	1	265	nscaf2759	120717	120981
BmMITE-8	1	265	nscaf3066	736504	736247
BmMITE-8	1	265	nscaf2970	671394	671131
BmMITE-8	1	265	nscaf2136	3747256	3747520
BmMITE-8	1	265	nscaf1898	3672675	3672936
BmMITE-8	1	265	nscaf1898	1949580	1949317
BmMITE-8	1	265	scaffold164	215	479
BmMITE-8	1	265	nscaf3093	630764	630500
BmMITE-8	1	265	nscaf3003	4591790	4591526
BmMITE-8	1	265	nscaf3003	4295338	4295074
BmMITE-8	1	265	nscaf2953	3556001	3556262
BmMITE-8	1	265	nscaf2937	774216	774478
BmMITE-8	1	265	nscaf2937	1034564	1034833
BmMITE-8	1	265	nscaf2931	53266	53531
BmMITE-8	1	265	nscaf2931	615616	615353
BmMITE-8	1	265	nscaf2902	987088	987488
BmMITE-8	1	265	nscaf2902	1742467	1742732
BmMITE-8	1	265	nscaf2902	6777861	6778124
BmMITE-8	1	265	nscaf2902	1894054	1893790
BmMITE-8	1	265	nscaf2888	1526317	1526580
BmMITE-8	1	265	nscaf2888	3944029	3944293
BmMITE-8	1	265	nscaf2888	2944158	2943894
BmMITE-8	1	265	nscaf2818	1160755	1161009
BmMITE-8	1	265	nscaf2818	2631958	2632222
BmMITE-8	1	265	nscaf2818	888412	888150
BmMITE-8	1	265	nscaf2818	502103	501836
BmMITE-8	1	265	nscaf3055	429521	429879
BmMITE-8	1	265	nscaf3055	441039	441301
BmMITE-8	1	265	nscaf2176	2854694	2854962

BmMITE-8	1	265	nscaf3027	3828642	3828898
BmMITE-8	1	265	nscaf2986	1311669	1311932
BmMITE-8	1	265	nscaf2986	1626809	1626545
BmMITE-8	1	265	nscaf2964	698215	698478
BmMITE-8	1	265	nscaf2964	2767845	2768106
BmMITE-8	1	265	nscaf2964	4884453	4884718
BmMITE-8	1	265	nscaf2800	2130533	2130805
BmMITE-8	1	265	nscaf2800	1475800	1473655
BmMITE-8	1	265	nscaf2674	4101458	4101195
BmMITE-8	1	265	nscaf2674	455074	454811
BmMITE-8	1	265	nscaf2511	50557	50293
BmMITE-8	1	265	nscaf1681	3939275	3939540
BmMITE-8	1	265	nscaf1681	1838494	1838230
BmMITE-8	1	265	nscaf3058	6900063	6900330
BmMITE-8	1	265	nscaf3058	6413264	6413004
BmMITE-8	1	265	nscaf3072	2668873	2668608
BmMITE-8	1	265	nscaf3035	57730	57466
BmMITE-8	1	265	nscaf2886	1177240	1176977
BmMITE-8	1	265	nscaf2886	541067	540803
BmMITE-8	1	265	nscaf2883	842530	842266
BmMITE-8	1	265	nscaf2789	553471	553735
BmMITE-8	1	265	nscaf2789	615099	614722
BmMITE-8	1	265	nscaf3099	2041989	2042255
BmMITE-8	1	265	nscaf3097	2509924	2510188
BmMITE-8	1	265	nscaf2948	1372783	1371277
BmMITE-8	1	265	nscaf2822	892602	892866
BmMITE-8	1	265	nscaf2822	1421231	1421499
BmMITE-8	1	265	nscaf2822	2117240	2117503
BmMITE-8	1	265	nscaf2589	992652	992912
BmMITE-8	1	265	nscaf2204	1503241	1503506
BmMITE-8	1	265	nscaf2204	2794605	2794333
BmMITE-8	1	265	nscaf2204	850605	850341
BmMITE-8	1	265	nscaf2860	1638859	1644338
BmMITE-8	1	265	nscaf2770	1099057	1099317
BmMITE-8	1	265	nscaf2891	436776	437038
BmMITE-8	1	265	nscaf2891	526977	526711
BmMITE-8	1	265	nscaf98	1558209	1557945
BmMITE-8	1	265	nscaf3031	3319498	3319762
BmMITE-8	1	265	nscaf2980	440848	441112
BmMITE-8	1	265	nscaf2828	4435441	4435705
BmMITE-8	1	265	nscaf2943	1167093	1166825
BmMITE-8	1	265	scaffold340	17	281
BmMITE-8	1	265	nscaf3060	109537	109275

BmMITE-8	1	265	nscaf3062	135498	135760
BmMITE-8	1	265	scaffold312	436	172
BmMITE-8	1	265	nscaf3080	64320	64578
BmMITE-8	1	265	nscaf2529	1246392	1246687
BmMITE-8	1	265	nscaf2986	3915464	3916446
BmMITE-8	1	265	nscaf2970	462060	462454
BmMITE-8	1	265	nscaf2883	1717713	1717961
BmMITE-8	1	265	nscaf2780	326157	325909
BmMITE-8	1	265	nscaf2905	84883	84635
BmMITE-8	1	265	nscaf3062	325455	325703
BmMITE-8	1	265	nscaf2529	1960896	1961151
BmMITE-8	1	265	nscaf3129	64473	64225
BmMITE-8	1	265	nscaf1690	2799290	2800147
BmMITE-8	1	265	nscaf3003	3746956	3747448
BmMITE-8	1	265	nscaf2330	1033702	1032982
BmMITE-8	1	265	nscaf2674	7942376	7942129
BmMITE-8	1	265	nscaf2767	497801	498070
BmMITE-8	1	265	nscaf2865	340941	341181
BmMITE-8	1	265	nscaf2829	3787904	3787664
BmMITE-8	1	265	nscaf2623	533874	533632
BmMITE-8	1	265	nscaf1690	7365450	7365209
BmMITE-8	1	265	nscaf3048	61806	61575
BmMITE-8	1	265	nscaf2970	1965463	1965701
BmMITE-8	1	265	nscaf2970	1998281	1998521
BmMITE-8	1	265	nscaf2970	1587844	1587605
BmMITE-8	1	265	nscaf2853	2588863	2588623
BmMITE-8	1	265	nscaf2136	4626278	4626038
BmMITE-8	1	265	nscaf1898	11273333	11273574
BmMITE-8	1	265	nscaf1898	13345385	13345147
BmMITE-8	1	265	nscaf1898	6838553	6837104
BmMITE-8	1	265	nscaf2953	3171656	3171895
BmMITE-8	1	265	nscaf2902	9421677	9421921
BmMITE-8	1	265	nscaf2888	2457111	2457351
BmMITE-8	1	265	nscaf2888	5395241	5395483
BmMITE-8	1	265	nscaf2888	8939341	8939101
BmMITE-8	1	265	nscaf2888	8630051	8629811
BmMITE-8	1	265	nscaf2818	1350377	1350136
BmMITE-8	1	265	nscaf2210	2193111	2193353
BmMITE-8	1	265	nscaf2210	895987	895750
BmMITE-8	1	265	nscaf2176	4226866	4226626
BmMITE-8	1	265	nscaf3015	3897080	3897319
BmMITE-8	1	265	nscaf2986	4074097	4074341
BmMITE-8	1	265	nscaf2964	1547983	1547743

BmMITE-8	1	265	nscaf2800	1846737	1846981
BmMITE-8	1	265	nscaf481	353670	353906
BmMITE-8	1	265	nscaf2674	1571957	1571716
BmMITE-8	1	265	nscaf2511	2190122	2190361
BmMITE-8	1	265	nscaf2511	6111112	6111351
BmMITE-8	1	265	nscaf1681	5793220	5793459
BmMITE-8	1	265	nscaf2983	3224087	3224327
BmMITE-8	1	265	nscaf2930	6007825	6008066
BmMITE-8	1	265	nscaf3058	6757909	6757671
BmMITE-8	1	265	nscaf3058	2293414	2293176
BmMITE-8	1	265	nscaf3035	678884	678644
BmMITE-8	1	265	nscaf2883	1367186	1367425
BmMITE-8	1	265	nscaf2789	1465739	1465979
BmMITE-8	1	265	nscaf3099	1753446	1753689
BmMITE-8	1	265	nscaf3097	2148107	2147866
BmMITE-8	1	265	nscaf3063	2848760	2849002
BmMITE-8	1	265	nscaf2993	1104879	1105122
BmMITE-8	1	265	nscaf2993	7670373	7670613
BmMITE-8	1	265	nscaf2993	7510367	7510128
BmMITE-8	1	265	nscaf2860	102677	102918
BmMITE-8	1	265	nscaf2770	286509	286748
BmMITE-8	1	265	nscaf2770	449450	449687
BmMITE-8	1	265	nscaf2891	1260254	1260493
BmMITE-8	1	265	nscaf2780	139012	139252
BmMITE-8	1	265	nscaf2655	2495499	2495741
BmMITE-8	1	265	nscaf2655	3252804	3252563
BmMITE-8	1	265	nscaf2847	7944884	7945123
BmMITE-8	1	265	nscaf2847	4650003	4649762
BmMITE-8	1	265	nscaf2943	744979	745218
BmMITE-8	1	265	nscaf2825	224078	224318
BmMITE-8	1	265	nscaf2767	2370224	2369984
BmMITE-8	1	265	nscaf2575	4310109	4310349
BmMITE-8	1	265	nscaf3060	63407	63647
BmMITE-8	1	265	nscaf2797	338238	338480
BmMITE-8	1	265	nscaf2868	258567	258807
BmMITE-8	1	265	nscaf2852	2234368	2234608
BmMITE-8	1	265	nscaf2529	2905944	2905704
BmMITE-8	1	265	nscaf3056	107333	107083
BmMITE-8	1	265	nscaf2136	5089810	5090050
BmMITE-8	1	265	nscaf2330	1585806	1585567
BmMITE-8	1	265	nscaf3066	1477376	1477618
BmMITE-8	1	265	nscaf481	1254590	1248370
BmMITE-8	1	265	nscaf2330	3150344	3150586

BmMITE-8	1	265	nscaf1681	5811693	5811453
BmMITE-8	1	265	nscaf2767	4298579	4298819
BmMITE-8	1	265	nscaf2888	4014864	4014623
BmMITE-8	1	265	nscaf2986	5043600	5044701
BmMITE-8	1	265	nscaf2855	2513634	2514263
BmMITE-8	1	265	nscaf2853	1288671	1288910
BmMITE-8	1	265	scaffold201	2036	1584
BmMITE-8	1	265	nscaf2937	1046917	1046676
BmMITE-8	1	265	nscaf2962	759069	758825
BmMITE-8	1	265	nscaf2795	1689333	1688717
BmMITE-8	1	265	nscaf3027	650812	650192
BmMITE-8	1	265	nscaf2529	5567358	5567103
BmMITE-8	1	265	nscaf2210	4763667	4763906
BmMITE-8	1	265	nscaf2889	1941566	1941810
BmMITE-8	1	265	nscaf3058	2840764	2840531
BmMITE-8	1	265	nscaf3056	1036619	1036869
BmMITE-8	1	265	nscaf1690	2714728	2714502
BmMITE-8	1	265	nscaf2930	5884732	5884967
BmMITE-8	1	265	nscaf2912	555069	554826
BmMITE-8	1	265	nscaf2847	2353045	2352456
BmMITE-8	1	265	nscaf1690	2505861	2505618
BmMITE-8	1	265	nscaf1690	1935281	1935042
BmMITE-8	1	265	nscaf2136	4523268	4523026
BmMITE-8	1	265	nscaf2210	4398259	4398023
BmMITE-8	1	265	nscaf2964	2236505	2236745
BmMITE-8	1	265	nscaf2589	1148760	1148518
BmMITE-8	1	265	nscaf2589	721228	720986
BmMITE-8	1	265	nscaf2053	237253	237012
BmMITE-8	1	265	nscaf3013	948442	948231
BmMITE-8	1	265	nscaf2830	25558	25796
BmMITE-8	1	265	nscaf3089	325428	325663
BmMITE-8	1	265	nscaf2674	7227086	7227310
BmMITE-8	1	265	nscaf463	35714	35952
BmMITE-8	1	265	nscaf1108	1304199	1304406
BmMITE-8	1	265	nscaf3058	6180743	6180509
BmMITE-8	1	265	nscaf3003	460622	460865
BmMITE-8	1	265	nscaf2888	5868623	5868384
BmMITE-8	1	265	nscaf2853	850272	850514
BmMITE-8	1	265	nscaf2860	2339578	2339784
BmMITE-8	1	265	nscaf2767	2512069	2511831
BmMITE-8	1	265	nscaf2529	2500561	2500801
BmMITE-8	1	265	nscaf3040	1293964	1288773
BmMITE-8	1	264	nscaf1690	5132694	5132407

BmMITE-8	1	264	nscaf2970	611618	611881
BmMITE-8	1	264	nscaf2930	6209143	6209406
BmMITE-8	1	264	nscaf2951	318711	318448
BmMITE-8	1	264	nscaf3097	1157241	1156979
BmMITE-8	1	264	nscaf1898	11234139	11234371
BmMITE-8	1	264	nscaf2930	1139669	1139429
BmMITE-8	1	263	nscaf2136	6795714	6795976
BmMITE-8	1	263	nscaf2983	1282319	1274364
BmMITE-8	1	263	nscaf2901	1423926	1424188
BmMITE-8	1	263	nscaf2838	489969	490207
BmMITE-8	1	263	nscaf2829	2912827	2912587
BmMITE-8	1	263	nscaf2204	4237798	4238037
BmMITE-8	1	263	nscaf3022	23578	23816
BmMITE-8	1	263	nscaf2829	695694	695931
BmMITE-8	1	263	nscaf2770	1079495	1079734
BmMITE-8	1	263	nscaf2511	4211148	4211635
BmMITE-8	1	262	nscaf2136	7902505	7902766
BmMITE-8	1	262	nscaf3034	1899733	1900413
BmMITE-8	1	262	nscaf3034	2236761	2236500
BmMITE-8	1	262	nscaf2888	7182202	7181941
BmMITE-8	1	262	nscaf3098	1018731	1018469
BmMITE-8	1	262	scaffold430	21	282
BmMITE-8	1	262	nscaf3015	2576533	2576272
BmMITE-8	1	262	nscaf2964	2327467	2327728
BmMITE-8	1	262	nscaf1690	6047358	6047121
BmMITE-8	1	262	nscaf2853	1715134	1715374
BmMITE-8	1	262	nscaf2902	6604774	6604538
BmMITE-8	1	262	nscaf2818	1591615	1591377
BmMITE-8	1	262	nscaf2204	2579174	2578942
BmMITE-8	1	262	nscaf2980	1056287	1056047
BmMITE-8	1	262	nscaf3062	290410	290173
BmMITE-8	1	262	nscaf2804	13034	13270
BmMITE-8	1	262	nscaf2905	452695	452456
BmMITE-8	1	261	nscaf3026	1242120	1241884
BmMITE-8	1	261	nscaf481	999225	998988
BmMITE-8	1	261	nscaf2912	1194620	1194853
BmMITE-8	1	261	nscaf2888	7145712	7145476
BmMITE-8	1	261	nscaf1690	2107579	2107814
BmMITE-8	1	260	nscaf3104	15599	15360
BmMITE-8	1	260	nscaf2800	660430	659972
BmMITE-8	1	260	nscaf2398	314640	314404
BmMITE-8	1	259	nscaf2136	5720159	5720564
BmMITE-8	1	259	nscaf3058	5427967	5427714

BmMITE-8	1	259	nscaf3027	1764057	1766585
BmMITE-8	1	258	nscaf2767	1685291	1684563
BmMITE-8	1	257	nscaf2865	2185248	2185478
BmMITE-8	1	257	nscaf2853	5923161	5923393
BmMITE-8	1	256	nscaf2964	2861106	2860850
BmMITE-8	1	256	nscaf3072	2088314	2088059
BmMITE-8	1	254	nscaf2948	1040042	1040297
BmMITE-8	1	254	nscaf3072	1298536	1298278
BmMITE-8	1	253	nscaf2887	481426	481677
BmMITE-8	1	253	scaffold494	254	1
BmMITE-8	1	246	nscaf2888	77069	77290
BmMITE-8	1	241	nscaf3045	2154496	2154257
BmMITE-8	1	237	nscaf2855	6353730	6353965
BmMITE-8	1	236	nscaf2865	4610230	4610016
BmMITE-8	1	236	nscaf1690	1941011	1941221
BmMITE-8	1	234	nscaf3035	590661	590893
BmMITE-8	1	233	nscaf2888	835643	835848
BmMITE-8	1	233	nscaf2916	37176	36967
BmMITE-8	1	231	nscaf2818	2221010	2221242
BmMITE-8	1	231	nscaf2860	1243031	1243261
BmMITE-8	1	227	nscaf2902	4105628	4105854
BmMITE-8	1	218	nscaf2511	562274	562053
BmMITE-9	2	418	nscaf3066	889151	888735
BmMITE-9	2	418	nscaf1299	316653	317059
BmMITE-9	2	418	nscaf2887	1276951	1276536
BmMITE-9	3	418	nscaf3026	5710446	5710857
BmMITE-9	3	418	nscaf2888	3495919	3495500
BmMITE-9	3	418	nscaf2986	2333425	2333009
BmMITE-9	3	418	nscaf2623	703581	703167
BmMITE-9	3	418	nscaf2780	19000	19409
BmMITE-9	3	418	nscaf3119	11910	11230
BmMITE-9	3	417	nscaf3058	7422547	7422133
BmMITE-9	3	418	nscaf2853	5481068	5480332
BmMITE-9	3	354	nscaf3058	6488476	6488126
BmMITE-9	4	418	nscaf2886	278063	278480
BmMITE-9	5	418	nscaf2828	4044260	4043844
BmMITE-9	5	418	nscaf2818	862848	862435
BmMITE-9	6	418	nscaf2954	38448	38037
BmMITE-9	7	418	nscaf2930	6281553	6281964
BmMITE-9	7	414	nscaf3071	215157	214754
BmMITE-9	8	418	nscaf1898	2369529	2369119
BmMITE-9	11	389	nscaf1485	30631	31009

BmMITE-9	12	418	nscaf2204	4297760	4297354
BmMITE-9	13	418	nscaf3040	1166833	1167238
BmMITE-9	13	418	nscaf2801	151505	151910
BmMITE-9	21	418	nscaf2865	1944947	1945317
BmMITE-9	21	418	nscaf3026	4186901	4187252
BmMITE-9	22	418	nscaf98	1014120	1014522
BmMITE-9	29	418	scaffold325	587	198
BmMITE-9	31	418	nscaf3026	5574540	5574153
BmMITE-9	42	389	nscaf2993	7988081	7987568
BmMITE-9	45	414	nscaf3040	1364455	1364111
BmMITE-9	46	418	nscaf2964	4396795	4397170
BmMITE-9	60	418	nscaf2176	708455	708098
BmMITE-9	69	418	nscaf2883	2521632	2521980
BmMITE-9	1	417	nscaf2686	740690	741106
BmMITE-9	1	417	scaffold107	229	645
BmMITE-9	1	417	nscaf2964	617346	615815
BmMITE-9	1	417	scaffold369	733	320
BmMITE-9	1	416	nscaf3034	2441943	2441528
BmMITE-9	1	416	nscaf3040	1570958	1571373
BmMITE-9	1	416	nscaf3089	386784	385947
BmMITE-9	1	416	nscaf2795	636653	636246
BmMITE-9	1	415	nscaf2176	1165118	1165531
BmMITE-9	1	415	nscaf3062	334807	334404
BmMITE-9	1	415	nscaf2962	1061642	1061228
BmMITE-9	1	414	nscaf3162	4064	4477
BmMITE-9	1	414	nscaf2986	2687818	2689105
BmMITE-9	1	414	scaffold313	710	297
BmMITE-9	1	414	nscaf3110	60555	60968
BmMITE-9	1	414	nscaf3045	79878	80291
BmMITE-9	1	414	nscaf2912	1444175	1443333
BmMITE-9	1	414	nscaf2829	860698	860069
BmMITE-9	1	414	nscaf1743	20430	19797
BmMITE-9	1	414	nscaf2826	38509	36504
BmMITE-9	1	414	nscaf2852	225962	225239
BmMITE-9	1	412	nscaf2993	7345008	7345419
BmMITE-9	1	408	nscaf2655	2607816	2608226
BmMITE-9	1	407	nscaf2655	2341472	2341878
BmMITE-9	1	407	scaffold442	640	1046
BmMITE-9	1	406	nscaf2829	3865904	3865506
BmMITE-9	1	400	nscaf2827	1065340	1064942
BmMITE-9	1	400	nscaf2770	2321654	2320838
BmMITE-9	1	390	nscaf1898	14984451	14984853
BmMITE-9	1	389	nscaf2888	8600559	8601557

BmMITE-9	1	389	nscaf2204	1871322	1871889
BmMITE-9	1	389	nscaf2793	30737	30349
BmMITE-9	1	389	nscaf3056	1392474	1392856
BmMITE-9	1	389	nscaf2943	2230980	2231368
BmMITE-9	1	388	nscaf2053	441259	441767
BmMITE-9	1	385	nscaf2912	1244855	1244471
BmMITE-9	1	380	nscaf2564	74137	74514
BmMITE-9	1	372	nscaf2931	996616	996987
BmMITE-9	1	358	nscaf3054	165196	165553
BmMITE-9	1	355	scaffold29	251	605
BmMITE-9	1	350	nscaf3162	26638	26294
BmMITE-9	1	341	nscaf3050	42275	42615
BmMITE-10	2	258	nscaf2852	1572881	1572620
BmMITE-10	2	258	nscaf1898	3535048	3535300
BmMITE-10	2	258	nscaf2176	2391068	2390813
BmMITE-10	2	258	nscaf2511	3906673	3906421
BmMITE-10	2	258	nscaf2823	1670659	1670391
BmMITE-10	2	253	nscaf2986	5142301	5142550
BmMITE-10	2	258	nscaf2797	1036836	1037280
BmMITE-10	2	258	nscaf3015	405568	405310
BmMITE-10	2	258	nscaf2681	754680	754915
BmMITE-10	2	258	nscaf2529	4354620	4354878
BmMITE-10	2	258	nscaf1705	950281	950042
BmMITE-10	2	256	nscaf3003	3728590	3728327
BmMITE-10	3	258	nscaf463	434780	434528
BmMITE-10	3	258	nscaf2902	6293163	6292911
BmMITE-10	3	258	nscaf2136	5513910	5514161
BmMITE-10	3	258	nscaf2828	343831	343576
BmMITE-10	3	258	nscaf3048	1324713	1324967
BmMITE-10	3	258	nscaf1108	1601084	1600833
BmMITE-10	3	258	nscaf1898	1845737	1845986
BmMITE-10	3	258	nscaf2888	1771587	1771336
BmMITE-10	3	258	nscaf2865	1940098	1939316
BmMITE-10	3	258	nscaf3071	888036	888274
BmMITE-10	3	256	nscaf2983	2707163	2706914
BmMITE-10	3	258	nscaf3026	6466870	6467249
BmMITE-10	3	258	nscaf2961	11810	12058
BmMITE-10	4	258	nscaf2330	77413	77664
BmMITE-10	4	258	nscaf3027	4831713	4831963
BmMITE-10	4	258	nscaf2838	1877371	1877623
BmMITE-10	5	258	nscaf1898	417355	417108
BmMITE-10	5	258	nscaf3027	4142705	4142453

BmMITE-10	5	258	nscaf2674	5108845	5109095
BmMITE-10	5	258	nscaf773	7717	7467
BmMITE-10	5	258	nscaf2887	534502	534745
BmMITE-10	5	258	nscaf2891	675836	676086
BmMITE-10	5	258	nscaf2887	1186966	1186721
BmMITE-10	5	258	nscaf2767	3232219	3232468
BmMITE-10	5	258	nscaf3003	4876779	4876530
BmMITE-10	5	258	nscaf2847	5032117	5031606
BmMITE-10	5	258	nscaf2136	4764734	4764981
BmMITE-10	5	258	nscaf2888	7865563	7865314
BmMITE-10	6	246	nscaf2887	1313622	1313390
BmMITE-10	6	258	nscaf2674	6419622	6419870
BmMITE-10	7	258	nscaf2210	1406420	1406174
BmMITE-10	7	258	nscaf1108	1627631	1627882
BmMITE-10	7	258	nscaf2851	49350	49595
BmMITE-10	7	256	nscaf3005	1846791	1846539
BmMITE-10	7	258	nscaf2210	1864181	1864718
BmMITE-10	7	257	nscaf2136	7136887	7137140
BmMITE-10	7	248	nscaf3035	1027503	1027258
BmMITE-10	7	253	nscaf2210	2797255	2797804
BmMITE-10	7	243	nscaf3040	1688925	1689158
BmMITE-10	7	258	nscaf3098	2058169	2057908
BmMITE-10	8	256	nscaf2792	38252	38496
BmMITE-10	8	258	nscaf2136	103089	102719
BmMITE-10	8	258	nscaf1898	7323550	7323814
BmMITE-10	8	258	nscaf3032	1476664	1476914
BmMITE-10	9	258	nscaf3026	3602345	3602097
BmMITE-10	9	258	nscaf2905	462994	462749
BmMITE-10	9	257	nscaf2865	2501643	2501889
BmMITE-10	9	254	scaffold771	18511	18756
BmMITE-10	9	258	nscaf2931	2126260	2126034
BmMITE-10	10	254	nscaf2204	3536876	3537300
BmMITE-10	11	248	nscaf2993	4644338	4644573
BmMITE-10	11	258	nscaf3026	5717056	5717306
BmMITE-10	11	256	nscaf2880	10794	10549
BmMITE-10	14	258	nscaf2836	469920	470161
BmMITE-10	15	258	nscaf2847	8571670	8571915
BmMITE-10	16	258	nscaf3040	4438120	4438359
BmMITE-10	18	258	nscaf2998	313974	313673
BmMITE-10	22	257	nscaf2655	781172	780937
BmMITE-10	26	258	nscaf2829	129379	129588
BmMITE-10	34	258	nscaf1898	12542116	12541895
BmMITE-10	34	258	nscaf2842	855688	855908

BmMITE-10	1	257	nscaf2853	5998328	5998075
BmMITE-10	1	257	nscaf2852	3875	3621
BmMITE-10	1	257	nscaf2847	4121254	4120999
BmMITE-10	1	257	nscaf2860	516553	516805
BmMITE-10	1	257	nscaf2912	65347	65095
BmMITE-10	1	257	nscaf2902	4830293	4830545
BmMITE-10	1	257	nscaf1898	13234153	13233915
BmMITE-10	1	257	nscaf2330	1450694	1450443
BmMITE-10	1	257	nscaf3026	6177555	6177809
BmMITE-10	1	257	nscaf3055	1244253	1243999
BmMITE-10	1	257	nscaf2916	135535	135224
BmMITE-10	1	257	nscaf3027	870742	870995
BmMITE-10	1	257	nscaf2800	1894713	1894962
BmMITE-10	1	257	nscaf3099	688734	688194
BmMITE-10	1	257	nscaf2789	537435	536838
BmMITE-10	1	257	nscaf2986	4139117	4139361
BmMITE-10	1	257	nscaf2889	1883167	1883453
BmMITE-10	1	257	nscaf2686	970316	969681
BmMITE-10	1	257	nscaf2890	1460714	1460476
BmMITE-10	1	256	nscaf2889	960862	960605
BmMITE-10	1	256	nscaf2902	5884997	5884748
BmMITE-10	1	256	nscaf2853	4883829	4884079
BmMITE-10	1	256	nscaf2937	391941	391311
BmMITE-10	1	256	nscaf2993	5218906	5218646
BmMITE-10	1	256	nscaf2795	3175354	3175609
BmMITE-10	1	256	nscaf3041	1556003	1556253
BmMITE-10	1	256	nscaf2948	418386	418026
BmMITE-10	1	256	nscaf2860	923058	922817
BmMITE-10	1	255	nscaf2902	9313849	9314102
BmMITE-10	1	255	nscaf2674	3106351	3106101
BmMITE-10	1	255	nscaf2887	1985866	1985615
BmMITE-10	1	255	nscaf2839	531208	531460
BmMITE-10	1	255	nscaf2575	1132856	1133134
BmMITE-10	1	255	nscaf2655	981117	981375
BmMITE-10	1	255	nscaf2954	199445	199200
BmMITE-10	1	254	nscaf3058	8104587	8104340
BmMITE-10	1	254	nscaf2529	4135086	4134834
BmMITE-10	1	254	nscaf2865	3235879	3236131
BmMITE-10	1	254	nscaf2800	1862585	1862826
BmMITE-10	1	254	nscaf2767	1847063	1847313
BmMITE-10	1	254	nscaf2888	4845504	4845754
BmMITE-10	1	254	nscaf2828	3184145	3183890
BmMITE-10	1	254	nscaf3055	853877	854105

BmMITE-10	1	254	nscaf2891	1328107	1328612
BmMITE-10	1	253	nscaf2529	406633	406386
BmMITE-10	1	253	nscaf2847	3575219	3575464
BmMITE-10	1	253	scaffold172	2470	1796
BmMITE-10	1	253	nscaf1898	14930057	14930305
BmMITE-10	1	253	nscaf2800	670780	671010
BmMITE-10	1	253	nscaf2855	3743778	3743540
BmMITE-10	1	252	nscaf2301	11180	10511
BmMITE-10	1	252	nscaf2204	3613942	3613550
BmMITE-10	1	252	nscaf3058	7246488	7246239
BmMITE-10	1	252	nscaf3040	1140653	1140898
BmMITE-10	1	251	nscaf2930	1054831	1054588
BmMITE-10	1	250	nscaf3031	2057160	2057406
BmMITE-10	1	250	nscaf1690	1524534	1524779
BmMITE-10	1	250	nscaf2951	613717	613468
BmMITE-10	1	250	nscaf2859	1749501	1749231
BmMITE-10	1	248	nscaf3058	4876786	4876539
BmMITE-10	1	248	nscaf2813	506219	506458
BmMITE-10	1	248	scaffold148	1275	1518
BmMITE-10	1	245	nscaf2912	204141	204387
BmMITE-10	1	243	nscaf2883	1333430	1333670
BmMITE-10	1	243	nscaf2902	3437607	3437363
BmMITE-10	1	226	nscaf3058	7559076	7559301
BmMITE-10	1	226	nscaf2767	4297067	4297294
BmMITE-10	1	226	nscaf2795	2807934	2808154
BmMITE-10	1	226	nscaf2853	2554963	2555184
BmMITE-11	1	233	nscaf2795	1497788	1497973
BmMITE-12	2	210	scaffold244	95	295
BmMITE-12	2	197	nscaf2888	8539441	8539632
BmMITE-12	3	210	nscaf2828	1083745	1083950
BmMITE-12	3	210	nscaf2828	5919516	5919721
BmMITE-12	4	210	nscaf2943	1938840	1939038
BmMITE-12	4	210	nscaf2511	4885641	4885453
BmMITE-12	5	210	nscaf2891	1692359	1692162
BmMITE-12	5	210	scaffold166	3819	3622
BmMITE-12	5	210	nscaf2859	376696	376495
BmMITE-12	5	210	nscaf2912	1357999	1358194
BmMITE-12	17	210	nscaf2823	969591	969780
BmMITE-12	25	210	nscaf2865	1269139	1269320
BmMITE-12	1	203	scaffold129	435	632
BmMITE-12	1	199	scaffold389	203	2

BmMITE-12	1	198	scaffold289	201	1
BmMITE-12	1	188	scaffold125	184	1
BmMITE-12	1	185	scaffold208	182	1
BmMITE-12	1	180	scaffold364	179	3
BmMITE-12	1	178	scaffold258	606	783
BmMITE-12	1	175	scaffold236	174	1
BmMITE-13	2	243	nscaf2964	3783637	3783396
BmMITE-13	2	216	nscaf2770	2424666	2424451
BmMITE-13	3	263	nscaf3034	3499464	3499204
BmMITE-13	3	249	nscaf2136	123405	123159
BmMITE-13	4	263	nscaf3015	1404951	1405210
BmMITE-13	4	263	nscaf1071	342744	342487
BmMITE-13	4	249	nscaf1898	11519563	11519318
BmMITE-13	5	263	nscaf3099	3042022	3042280
BmMITE-13	5	249	nscaf2847	2134346	2134585
BmMITE-13	8	263	nscaf3086	3897	3642
BmMITE-13	35	263	nscaf3066	420361	420589
BmMITE-13	36	263	nscaf3099	4043459	4043232
BmMITE-13	39	263	nscaf2832	22907	22683
BmMITE-13	1	262	nscaf2589	416034	415773
BmMITE-13	1	262	nscaf1690	6600946	6600685
BmMITE-13	1	262	nscaf2822	429914	429653
BmMITE-13	1	262	nscaf3097	3051094	3051350
BmMITE-13	1	260	nscaf3099	3524910	3525182
BmMITE-13	1	259	nscaf2330	159761	160236
BmMITE-13	1	259	nscaf2767	573093	572836
BmMITE-13	1	259	nscaf1898	14175817	14176065
BmMITE-13	1	259	nscaf1898	15141184	15140925
BmMITE-13	1	259	nscaf2930	1849077	1849337
BmMITE-13	1	259	nscaf2681	613550	613799
BmMITE-13	1	259	nscaf1681	1646298	1646556
BmMITE-13	1	259	nscaf3031	1023993	1024248
BmMITE-13	1	259	nscaf3097	3074026	3073776
BmMITE-13	1	259	nscaf2204	4373780	4374034
BmMITE-13	1	259	nscaf2865	2491197	2491513
BmMITE-13	1	259	nscaf2826	96301	96551
BmMITE-13	1	259	nscaf2860	3037177	3037436
BmMITE-13	1	259	nscaf3099	1447235	1447895
BmMITE-13	1	259	nscaf2136	464667	464414
BmMITE-13	1	255	nscaf2954	26238	26492
BmMITE-13	1	250	nscaf2176	3464336	3464087
BmMITE-13	1	249	nscaf2951	1279636	1279887

BmMITE-13	1	249	nscaf3058	8930860	8930610
BmMITE-13	1	249	nscaf2809	103372	103622
BmMITE-13	1	249	nscaf3110	9757	9508
BmMITE-13	1	249	nscaf2902	5232139	5231912
BmMITE-13	1	243	nscaf2847	7900023	7899776
BmMITE-13	1	243	nscaf2930	1198887	1198650
BmMITE-13	1	243	nscaf2986	2731567	2731811
BmMITE-13	1	238	nscaf2993	7923059	7923295
BmMITE-13	1	237	nscaf3015	4268685	4268448
BmMITE-13	1	236	nscaf3099	4672050	4671815
BmMITE-13	1	236	nscaf3078	377968	378203
BmMITE-13	1	236	nscaf1821	544	778
BmMITE-13	1	235	nscaf2964	4490319	4490084
BmMITE-13	1	235	nscaf1898	8682891	8683126
BmMITE-13	1	235	nscaf2883	1351073	1350839
BmMITE-13	1	235	nscaf1681	2966505	2966269
BmMITE-13	1	235	nscaf3093	421425	421660
BmMITE-13	1	233	nscaf2883	2254515	2254747
BmMITE-13	1	233	nscaf2986	2281989	2281757
BmMITE-13	1	233	scaffold432	35	267
BmMITE-13	1	225	nscaf2176	3199486	3199259
BmMITE-13	1	223	nscaf2889	2106339	2106559
BmMITE-13	1	222	nscaf2855	1938391	1938169
BmMITE-13	1	222	nscaf2847	3837558	3837336
BmMITE-13	1	222	nscaf1898	6360633	6360420
BmMITE-13	1	222	nscaf2780	87468	87246
BmMITE-13	1	222	nscaf1690	3144014	3144235
BmMITE-13	1	222	nscaf3003	1522501	1522279
BmMITE-13	1	217	nscaf2886	1140192	1139972
BmMITE-13	1	216	nscaf3099	501752	501537
BmMITE-13	1	214	nscaf3079	381800	381596
BmMITE-13	1	214	nscaf1681	680777	680563
BmMITE-13	1	214	nscaf2865	4585429	4585643
BmMITE-14	2	429	nscaf1495	723	1421
BmMITE-14	3	431	nscaf2993	2115561	2115164
BmMITE-14	4	431	nscaf2847	2091769	2091339
BmMITE-14	5	431	nscaf2931	154013	155267
BmMITE-14	5	422	nscaf125	91284	90899
BmMITE-14	10	431	nscaf2795	812919	813281
BmMITE-14	1	430	nscaf2766	157854	157484
BmMITE-14	1	428	nscaf2912	945868	946530
BmMITE-14	1	428	nscaf2859	944167	943735

BmMITE-14	1	428	nscaf2759	944655	944028
BmMITE-14	1	383	scaffold896	33	783
BmMITE-14	1	366	nscaf3008	707660	708011
BmMITE-16	4	265	nscaf2883	2291813	2292096
BmMITE-16	5	265	nscaf3026	4922858	4922574
BmMITE-16	11	243	nscaf3026	2563131	2563656
BmMITE-16	18	265	nscaf3003	550860	550590
BmMITE-16	23	265	nscaf3013	1061688	1061937
BmMITE-16	31	265	nscaf2829	4157098	4157636
BmMITE-16	31	263	nscaf1690	1437984	1437732
BmMITE-16	31	265	nscaf2136	5833760	5833364
BmMITE-16	31	265	nscaf2795	1683644	1683299
BmMITE-16	36	265	nscaf3071	898831	898579
BmMITE-16	43	265	nscaf3003	3437287	3437049
BmMITE-16	46	265	nscaf481	1001706	1002229
BmMITE-16	48	265	nscaf2681	1205513	1205733
BmMITE-16	51	262	nscaf2948	402280	402047
BmMITE-16	1	263	nscaf2868	170945	171329
BmMITE-16	1	261	nscaf1681	1174940	1174657
BmMITE-16	1	261	nscaf605	1273	111
BmMITE-16	1	137	nscaf2176	460828	460691
BmMITE-17	2	422	nscaf2903	332333	332746
BmMITE-17	3	494	nscaf2623	1851432	1850470
BmMITE-17	4	494	nscaf2888	7230741	7231224
BmMITE-17	4	484	scaffold173	527	55
BmMITE-17	4	494	nscaf2767	3157310	3156861
BmMITE-17	5	494	nscaf2176	830588	831062
BmMITE-17	11	494	scaffold320	1	477
BmMITE-17	19	494	nscaf2986	2867886	2868338
BmMITE-17	1	489	nscaf2674	680015	679536
BmMITE-17	1	489	nscaf2797	1113679	1113217
BmMITE-17	1	489	nscaf3027	1191117	1191592
BmMITE-17	1	487	nscaf3099	2828947	2829425
BmMITE-17	1	487	nscaf2330	3139215	3139686
BmMITE-17	1	487	nscaf3026	3765285	3765770
BmMITE-17	1	487	nscaf1016	3647	3172
BmMITE-17	1	486	nscaf3141	793	368
BmMITE-17	1	475	nscaf2759	415903	416363
BmMITE-17	1	475	scaffold111	4131	3509
BmMITE-17	1	461	nscaf1898	13319911	13320367
BmMITE-17	1	461	nscaf3027	1457001	1457442

-
-
+
-
+
-
+
+
+
-
+
-

+
+
+
+
+
+

+
+
+

+
+
+
+
-
-
+
-
+
+
+
-
-
+
-
+
+
-
-
+
-
-
+
-
+
-
+
+
+
+
+
-
-
+
-
-
+
-
-

+
-
-
-
+
-
-
+
+
+
-
+
-
-

-
-
+
-
-
-
+
-
+
-
+

+
-
-
-
-
-
+

+
-
+
+
-
-
-
+

+
-
+
+
+
+
-
-
+
-
+
-
+
+
+
+
+
+
-
+
-
+
-
-
-
-
+
+
+
-
+
-
-
-
-
-
-
+
+
-
+
-
-

-
-
-
+
+
-
-
-
-
-
+
+
-
+
+
-
-
+
+
-
-
-
+
-
-
-
-
-
+
-
-
+
+
+
-
-
-
-
+
+
+
-
-

-
-
-
+
+
-
+
+
-
+
-
+
+
-
+
+
-
-
+
+
+
-
+
+
-
-
+
+
+
-
+
+
+
-
+
+
+
+
-
+
+
-
-

-
+
+
-
+
+
-
-
+
+
-
-
+
-
-
+
+
-
-
-
-
-
-
-
-
-
-
+
-
+
-
-
-
+
-
+
-
+
+
+
+
+
+

+
+
+
-
-
+
-
-
-
+
-
+
-
+
-
+
+
-
+
+
+
+
-
-
-
+
+
+
+
+
-
-
-
+
+
-
+
-
+
-
+
-
+

+

-

+

-

-

-

-

-

+

-

+

-

+

+

+

-

+

+

-

+

-

-

-

-

-

+

-

-

+

-

+

-

+

-

-

+

+

-

+

+

-

-

-

-
+
-
-
-
+
+
-
-
-
+
+
-
-
-
+
+
-
+
-
-
+
-
+
-
-
-
-
-
+
+
+
-
+
+
+
-
-
+
+
+
-
+

+
-
+
+
+
+
+
-
-
+
+
+
-
+
-
-
-
-
-
+
+
+
+
+
-
-
+
-
-
-
-
-
-
-
-
+
+
+
+
-
-
+
-

+
-
+
+
+
+
-
-
+
-
+
-
+
+
-
-
+
+
-
-
+
-
+
-
-
+
+
+
+
+
+
-
+
+
+
+
+
-
+
+
-
+
-

-
+
+
-
+
-
+
-
+
-
+
-
-
+
+
+
+
-
-
-
+
-
-
+
+
+
+
-
-
+
+
-
+
-
+
-
+
+
+
-
-
-
-

+
+
-
-
-
+
+
-
-
+
-
+
+
+
+
-
-
+
-
+
+
-
+
+
-
+
-
+
+
+
+
+
-
-
-
-
+
-
-
-
+
+
+

-
-
-
-
+
-
+
-
-
-
+
-
+
+
-
+
-
-
+
+
+
-
-
+
+
-
+
-
+
-
+
-
+
-
+
-
-
+
-
+
+
+
+

-
-
-
-
+
+
-
+
-
-
+
-
-
+
-
+
-
+
+
-
-
+
+
-
+
+
+
+
+
-
-
+
-
-
-
+
+
+
+
+
-
+
+

-
-
-
+
-
+
+
+
-
+
-
+
+
+
-
+
+
+
+
-
-
-
+
+
+
-
+
+
+
-
-
+
+
-
-
-
+
-
+
-
-
-
+

+
-
+
+
+
-
+
-
-
-
-
-
+
-
+
+
-
+
-
+
-
-
-
-
-
+
+
+
-
-
-
-
-
-
-
-
-
-
-
-
+
-
-

-
+
-
-
-
+
+
+
+
+
-
-
+
-
-
-
-
+
+
+
+
+
+
+
-
+
+
+
+
-
-
+
-
+
-
-
-
-
+
+
+
+
+

-
-
-
-
+
-
+
-
-
-
-
+
+
+
-
-
+
-
-
-
-
-
-
+
-
+
-
-
-
+
+
-
+
-
-
+
-
+
+
+
-
-
-

+

+

-

-

+

+

-

+

-

-

+

-

+

+

-

+

+

-

+

-

-

-

-

-

-

-

+

+

-

-

+

-

+

+

+

+

+

-

-

+

+

-

-

+
-
-
-
-
+
-
-
+
-
-
-
-
-
+
-
-
+
-
+
-
+
-
+
+
-
+
-
+
-
-
+
-
+
-
-
-
+
+
+
-
-
-

+
-
-
-
+
+
-
+
+
-
+
+
+
+
+
-
+
+
+
-
-
-
+
+
+
-
+
-
+
-
+
-
+
+
+
+
+
-
-
-
-
-
-

-
-
-
+
-
-
+
-
-
-
-
+
+
+
-
+
-
-
+
+
-
-
+
+
-
+
-
-
+
+
+
+
-
+
-
-
-
+
+
-
-
-
+

-
+
+
-
-
+
+
-
-
+
+
+
+
-
-
-
-
+
+
-
+
+
-
+
+
+
+
-
-
-
-
-
+
-
+
-
-
-
+
-
+
-
+

+
+
-
+
+
+
-
-
-
+
+
-
+
+
-
-
-
-
+
+
-
-
-
+
-
-
+
-
+
-
+
-
+
-
-
+
-
-
-
+
+
+
+

+
-
-
+
-
+
+
-
+
+
-
-
-
-
-
-
-
-
-
-
-
+
+
-
+
+
+
+
+
-
-
+
-
-
+
+
-
-
+
+
+
+
-

-
-
+
-
+
+
+
-
+
-
-
+
+
+
+
+
-
-
+
+
-
+
-
+
-
+
-
+
+
-
+
+
-
+
+
-
-
-
-
+
+
+
+

-
+
+
-
-
-
+
+
+
-
+
+
+
-
+
-
-
+
+
-
+
-
-
-
+
+
+
+
-
+
+
+
-
+
+
-
+
+
-
-
+
+
+

+

+

-

+

+

+

+

-

-

-

-

+

-

+

-

-

-

-

-

-

+

-

+

+

-

+

+

+

+

+

-

-

+

+

+

-

-

+

+

+

-

+

-

+
-
+
+
+
+
+
-
-
+
+
-
+
+
-
-
+
+
-
-
-
-
+
+
-
-
-
+
-
-
+
+
+
+
-
-
-
+
-
-
+
+
-

+
+
-
+
+
+
+
+
-
-
-
+
+
+
-
+
+
+
-
+
+
+
+
+
+
-
+
-
+
+
-
+
+
+
+
+
-
-
+
-
+
-
+

-
+
-
+
+
+
-
-
-
-
-
-
+
+
-
+
-
+
-
-
-
-
-
-
+
-
-
-
-
+
+
+
+
+
-
+
-
+
+
-
+
-
-

+
+
-
-
+
-
+
-
+
+
-
+
+
+
+
+
+
+
-
-
-
+
-
+
-
+
-
-
-
-
-
+
-
-
-
+
-
+
-
-
-
+
-

+
-
+
+
-
-
+
-
+
-
+
-
+
-
+
+
+
-
+
+
+
-

-
+
-
+
-
-
-
+
-
-
-
-
+
-
-
-
+
-
-
+

-
+
+
+
+
+
-
-
-
-
+
-
+
+
+
-
-
-
+
-
-
+
-
-
+
+
-
+
+
-
-
-
-
-
+
+
+
+
-
-
-
+
+

+
-
+
+
+
-
+
+
+
+
-
+

-
+
-
-
-
+
+
-
+
+
-
-
-
-
+
-
+
-
+
-
-
+
-
+
+
+
+
+
-
-

+
-
+
+
-
+
-
-
+
-
-
+
-
+
+
-
+
+
-
+
+
-
+
-
+
+
-
-
+
+
-
+
+
+
-
+
+
+
-
-
+
-
+

-

-

-

+

-

+

-

-

+

-

-

+

+

-

-

+

+

-

-

-

-

+

-

-

+

+

-

-

+

-

-

+

+

+

-

-

-

+

+

+

+

-

+

+
-
+
-
+
+
-
-
-
-
+
-
+
+
-
-
-
+
+
+
+
-
+
+
+
+

+

+
+
+
+
+
-
-
-
-
+
+
+
+
-

-
-
-
-
+
-

-
-
-
-
+
-
-
+
+
-
+
-
-
-
-
-
+
+
+
-
+
-
+
+
+
+
-
+
+
+
+
+
-
+
-
+

-
+
-
-
-
-
+
+
-
-
+
+
-
+
-
-
+
+
-
+
-
+
-
-
-
-
+
-
-
-
-
-
+

+
-
-
+
-
+
-
+
-

-
+
+

+
-
+
-
+
+
-
-
-
-
-
+
+
-
+
-
-
-

+
-
+
-
-
+
+
+
-
-
+
+
+
+
-
-
+
-
+
+
