# Supplementary figures and images for: Burst expansion, distribution and diversification of MITEs in the silkworm genome
Source: BMC Genomics. 2010 Sep 27;11:520. doi: 10.1186/1471-2164-11-520 (PMC2997013; doi:10.1186/1471-2164-11-520)

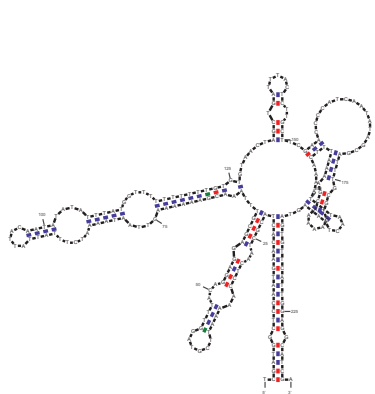

1

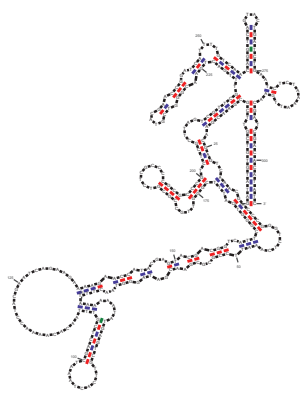

2

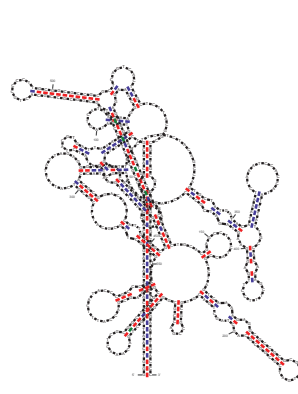

3

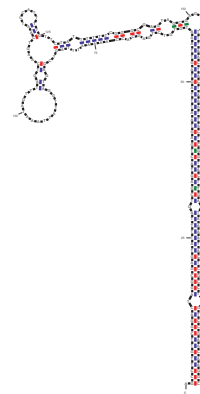

4

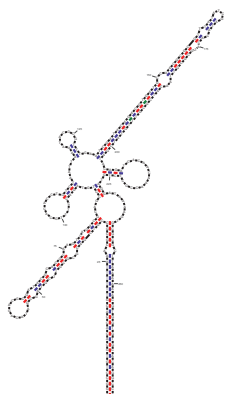

5

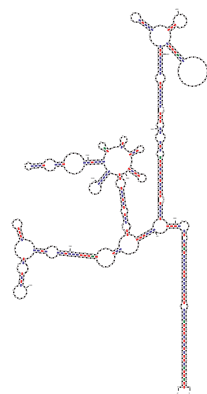

6

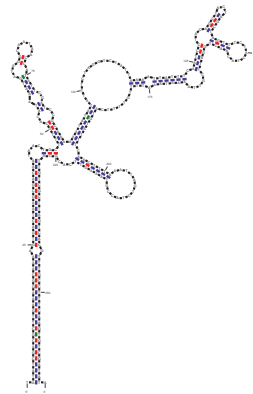

7

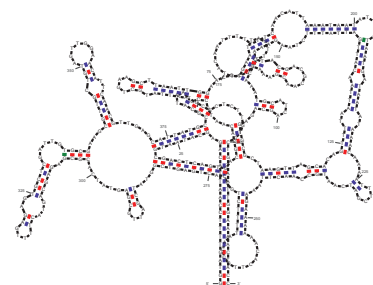

8

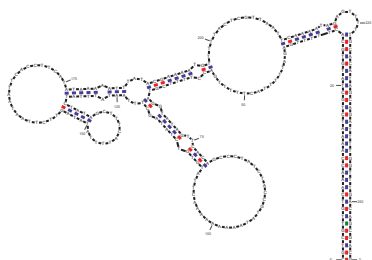

9

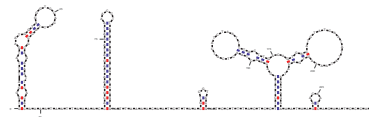

10

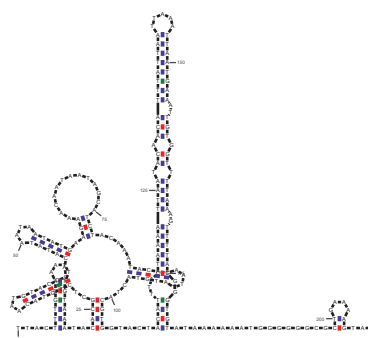

11

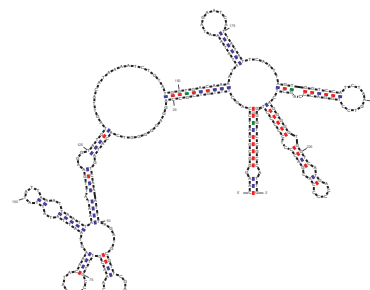

12

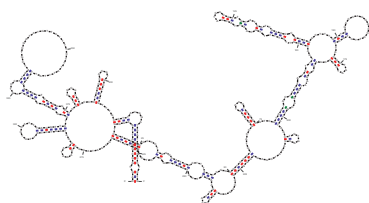

13

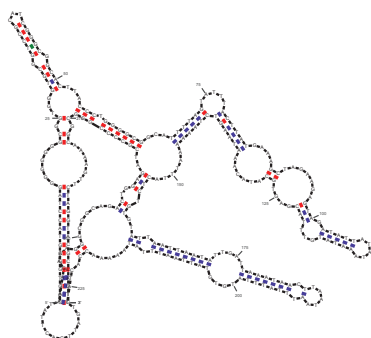

14

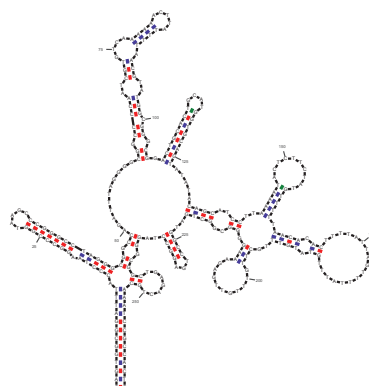

15

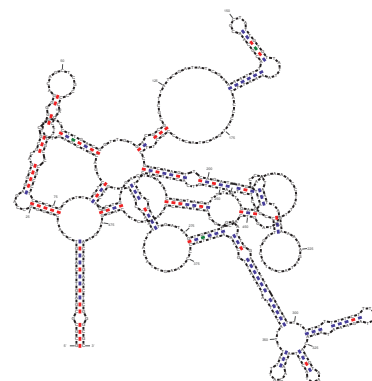

16

Supplement: Additional file 3 — The secondary structures of silkworm MITEs predicted by the program of UNADOLD: 1: BmMITE-1,2-16: BmMITE-3-17, respectively. [file 1471-2164-11-520-S3.PDF]

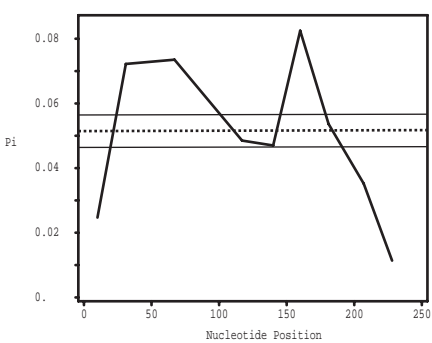

BmMITE-1

A

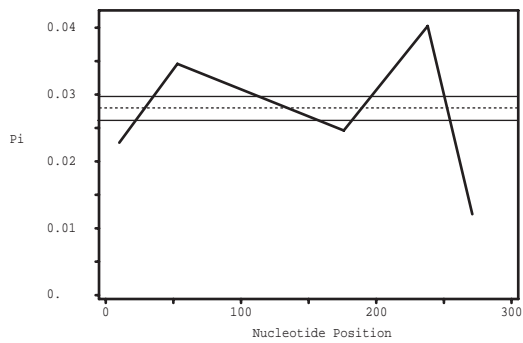

BmMITE-2

B

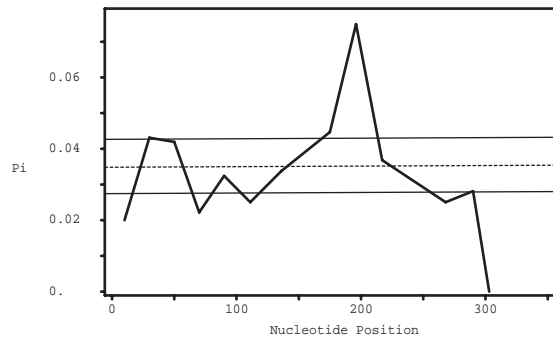

BmMITE-3

C

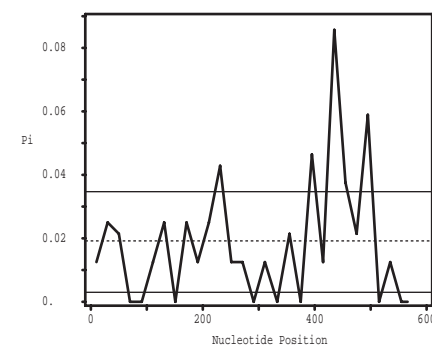

BmMITE-4

D

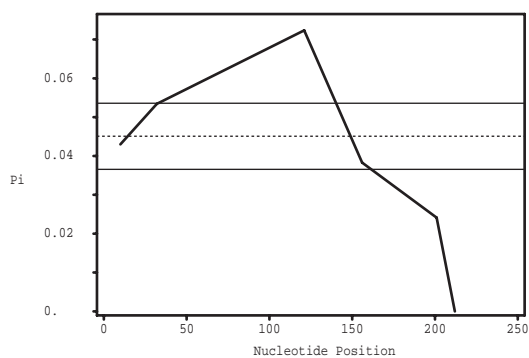

BmMITE-5

E

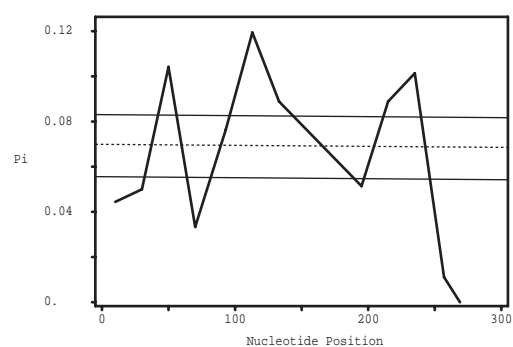

BmMITE-6

F

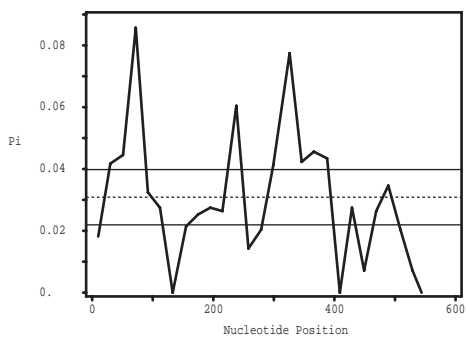

BmMITE-7

G

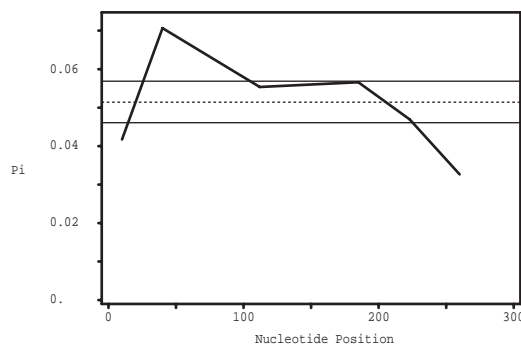

BmMITE-8

H

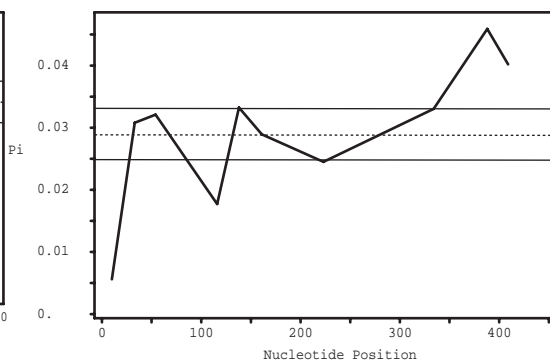

BmMITE-9

I

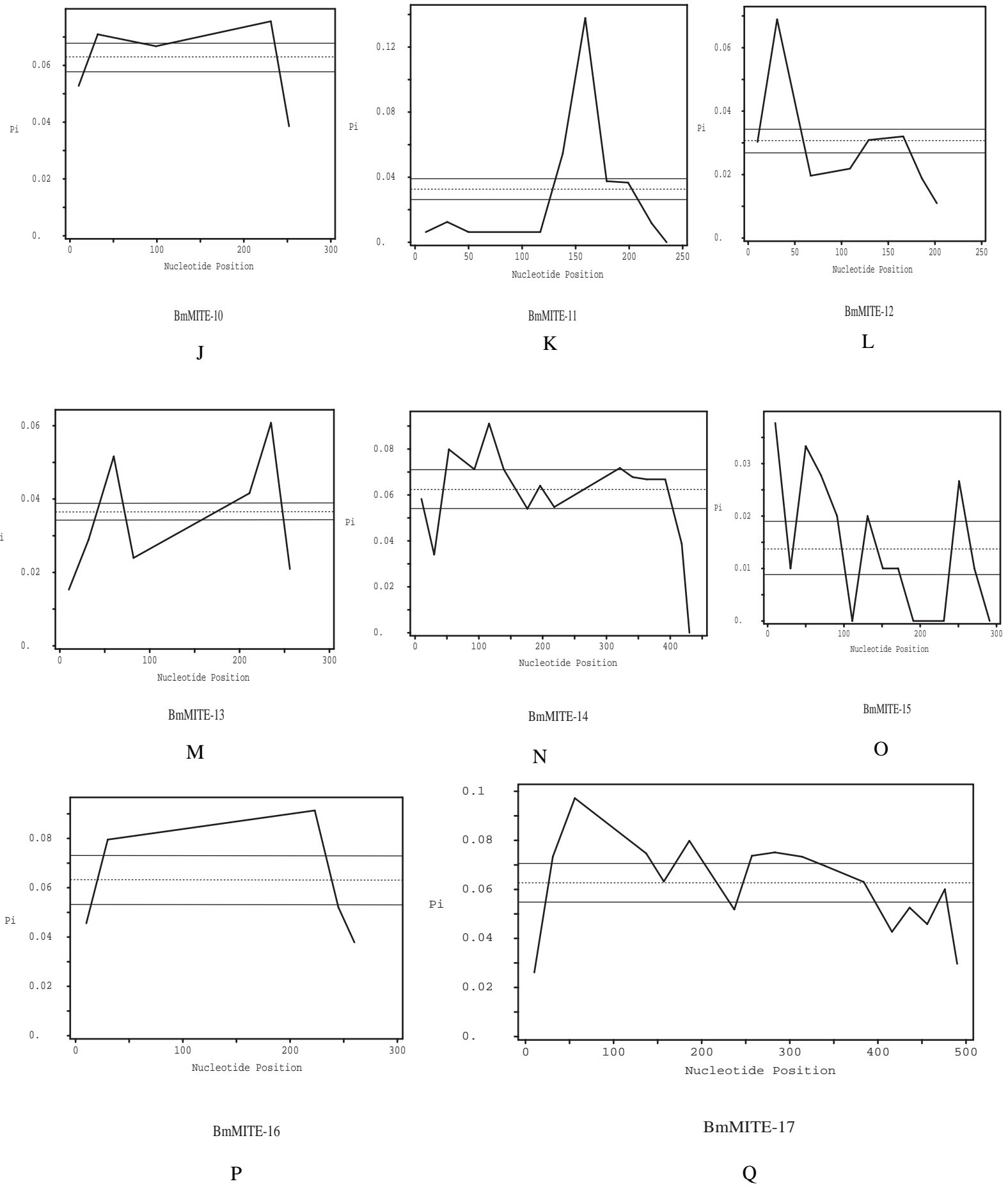

Figure S3.

Supplement: Additional file 7 — Nucleotide variation (π) along MITE sequence for each family. Nucleotide diversity π and its standard deviation were calculated by the program of DnaSP version 5.10. Then, the same program was used to define conserved and variable of the MITE sequences by sliding window analysis with both window size and step size 20 nucleotides. Windows with diversity equal or higher than average sequence diversity (π) + 2SD were defined as variable. Those with diversity less than average sequence diversity (π) - 2SD were considered as conserved. [file 1471-2164-11-520-S7.PDF]

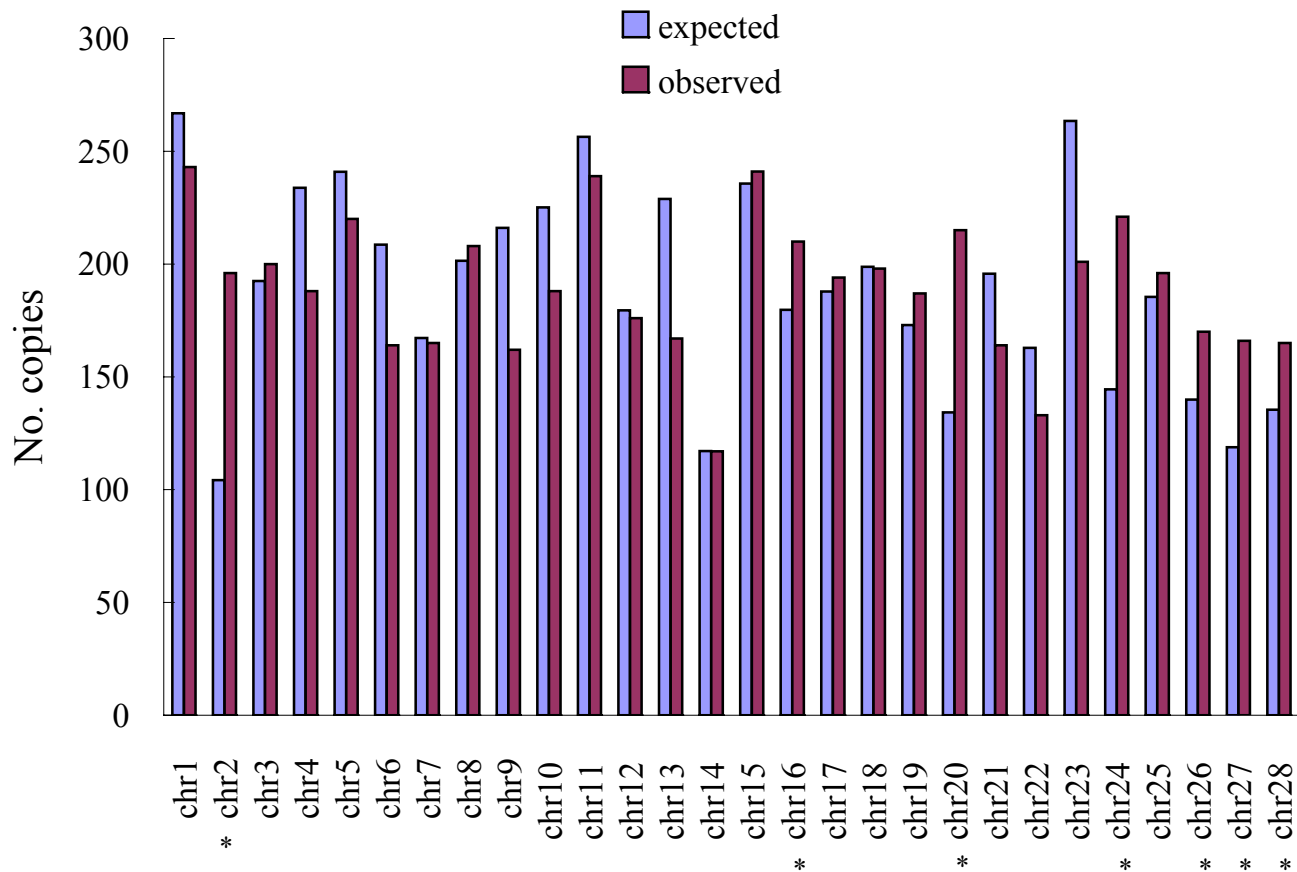

silkmoth chromosomes

Supplement: Additional file 8 — Distribution of the silkworm MITEs on the 28 chromosomes. The observed distribution is significantly different from the expected one based on the total length of 28 chromosomes (Chi-square = 297, df = 27, P < 0.01). *The chromosomes that show the observed copies more than expected. [file 1471-2164-11-520-S8.PDF]

# Gene Ontology Annotation

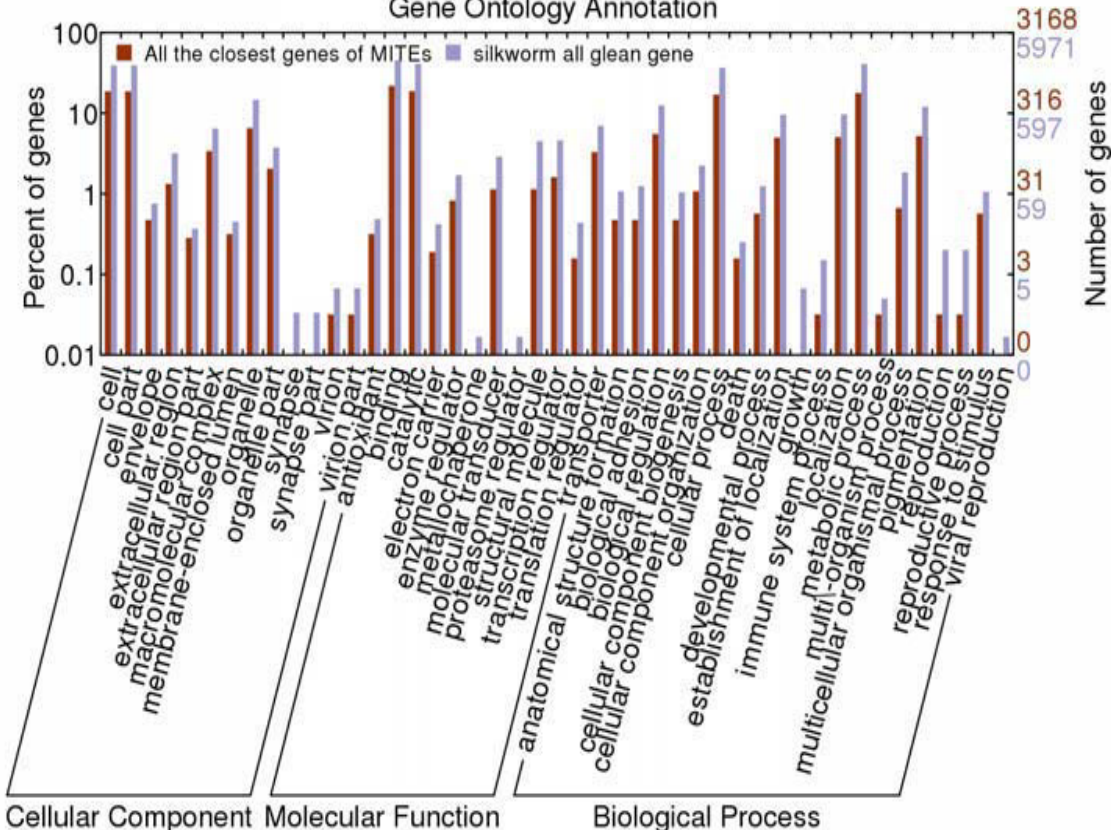

Supplement: Additional file 10 — The annotation of the closest genes using WEGO. [file 1471-2164-11-520-S10.PDF]
