# Supplementary material for: Burst expansion, distribution and diversification of MITEs in the silkworm genome
Source: BMC Genomics. 2010 Sep 27;11:520. doi: 10.1186/1471-2164-11-520 (PMC2997013; doi:10.1186/1471-2164-11-520)
Supplement: Additional file 4 — Primers for PCR verification of BmMITE-2; [file 1471-2164-11-520-S4.DOC]

**Primers for PCR verification of BmMITE-2**.

| **Locus** | **Primer sequence (5`-3`)** |
| --- | --- |
| Insertion site1 | F: ATGTGAGCCCGCTTGAATAG |
|  | R: GACCAGCACTTTGTTATCGAC |
| Insertion site2 | F: GGTTTCAGAACTGCAAGCAT |
|  | R: TCGTTCGCAACAAGATGG |
| Insertion site3 | F: GTGGGAAAATACCCCTCAGC |
|  | R: AAATACAGTCCACCCACC |
| Insertion site4 | F: AGCGTCTTCGGGTAGTATGG |
|  | R: GCGACTGTTAAGCTCTGAGTAC |
