# Supplementary material for: Burst expansion, distribution and diversification of MITEs in the silkworm genome
Source: BMC Genomics. 2010 Sep 27;11:520. doi: 10.1186/1471-2164-11-520 (PMC2997013; doi:10.1186/1471-2164-11-520)
Supplement: Additional file 5 — The results for PCR verification of predicted BmMITE-2. [file 1471-2164-11-520-S5.DOC]

**The results for PCR verification of predicted BmMITE-2**

|  |  | **Predicted BmMITE-2** | |  |
| --- | --- | --- | --- | --- |
| **Strains** | **Insertion site 1** | **Insertion site 2** | **Insertion site 3** | **Insertion site 4** |
| Wu-B | + | + | - | - |
| Wu-D | + | + | - | + |
| DongDe-201 | + | + | - | + |
| Wu-E | + | + | - | + |
| Lu-10 | - | + | - | + |
| DaXianTuZhong | - | + | - | + |
| FuRongHuiLuan | + | + | - | + |
| PeiXianZhong | + | + | - | - |
| Ri-9 | + | + | - | + |
| HeiZi | + | + | - | + |
| ChunSi | - | + | - | + |
| QiongShanHaiNan | + | + | - | + |
| Ri-110 | + | + | - | + |
| ShangSanHuBan | + | + | + | + |

+: BmMITE-2 occupied at this genomic location. –: The BmMITE-2 lack at this genomic location.
