# Supplementary material for: Burst expansion, distribution and diversification of MITEs in the silkworm genome
Source: BMC Genomics. 2010 Sep 27;11:520. doi: 10.1186/1471-2164-11-520 (PMC2997013; doi:10.1186/1471-2164-11-520)
Supplement: Additional file 9 — MITE distances to the nearest genes. [file 1471-2164-11-520-S9.DOC]

**Table 2. Characteristics of insertion sites of the silkworm MITEs.**

| Family | No.analysis | Insert into | | | | | |
| --- | --- | --- | --- | --- | --- | --- | --- |
|  |  | Exon | Intron | <500 | 500-3000 | 3000-5000 | Total |
| BmMITE-1 | 260 | 3 | 53 | 11 | 57 | 31 | 155 |
| BmMITE-2 | 2790 | 15 | 651 | 147 | 580 | 398 | 1791 |
| BmMITE-3 | 46 | 0 | 16 | 2 | 12 | 13 | 43 |
| BmMITE-4 | 16 | 2 | 3 | 1 | 5 | 4 | 15 |
| BmMITE-5 | 134 | 8 | 26 | 16 | 21 | 10 | 81 |
| BmMITE-6 | 19 | 4 | 6 | 1 | 4 | 1 | 16 |
| BmMITE-7 | 20 | 0 | 8 | 1 | 5 | 2 | 16 |
| BmMITE-8 | 1511 | 23 | 431 | 86 | 273 | 231 | 1044 |
| BmMITE-9 | 163 | 0 | 26 | 12 | 46 | 40 | 124 |
| BmMITE-10 | 301 | 0 | 96 | 17 | 41 | 31 | 185 |
| BmMITE-11 | 16 | 0 | 2 | 1 | 3 | 0 | 6 |
| BmMITE-12 | 141 | 0 | 16 | 5 | 15 | 19 | 55 |
| BmMITE-13 | 240 | 2 | 42 | 11 | 49 | 43 | 147 |
| BmMITE-14 | 34 | 0 | 16 | 4 | 5 | 5 | 30 |
| BmMITE-15 | 9 | 1 | 1 | 2 | 4 | 1 | 9 |
| BmMITE-16 | 43 | 1 | 9 | 4 | 14 | 6 | 34 |
| BmMITE-17 | 42 | 1 | 25 | 3 | 6 | 6 | 41 |
| Total | 5785 | 60 | 1427 | 324 | 1140 | 841 | 3792 |
| Control | 5000 | 152 | 453 | 253 | 810 | 302 | 1970 |
